# Global Burden of Musculoskeletal Disorders in Adults Aged 50 and Over, 1990–2021: Risk Factors and Sociodemographic Inequalities

**DOI:** 10.1002/jcsm.70008

**Published:** 2025-07-10

**Authors:** Shi‐Yang Guan, Jin‐Xin Zheng, Shun‐Xian Zhang, Shengqian Xu, Zongwen Shuai, Hong‐Yan Cai, Faming Pan

**Affiliations:** ^1^ Department of Epidemiology and Biostatistics, School of Public Health Anhui Medical University Hefei Anhui China; ^2^ Inflammation and Immune Mediated Diseases Laboratory of Anhui Province Hefei Anhui China; ^3^ School of Global Health, Chinese Center for Tropical Diseases Research Shanghai Jiao Tong University School of Medicine Shanghai China; ^4^ National Institute of Parasitic Diseases at the Chinese Center for Disease Control and Prevention & Chinese Center for Tropical Diseases Research WHO Collaborating Center for Tropical Diseases Shanghai China; ^5^ Clinical Research Center, Longhua Hospital Shanghai University of Traditional Chinese Medicine Shanghai China; ^6^ Department of Rheumatism and Immunity The First Affiliated Hospital of Anhui Medical University Hefei Anhui China; ^7^ Department of Nephrology Anhui No. 2 Provincial People's Hospital Hefei Anhui China

**Keywords:** global burden, inequalities, musculoskeletal disorders, temporal trends

## Abstract

**Background:**

Adults aged 50 and over are particularly vulnerable to musculoskeletal (MSK) disorders, with their impact expected to intensify as the global population ages. This study aims to comprehensively assess the global burden of MSK disorders among adults aged 50 and over from 1990 to 2021, as well as temporal trends, risk factors and sociodemographic inequalities.

**Methods:**

Data were sourced from the Global Burden of Disease 2021 study. Temporal trends in age‐standardized rates were evaluated by calculating average annual percent changes (AAPC). Absolute and relative inequalities were assessed using the slope index of inequality and concentration index, respectively.

**Results:**

From 1990 to 2021, MSK disorders remained the largest contributor to total years lived with disability (YLDs) among adults aged 50 and over globally. The global age‐standardized incidence rate significantly decreased (AAPC = −0.181, 95% CI: −0.190 to −0.172), whereas the global prevalence and disability‐adjusted life‐year (DALY) rates significantly increased (AAPC = 0.126, 95% CI: 0.118–0.134, and AAPC = 0.057, 95% CI: 0.042–0.072, respectively). High sociodemographic index (SDI) countries exhibited the highest age‐standardized incidence, prevalence and DALY rates (11 236.6, 56 308.1 and 5277.1 per 100 000 population, respectively), whereas low‐middle SDI countries showed the most rapid increases in prevalence and DALY rates (AAPC = 0.229, 95% CI: 0.218–0.240, and AAPC = 0.230, 95% CI: 0.204–0.256, respectively). Significantly positive associations were identified between SDI and age‐standardized incidence, prevalence and DALY rates (all *p* < 0.001). High body mass index (BMI) was the largest contributor to global DALYs of MSK disorders, accounting for 10.3% of the total in this population, whereas occupational ergonomic factors, smoking and kidney dysfunction contributed 7.3%, 6.0% and 0.2%, respectively. Although the proportions of DALYs due to occupational ergonomic factors and smoking declined globally (from 9.2% to 7.3% and from 8.9% to 6.0%, respectively), those due to high BMI and kidney dysfunction increased (from 7.7% to 10.3% and from 0.19% to 0.22%, respectively). Both absolute and relative SDI‐related inequalities persisted across 204 countries and territories, with no significant changes observed.

**Conclusions:**

MSK disorders have remained the largest contributor to disability among adults aged 50 and over. Despite significant progress in reducing the incidence rate, both the prevalence and DALY rates have significantly increased. With the expanding global ageing population, there is an urgent need for improved prevention strategies to mitigate the long‐term impacts of MSK disorders in this vulnerable population.

## 1 Introduction

Musculoskeletal (MSK) disorders comprise a broad array of conditions that primarily affect muscles, bones and joints, often leading to chronic pain and functional limitations [1, 2]. These disorders commonly manifest as stiffness, swelling and restricted mobility, which can significantly impair daily activities and increase susceptibility to other chronic illnesses, including cardiovascular diseases, metabolic disorders and mental health challenges [1, 3]. Beyond their direct effects on individual health, MSK disorders impose significant societal and economic burdens, particularly through increased healthcare expenditures and productivity losses due to long‐term disability and work absenteeism [4]. For instance, within the European Union, productivity losses linked to MSK disorders account for approximately 2% of gross domestic product [4]. In the United Kingdom, healthcare costs related to MSK disorders amount to 4.7 billion pounds, with major expenses stemming from drug therapies, rehabilitation and surgical interventions, making MSK disorders the third most costly condition in the National Health Service programme [5].

Adults aged 50 and over are especially vulnerable to MSK disorders due to the natural ageing process, which involves muscle mass decline, joint wear and tear, and metabolic changes [6, 7]. This demographic often faces coexisting chronic conditions, which exacerbate the effects of MSK disorders and further limit physical and social functioning [8, 9]. Additionally, age‐related hormonal changes, such as those experienced by postmenopausal women, can accelerate the onset and progression of MSK disorders like osteoarthritis and osteoporosis [6, 7]. As the global population continues to age, with individuals aged 50 and over representing an increasing share of societies, understanding the global burden of MSK disorders in this group has become more crucial than ever [10]. Despite the recognized impact of MSK disorders, there remains insufficient understanding of the epidemiological trends that shape the global burden of these disorders within this age group.

In light of this gap, the present study leverages data from the latest round of the Global Burden of Diseases (GBD) 2021 study to provide a comprehensive analysis of the global burden, temporal trends and risk factors for MSK disorders among adults aged 50 and over from 1990 to 2021. Moreover, this study evaluates sociodemographic inequalities associated with MSK disorders across 204 countries and territories. By offering an in‐depth evaluation of the epidemiological landscape and risk factors, this research aims to guide public health interventions and inform strategies to alleviate the global burden of MSK disorders in this vulnerable population.

## 2 Materials and Methods

### 2.1 Data Sources

This study utilized data from the GBD 2021 study, a large‐scale collaborative effort that tracks 371 diseases and injuries, along with 88 risk factors, across 204 countries and territories from 1990 to 2021 [8, 11]. Drawing on diverse data sources and advanced statistical methods, the GBD 2021 study offers detailed insights into both fatal and nonfatal health outcomes and tracks global health disparities [8, 11]. It covers a wide range of conditions, including infectious and noncommunicable diseases, mental health disorders, injuries and risk factors related to environment, behaviour and metabolism [8, 11]. The GBD 2021 study offers an essential framework for examining global health trends and disparities, providing comprehensive data that support the identification of key health challenges and informs strategies to improve health outcomes across diverse populations worldwide [8, 11].

In this study, data on the incidence, prevalence and disability‐adjusted life years (DALYs) for MSK disorders among adults aged 50 and over were extracted using the GBD Results Tool, an online platform providing access to data generated by the GBD 2021 study [8]. Estimates for incidence and prevalence were generated using the DisMod‐MR 2.1 Bayesian meta‐regression tool, which integrates all available data [8]. Years lived with disability (YLDs) were calculated by multiplying prevalence by disability weights [8], whereas years of life lost (YLLs) were determined by multiplying the death numbers by the standard life expectancy. Disability‐adjusted life years (DALYs) were obtained by summing YLDs and YLLs [8]. To account for uncertainty, uncertainty intervals (UIs) were calculated using the 2.5th and 97.5th percentiles of 1000 draws [8]. Details on case definitions, data sources and inclusion/exclusion criteria for MSK disorders were available in Table S1 and prior publications [8, 9].

### 2.2 Sociodemographic Index

To assess the sociodemographic context, this study employed the sociodemographic index (SDI), a composite measure of socioeconomic development. The SDI is derived from fertility rates in women under 25, average educational attainment for individuals aged 15 and older and per capita income [8]. Locations were assigned SDI scores ranging from 0.0 (least developed) to 1.0 (most developed) for each year and were categorized into quintiles: low‐SDI, low‐middle‐SDI, middle‐SDI, high‐middle‐SDI and high‐SDI [8]. Furthermore, the 204 countries and territories were grouped into 21 geographical regions to enable regional analysis, such as for East Asia [8].

### 2.3 Risk Factors for MSK Disorders

The GBD 2021 study also evaluated disease burden attributed to main risk factors for MSK disorders, including high body mass index (BMI), occupational ergonomic issues, smoking and kidney dysfunction across 204 countries and territories from 1990 to 2021 [11]. The impact of these risk factors was quantified by multiplying the total DALYs for MSK disorders by the population attributable fraction (PAF), which indicates the proportion of DALYs that could be reduced if exposure to the risk factor were minimized to an ideal level [11]. Data on occupational ergonomic risks were sourced from the International Labour Organization, smoking exposure data were obtained from individual‐level microdata and survey reports, and high BMI data were extracted from the Global Health Data Exchange (GHDx) [11]. Data on kidney dysfunction were gathered from systematic reviews and previous GBD studies [11]. Detailed definitions and methodologies for these risk factors were provided in Table S2 and prior publications [11].

### 2.4 Statistical Analysis

Age‐standardized incidence, prevalence and DALY rates for MSK disorders were calculated by aggregating data from 10 age subgroups (ranging from 50–55 to 95+ years) into a single 50+ group, applying the world population age structure as per the GBD 2021 study framework [8]. To assess temporal trends, the average annual percent change (AAPC) and corresponding 95% confidence intervals (CIs) were computed using joinpoint regression analysis [12]. The Joinpoint Regression Program (Version 5.2.0, National Cancer Institute, United States) was employed to identify significant trend changes, starting with no joinpoints (a straight‐line trend) and testing for additional joinpoints using the Monte Carlo permutation method [12]. The AAPC was calculated by weighting the annual percent change of each segment by its duration [12].

To quantify sociodemographic inequalities in MSK disorder burden, two key measures were utilized: the slope index of inequality (SII) and the concentration index (CI) [13, 14]. The SII measured absolute inequality in incidence, prevalence and DALY rates across countries and territories ranked by SDI [13, 14]. Each country or territory was weighted by population size, and regression analyses were performed using crude rates against SDI rankings [13, 14]. The SII represented the absolute difference between the predicted rates for the highest and lowest SDI‐ranked countries [13, 14]. The CI measured relative inequality by assessing the distribution of absolute numbers of incidence, prevalence and DALYs across SDI‐ranked countries [13, 14]. It was derived from the area between the concentration curve and the line of equality, with positive CI values indicating a concentration of absolute numbers in higher SDI countries, and negative values reflecting a concentration in lower SDI countries [13, 14].

Spearman's rank correlation was employed to explore associations between SDI and age‐standardized rates, as well as the disease burden attributable to risk factors, across 204 countries and territories. All statistical analyses and data visualizations were performed using R software (Version 4.2.3). A two‐sided *p* value of less than 0.05 was considered statistically significant.

## 3 Results

### 3.1 Global Burden of MSK Disorders Among Adults Aged 50 and Over, 2021

In 2021, MSK disorders accounted for 91 617 476 incident cases, 426 665 318 prevalent cases and 39 513 134 DALYs among adults aged 50 and over worldwide (Table S3). Within the major causes, MSK disorders were the largest contributor to global YLDs in this population, contributing to 21.9% of total YLDs, while ranked as the sixth leading contributor to global DALYs, accounting for 6.2% of the total (Figures S1 and S2). At the regional level, South Asia showed the highest numbers of incident cases, prevalent cases and DALYs (18 624 859, 88 551 905 and 7 901 461, respectively) (Table S3). Among 204 countries and territories, China reported the highest number of incident cases, prevalent cases and DALYs (43 140 113, 235 039 035 and 19 818 337, respectively) (Figures S3–S5 and Table S4).

In 2021, the global age‐standardized incidence, prevalence and DALY rates reached 9869.1, 50 848 and 4592.2 per 100 000 population for MSK disorders among adults aged 50 and over, respectively (Figure S6 and Table S5). Regionally, Eastern Europe displayed the highest age‐standardized incidence rate (13 391.3 per 100 000 population), whereas High‐income North America recorded the highest prevalence and DALY rates (61 332.5 and 5821.4 per 100 000 population, respectively) (Figure S6 and Table S5).

Across SDI quintiles, high SDI countries had the highest age‐standardized incidence, prevalence and DALY rates (11 236.6, 56 308.1, and 5277.1 per 100 000 population, respectively). Meanwhile, middle SDI countries exhibited the lowest age‐standardized incidence rate (9026.8 per 100 000 population), and low SDI countries showed the lowest prevalence and DALY rates (46 091.1 and 4178.7 per 100 000 population, respectively) (Figure S6 and Table S5).

**FIGURE 1:**
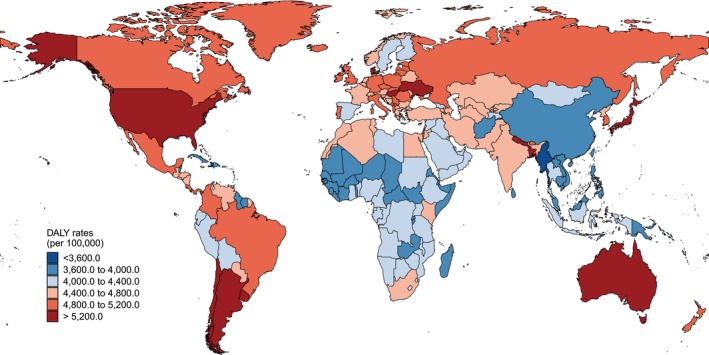
Age‐standardized DALY rates for MSK disorders among adults aged 50 and over across 204 countries and territories, 2021.

**FIGURE 2:**
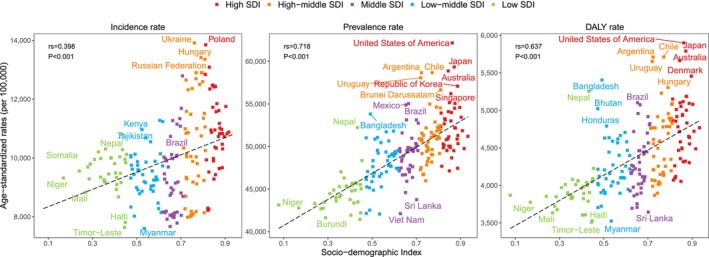
Association between SDI and age‐standardized incidence, prevalence and DALY rates for MSK disorders among adults aged 50 and over across 204 countries and territories, 2021.

At the national level, Ukraine had the highest age‐standardized incidence rate (13 915.7 per 100 000 population), whereas the United States presented the highest prevalence and DALY rates (62 166.0 and 5899.2 per 100 000 population, respectively) (Figures 1, S7 and S8 and Table S6). Additionally, Figure 2 illustrated significantly positive associations between SDI and age‐standardized incidence, prevalence and DALY rates across 204 countries and territories (all *p* < 0.001) (Figure 2).

Globally and across all SDI quintiles, women had higher age‐standardized incidence, prevalence and DALY rates for MSK disorders among adults aged 50 and over (Figure S9 and Table S7). Similar patterns were also observed across geographic regions. Furthermore, global age‐specific incidence and DALY rates increased with age, peaking in the 75–79 age group before declining, whereas global prevalence rates rose steadily with age, stabilizing after the 80–84 age group (Figure S10 and Table S8).

### 3.2 Temporal Trends of MSK Disorders Among Adults Aged 50 and Over, 1990–2021

From 1990 to 2021, global incident cases, prevalent cases and DALYs increased by 21.2%, 39.3% and 36.2% for MSK disorders among adults aged 50 and over, respectively (Table S3). Over this period, MSK disorders remained the largest contributor to total YLDs in this population, while declining their rank from fifth to sixth among the major causes of total DALYs (Figures S1 and S2). The numbers of incident cases, prevalent cases and DALYs increased across all geographic regions, with Central Latin America experiencing the highest percentage increases (215.6%, 232.9% and 234.3%, respectively) (Table S3). At the national level, the United Arab Emirates had the highest percentage increases (1283.8%, 1353.6% and 1347.2%, respectively), whereas Georgia showed the largest percentage decreases (−11.6%, −7.2% and −10.0%, respectively) (Figures S11–13 and Table S4).

**FIGURE 3:**
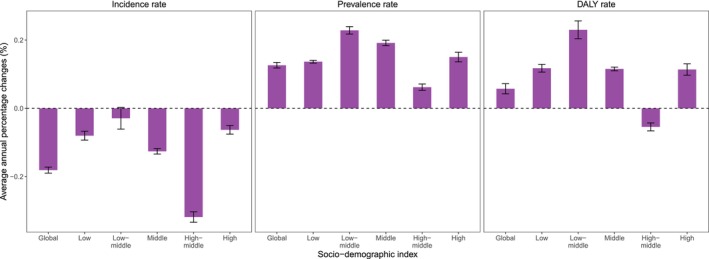
Average annual percentage changes of age‐standardized incidence, prevalence and DALY rates for MSK disorders among adults aged 50 and over globally and across SDI quintiles, 1990–2021.

Global age‐standardized incidence rate significantly decreased from 1990 to 2021 (AAPC = −0.181, −0.190 to −0.172), whereas global age‐standardized prevalence and DALY rates significantly increased (AAPC = 0.126, 0.118–0.134; 0.057, 0.042–0.072, respectively) (Figure 3 and Table S5). Across all SDI quintiles, age‐standardized incidence rates showed decreasing trends, with the steepest decrease in high‐middle SDI countries (AAPC = −0.319, −0.334 to −0.303) (Figure 3 and Table S5). Conversely, age‐standardized prevalence and DALY rates increased across most SDI quintiles, with the fastest increases observed in low‐middle SDI countries (AAPC = 0.229, 0.218–0.240; 0.230, 0.204 to 0.256, respectively) (Figure 3 and Table S5). Notably, the only decline in DALY rates was observed in high‐middle SDI countries (AAPC = −0.054, −0.066 to −0.043, respectively) (Figure 3 and Table S5).

At the regional level, age‐standardized incidence rates showed various trends, with Tropical Latin America showing the fastest increase, while East Asia exhibiting the most rapid decrease (AAPC = −0.163, 0.147–0.179; −0.319, −0.334 to −0.303, respectively) (Figure S14 and Table S5). However, age‐standardized prevalence and DALY rates increased across most regions, with the sharpest rises observed in North Africa and the Middle East for prevalence and in Central Latin America for DALYs (AAPC = 0.274, 0.261–0.287; 0.264, 0.250–0.278, respectively) (Figure S14 and Table S5).

Among 204 countries and territories, Sweden displayed the most rapid increase in the age‐standardized incidence rate (AAPC = 0.388, 0.359–0.416), whereas China showed the fastest decrease (AAPC = −0.288, −0.321 to −0.256) (Figure S15 and Table S6). Furthermore, Taiwan (Province of China) saw the fastest increases in age‐standardized prevalence and DALY rates (AAPC = 0.439, 0.403–0.474; 0.627, 0.607–0.647, respectively), whereas Burundi and Spain revealed the fastest decreases, respectively (AAPC = −0.143, −0.168 to −0.117; −0.274, −0.396 to −0.152) (Figures S16 and S17 and Table S6).

Globally and across most SDI quintiles, men experienced faster decreases in the age‐standardized incidence rate and more rapid increases in prevalence and DALY rates compared with women (Figure S18 and Table S7). However, in low and low‐middle countries, women exhibited faster increases in age‐standardized DALY rates (Figure S18 and Table S7). In addition, age‐specific incidence rates showed decreasing trends across most age groups, except for an increase in the 95+ age group. Conversely, most age groups exhibited increasing trends in age‐specific prevalence and DALY rates, with notable peaks in the 60–64 and 95+ age groups (Figure S19 and Table S8).

### 3.3 Risk Factors for MSK Disorders Among Adults Aged 50 and Over, 1990–2021

**FIGURE 4:**
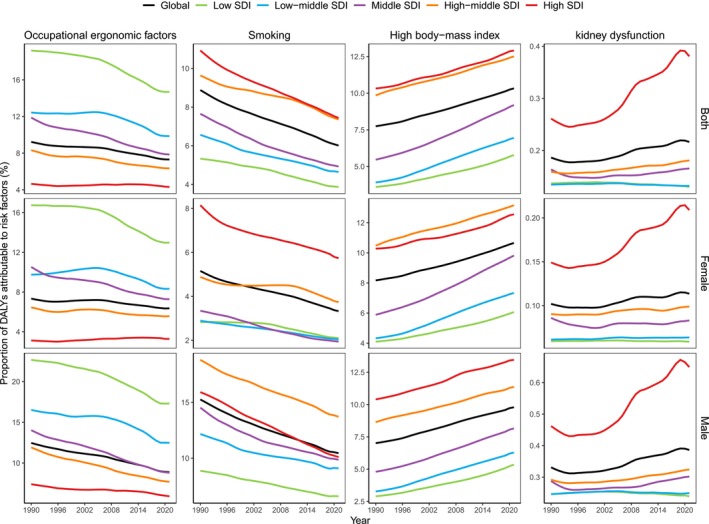
Proportions of DALYs attributable to high BMI, occupational ergonomic factors, smoking and kidney dysfunction for MSK disorders among adults aged 50 and over globally and across SDI quintiles, 1990–2021.

In 2021, 10.3%, 7.3%, 6.0% and 0.2% of global DALYs for MSK disorders among adults aged 50 and over were attributed to high BMI, occupational ergonomic factors, smoking and kidney dysfunction, respectively (Figure 4 and Table S9). Across SDI quintiles, low SDI countries showed the highest proportion of DALYs due to occupational ergonomic factors (14.7%), whereas high SDI countries had the lowest proportion (4.3%) (Figure 4). Moreover, high SDI countries displayed the highest proportions of DALYs due to high BMI, smoking and kidney dysfunction (12.9%, 7.4% and 0.4%), whereas low SDI countries had the lowest proportions (5.8%, 3.9% and 0.1%) (Figure 4 and Table S9). Furthermore, there was a significant negative association between SDI and the proportion of DALYs due to occupational ergonomic factors across 204 countries and territories. Conversely, significantly positive associations were observed between SDI and the proportions due to smoking, high BMI and kidney dysfunction, respectively (all *p* < 0.001) (Figure S20).

From 1990 to 2021, the proportions of DALYs due to occupational ergonomic factors and smoking declined globally and across most SDI quintiles, with global decreases from 9.2% to 7.3% for occupational ergonomic factors and from 8.9% to 6.0% for smoking (Figure 4). Conversely, the proportions of DALYs due to high BMI and kidney dysfunction increased globally and across most SDI quintiles, rising from 7.7% to 10.3% for high BMI and from 0.19% to 0.22% for kidney dysfunction (Figure 4 and Table S9). Notably, the proportion of DALYs attributed to occupational ergonomic factors remained stable in high‐SDI countries, whereas those due to kidney dysfunction exhibited steadiness in low‐ and low‐middle SDI countries (Figure 4 and Table S9).

In 2021, among geographic regions, Eastern Sub‐Saharan Africa and High‐Income North America had the highest proportions of DALYs due to occupational ergonomic factors and kidney dysfunction (20.4% and 0.6%), respectively, whereas Central Europe displayed the highest proportions due to high BMI and smoking (16.8% and 12.1%) (Figure S21 and Table S9). Among 204 countries and territories, Kingdom of Tonga, Republic of Mozambique, Montenegro and the United States showed the highest proportions of DALYs due to high BMI, occupational ergonomic factors, smoking and kidney dysfunction, respectively (19.9%, 30.4%, 16.6% and 0.6%) (Figures S22–S25 and Table S10).

Globally and among all SDI quintiles, men exhibited higher proportions of DALYs due to occupational ergonomic factors, smoking and kidney dysfunction compared with women (8.9% vs. 6.4% for occupational ergonomic factors, 10.4% vs. 3.3% for smoking and 0.4% vs. 0.1% for kidney dysfunction, globally) (Figure 4 and Table S11). In contrast, women revealed higher proportions of DALYs due to high BMI globally and among most SDI quintiles (10.7% vs. 9.8%, globally), except in high SDI countries, where men exhibited a higher proportion (13.5% vs. 12.6%) (Figure 4 and Table S11).

### 3.4 Inequalities for MSK Disorders Among Adults Aged 50 and Over, 1990–2021

**FIGURE 5:**
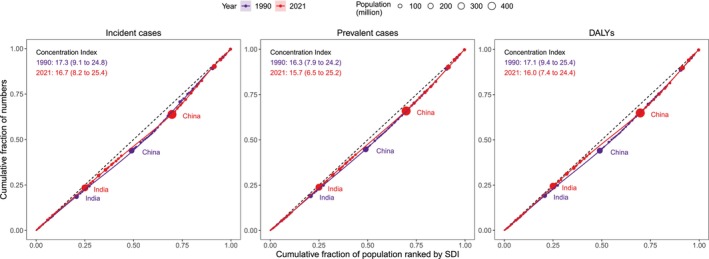
Relative SDI‐related inequalities in the numbers of incident cases, prevalent cases and DALYs for MSK disorders among adults aged 50 across 204 countries and territories, 1990–2021.

In 2021, MSK disorders showed significantly positive SII values in incidence, prevalence and DALY rates among adults aged 50 and over (SII = 2048.6, 10 831.0 and 1084.3, respectively), indicating significant absolute SDI‐related inequalities across 204 countries and territories (Figure S26). Furthermore, significantly positive CI values were observed in the numbers of incident cases, prevalent cases and DALYs (CI = 16.7, 15.7 and 16.0, respectively), highlighting significant relative sociodemographic inequalities (Figure 5). Between 1990 and 2021, both the absolute and relative SDI‐related inequalities persisted across 204 countries and territories, with no significant changes over time (Figures S26 and 5).

## 4 Discussion

To our knowledge, this study is the first to comprehensively assess the global burden, temporal trends, risk factors and sociodemographic inequalities related to MSK disorders among adults aged 50 and over across 204 countries and territories from 1990 to 2021. Our findings indicated that over the past three decades, the global age‐standardized incidence rate for MSK disorders in this population has shown a decreasing trend, whereas the global age‐standardized prevalence and DALY rates have revealed increasing trends. The decline in incidence rates may reflect improvements in early detection, prevention and management strategies. In contrast, the persistence of existing prevalent cases has likely contributed to rising prevalence and DALY rates, possibly due to improved survival rates and enhanced chronic disease management. In 2021, MSK disorders accounted for 91.6 million incident cases, 426.7 million prevalent cases and 39.5 million DALYs among adults aged 50 and over worldwide, reflecting significant increases of 21.2%, 39.3% and 36.2% since 1990, respectively. These increases can primarily be attributed to global population growth and the acceleration of ageing during this period [15]. These findings underscore the urgent need for improved prevention strategies to mitigate the long‐term impacts of MSK disorders, particularly as the ageing population continues to expand.

In 2021, high‐SDI countries exhibited the highest age‐standardized incidence, prevalence and DALY rates for MSK disorders among adults aged 50 and over. Conversely, middle‐SDI countries had the lowest incidence rates, whereas low‐SDI countries showed the lowest prevalence and DALY rates. These trends may be explained by the larger ageing populations and higher prevalences of risk factors, such as obesity and sedentary lifestyles, in high‐SDI countries [16]. Meanwhile, low‐ and middle‐SDI countries generally have smaller ageing populations and are likely affected by underreporting and limited access to healthcare, which can lead to lower recorded incidence and prevalence rates [16]. Additionally, the global age‐standardized incidence, prevalence and DALY rates were positively associated with SDI across the 204 countries and territories, aligning with previous studies on the general population [17].

From 1990 to 2021, age‐standardized incidence rates showed decreasing trends across all SDI quintiles, with the sharpest decline observed in high‐middle SDI countries. This notable decrease may benefit from considerable economic development in these countries, resulting in enhanced healthcare systems, greater access to early diagnosis and treatment, and improvements in workplace ergonomics [18]. However, age‐standardized prevalence and DALY rates demonstrated increasing trends across most SDI quintiles, with the most rapid increases occurring in low‐middle SDI countries. This finding could be attributed to rapid societal transitions, lifestyle changes such as increased sedentary behaviour and obesity, and inadequate access to early diagnosis and effective treatment in these countries [18, 19]. Notably, high‐middle SDI countries revealed the sole decrease in age‐standardized DALY rates, likely due to the significant decline in incidence rates. These disparities underscore the urgent need for targeted interventions tailored to the varying sociodemographic contexts to effectively address the substantial burden and rapid increases of MSK disorders in specific SDI countries.

In 2021, women experienced higher age‐standardized incidence, prevalence and DALY rates of MSK disorders among adults aged 50 and over globally and across all SDI quintiles. These gender differences reflect inherent physiological variations, including hormonal differences, body structure and genetic predispositions [20]. From 1990 to 2021, men experienced a more rapid decrease in age‐standardized incidence rates globally and across most SDI quintiles, alongside faster increases in prevalence and DALY rates. The decline in incidence rates among men can likely be attributed to improvements in ergonomics and greater engagement in physical activity, which tend to be more pronounced in male populations [21]. Furthermore, the more rapid increases in prevalence and DALY rates among men may be explained by limited awareness and low adherence to treatment [22]. However, in low and low‐middle SDI countries, women experienced faster increases in age‐standardized DALY rates. This phenomenon reflects that women in these countries are more likely to engage in physically demanding activities, such as household chores, while also facing limited access to healthcare due to cultural norms [23, 24].

In 2021, global incidence and DALY rates for MSK disorders increased with age, peaking in the 75–79 age group before declining in older populations. In contrast, global prevalence rates rose with age, reaching their peak in the 80–84 age group before stagnating. The declines in incidence and DALY rates may be attributed to the survivor effect, wherein individuals more vulnerable to MSK disorders are affected earlier, leaving relatively healthier individuals among the elderly [20]. Additionally, reduced physical activity in individuals aged 80 and above, along with potential underdiagnosis or underreporting among this group, contributes to the decreases in incidence and DALY rates [23, 25]. However, the stagnation in prevalence rates beyond the 80–84 age group suggests that although fewer new cases are being diagnosed, the chronic nature of MSK disorders sustains the existing prevalence. Over the past three decades, age‐specific incidence rates showed decreasing trends across most age groups, with the only increase observed in the 95+ age group, possibly reflecting increased longevity and heightened vulnerability to MSK disorders within this population. In contrast, most age groups revealed increasing trends in age‐specific prevalence and DALY rates, with notable peaks found in the 60–64 and 95+ age groups. These rapid increases are likely due to hormonal changes following menopause and lifestyle shifts after retirement among individuals aged 60–64, along with elevated longevity leading to greater vulnerability among those aged 95 and older [6, 25].

This study further revealed the global burden attributed to the main risk factors for MSK disorders among adults aged 50 and over. In 2021, high BMI was the leading contributor to global DALYs from MSK disorders in this population, underscoring the heightened vulnerability of older individuals to the adverse effects of excess weight [26]. This contrasts with younger adults, whose MSK disorders are predominantly attributed to occupational ergonomic factors [9]. Additionally, occupational ergonomic factors were the predominant contributor to the DALYs of MSK disorders in low‐SDI countries, whereas high BMI, smoking and kidney dysfunction predominated in high‐SDI countries. Moreover, SDI was positively associated with the proportions of DALYs due to occupational ergonomic factors but negatively associated with those due to high BMI, smoking and kidney dysfunction. These findings echo that individuals in low‐SDI countries often engage in more physically demanding labour‐intensive jobs, such as farming, construction and manual labour, frequently under poor workplace ergonomics [27, 28]. Meanwhile, sedentary lifestyles, processed diets and longer life expectancies in high‐SDI countries contribute to elevated risks of obesity and kidney dysfunction, alongside higher smoking prevalence due to easier access to tobacco products [16, 29]. Additionally, men bore higher proportions of DALYs due to occupational ergonomic factors, smoking and kidney dysfunction, whereas women suffered higher proportions of DALYs from high BMI, reflecting inherent gender disparities in exposure to these risk factors [9, 30].

Over the past 30 years, consistent increases have been identified in the global DALYs due to high BMI and kidney dysfunction in the general population [26, 31]. Our study refines this evidence, indicating that the proportions of DALYs due to high BMI and kidney dysfunction have also increased globally for MSK disorders among adults aged 50 and over, highlighting the urgent need to halt these rising trends. Notably, significant decreases were observed in the proportions of DALYs due to occupational ergonomic factors and smoking. These encouraging trends likely result from improvements in workplace ergonomics, reduced physical exertion due to technological advancements and declining smoking prevalence [28, 29]. Interestingly, relatively stable trends were noted in high‐SDI countries for occupational ergonomic factors and in low and low‐middle SDI countries for kidney dysfunction. These findings reflect that although ergonomic improvements in high‐SDI countries have mitigated the impact of labour‐intensive work, limited awareness of kidney dysfunction and inadequate healthcare access in low and low‐middle SDI countries have hindered progress in managing this risk factor [29, 32].

This study further revealed that in 2021, MSK disorders were associated with significantly positive SII values for incidence, prevalence and DALY rates, suggesting that countries with higher SDI levels tend to have higher incidence, prevalence and DALY rates. Additionally, the significantly positive CI values for the numbers of incident cases, prevalent cases and DALYs indicate that higher SDI countries face larger these numbers. Over the period from 1990 to 2021, both absolute and relative SDI‐related inequalities have persisted in MSK disorders, reflecting ongoing disparities despite advancements in global health resources and technology. These findings underscore the importance of developing tailored healthcare strategies to reduce SDI‐related inequalities and more effectively address the global burden of MSK disorders across different socioeconomic contexts.

This study has several notable strengths. First, it is the first to provide a comprehensive assessment of the global burden, temporal trends and risk factors for MSK disorders specifically among adults aged 50 and over. It captures variations across age groups, gender, geographical regions and sociodemographic levels. Furthermore, it offers an in‐depth analysis of sociodemographic inequalities in incidence, prevalence and DALY rates, as well as the corresponding number of MSK disorders over the past 30 years. By utilizing data from the latest GBD 2021 study, this study provides up‐to‐date insights that could inform prevention and management strategies for this vulnerable population. However, several limitations should be acknowledged. First, national estimates may introduce compositional bias due to differences in subnational data sources, methodologies and healthcare access, potentially affecting diagnostic accuracy. Second, although hormonal changes during menopause may contribute to MSK disorders in women, and hormone replacement therapy (HRT) has shown potential benefits in alleviating menopause‐related MSK symptoms [33], the current analysis could not account for HRT use due to data limitations in the GBD 2021 study. Future research linking epidemiological estimates with clinical or pharmacological records is warranted to elucidate this potential association. Additionally, although the study examined major risk factors for MSK disorders, these factors collectively account for less than one third of the global DALYs. Future study should aim to explore the global burden attributable to other potential risk factors to better address the widespread prevalence of MSK disorders in this population.

## 5 Conclusions

Over the past three decades, MSK disorders have remained the largest contributor to disability among adults aged 50 and over. Although significant progress has been achieved in reducing the global age‐standardized incidence rate, both the global prevalence and DALY rates have generally increased. High SDI countries exhibit the highest incidence, prevalence and DALY rates, whereas low‐middle SDI countries show the most rapid increases in prevalence and DALY rates. Furthermore, women have higher age‐standardized incidence, prevalence and DALY rates globally, whereas men experience more rapid increases in prevalence and DALY rates. High BMI serves as the largest contributor to global DALYs for MSK disorders in this population, along with kidney dysfunction, both demonstrating aggravating trends. As the ageing population continues to expand, there is an urgent need for improved prevention strategies tailored to specific contexts of age, gender and sociodemographic factors to mitigate the long‐term impacts of MSK disorders in this vulnerable population.

## Ethics Statement

The authors have nothing to report.

## Conflicts of Interest

The authors declare no conflicts of interest.

## Supporting information


**Figure S1.** Leading causes for the YLDs of adults aged 50 and over and their proportions to overall YLDs in this population between 1990 and 2021.
**Figure S2.** Leading causes for the DALYs of adults aged 50 and over and their proportions to overall DALYs in this population between 1990 and 2021.
**Figure S3.** Numbers of incident cases for MSK disorders among adults aged 50 and over across 204 countries and territories, 2021.
**Figure S4.** Numbers of prevalent cases for MSK disorders among adults aged 50 and over across 204 countries and territories, 2021.
**Figure S5.** DALYs for MSK disorders among adults aged 50 and over across 204 countries and territories, 2021.
**Figure S6.** Global age‐standardized incidence, prevalence and DALY rates for MSK disorders among adults aged 50 and over by SDI and geographic regions, 2021.
**Figure S7.** Age‐standardized incidence rates for MSK disorders among adults aged 50 and over across 204 countries and territories, 2021.
**Figure S8.** Age‐standardized prevalence rates for MSK disorders among adults aged 50 and over across 204 countries and territories, 2021.
**Figure S9.** Gender difference in global age‐standardized incidence, prevalence and DALY rates for MSK disorders among adults aged 50 and over by SDI and geographic regions, 2021.

**Table S1.** Case definitions, data resources, inclusion and exclusion criteria for musculoskeletal disorders 1.
**Table S2.** The definitions, data resources, inclusion and exclusion criteria for risk factors of common musculoskeletal disorders.
**Table S3.** Global numbers of incident cases, prevalent cases and DALYs and their percentage changes for MSK disorders among adults aged 50 and over by SDI and geographic regions, 1990‐2021.
**Table S4.** Numbers of incident cases, prevalent cases and DALYs and their percentage changes for MSK disorders among adults aged 50 and over across 204 countries and territories, 1990‐2021.
**Table S5.** Global age standardized incidence, prevalence and DALY rates (per 100000 population) and their average annual percentage changes for MSK disorders among adults aged 50 and over by SDI and geographic regions, 1990‐2021.
**Table S6.** Age standardized incidence, prevalence and DALY rates (per 100000 population) and their average annual percentage changes for MSK disorders among adults aged 50 and over across 204 countries and territories, 1990‐2021.
**Table S7.** Gender difference in global age standardized incidence, prevalence and DALY rates (per 100000 population) and their average annual percent changes for MSK disorders among adults aged 50 and over by SDI, 1990‐2021.
**Table S8.** Global age‐specific incidence, prevalence and DALY rates (per 100000 population) and their average annual percent changes for MSK disorders among adults aged 50 and over, 1990‐2021.
**Table S9.** Global DALYs attributable to main risk factors and their proportions to overall DALYs for MSK disorders among adults aged 50 and over by SDI and geographic regions, 1990‐2021.
**Table S10.** DALYs attributable to main risk factors and their proportions to overall DALYs for MSK disorders among adults aged 50 and over across 204 countries and territories, 1990‐2021 62.
**Table S11.** Gender difference in global DALYs attributable to main risk factors and their proportions to overall DALYs for MSK disorders among adults aged 50 and over by SDI and geographic regions, 1990‐2021.

## Data Availability

The data used in this study can be derived from the GBD 2021 (available at: https://ghdx.healthdata.org/gbd‐2021).

## References

[1] Z. Paskins , C. E. Farmer , F. Manning , et al., “Research Priorities to Reduce the Impact of Musculoskeletal Disorders: A Priority Setting Exercise With the Child Health and Nutrition Research Initiative Method,” Lancet Rheumatology 4 (2022): e635–e645. 36275038 10.1016/S2665-9913(22)00136-9 PMC9584828

[2] Z. Jin , D. Wang , H. Zhang , et al., “Incidence Trend of Five Common Musculoskeletal Disorders From 1990 to 2017 at the Global, Regional and National Level: Results From the Global Burden of Disease Study 2017,” Annals of the Rheumatic Diseases 8 (2020): 1014–1022. 10.1136/annrheumdis-2020-217050 32414807

[3] A. M. Briggs , A. D. Woolf , K. Dreinhofer , et al., “Reducing the Global Burden of Musculoskeletal Conditions,” Bulletin of the World Health Organization 96 (2018): 366–368. 29875522 10.2471/BLT.17.204891 PMC5985424

[4] S. Bevan , “Economic Impact of Musculoskeletal Disorders (MSDs) on Work in Europe,” Best Practice & Research. Clinical Rheumatology 29 (2015): 356–373. 26612235 10.1016/j.berh.2015.08.002

[5] Versus Arthritis . “The State of Musculoskeletal Health 2019,” no. 91 (2019): 31–32.

[6] R. A. Lobo and A. Gompel , “Management of Menopause: A View Towards Prevention,” Lancet Diabetes & Endocrinology 10 (2022): 457–470. 35526556 10.1016/S2213-8587(21)00269-2

[7] M. Gulati , E. Dursun , K. Vincent , and F. E. Watt , “The Influence of Sex Hormones on Musculoskeletal Pain and Osteoarthritis,” Lancet Rheumatology 5 (2023): e225–e238. 38251525 10.1016/S2665-9913(23)00060-7

[8] GBD 2021 Diseases and Injuries Collaborators , “Global Incidence, Prevalence, Years Lived With Disability (YLDs), Disability‐Adjusted Life‐Years (DALYs), and Healthy Life Expectancy (HALE) for 371 Diseases and Injuries in 204 Countries and Territories and 811 Subnational Locations, 1990–2021: A Systematic Analysis for the Global Burden of Disease Study 2021,” Lancet 403 (2024): 2133–2161. 38642570 10.1016/S0140-6736(24)00757-8 PMC11122111

[9] S. Y. Guan , J. X. Zheng , N. B. Sam , S. Xu , Z. Shuai , and F. Pan , “Global Burden and Risk Factors of Musculoskeletal Disorders Among Adolescents and Young Adults in 204 Countries and Territories, 1990–2019,” Autoimmunity Reviews 22 (2023): 103361. 37230312 10.1016/j.autrev.2023.103361

[10] G. B. D. D. Collaborators , “Global Age‐Sex‐Specific Mortality, Life Expectancy, and Population Estimates in 204 Countries and Territories and 811 Subnational Locations, 1950‐2021, and the Impact of the COVID‐19 Pandemic: A Comprehensive Demographic Analysis for the Global Burden of Disease Study 2021,” Lancet 403 (2024): 1989–2056. 38484753 10.1016/S0140-6736(24)00476-8 PMC11126395

[11] GBD 2021 Demographics Collaborators , “Global Burden and Strength of Evidence for 88 Risk Factors in 204 Countries and 811 Subnational Locations, 1990–2021: A Systematic Analysis for the Global Burden of Disease Study 2021,” Lancet 403 (2024): 2162–2203. 38762324 10.1016/S0140-6736(24)00933-4 PMC11120204

[12] H. J. Kim , M. P. Fay , E. J. Feuer , and D. N. Midthune , “Permutation Tests for Joinpoint Regression With Applications to Cancer Rates,” Statistics in Medicine 19 (2000): 335–351. 10649300 10.1002/(sici)1097-0258(20000215)19:3<335::aid-sim336>3.0.co;2-z

[13] X. Lai , H. Zhang , K. B. Pouwels , B. Patenaude , M. Jit , and H. Fang , “Estimating Global and Regional Between‐Country Inequality in Routine Childhood Vaccine Coverage in 195 Countries and Territories From 2019 to 2021: A Longitudinal Study,” eClinicalMedicine 60 (2023): 102042. 37304497 10.1016/j.eclinm.2023.102042 PMC10249397

[14] World Health Organization , Handbook on Health Inequality Monitoring: With a Special Focus on Low‐ and Middle‐Income Countries (World Health Organization, 2013).

[15] GBD 2019 Demographics Collaborators , “Global Age‐Sex‐Specific Fertility, Mortality, Healthy Life Expectancy (HALE), and Population Estimates in 204 Countries and Territories, 1950–2019: A Comprehensive Demographic Analysis for the Global Burden of Disease Study 2019,” Lancet 396 (2020): 1160–1203. 33069325 10.1016/S0140-6736(20)30977-6 PMC7566045

[16] X. Cheng , Y. Yang , D. C. Schwebel , et al., “Population Ageing and Mortality During 1990–2017: A Global Decomposition Analysis,” PLoS Medicine 17 (2020): e1003138. 32511229 10.1371/journal.pmed.1003138 PMC7279585

[17] S. Safiri , A. A. Kolahi , M. Cross , et al., “Prevalence, Deaths, and Disability‐Adjusted Life Years Due to Musculoskeletal Disorders for 195 Countries and Territories 1990‐2017,” Arthritis & Rheumatology 73 (2021): 702–714. 33150702 10.1002/art.41571

[18] GBD 2019 Universal Health Coverage Collaborators , “Measuring Universal Health Coverage Based on an Index of Effective Coverage of Health Services in 204 Countries and Territories, 1990–2019: A Systematic Analysis for the Global Burden of Disease Study 2019,” Lancet 396 (2020): 1250–1284. 32861314 10.1016/S0140-6736(20)30750-9 PMC7562819

[19] T. J. Bollyky , T. Templin , M. Cohen , and D. JLJHa , “Lower‐Income Countries That Face the Most Rapid Shift in Noncommunicable Disease Burden Are Also the Least Prepared,” 36 (2017): 1866–1875. 10.1377/hlthaff.2017.0708 PMC7705176 29137514

[20] S. Safiri , A. A. Kolahi , M. Cross , et al., “Prevalence, Deaths, and Disability‐Adjusted Life Years Due to Musculoskeletal Disorders for 195 Countries and Territories 1990–2017,” Arthritis & Rhematology 73 (2021): 702–714. 10.1002/art.41571 33150702

[21] C. Bontrup , W. R. Taylor , M. Fliesser , et al., “Low Back Pain and Its Relationship With Sitting Behaviour Among Sedentary Office Workers,” Applied Ergonomics 81 (2019): 102894. 31422243 10.1016/j.apergo.2019.102894

[22] B. Maranini , A. Bortoluzzi , E. Silvagni , and M. Govoni , “Focus on Sex and Gender: What We Need to Know in the Management of Rheumatoid Arthritis,” Journal of Personalized Medicine 12 (2022): 499. 35330498 10.3390/jpm12030499 PMC8948892

[23] C. Suso‐Ribera , E. Yakobov , J. S. Carriere , and A. Garcia‐Palacios , “The Impact of Chronic Pain on Patients and Spouses: Consequences on Occupational Status, Distribution of Household Chores and Care‐Giving Burden,” European Journal of Pain 24 (2020): 1730–1740. 32533892 10.1002/ejp.1616

[24] H. Shibli , L. Aharonson‐Daniel , and P. Feder‐Bubis , “Perceptions About the Accessibility of Healthcare Services Among Ethnic Minority Women: A Qualitative Study Among Arab Bedouins in Israel,” International Journal for Equity in Health 20 (2021): 117. 33964946 10.1186/s12939-021-01464-9 PMC8106134

[25] C. Genebra , N. M. Maciel , T. P. F. Bento , S. Simeao , and A. Vitta , “Prevalence and Factors Associated With Neck Pain: A Population‐Based Study,” Brazilian Journal of Physical Therapy 21 (2017): 274–280. 28602744 10.1016/j.bjpt.2017.05.005 PMC5537482

[26] H. Dai , T. A. Alsalhe , N. Chalghaf , M. Ricco , N. L. Bragazzi , and J. Wu , “The Global Burden of Disease Attributable to High Body Mass Index in 195 Countries and Territories, 1990–2017: An Analysis of the Global Burden of Disease Study,” PLoS Medicine 17 (2020): e1003198. 32722671 10.1371/journal.pmed.1003198 PMC7386577

[27] D. G. Hoy , E. Smith , M. Cross , et al., “Reflecting on the Global Burden of Musculoskeletal Conditions: Lessons Learnt From the Global Burden of Disease 2010 Study and the Next Steps Forward,” Annals of the Rheumatic Diseases 74 (2015): 4–7. 24914071 10.1136/annrheumdis-2014-205393

[28] M. B. Reitsma , N. Fullman , M. Ng , et al., “Smoking Prevalence and Attributable Disease Burden in 195 Countries and Territories, 1990–2015: A Systematic Analysis From the Global Burden of Disease Study 2015,” Lancet 389 (2017): 1885–1906. 28390697 10.1016/S0140-6736(17)30819-X PMC5439023

[29] Collaborators GBDORF , “Global and Regional Burden of Disease and Injury in 2016 Arising From Occupational Exposures: A Systematic Analysis for the Global Burden of Disease Study 2016,” Occupational and Environmental Medicine 77 (2020): 133–141. 32054817 10.1136/oemed-2019-106008 PMC7035694

[30] M. Cross , K. L. Ong , G. T. Culbreth , et al., “Global, Regional, and National Burden of Gout, 1990–2020, and Projections to 2050: A Systematic Analysis of the Global Burden of Disease Study 2021,” Lancet Rheumatology. 6 (2024): e507–e517. 38996590 10.1016/S2665-9913(24)00117-6 PMC11263476

[31] S. Zhang , H. F. Ren , R. X. Du , W. L. Sun , M. L. Fu , and X. C. Zhang , “Global, Reg5ional, and National Burden of Kidney Dysfunction From 1990 to 2019: A Systematic Analysis From the Global Burden of Disease Study 2019,” BMC Public Health 23 (2023): 1218. 37353821 10.1186/s12889-023-16130-8 PMC10288715

[32] A. J. Lewington , J. Cerda , and R. L. Mehta , “Raising Awareness of Acute Kidney Injury: A Global Perspective of a Silent Killer,” Kidney International 84 (2013): 457–467. 23636171 10.1038/ki.2013.153 PMC3758780

[33] H. Pang , S. Chen , D. M. Klyne , et al., “Low Back Pain and Osteoarthritis Pain: A Perspective of Estrogen,” Bone Research 11 (2023): 42. 37542028 10.1038/s41413-023-00280-x PMC10403578

