# Supplementary material for: Global Burden of Musculoskeletal Disorders in Adults Aged 50 and Over, 1990–2021: Risk Factors and Sociodemographic Inequalities
Source: J Cachexia Sarcopenia Muscle. 2025 Jul 10;16(4):e70008. doi: 10.1002/jcsm.70008 (PMC12242706; doi:10.1002/jcsm.70008)
Supplement: Supplementary file 1 — Figure S1. Leading causes for the YLDs of adults aged 50 and over and their proportions to overall YLDs in this population between 1990 and 2021. Figure S2. Leading causes for the DALYs of adults aged 50 and over and their proportions to overall DALYs in this population between 1990 and 2021. Figure S3. Numbers of incident cases for MSK disorders among adults aged 50 and over across 204 countries and territories, 2021. Figure S4. Numbers of prevalent cases for MSK disorders among adults aged 50 and over across 204 countries and territories, 2021. Figure S5. DALYs for MSK disorders among adults aged 50 and over across 204 countries and territories, 2021. Figure S6. Global age‐standardized incidence, prevalence and DALY rates for MSK disorders among adults aged 50 and over by SDI and geographic regions, 2021. Figure S7. Age‐standardized incidence rates for MSK disorders among adults aged 50 and over across 204 countries and territories, 2021. Figure S8. Age‐standardized prevalence rates for MSK disorders among adults aged 50 and over across 204 countries and territories, 2021. Figure S9. Gender difference in global age‐standardized incidence, prevalence and DALY rates for MSK disorders among adults aged 50 and over by SDI and geographic regions, 2021. [file JCSM-16-e70008-s001.pdf]

Supplementary Figures

Supplementary Figure 1 Leading causes for the YLDs of adults aged 50 and over and their proportions to overall YLDs in this population between 1990 and 2021 1

Supplementary Figure 2 Leading causes for the DALYs of adults aged 50 and over and their proportions to overall DALYs in this population between 1990 and 2021 2

Supplementary Figure 3 Numbers of incident cases for MSK disorders among adults aged 50 and over across 204 countries and territories, 2021 3

Supplementary Figure 4 Numbers of prevalent cases for MSK disorders among adults aged 50 and over across 204 countries and territories, 2021 4

Supplementary Figure 5 DALYs for MSK disorders among adults aged 50 and over across 204 countries and territories, 2021 5

Supplementary Figure 6 Global age-standardized incidence, prevalence and DALY rates for MSK disorders among adults aged 50 and over by SDI and geographic regions, 2021 6

Supplementary Figure 7 Age-standardized incidence rates for MSK disorders among adults aged 50 and over across 204 countries and territories, 2021 7

Supplementary Figure 8 Age-standardized prevalence rates for MSK disorders among adults aged 50 and over across 204 countries and territories, 2021 8

Supplementary Figure 9 Gender difference in global age-standardized incidence, prevalence and DALY rates for MSK disorders among adults aged 50 and over by SDI and geographic regions, 2021 9

Supplementary Figure 10 Global age-specific incidence, prevalence and DALY rates for MSK disorders among adults aged 50 and over 50, 2021 10
Supplementary Figure 11 Percentage changes of incident cases for MSK disorders among adults aged 50 and over across 204 countries and territories, 1990-2021 11
Supplementary Figure 12 Percentage changes of prevalent cases for MSK disorders among adults aged 50 and over across 204 countries and territories, 1990-2021 12
Supplementary Figure 13 Percentage changes of DALYs for MSK disorders among adults aged 50 and over across 204 countries and territories, 1990-2021 13
Supplementary Figure 14 Average annual percent changes in age-standardized incidence, prevalence and DALY rates for MSK disorders among adults aged 50 and over across 21 geographic regions, 1990-2021 14
Supplementary Figure 15 Average annual percent changes in age-standardized incidence rates for MSK disorders among adults aged 50 and over across 204 countries and territories, 1990-2021 15
Supplementary Figure 16 Average annual percent changes in age-standardized prevalence rates for MSK disorders among adults aged 50 and over across 204 countries and territories, 1990-2021 16
Supplementary Figure 17 Average annual percent changes in age-standardized DALY rates for MSK disorders among adults aged 50 and over across 204 countries and territories, 1990-2021 17
Supplementary Figure 18 Gender difference in average annual percent changes in global age-standardized incidence, prevalence and DALY rates for MSK disorders among adults aged 50 and over by SDI, 1990-2021 18
Supplementary Figure 19 Average annual percent changes in global age-specific incidence, prevalence and DALY rates for MSK disorders among

adults aged 50 and over, 1990-2021 19

Supplementary Figure 20 Association between SDI and the proportions of DALYs attributable to high BMI, occupational ergonomic factors, smoking, and kidney dysfunction for MSK disorders among adults aged 50 and over across 204 countries and territories, 2021 20

Supplementary Figure 21 The proportions of DALYs attributable to high BMI, occupational ergonomic factors, smoking, and kidney dysfunction for MSK disorders among adults aged 50 and over across 21 geographic regions, 2021 21

Supplementary Figure 22 The proportions of DALYs attributable to high BMI for MSK disorders among adults aged 50 and over across 204 countries and territories, 2021........ 22

Supplementary Figure 23 The proportions of DALYs attributable to occupational ergonomic factors for MSK disorders among adults aged 50 and over across 204 countries and territories, 2021........ 23

Supplementary Figure 24 The proportions of DALYs attributable to smoking for MSK disorders among adults aged 50 and over across 204 countries and territories, 2021........ 24

Supplementary Figure 25 The proportions of DALYs attributable to kidney dysfunction for MSK disorders among adults aged 50 and over across 204 countries and territories, 2021........ 25

Supplementary Figure 26 Absolute SDI-related inequalities in incidence, prevalence and DALY rates for MSK disorders among adults aged 50 and over across 204 countries and territories, 1990-2021 26

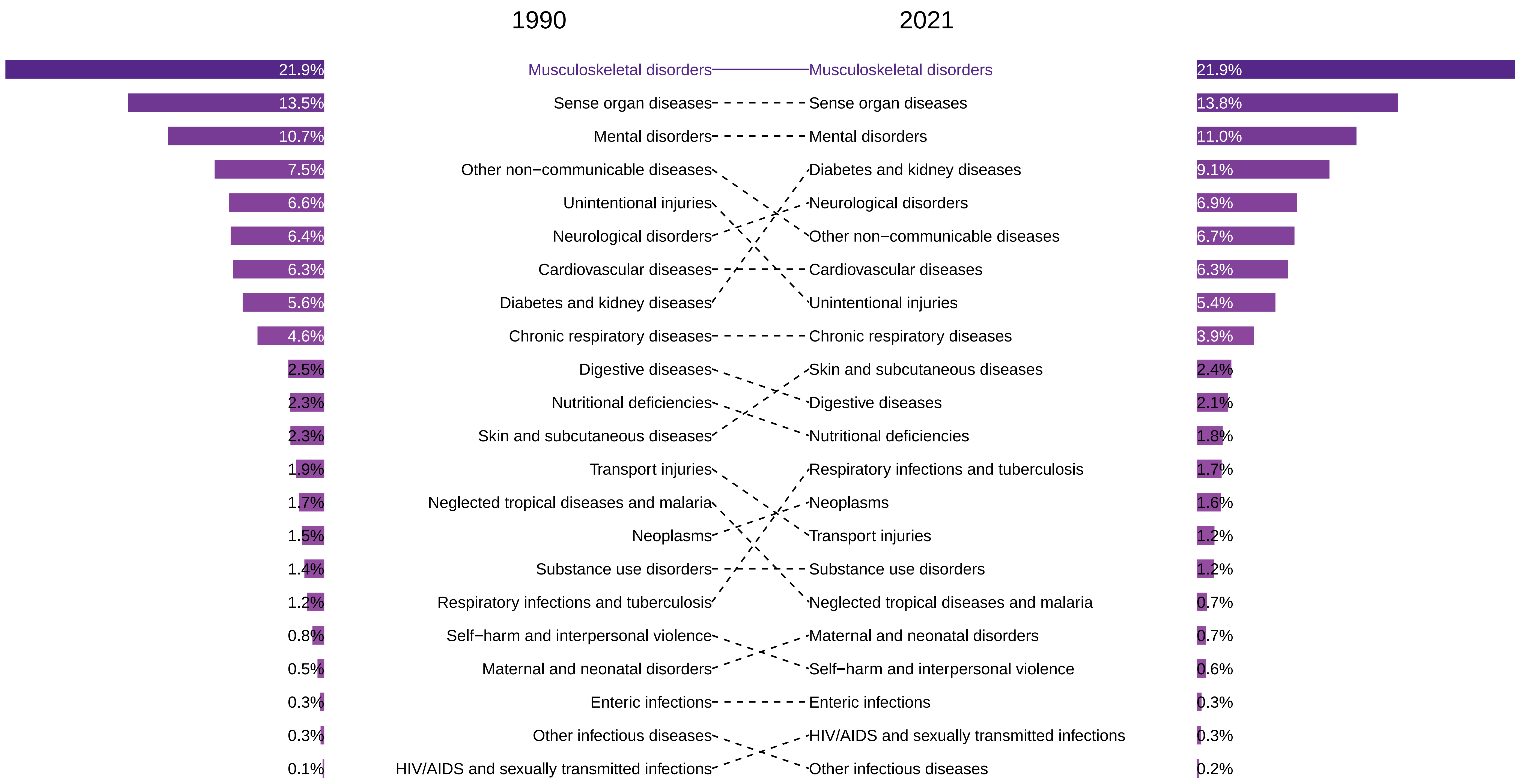

Supplementary Figure 1 Leading causes for the YLDs of adults aged 50 and over and their proportions to overall YLDs in this population between 1990 and 2021.

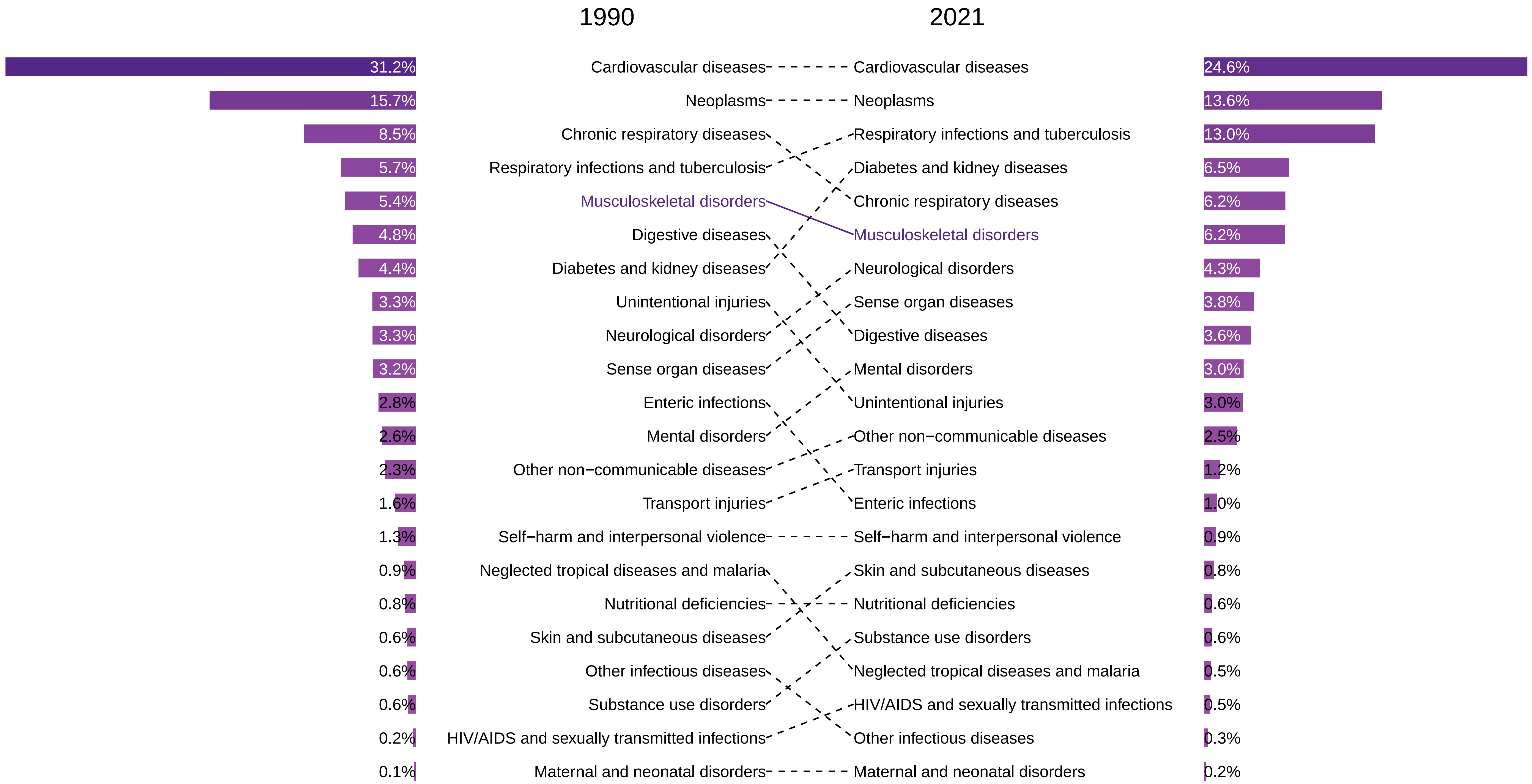

Supplementary Figure 2 Leading causes for the DALYs of adults aged 50 and over and their proportions to overall DALYs in this population between 1990 and 2021.

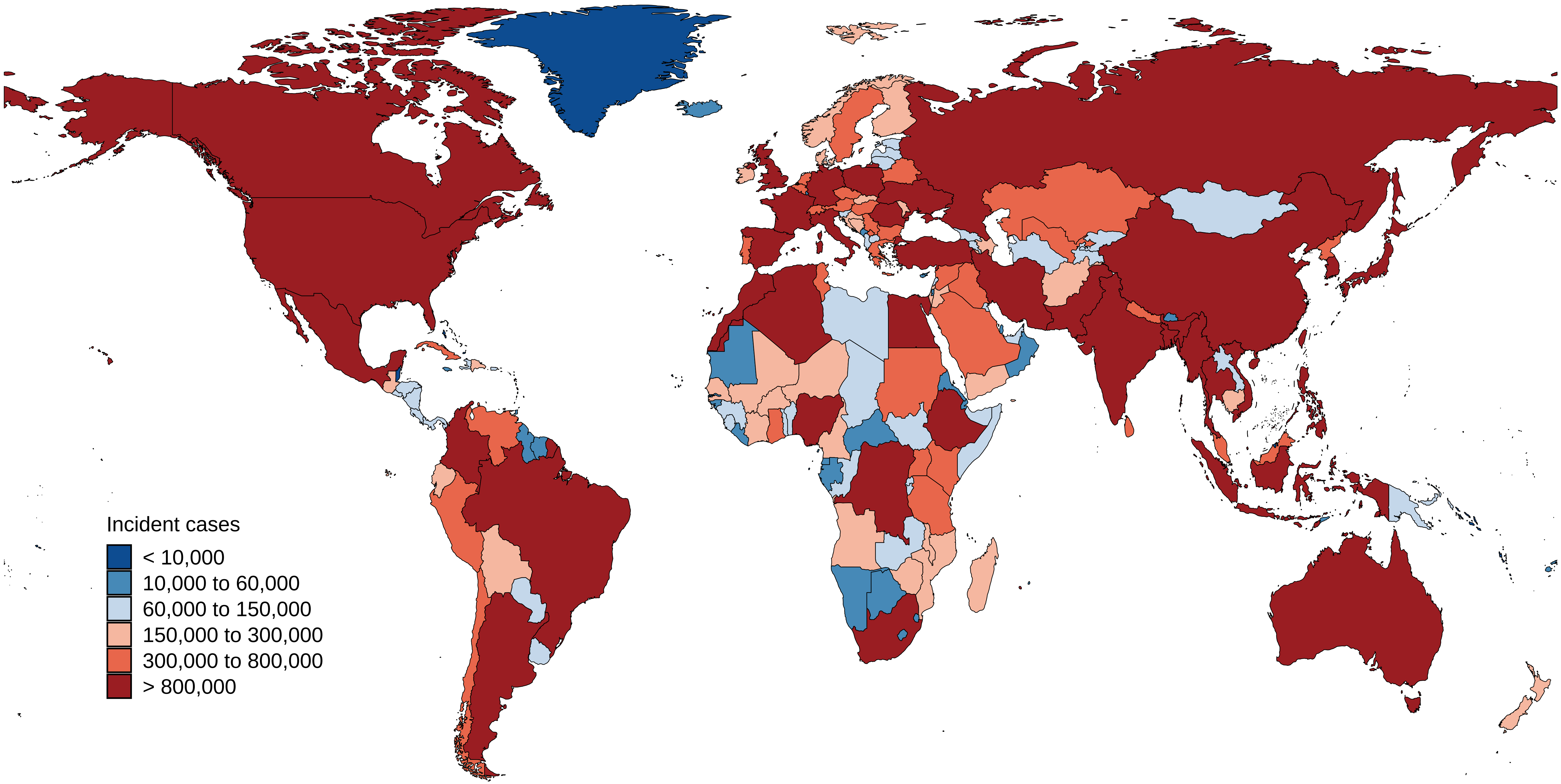

Supplementary Figure 3 Numbers of incident cases for MSK disorders among adults aged 50 and over across 204 countries and territories, 2021.

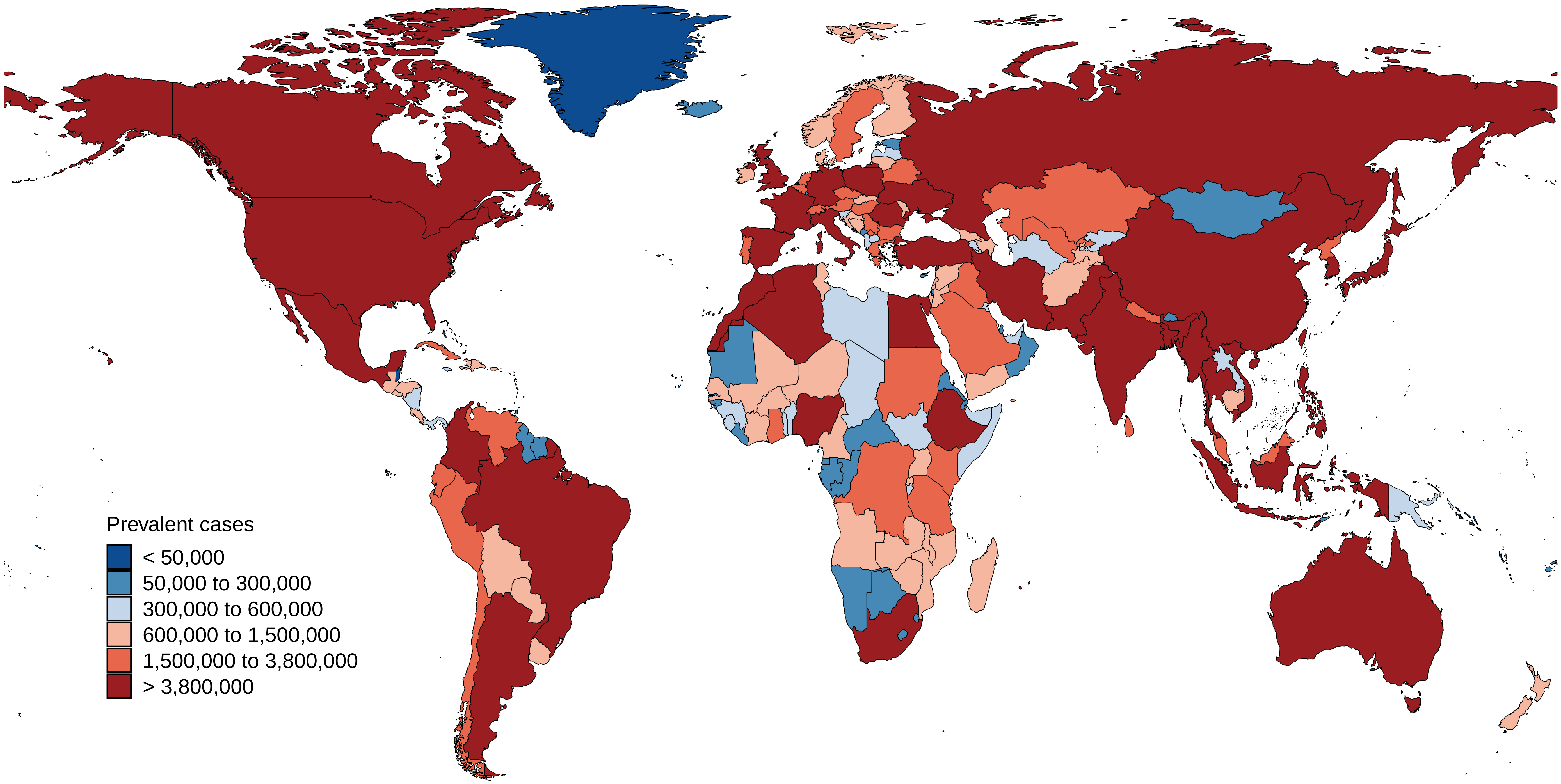

Supplementary Figure 4 Numbers of prevalent cases for MSK disorders among adults aged 50 and over across 204 countries and territories, 2021.

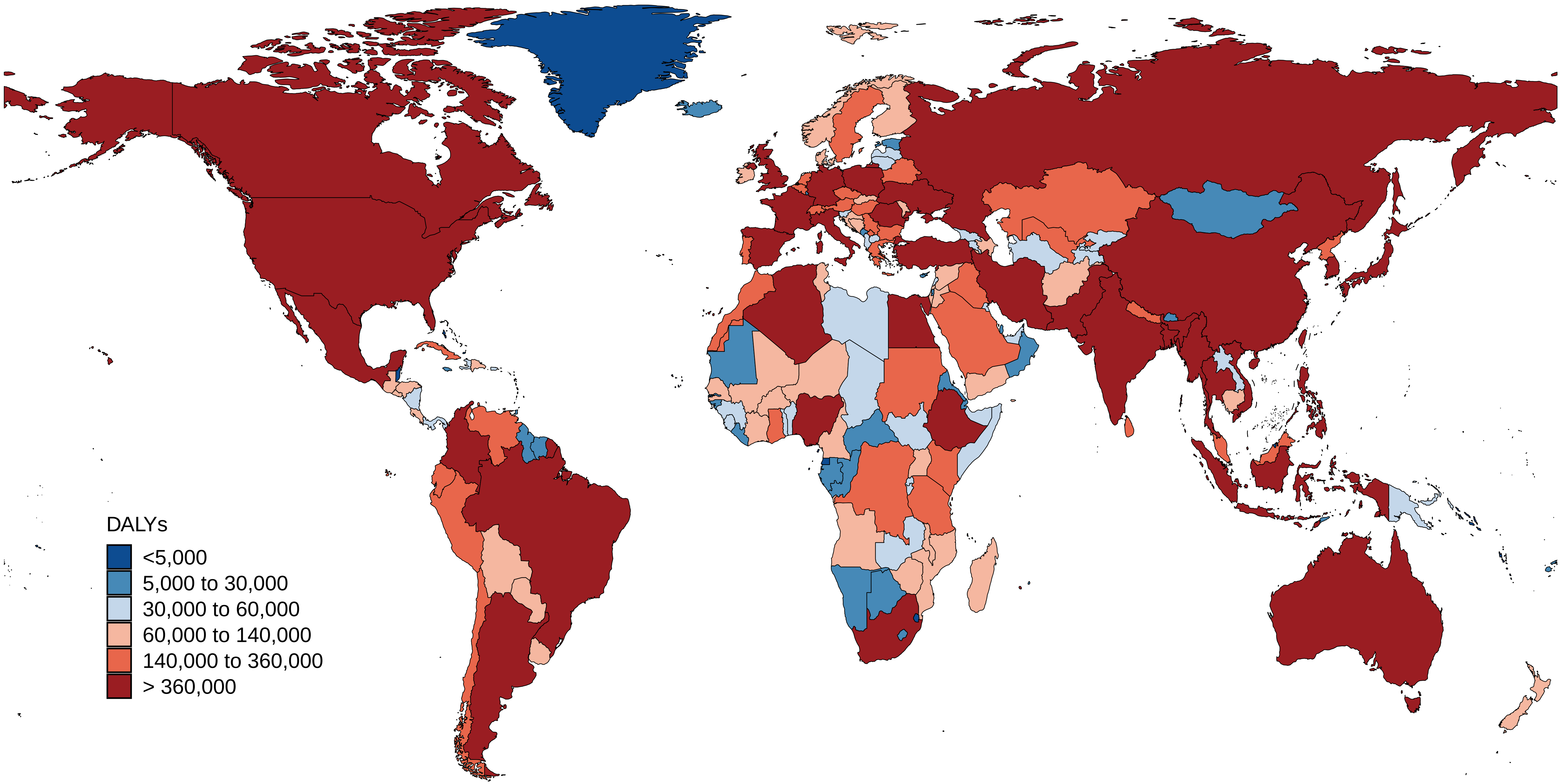

Supplementary Figure 5 DALYs for MSK disorders among adults aged 50 and over across 204 countries and territories, 2021.

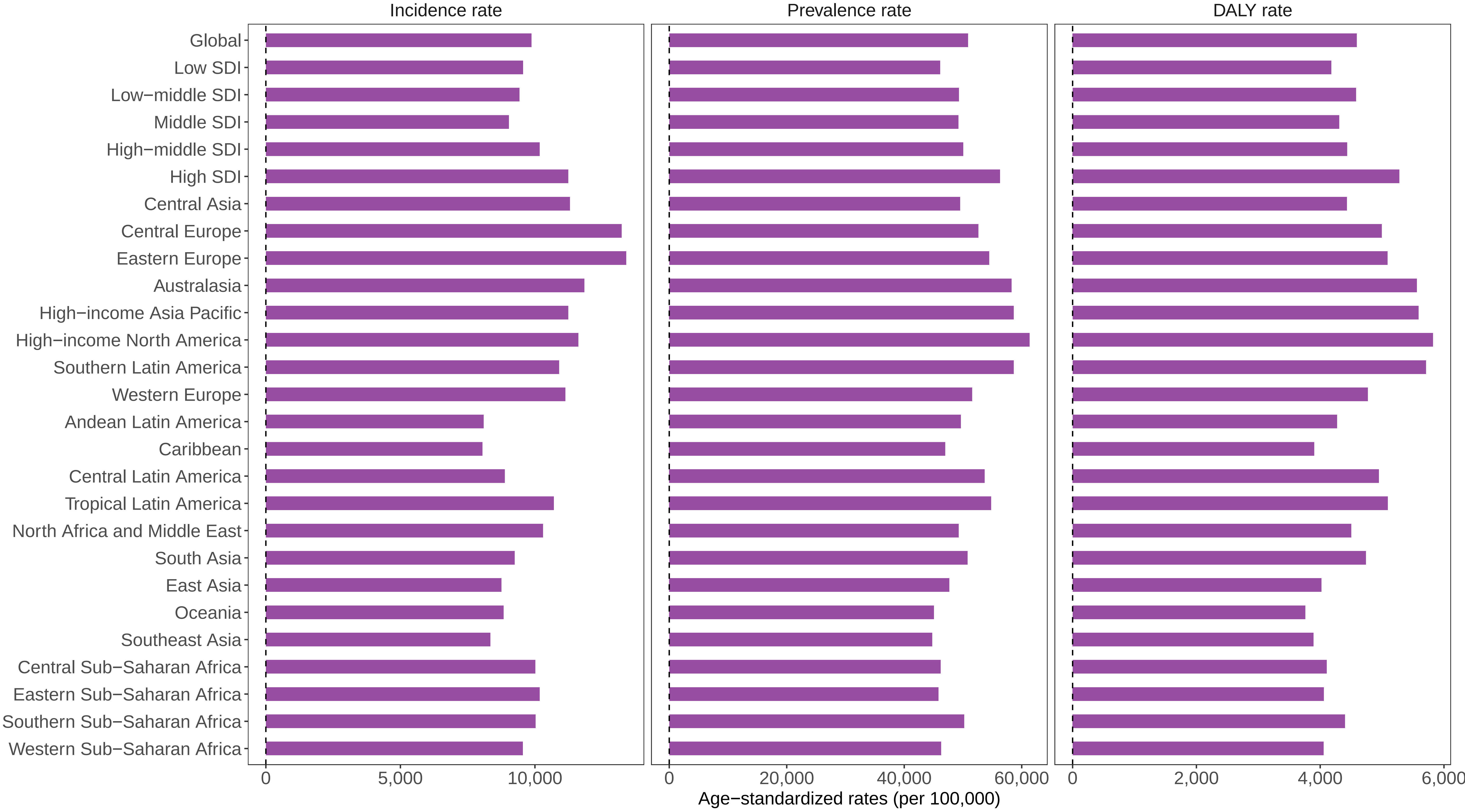

Supplementary Figure 6 Global age-standardized incidence, prevalence and DALY rates for MSK disorders among adults aged 50 and over by SDI and geographic regions, 2021.

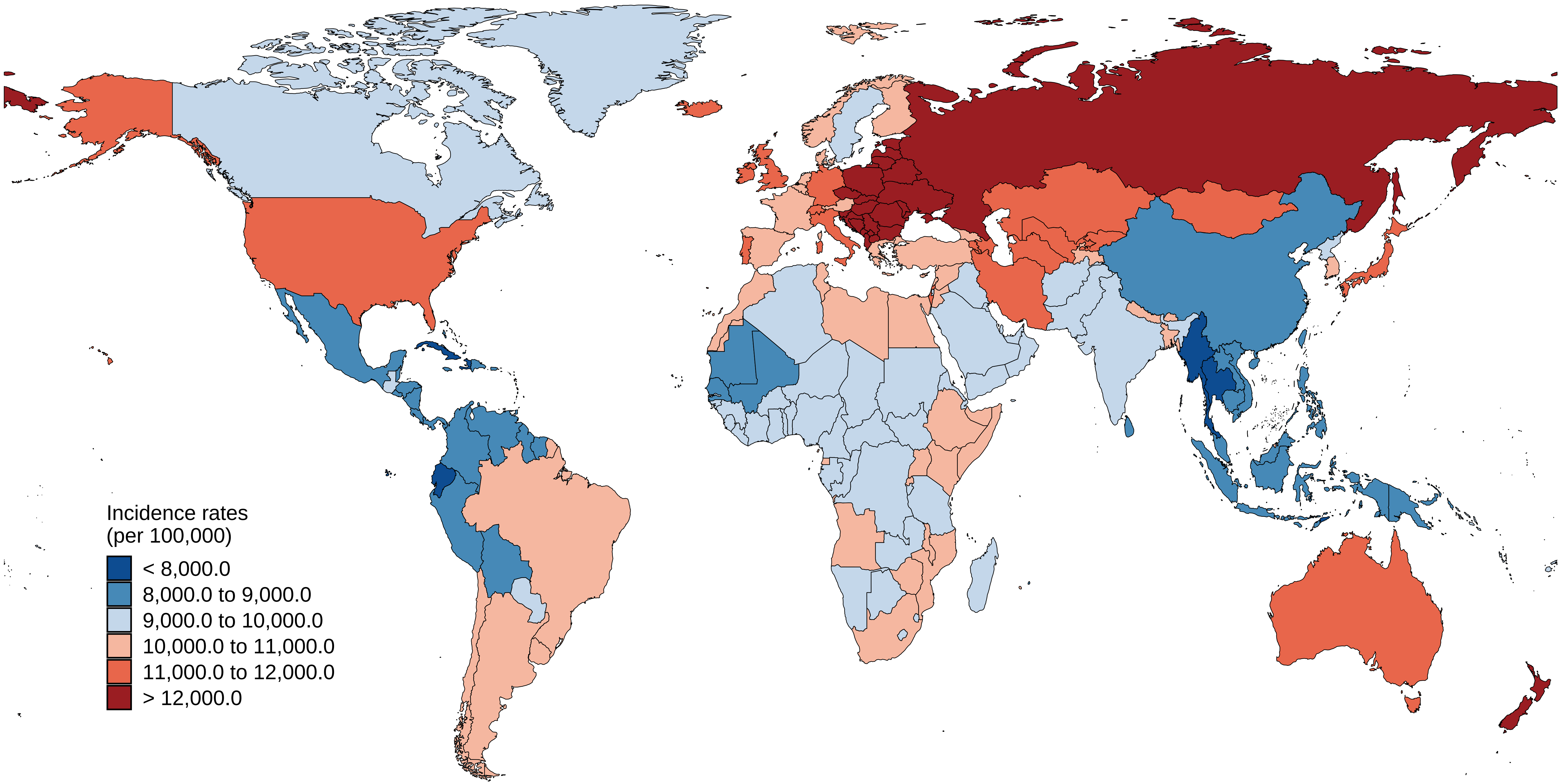

Supplementary Figure 7 Age-standardized incidence rates for MSK disorders among adults aged 50 and over across 204 countries and territories, 2021.

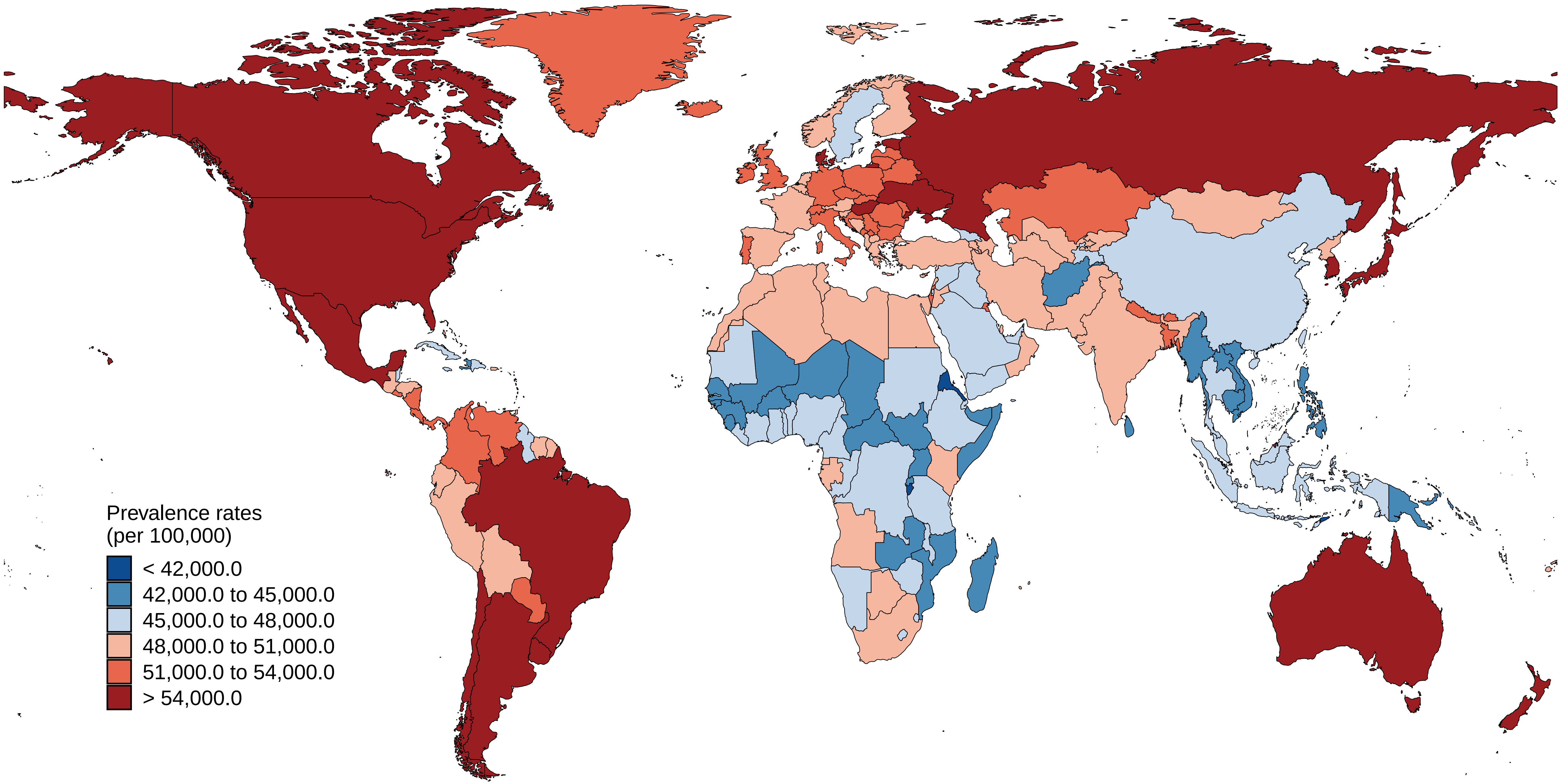

Supplementary Figure 8 Age-standardized prevalence rates for MSK disorders among adults aged 50 and over across 204 countries and territories, 2021.

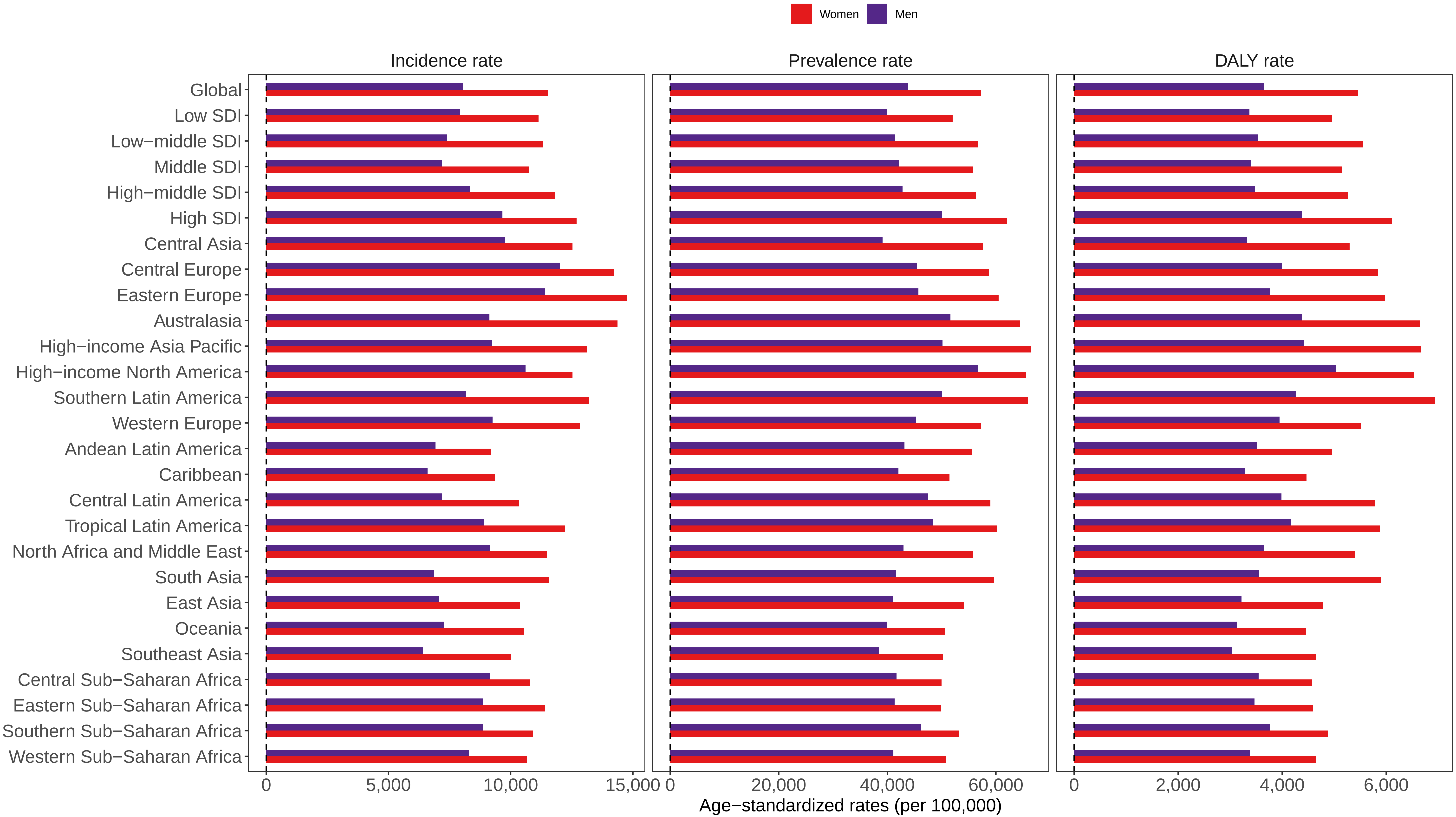

Supplementary Figure 9 Gender difference in global age-standardized incidence, prevalence and DALY rates for MSK disorders among adults aged 50 and over by SDI and geographic regions, 2021.

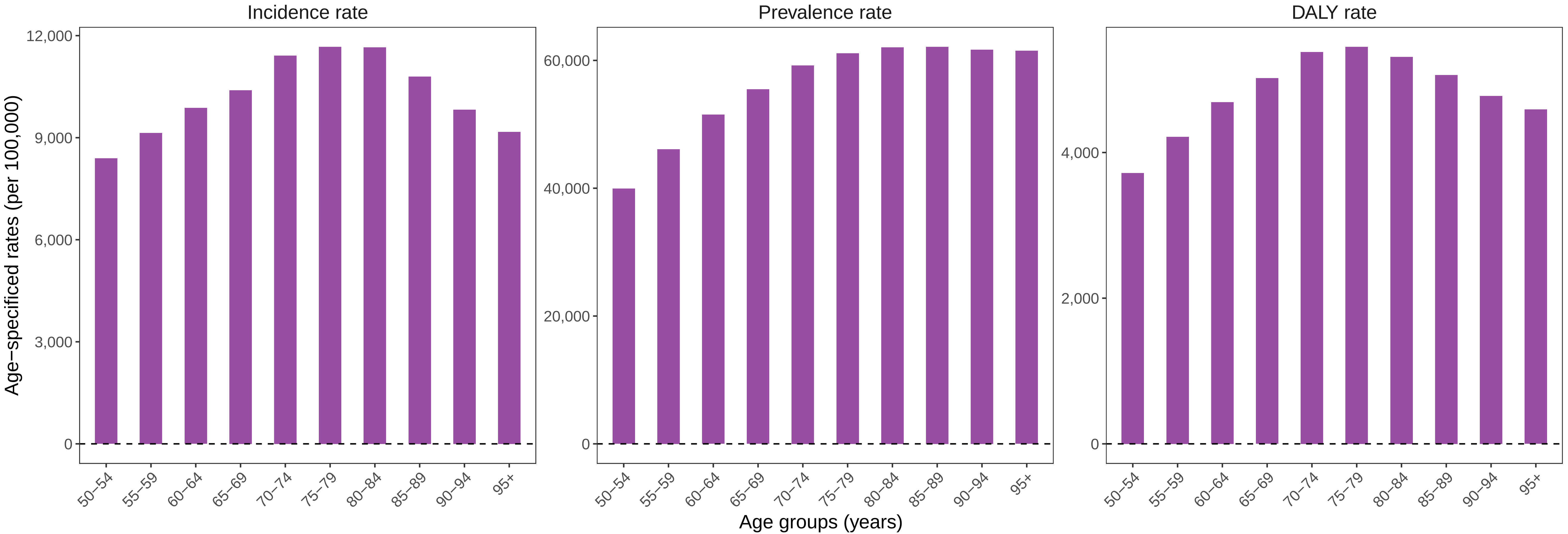

Supplementary Figure 10 Global age-specific incidence, prevalence and DALY rates for MSK disorders among adults aged 50 and over 50, 2021.

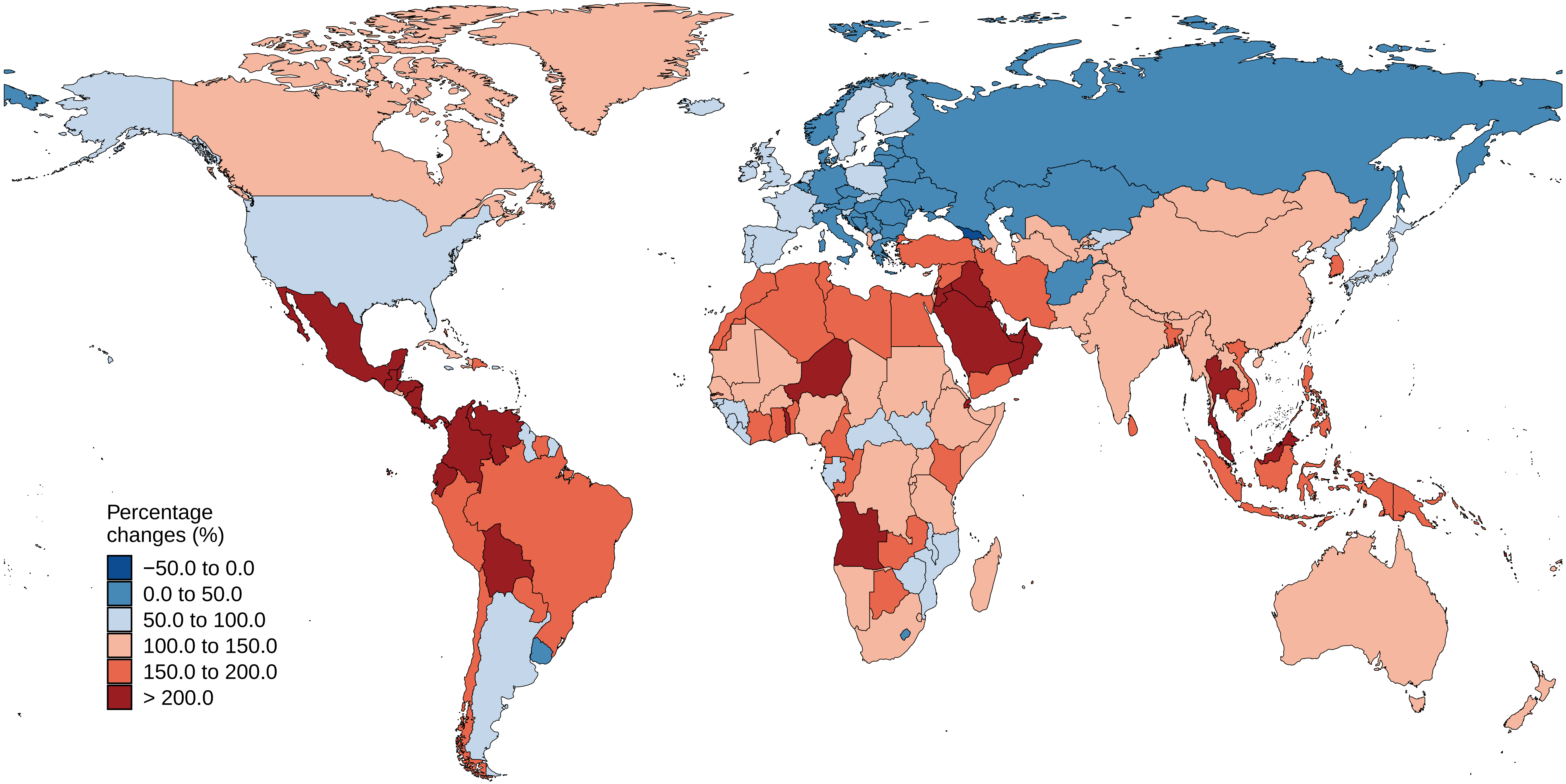

Supplementary Figure 11 Percentage changes of incident cases for MSK disorders among adults aged 50 and over across 204 countries and territories, 1990-2021.

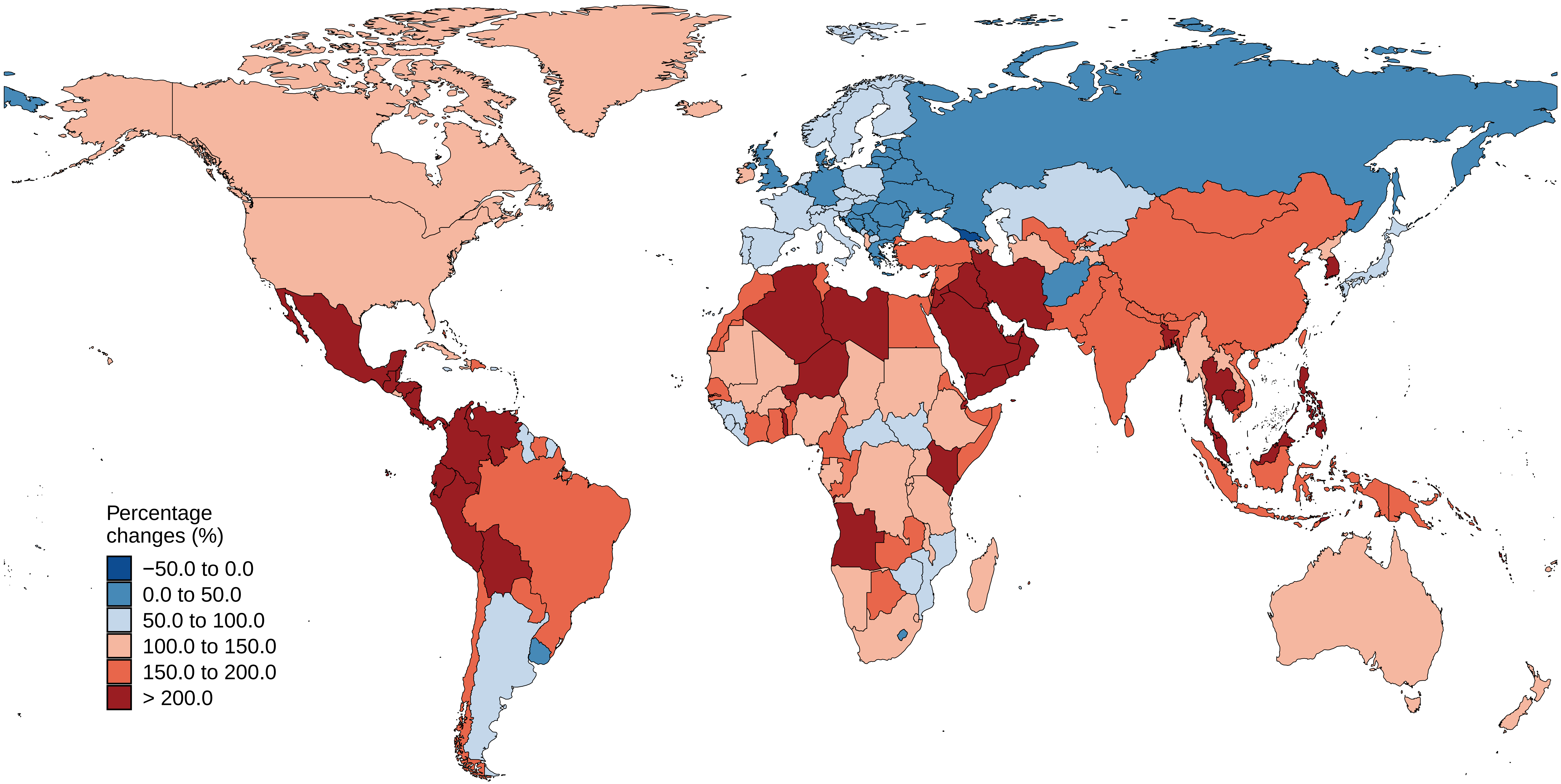

Supplementary Figure 12 Percentage changes of prevalent cases for MSK disorders among adults aged 50 and over across 204 countries and territories, 1990-2021.

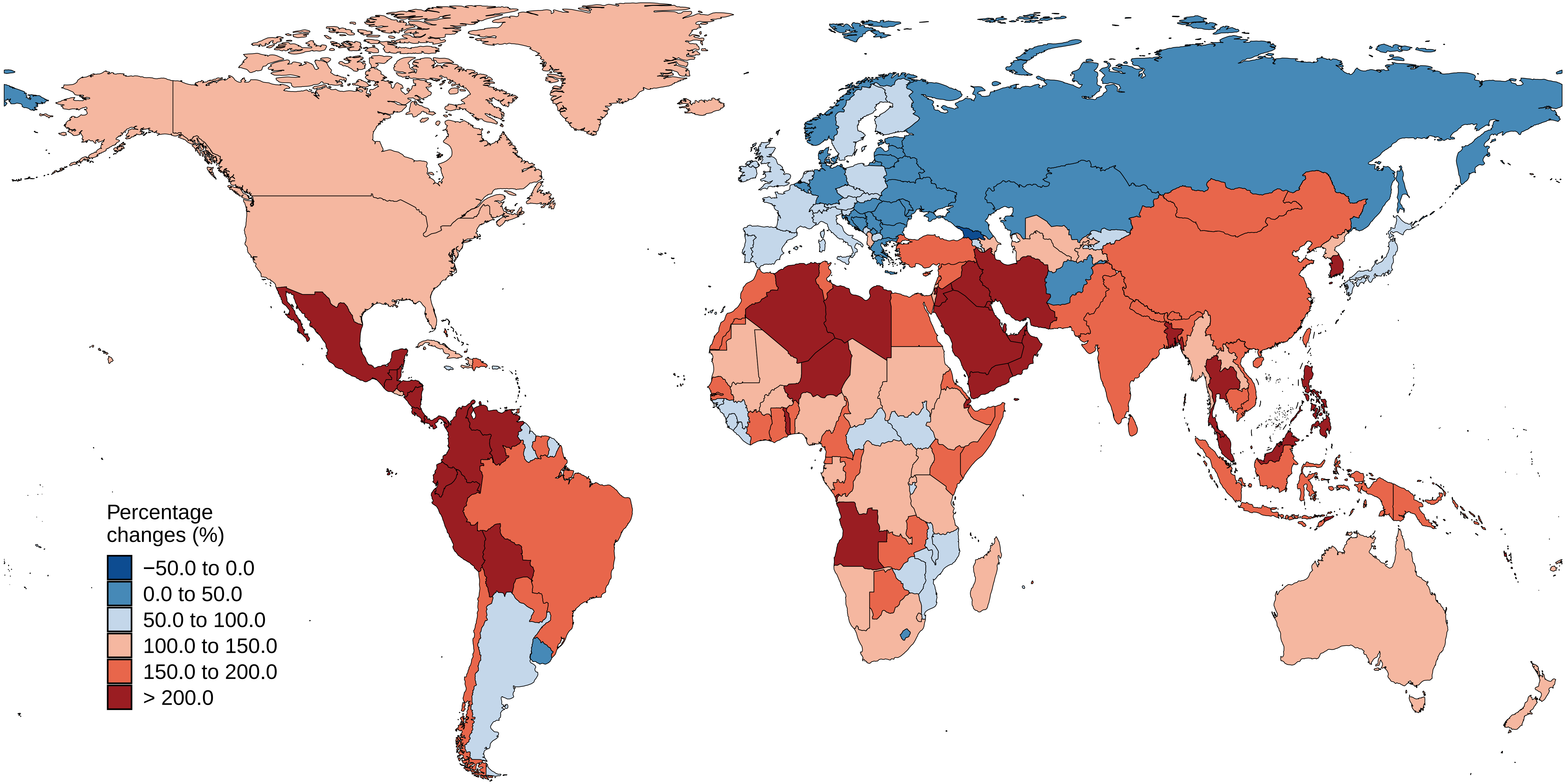

Supplementary Figure 13 Percentage changes of DALYs for MSK disorders among adults aged 50 and over across 204 countries and territories, 1990-2021.

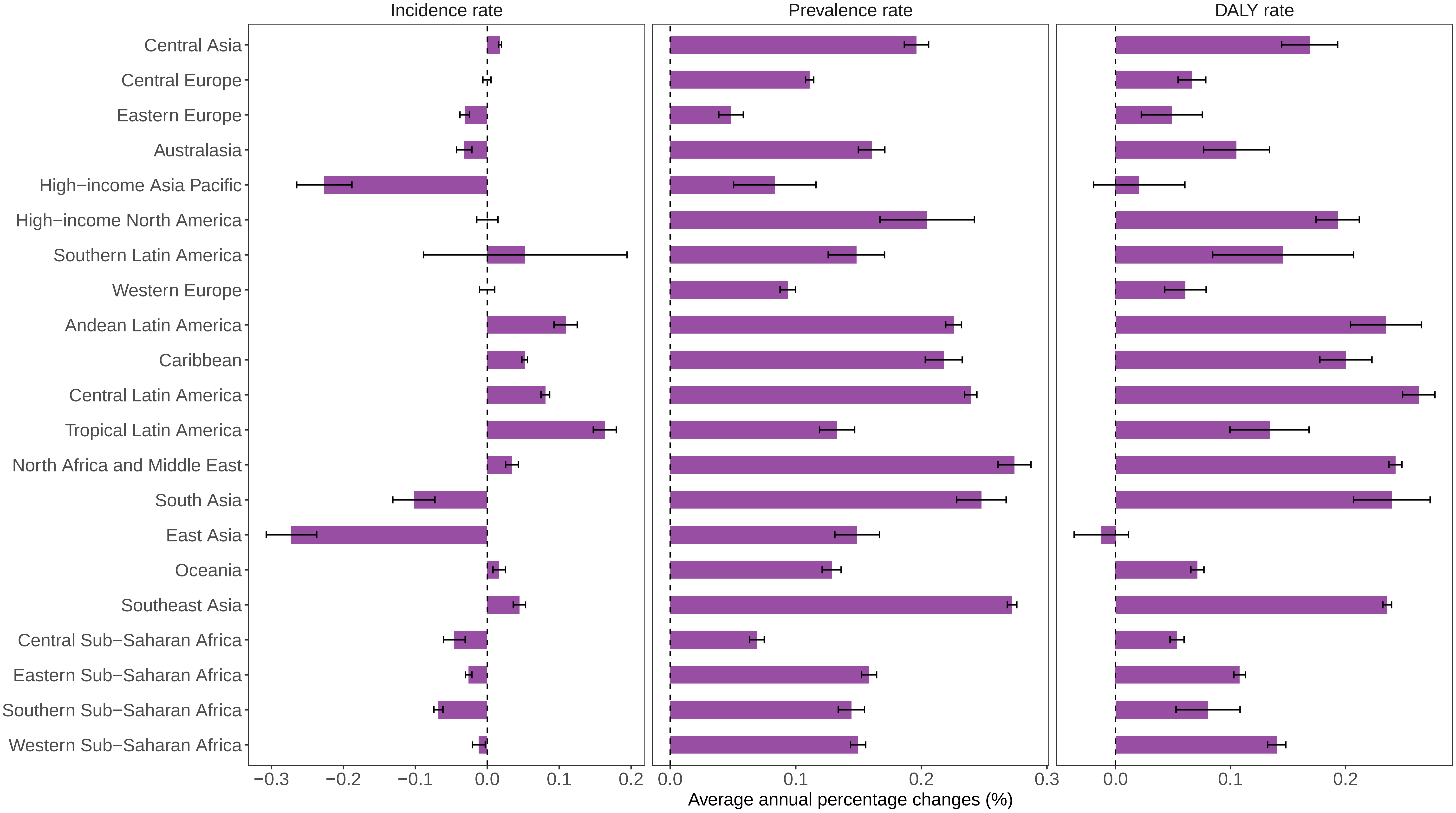

Supplementary Figure 14 Average annual percent changes in age-standardized incidence, prevalence and DALY rates for MSK disorders among adults aged 50 and over across 21 geographic regions, 1990-2021.

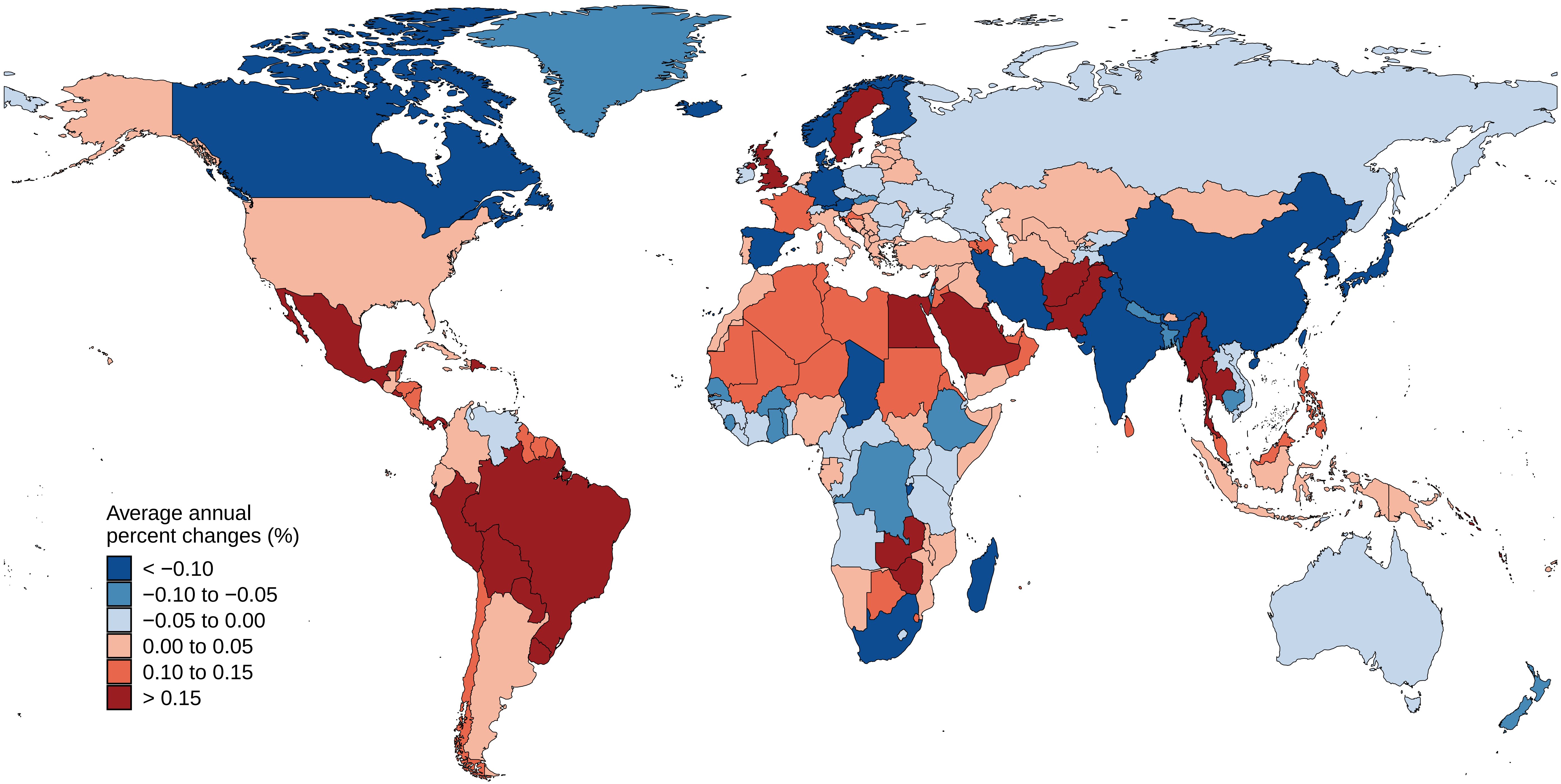

Supplementary Figure 15 Average annual percent changes in age-standardized incidence rates for MSK disorders among adults aged 50 and over across 204 countries and territories, 1990-2021.

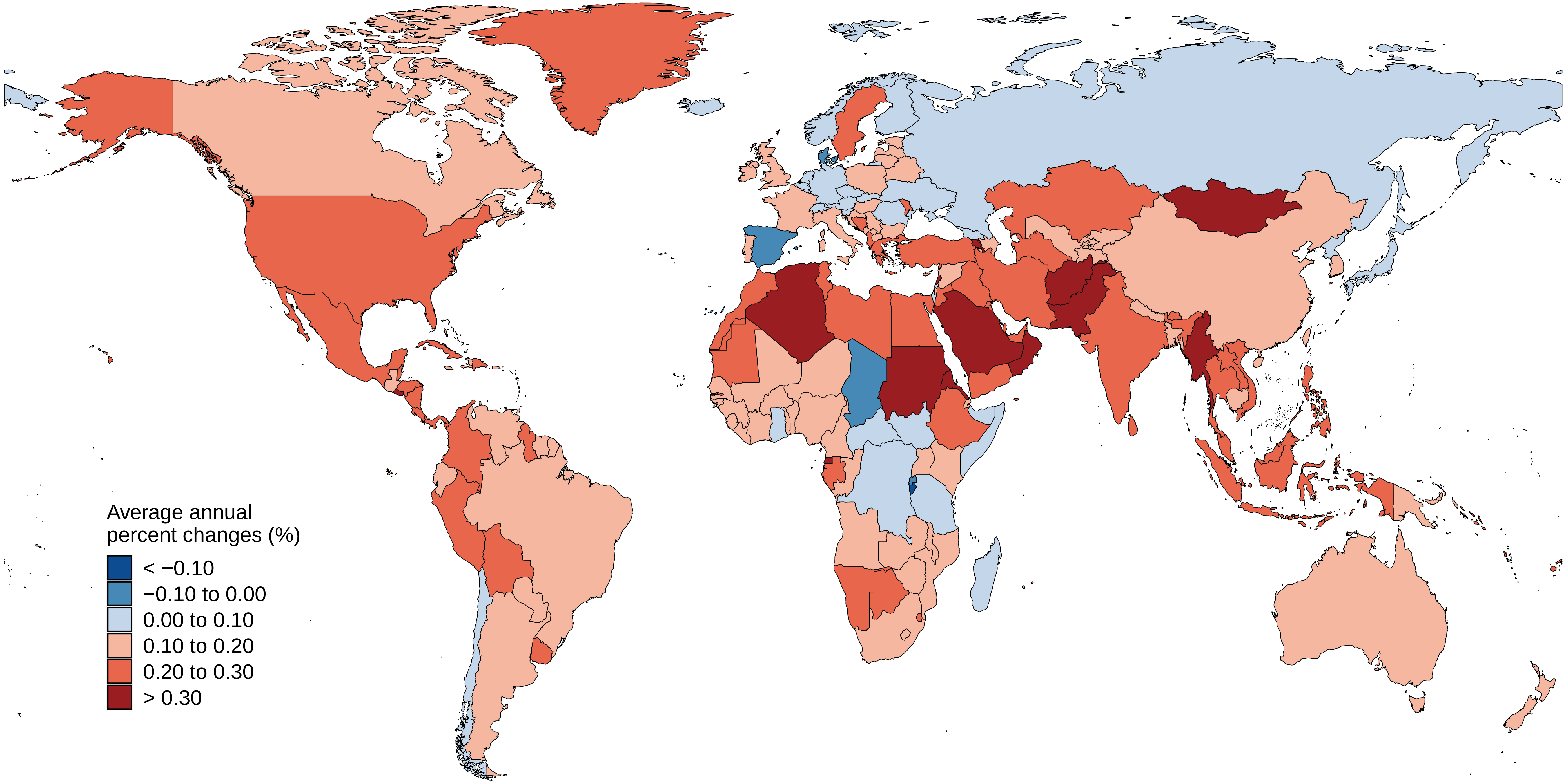

Supplementary Figure 16 Average annual percent changes in age-standardized prevalence rates for MSK disorders among adults aged 50 and over across 204 countries and territories, 1990-2021.

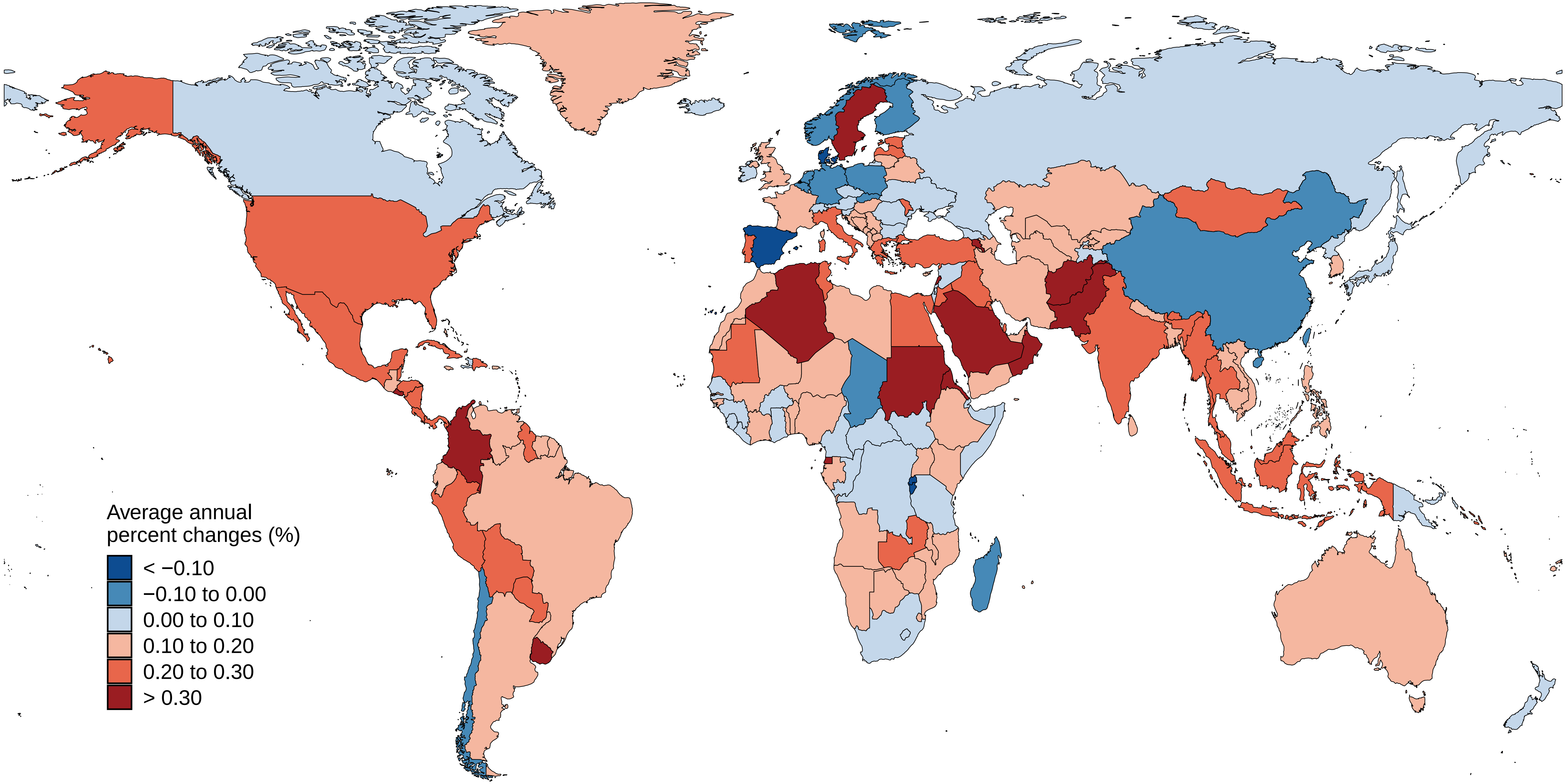

Supplementary Figure 17 Average annual percent changes in age-standardized DALY rates for MSK disorders among adults aged 50 and over across 204 countries and territories, 1990-2021.

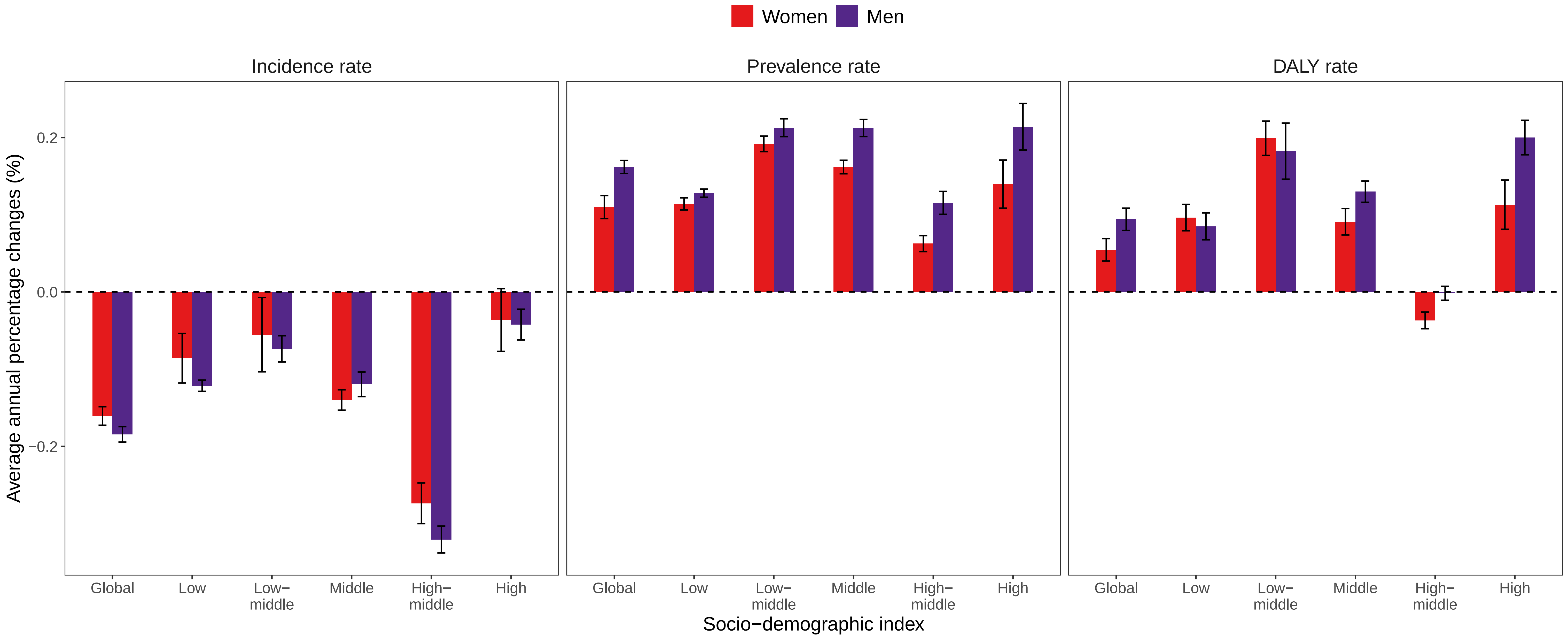

Supplementary Figure 18 Gender difference in average annual percent changes in global age-standardized incidence, prevalence and DALY rates for MSK disorders among adults aged 50 and over by SDI, 1990-2021.

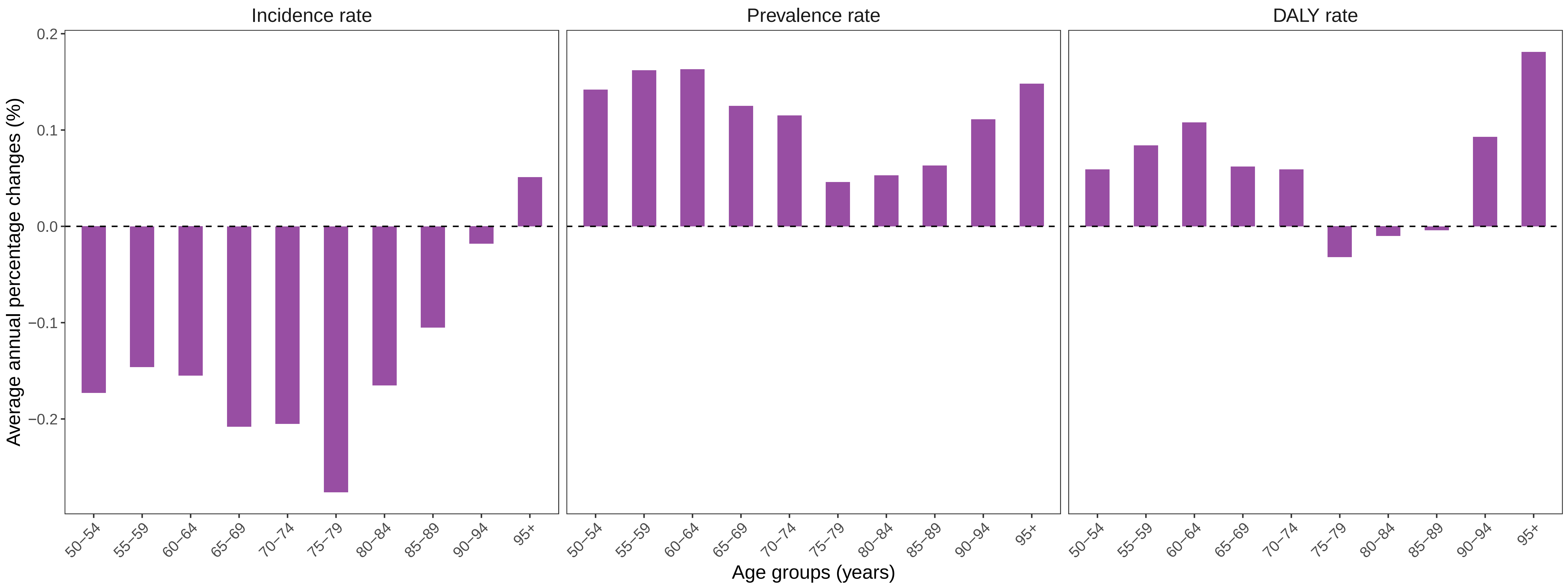

Supplementary Figure 19 Average annual percent changes in global age-specific incidence, prevalence and DALY rates for MSK disorders among adults aged 50 and over, 1990-2021.

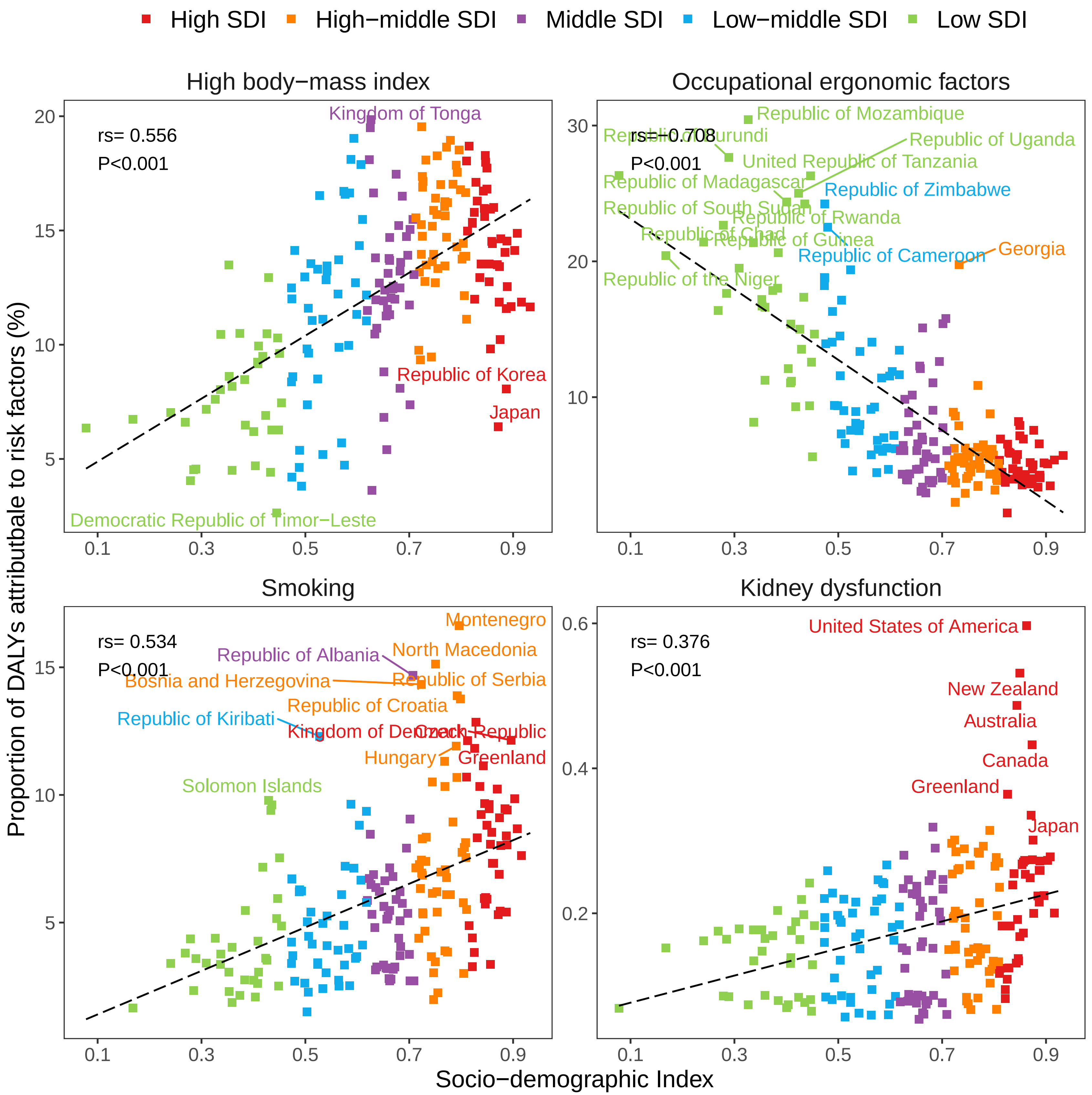

Supplementary Figure 20 Association between SDI and the proportions of DALYs attributable to high BMI, occupational ergonomic factors, smoking, and kidney dysfunction for MSK disorders among adults aged 50 and over across 204 countries and territories, 2021.

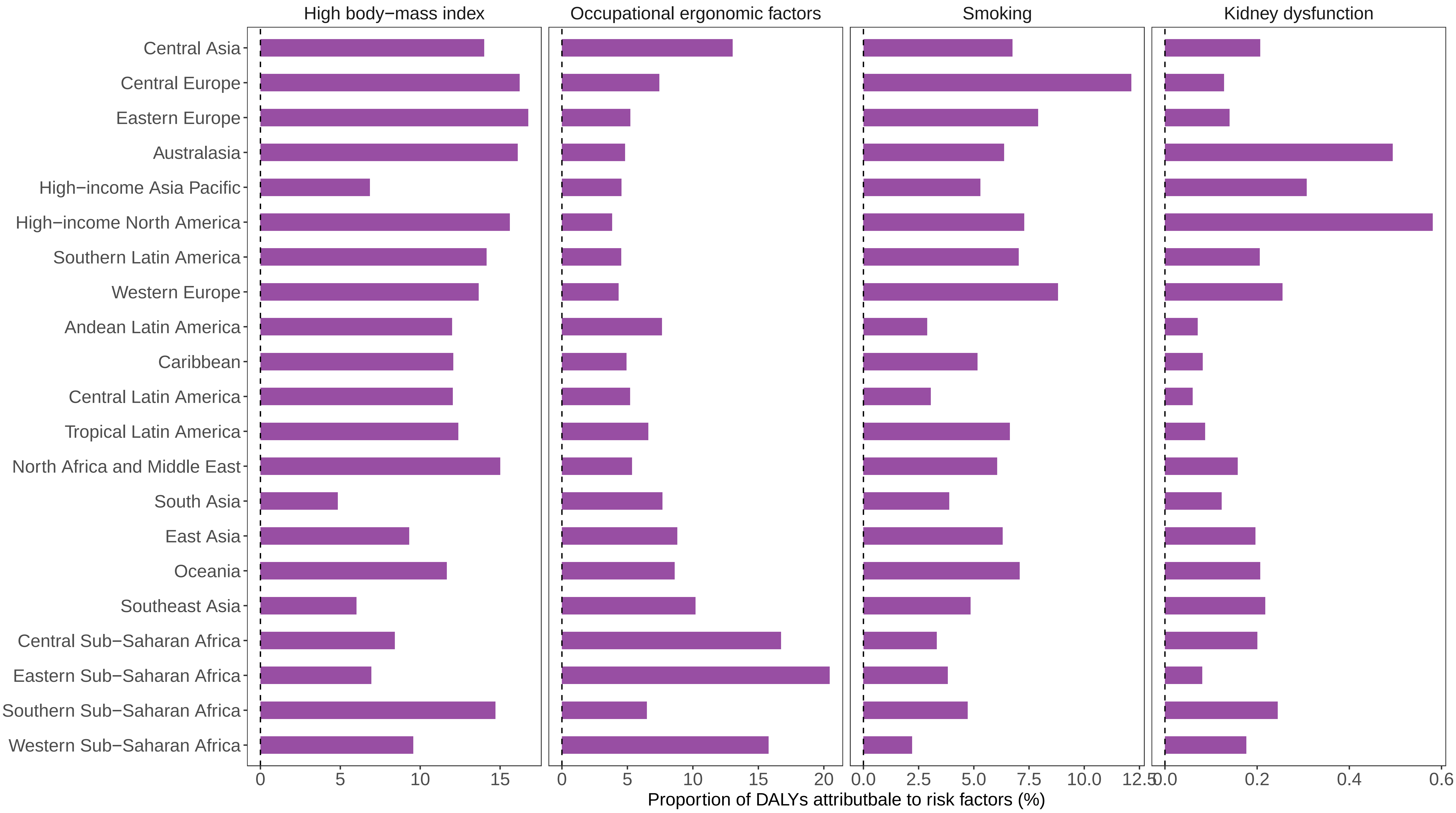

Supplementary Figure 21 The proportions of DALYs attributable to high BMI, occupational ergonomic factors, smoking, and kidney dysfunction for MSK disorders among adults aged 50 and over across 21 geographic regions, 2021.

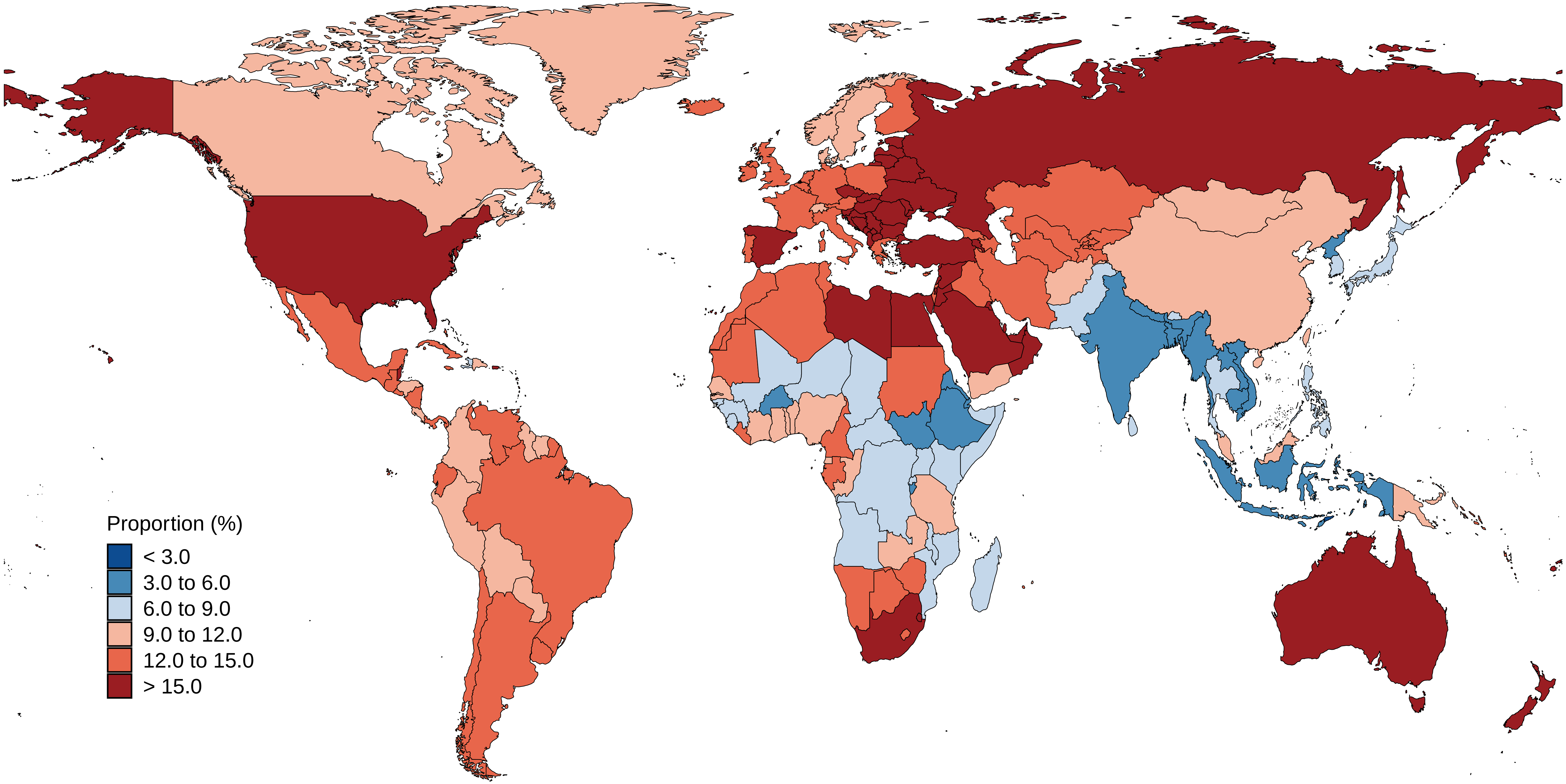

Supplementary Figure 22 The proportions of DALYs attributable to high BMI for MSK disorders among adults aged 50 and over across 204 countries and territories, 2021.

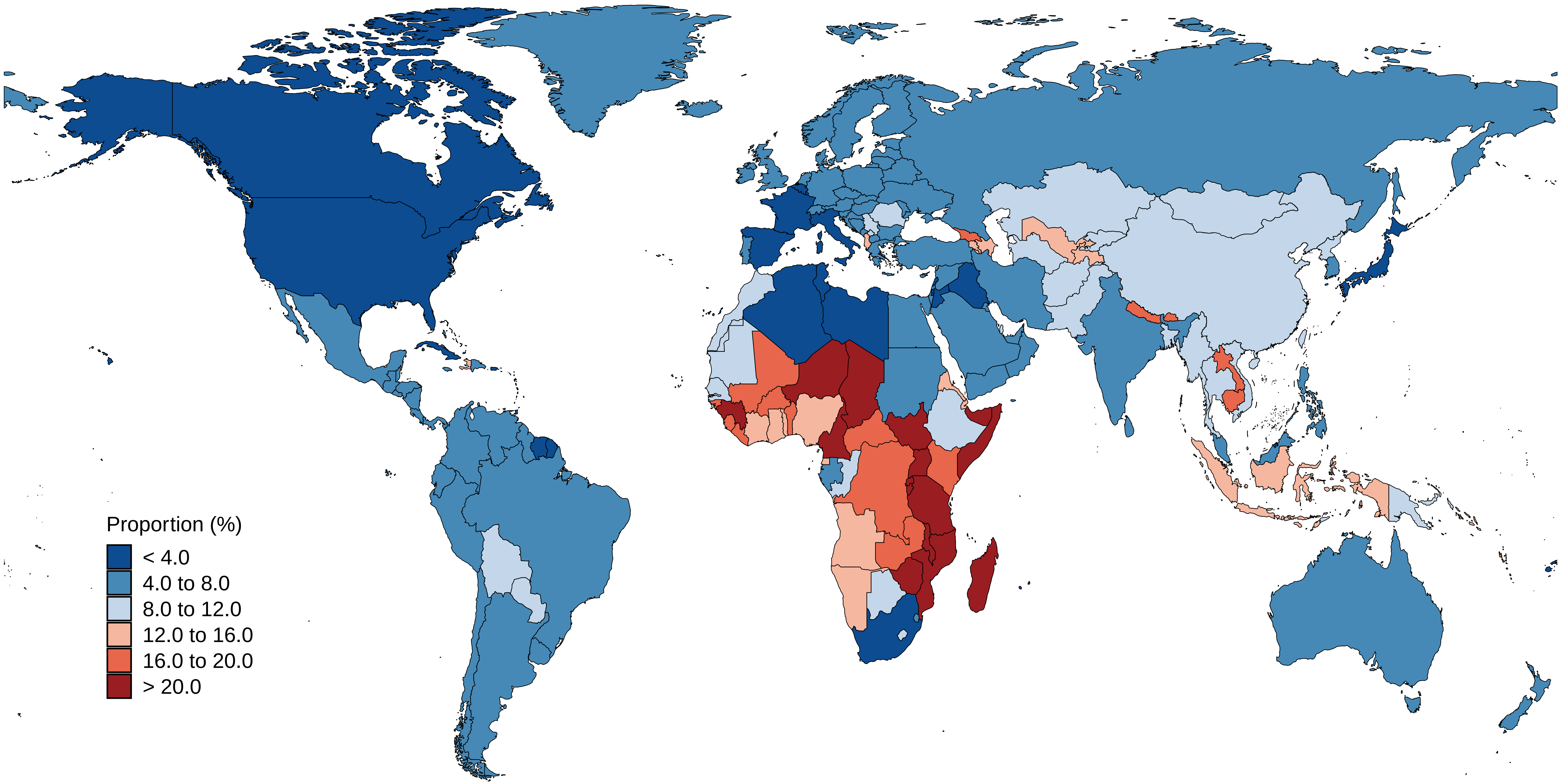

Supplementary Figure 23 The proportions of DALYs attributable to occupational ergonomic factors for MSK disorders among adults aged 50 and over across 204 countries and territories, 2021.

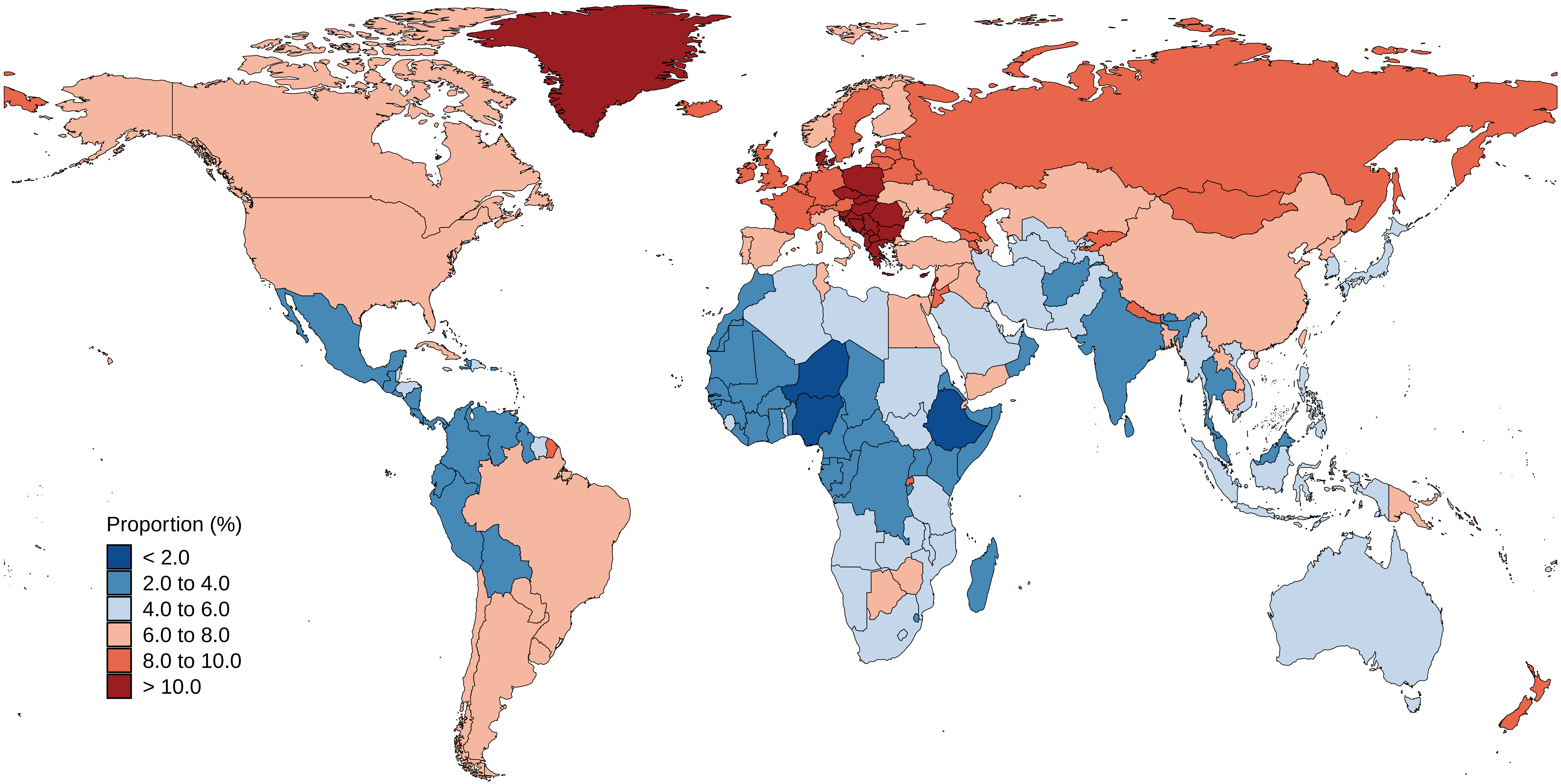

Supplementary Figure 24 The proportions of DALYs attributable to smoking for MSK disorders among adults aged 50 and over across 204 countries and territories, 2021.

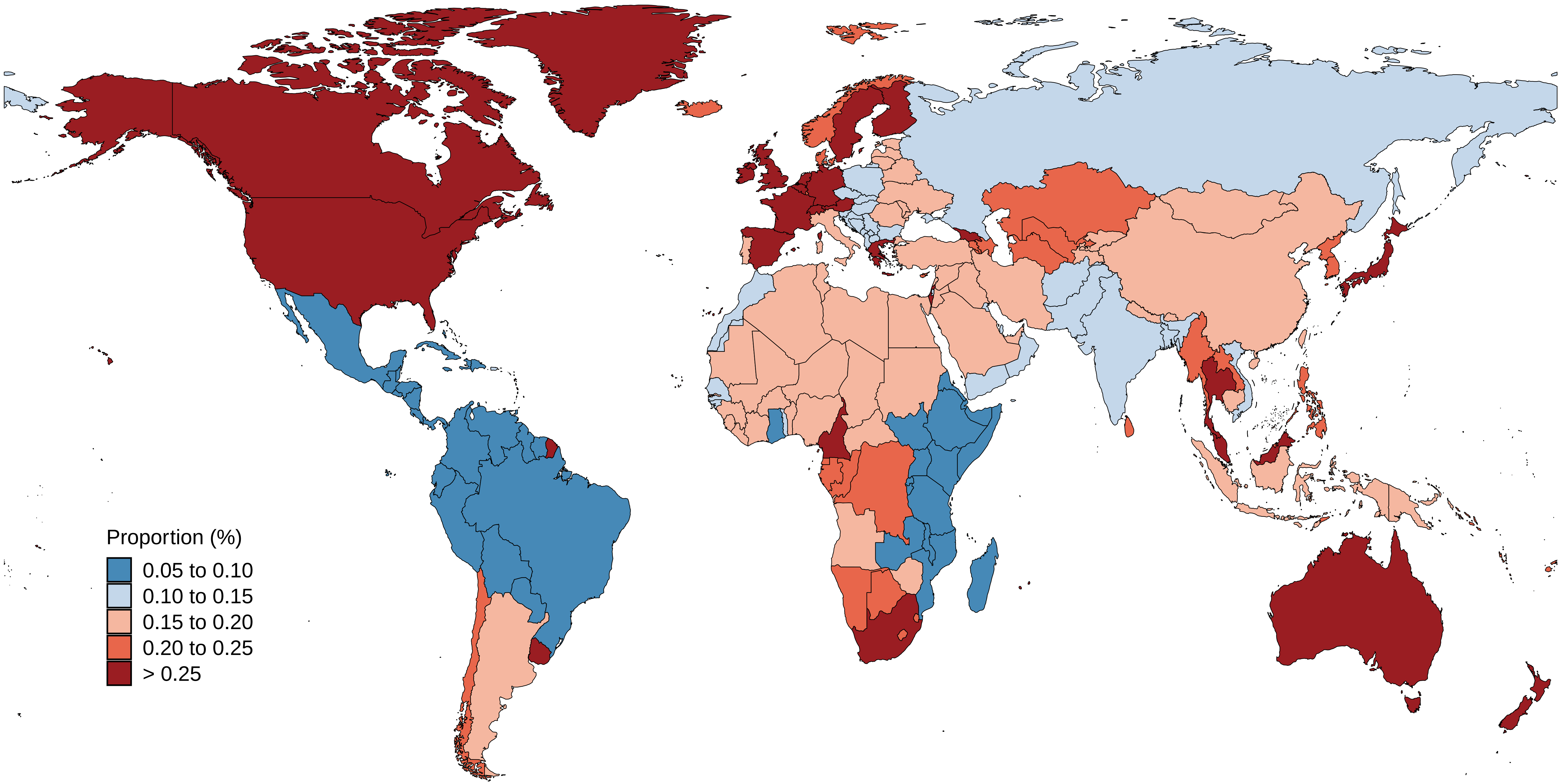

Supplementary Figure 25 The proportions of DALYs attributable to kidney dysfunction for MSK disorders among adults aged 50 and over across 204 countries and territories, 2021.

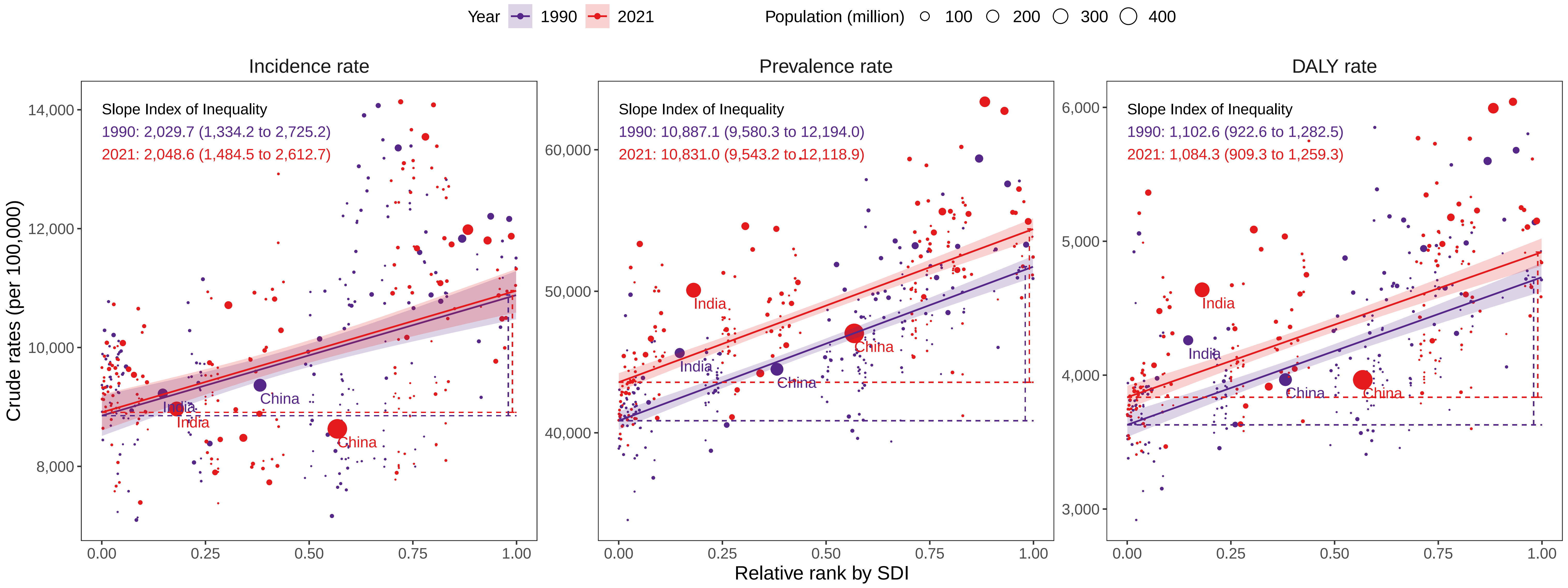

Supplementary Figure 26 Absolute SDI-related inequalities in incidence, prevalence and DALY rates for MSK disorders among adults aged 50 and over across 204 countries and territories, 1990-2021.
