# Supplementary material for: Global Burden of Musculoskeletal Disorders in Adults Aged 50 and Over, 1990–2021: Risk Factors and Sociodemographic Inequalities
Source: J Cachexia Sarcopenia Muscle. 2025 Jul 10;16(4):e70008. doi: 10.1002/jcsm.70008 (PMC12242706; doi:10.1002/jcsm.70008)
Supplement: Supplementary file 2 — Table S1. Case definitions, data resources, inclusion and exclusion criteria for musculoskeletal disorders 1. Table S2. The definitions, data resources, inclusion and exclusion criteria for risk factors of common musculoskeletal disorders. Table S3. Global numbers of incident cases, prevalent cases and DALYs and their percentage changes for MSK disorders among adults aged 50 and over by SDI and geographic regions, 1990‐2021. Table S4. Numbers of incident cases, prevalent cases and DALYs and their percentage changes for MSK disorders among adults aged 50 and over across 204 countries and territories, 1990‐2021. Table S5. Global age standardized incidence, prevalence and DALY rates (per 100000 population) and their average annual percentage changes for MSK disorders among adults aged 50 and over by SDI and geographic regions, 1990‐2021. Table S6. Age standardized incidence, prevalence and DALY rates (per 100000 population) and their average annual percentage changes for MSK disorders among adults aged 50 and over across 204 countries and territories, 1990‐2021. Table S7. Gender difference in global age standardized incidence, prevalence and DALY rates (per 100000 population) and their average annual percent changes for MSK disorders among adults aged 50 and over by SDI, 1990‐2021. Table S8. Global age‐specific incidence, prevalence and DALY rates (per 100000 population) and their average annual percent changes for MSK disorders among adults aged 50 and over, 1990‐2021. Table S9. Global DALYs attributable to main risk factors and their proportions to overall DALYs for MSK disorders among adults aged 50 and over by SDI and geographic regions, 1990‐2021. Table S10. DALYs attributable to main risk factors and their proportions to overall DALYs for MSK disorders among adults aged 50 and over across 204 countries and territories, 1990‐2021 62. Table S11. Gender difference in global DALYs attributable to main risk factors and their proportions to overall DALYs for MSK disord [file JCSM-16-e70008-s002.docx]

**Supplemntary Tables**

[**Supplementary Table 1** Case definitions, data resources, inclusion and exclusion criteria for musculoskeletal disorders 1](#_Toc180077470)

[**Supplementary Table 2** The definitions, data resources, inclusion and exclusion criteria for risk factors of common musculoskeletal disorders 5](#_Toc180077471)

[**Supplementary Table 3** Global numbers of incident cases, prevalent cases and DALYs and their percentage changes for MSK disorders among adults aged 50 and over by SDI and geographic regions, 1990-2021 8](#_Toc180077472)

[**Supplementary Table 4** Numbers of incident cases, prevalent cases and DALYs and their percentage changes for MSK disorders among adults aged 50 and over across 204 countries and territories, 1990-2021 9](#_Toc180077473)

[**Supplementary Table 5** Global age standardized incidence, prevalence and DALY rates (per 100000 population) and their average annual percentage changes for MSK disorders among adults aged 50 and over by SDI and geographic regions, 1990-2021 19](#_Toc180077474)

[**S**upplementary Table 6 Age standardized incidence, prevalence and DALY rates (per 100000 population) and their average annual percentage changes for MSK disorders among adults aged 50 and over across 204 countries and territories, 1990-2021 23](#_Toc180077475)

[**Supplementary Table 7** Gender difference in global age standardized incidence, prevalence and DALY rates (per 100000 population) and their average annual percent changes for MSK disorders among adults aged 50 and over by SDI, 1990-2021 53](#_Toc180077476)

[**Supplementary Table 8** Global age-specific incidence, prevalence and DALY rates (per 100000 population) and their average annual percent changes for MSK disorders among adults aged 50 and over, 1990-2021 55](#_Toc180077477)

[**Supplementary Table 9** Global DALYs attributable to main risk factors and their proportions to overall DALYs for MSK disorders among adults aged 50 and over by SDI and geographic regions, 1990-2021 57](#_Toc180077478)

[**Supplementary Table 10** DALYs attributable to main risk factors and their proportions to overall DALYs for MSK disorders among adults aged 50 and over across 204 countries and territories, 1990-2021 62](#_Toc180077479)

[**Supplementary Table 11** Gender difference in global DALYs attributable to main risk factors and their proportions to overall DALYs for MSK disorders among adults aged 50 and over by SDI and geographic regions, 1990-2021 100](#_Toc180077480)

| **Supplementary Table 1** Case definitions, data resources, inclusion and exclusion criteria for musculoskeletal disorders | | | | | | | | | | |
| --- | --- | --- | --- | --- | --- | --- | --- | --- | --- | --- |
| Categories | | Case definitions | | Data resources | | Inclusion criteria | Exclusion criteria | | ICD 10 codes | |
| Low back pain | | Low back pain is characterized by discomfort or pain in the region of the lower back, which may extend to one or both legs and persists for a minimum of 24 hours. The low back region is generally described as the area on the posterior side of the body, starting just below the twelfth rib and extending to the lower part of the buttocks. | | Systematic review was conducted using multiple electronic databases, including PubMed, Ovid Medline, Embase, and CINAHL. In addition to these databases, data from sources such as USA claims, World Health Surveys, and National Health Surveys were also included in the study. | | Individuals experiencing discomfort or pain in the region of the lower back; pain may extend to one or both legs; pain persists for a minimum of 24 hours. | Sub-populations not representative of the national population; non-population-based study design; small sample size (less than 150); literature review instead of original research studies | | M54.3, M54.4, M54.5 | |
| Neck pain | | Neck pain is characterized by discomfort or pain in the region of the neck, which may extend to the upper limbs and persists for a minimum of 24 hours. | | Systematic review was conducted using multiple databases, including Ovid MEDLINE, EMBASE, CINAHL, CAB abstracts, WHOLIS, SIGLE, and PUBMED. In addition to these databases, data from prominent surveys such as the National Health and Nutrition Examination Survey (NHANES), National Health Interview Survey (NHIS), as well as USA claims and Taiwan claims, were also utilized in the study. | | Individuals experiencing discomfort or pain in the region of the neck; pain may radiate to other areas such as the shoulders, arms, or head; pain persists for a minimum duration, often specified (e.g., 24 hours or more). | Sub-populations not representative of the national population; non-population-based study design; Small sample size (less than 150); studies on a specific type of neck pain (eg, following neck fracture) Studies that are literature reviews rather than original research studies. | | M54.2 | |
| Osteoarthritis | | Osteoarthritis (OA) is defined as the presence of radiologically confirmed Kellgren-Lawrence grade 2-4. Symptomatic grade 2 OA is characterized by the presence of one osteophyte in the affected joint and pain experienced for at least one month in the past 12 months. Symptomatic grade 3-4 OA requires the presence of osteophytes, joint space narrowing, and deformity in the affected joint, along with pain persisting for at least one month in the past 12 months. | | Systematic review was performed using the databases of MEDLINE, EMBASE, CINAHL, CAB Abstracts, WHO Library (WHOLIS), and OpenSIGLE. | | Individuals diagnosed with osteoarthritis based on radiographic assessment, typically using grading systems like the Kellgren-Lawrence grade; data sources reporting on the prevalence, incidence, or burden of osteoarthritis in specific populations or regions; studies providing data on the impact of osteoarthritis on morbidity, mortality, or disability. | Sub-populations not representative of the national or global population; non-population-based study designs, such as case reports or case series; studies with small sample sizes (less than 150) or insufficient data to reliably estimate the burden of osteoarthritis; data sources lacking diagnostic specificity or reliability. | | M16, M17, M18, M19 | |
| Gout | | The diagnosis of gout is based on the 1977 survey criteria established by the American Rheumatism Association (ARA). It requires the presence of specific criteria for a confirmed diagnosis. These criteria include the detection of monosodium urate (MSU) crystals either in the joint fluid or in a tophus containing MSU crystals. Additionally, at least six out of twelve gout symptoms or findings must be present. These symptoms include experiencing an acute arthritis attack, rapid development of maximum inflammation within a day, arthritis affecting a single joint (monoarticular arthritis), redness, pain, or swelling in the first metatarsophalangeal (MTP) joint, an attack specifically targeting the first MTP joint or tarsal joint, the presence of a suspected tophus, elevated levels of uric acid in the blood (hyperuricemia), asymmetrical joint swelling observed on X-ray, and negative results in joint fluid culture for microorganisms during a joint inflammation episode. | | Systematic review was conducted using multiple databases, including MEDLINE, EMBASE, CINAHL, CAB Abstracts, WHO Library (WHOLIS), and OpenSIGLE. Additionally, claims data from the United States and Taiwan were also included in the analysis. | | Individuals diagnosed with gout based on established clinical criteria, such as the presence of characteristic symptoms (e.g., acute joint pain, swelling, redness) and elevated serum urate levels; data sources reporting on the prevalence, incidence, or burden of gout in specific populations or regions; studies providing data on the impact of gout on morbidity, mortality, or disability. | Data from sub-populations not representative of the national or global population; non-population-based study designs, such as case reports or case series; studies with small sample sizes (less than 150) or insufficient data to reliably estimate the burden of gout; data sources lacking diagnostic specificity or reliability. | | M10 | |
| Rheumatoid arthritis | | The reference definition for rheumatoid arthritis (RA) is based on the 1987 criteria established by the American College of Rheumatology (ACR 1987). According to these criteria, a diagnosis of RA requires the presence of at least four out of seven diagnostic factors, with the first four persisting for a minimum of six weeks. These criteria encompass morning stiffness, arthritis in three or more joints, arthritis in hand joints, symmetrical arthritis, rheumatoid nodules, presence of serum rheumatoid factor, and radiographic changes. | | Systematic review utilized multiple databases, such as Ovid MEDLINE, EMBASE, CINAHL, CAB abstracts, WHOLIS, and SIGLE. Additionally, data from claims databases in the United States and Taiwan were included to ensure a comprehensive analysis. | | Individuals diagnosed with rheumatoid arthritis based on established clinical criteria, such as those outlined by the American College of Rheumatology; data sources reporting on the prevalence, incidence, or burden of rheumatoid arthritis in specific populations or regions; studies providing data on the impact of rheumatoid arthritis on morbidity, mortality, or disability. | Studies clearly not representative of the national population; studies that were not population-based, such as hospital or clinic-based studies; studies that did not provide primary data on epidemiological parameters, such as commentary pieces; studies focusing on a specific type of RA, such as seropositive RA; studies with a sample size of less than 150 participants; reviews. | | M05, M06, M08 | |
| Other musculoskeletal disorders | | Other musculoskeletal disorders is a heterogeneous rest category comprising a wide range of disorders of muscles, bones, and ligaments that are not included in the five GBD defined musculoskeletal diseases – rheumatoid arthritis, osteoarthritis, low back pain, neck pain, and gout – and are not captured as longterm sequelae of injuries. | | The ICD codes were used to extract other MSK prevalence from USA claims data for 2000 and 2010–2016 by state. The systematic review concentrated on finding health surveys that measured an overall amount of musculoskeletal disorders and reported information to distinguish a rest category that was not OA, RA, gout, or low back or neck pain. These data sources are based on self-reported musculoskeletal conditions or symptoms and did not use the listed ICD codes. | | Lupus erythematosus, infectious arthropathies, inflammatory polyarthropathies, other joint disorders, systemic connective tissue disorders, deforming dorsopathies, spondylopathies, disorders of the muscles, disorders of synovium and tendon, other soft tissue disorders, disorders of bone density and structure, osteomyelitis, other otseopathies, chondropathies, other disorders of the musculoskeletal system and connective tissue. | Not included under gout, rheumatoid arthritis, osteoarthritis, low back pain, or neck pain. | | L93;  M00-M0; M08, M11-M13; M20-M25; M30-M35;  M40-M43; M45-M46; M60 -M63; M65-M68; M70- M73, M75-M79;  M80-M85; M86; M87-M90; M91-M94; M95-M99. | |
| **Supplementary Table 2** The definitions, data resources, inclusion and exclusion criteria for risk factors of common musculoskeletal disorders | | | | | | | | | |  |
| Risk factors | Definitions | | Data resources | | Inclusion criteria | | | Exclusion criteria | |  |
| Occupational ergonomic factors | Occupational ergonomic factors encompass a variety of physical stressors and conditions encountered in the workplace that can affect musculoskeletal health, particularly contributing to low back pain (LBP). These factors include physical exertion, demanding postures, repetitive work tasks, exposure to hand-arm vibration, activities like kneeling or squatting, as well as tasks involving rising and climbing. However, these exposures haven not been adequately measured or characterized on a global scale. Therefore, occupation is used as a proxy for these ergonomic factors, with occupations grouped into broad risk categories based on economic sector and similar physical and psychosocial exposures. The proportion of the working population exposed to these occupational factors associated with LBP is determined by analyzing population distributions across nine occupational categories. | | Initial data for the analysis were sourced from the International Labour Organization (ILO), encompassing various parameters such as proportions of economic activities and occupations, fatal injury rates, and employment-to-population ratios. Additional datasets were acquired through requests to agencies and collaboration with the GBD network. | | Nine distinct occupational categories, including legislators, senior officials, managers, professionals, technicians, clerks, service workers, skilled agricultural workers, machine operators, craft workers, and those in elementary occupations. These assessments were conducted for individuals aged 15 and older. | | | Informal employment statistics were omitted due to data limitations. Additionally, assessments of occupational exposures were not carried out for individuals under the age of 15. | |  |
| High body-mass index | For adults aged 20 and above, a high body mass index (BMI) is typically defined as having a BMI greater than 20 to 25 kg/m^2^. For children aged 1 to 19, a high BMI is determined based on the International Obesity Task Force standards, which classify individuals as overweight or obese. | | In GBD 2019, new data were incorporated from sources included in the annual GHDx update of established survey series. A systematic review was conducted in GBD 2017 to identify studies offering nationally or subnationally representative estimates of overweight prevalence, obesity prevalence, or mean body-mass index (BMI). The search was confined to literature published between January 1, 2016, and December 31, 2016, to refresh the systematic literature search previously conducted as part of GBD 2015. | | Representative studies providing data on mean BMI or prevalence of overweight or obesity among adults or children were included. Regarding adults, studies met the inclusion criteria if they defined overweight as BMI≥25 kg/m2 and obesity as BMI≥30 kg/m2, or if estimates using those thresholds could be inferred from reported categories. For children (ages 2–19), studies were included if they utilized International Obesity Task Force (IOTF) standards to define overweight and obesity thresholds. Only studies reporting data collected after January 1, 1980, were considered. | | | Exclusion criteria encompassed studies using non-random samples (e.g., case-control studies or convenience samples), conducted among specific subpopulations (e.g., pregnant women, racial or ethnic minorities, immigrants, or individuals with specific diseases), employing alternative methods to assess adiposity (e.g., waist circumference, skin-fold thickness, or hydrodensitometry), featuring sample sizes of less than 20 per age-sex group, or providing insufficient information on any of the inclusion criteria. Additionally, review articles and non-English-language articles were excluded. | |  |
| Smoking | The prevalence of current smoking and former smoking was estimated using data from cross-sectional nationally representative household surveys. Current smokers were defined as individuals who currently use any smoked tobacco product daily or occasionally. Former smokers were defined as individuals who ceased using all smoked tobacco products for at least six months, if possible, or as per the survey's definition. | | Primary data was extracted from individual-level microdata and survey report tabulations. Data on current, former, and/or ever smoked tobacco use was collected, considering various combinations of frequency of use and type of smoked tobacco products. Other tobacco product variants, such as hand-rolled cigarettes, were grouped into the specified type categories. | | All smoked tobacco products, such as cigarettes, pipes, cigars, shisha, bidis, kreteks, and other local variants, were included in the analysis. Data were collected from January 1, 1980, to December 31, 2019. Information from 1980-1989 was included to inform time trends, although details from this period are not reported. Tobacco use was self-reported by individuals, not by proxies. Respondents were aged 10 and older. | | | Smokeless tobacco, electronic cigarettes (also known as e-cigarettes), vaping products, and heated tobacco products were not included in the study. Risks associated with chewing tobacco and second-hand smoke were considered as other risk factors in GBD and were beyond the scope of this study. | |  |
| kidney dysfunction | The kidney dysfunction risk factor exposure is divided into four categories of renal function defined by urinary albumin to creatinine ratio (ACR) and estimated glomerular filtration rate (eGFR): 1. Albuminuria with preserved eGFR (ACR >30 mg/g & eGFR >=60 ml/min/1.73m2); this corresponds to stages 1 and 2 chronic kidney disease (CKD) in the Kidney Disease Improving Global Outcomes (KDIGO) classification 2. CKD stage 3 (eGFR of 30-59 ml/min/1.73m2); 3. CKD stage 4 (eGFR of 15-29 ml/min/1.73m2); and 4. CKD stage 5 (eGFR <15ml/min/1.73m2, not (yet) on renal replacement therapy) | | The last systematic review of prevalence of low glomerular filtration rate was conducted for GBD 2017, updating searches done in GBD 2016, GBD 2015, GBD 2013, and GBD 2010. The prevalence of exposure to Stage 1-2, Stage 3, Stage 4 and Stage 5 CKD were obtained from the GBD 2021 non-fatal burden of disease analysis. | | Data on relative risks were contributed by the Chronic Kidney Disease Prognosis Consortium (CKD-PC). The Chronic Kidney Disease Prognosis Consortium is a research group composed of investigators representing cohorts from around the world. Investigators share data for the purpose of collaborative meta-analyses to study prognosis in CKD. | | | Surveys that were not population-representative and studies not reporting on CKD by stage | |  |

| **Supplementary Table 3** Global numbers of incident cases, prevalent cases and DALYs and their percentage changes for MSK disorders among adults aged 50 and over by SDI and geographic regions, 1990-2021 | | | | | | | | | | | | | | | | | | |
| --- | --- | --- | --- | --- | --- | --- | --- | --- | --- | --- | --- | --- | --- | --- | --- | --- | --- | --- |
| Location name | | Incident cases | | | | | Prevalent cases | | | | | DALYs | | | | | | |
|  |  | 1990 | 2021 | | Percentage changes (%) | | 1990 | | 2021 | Percentage changes (%) | | 1990 | | | 2021 | | Percentage changes (%) | |
| Global | | 91617476 | 190075244 | | 107.5 | | 426665318 | | 977642796 | 129.1 | | 39513134 | | | 88432912 | | 123.8 | |
| High SDI | | 26884624 | 47753950 | | 77.6 | | 126833271 | | 242168083 | 90.9 | | 11999074 | | | 22563236 | | 88 | |
| High-middle SDI | | 25197611 | 45183173 | | 79.3 | | 109340805 | | 222075825 | 103.1 | | 10090996 | | | 19706909 | | 95.3 | |
| Middle SDI | | 21669902 | 55985060 | | 158.4 | | 106364131 | | 303873334 | 185.7 | | 9589406 | | | 26714630 | | 178.6 | |
| Low-middle SDI | | 12843081 | 30249780 | | 135.5 | | 61725857 | | 157494362 | 155.2 | | 5769447 | | | 14691940 | | 154.7 | |
| Low SDI | | 4911196 | 10726974 | | 118.4 | | 21928753 | | 51210268 | 133.5 | | 2020358 | | | 4682243 | | 131.8 | |
| Central Asia | | 1204701 | 2135021 | | 77.2 | | 4936212 | | 9254373 | 87.5 | | 447104 | | | 830032 | | 85.6 | |
| Central Europe | | 4434813 | 6034531 | | 36.1 | | 16987118 | | 24316053 | 43.1 | | 1639822 | | | 2295025 | | 40 | |
| Eastern Europe | | 8698483 | 10201058 | | 17.3 | | 34282791 | | 41751407 | 21.8 | | 3212661 | | | 3900095 | | 21.4 | |
| Australasia | | 592288 | 1289331 | | 117.7 | | 2766031 | | 6426695 | 132.3 | | 268807 | | | 611175 | | 127.4 | |
| High-income Asia Pacific | | 5415948 | 9680257 | | 78.7 | | 25688486 | | 51901434 | 102 | | 2498920 | | | 4902617 | | 96.2 | |
| High-income North America | | 8248771 | 15958231 | | 93.5 | | 41549773 | | 84993036 | 104.6 | | 3929556 | | | 8036190 | | 104.5 | |
| Southern Latin America | | 1080914 | 2028199 | | 87.6 | | 5676975 | | 10960569 | 93.1 | | 553578 | | | 1065557 | | 92.5 | |
| Western Europe | | 13529460 | 20405433 | | 50.8 | | 61373260 | | 96109704 | 56.6 | | 5714950 | | | 8799158 | | 54 | |
| Andean Latin America | | 349047 | 1052420 | | 201.5 | | 2044391 | | 6423537 | 214.2 | | 176027 | | | 553629 | | 214.5 | |
| Caribbean | | 444844 | 964044 | | 116.7 | | 2456765 | | 5620369 | 128.8 | | 205452 | | | 467492 | | 127.5 | |
| Central Latin America | | 1580044 | 4986947 | | 215.6 | | 9012769 | | 30005710 | 232.9 | | 829405 | | | 2772845 | | 234.3 | |
| Tropical Latin America | | 2064452 | 6139286 | | 197.4 | | 10579657 | | 31350005 | 196.3 | | 992786 | | | 2920125 | | 194.1 | |
| North Africa and Middle East | | 3812087 | 10537517 | | 176.4 | | 16863339 | | 50372892 | 198.7 | | 1568813 | | | 4636467 | | 195.5 | |
| South Asia | | 12185269 | 30439023 | | 149.8 | | 59837170 | | 166801018 | 178.8 | | 5642575 | | | 15645295 | | 177.3 | |
| East Asia | | 18624859 | 44886875 | | 141 | | 88551905 | | 243894089 | 175.4 | | 7901461 | | | 20641924 | | 161.2 | |
| Oceania | | 57506 | 151469 | | 163.4 | | 280277 | | 762096 | 171.9 | | 24123 | | | 64436 | | 167.1 | |
| Southeast Asia | | 4633064 | 12586697 | | 171.7 | | 23202552 | | 67555209 | 191.2 | | 2059599 | | | 5918922 | | 187.4 | |
| Central Sub-Saharan Africa | | 519636 | 1260835 | | 142.6 | | 2264528 | | 5684250 | 151 | | 204966 | | | 511453 | | 149.5 | |
| Eastern Sub-Saharan Africa | | 1681287 | 3773459 | | 124.4 | | 7056725 | | 16743150 | 137.3 | | 641563 | | | 1495884 | | 133.2 | |
| Southern Sub-Saharan Africa | | 603857 | 1292610 | | 114.1 | | 2801270 | | 6388049 | 128 | | 251776 | | | 562381 | | 123.4 | |
| Western Sub-Saharan Africa | | 1856147 | 4272001 | | 130.2 | | 8453322 | | 20329151 | 140.5 | | 749190 | | | 1802212 | | 140.6 | |
| DALYs, disability-adjusted life-years; MSK disorders, musculoskeletal disorders; SDI, Socio-demographic Index. | | | | | | | | | | | | | | | | | | |
| **Supplementary Table 4** Numbers of incident cases, prevalent cases and DALYs and their percentage changes for MSK disorders among adults aged 50 and over across 204 countries and territories, 1990-2021 | | | | | | | | | | | | | | | | | |  |
| Location name | Incident cases | | | | | | | Prevalent cases | | | | | DALYs | | | | |  |
|  | 1990 | | | 2021 | | Percentage changes (%) | | 1990 | 2021 | | Percentage changes (%) | | 1990 | 2021 | | Percentage changes (%) | |  |
| Afghanistan | 152759 | | | 209188 | | 36.9 | | 629273 | 907371 | | 44.2 | | 56968 | 82785 | | 45.3 | |  |
| Albania | 59219 | | | 124661 | | 110.5 | | 215209 | 483198 | | 124.5 | | 21185 | 46857 | | 121.2 | |  |
| Algeria | 271419 | | | 808191 | | 197.8 | | 1227506 | 3988888 | | 225 | | 112623 | 362960 | | 222.3 | |  |
| American Samoa | 465 | | | 1064 | | 129 | | 2425 | 5736 | | 136.6 | | 206 | 477 | | 131.1 | |  |
| Andorra | 1358 | | | 3653 | | 168.9 | | 6070 | 16895 | | 178.4 | | 543 | 1473 | | 171 | |  |
| Angola | 89669 | | | 274357 | | 206 | | 393529 | 1277137 | | 224.5 | | 35257 | 112962 | | 220.4 | |  |
| Antigua and Barbuda | 857 | | | 2009 | | 134.4 | | 5009 | 11900 | | 137.6 | | 417 | 1001 | | 140.3 | |  |
| Argentina | 757957 | | | 1283690 | | 69.4 | | 3945528 | 6980953 | | 76.9 | | 381577 | 678754 | | 77.9 | |  |
| Armenia | 74072 | | | 111356 | | 50.3 | | 289209 | 482019 | | 66.7 | | 26457 | 43755 | | 65.4 | |  |
| Australia | 488077 | | | 1069626 | | 119.2 | | 2323495 | 5439098 | | 134.1 | | 227653 | 520988 | | 128.9 | |  |
| Austria | 261379 | | | 382873 | | 46.5 | | 1195519 | 1862509 | | 55.8 | | 108956 | 165979 | | 52.3 | |  |
| Azerbaijan | 132388 | | | 281988 | | 113 | | 550350 | 1221899 | | 122 | | 49724 | 109426 | | 120.1 | |  |
| Bahamas | 2693 | | | 7810 | | 190 | | 15580 | 46513 | | 198.5 | | 1342 | 3985 | | 196.9 | |  |
| Bahrain | 3863 | | | 23201 | | 500.5 | | 18658 | 118418 | | 534.7 | | 1706 | 10797 | | 532.8 | |  |
| Bangladesh | 1044680 | | | 3113936 | | 198.1 | | 5091040 | 16487087 | | 223.8 | | 517570 | 1657731 | | 220.3 | |  |
| Barbados | 4701 | | | 9250 | | 96.8 | | 27822 | 56233 | | 102.1 | | 2407 | 4828 | | 100.6 | |  |
| Belarus | 369054 | | | 440332 | | 19.3 | | 1495234 | 1874334 | | 25.4 | | 136815 | 169464 | | 23.9 | |  |
| Belgium | 361830 | | | 507278 | | 40.2 | | 1614309 | 2352404 | | 45.7 | | 148044 | 209065 | | 41.2 | |  |
| Belize | 1577 | | | 5538 | | 251.3 | | 8753 | 31731 | | 262.5 | | 733 | 2659 | | 262.6 | |  |
| Benin | 40282 | | | 107990 | | 168.1 | | 183377 | 521073 | | 184.2 | | 16242 | 45560 | | 180.5 | |  |
| Bermuda | 1131 | | | 2367 | | 109.4 | | 6592 | 14549 | | 120.7 | | 563 | 1228 | | 118.2 | |  |
| Bhutan | 5453 | | | 13247 | | 142.9 | | 25608 | 66915 | | 161.3 | | 2536 | 6595 | | 160 | |  |
| Bolivia (Plurinational State of) | 55663 | | | 169031 | | 203.7 | | 310637 | 988268 | | 218.1 | | 27156 | 86285 | | 217.7 | |  |
| Bosnia and Herzegovina | 123201 | | | 169490 | | 37.6 | | 458106 | 684519 | | 49.4 | | 44163 | 64152 | | 45.3 | |  |
| Botswana | 11729 | | | 31782 | | 171 | | 54893 | 157679 | | 187.2 | | 4942 | 13679 | | 176.8 | |  |
| Brazil | 2022448 | | | 6020695 | | 197.7 | | 10344806 | 30685334 | | 196.6 | | 971836 | 2859147 | | 194.2 | |  |
| Brunei Darussalam | 2244 | | | 8593 | | 282.8 | | 11375 | 46093 | | 305.2 | | 1028 | 4183 | | 306.8 | |  |
| Bulgaria | 358204 | | | 373875 | | 4.4 | | 1393713 | 1538695 | | 10.4 | | 130589 | 141806 | | 8.6 | |  |
| Burkina Faso | 90769 | | | 189784 | | 109.1 | | 400539 | 877949 | | 119.2 | | 36057 | 78523 | | 117.8 | |  |
| Burundi | 50796 | | | 105898 | | 108.5 | | 215183 | 443954 | | 106.3 | | 19815 | 39624 | | 100 | |  |
| Cabo Verde | 4595 | | | 9252 | | 101.3 | | 21581 | 46370 | | 114.9 | | 1908 | 3999 | | 109.5 | |  |
| Cambodia | 83489 | | | 234851 | | 181.3 | | 391041 | 1194093 | | 205.4 | | 35451 | 105935 | | 198.8 | |  |
| Cameroon | 98256 | | | 271165 | | 176 | | 447220 | 1304531 | | 191.7 | | 40217 | 115436 | | 187 | |  |
| Canada | 682653 | | | 1432325 | | 109.8 | | 3578252 | 8148778 | | 127.7 | | 348719 | 769959 | | 120.8 | |  |
| Central African Republic | 25864 | | | 50741 | | 96.2 | | 110458 | 220416 | | 99.5 | | 10064 | 20008 | | 98.8 | |  |
| Chad | 60215 | | | 122865 | | 104 | | 262070 | 548231 | | 109.2 | | 23995 | 49356 | | 105.7 | |  |
| Chile | 236132 | | | 621717 | | 163.3 | | 1275676 | 3322955 | | 160.5 | | 128926 | 323213 | | 150.7 | |  |
| China | 17908807 | | | 43140113 | | 140.9 | | 85076416 | 235039035 | | 176.3 | | 7585091 | 19818337 | | 161.3 | |  |
| Colombia | 343317 | | | 1104747 | | 221.8 | | 1877270 | 6531392 | | 247.9 | | 173781 | 609353 | | 250.6 | |  |
| Comoros | 4159 | | | 10699 | | 157.2 | | 18308 | 49082 | | 168.1 | | 1638 | 4375 | | 167.1 | |  |
| Congo | 23637 | | | 61408 | | 159.8 | | 107573 | 289558 | | 169.2 | | 9705 | 25673 | | 164.5 | |  |
| Cook Islands | 253 | | | 554 | | 118.9 | | 1323 | 3033 | | 129.3 | | 112 | 251 | | 125.5 | |  |
| Costa Rica | 32663 | | | 107802 | | 230 | | 185886 | 650126 | | 249.7 | | 17172 | 60188 | | 250.5 | |  |
| Croatia | 174814 | | | 228915 | | 30.9 | | 686282 | 941764 | | 37.2 | | 64134 | 87366 | | 36.2 | |  |
| Cuba | 174586 | | | 351239 | | 101.2 | | 949670 | 2068526 | | 117.8 | | 78483 | 171395 | | 118.4 | |  |
| Cyprus | 19630 | | | 48079 | | 144.9 | | 85505 | 224107 | | 162.1 | | 8196 | 20996 | | 156.2 | |  |
| Czechia | 386144 | | | 562119 | | 45.6 | | 1523201 | 2316245 | | 52.1 | | 143268 | 216289 | | 51 | |  |
| C么te d'Ivoire | 85996 | | | 238606 | | 177.5 | | 386108 | 1130966 | | 192.9 | | 34169 | 100032 | | 192.8 | |  |
| Democratic People's Republic of Korea | 372245 | | | 733316 | | 97 | | 1758933 | 3679800 | | 109.2 | | 163155 | 335881 | | 105.9 | |  |
| Democratic Republic of the Congo | 363569 | | | 838836 | | 130.7 | | 1577309 | 3726464 | | 136.3 | | 143116 | 337682 | | 135.9 | |  |
| Denmark | 187242 | | | 254657 | | 36 | | 917718 | 1310280 | | 42.8 | | 92143 | 130601 | | 41.7 | |  |
| Djibouti | 2913 | | | 13918 | | 377.8 | | 12680 | 65145 | | 413.8 | | 1147 | 5690 | | 396.1 | |  |
| Dominica | 1021 | | | 1573 | | 54 | | 5703 | 9262 | | 62.4 | | 487 | 778 | | 59.9 | |  |
| Dominican Republic | 63532 | | | 179761 | | 182.9 | | 353783 | 1038910 | | 193.7 | | 29515 | 86185 | | 192 | |  |
| Ecuador | 91263 | | | 287722 | | 215.3 | | 531357 | 1774564 | | 234 | | 45722 | 150898 | | 230 | |  |
| Egypt | 593808 | | | 1493333 | | 151.5 | | 2712543 | 7248223 | | 167.2 | | 249657 | 666139 | | 166.8 | |  |
| El Salvador | 55091 | | | 117912 | | 114 | | 301430 | 684951 | | 127.2 | | 26602 | 60982 | | 129.2 | |  |
| Equatorial Guinea | 4372 | | | 11396 | | 160.7 | | 18642 | 54661 | | 193.2 | | 1703 | 4875 | | 186.3 | |  |
| Eritrea | 23675 | | | 58651 | | 147.7 | | 92240 | 254325 | | 175.7 | | 7954 | 22149 | | 178.5 | |  |
| Estonia | 57175 | | | 66894 | | 17 | | 235327 | 294616 | | 25.2 | | 21937 | 27756 | | 26.5 | |  |
| Eswatini | 5689 | | | 11397 | | 100.3 | | 27198 | 57620 | | 111.9 | | 2419 | 4982 | | 106 | |  |
| Ethiopia | 462371 | | | 962859 | | 108.2 | | 1880962 | 4339146 | | 130.7 | | 171338 | 379368 | | 121.4 | |  |
| Fiji | 7173 | | | 16470 | | 129.6 | | 36279 | 88770 | | 144.7 | | 3114 | 7394 | | 137.5 | |  |
| Finland | 158834 | | | 250322 | | 57.6 | | 709828 | 1202880 | | 69.5 | | 64950 | 105221 | | 62 | |  |
| France | 1836822 | | | 2936214 | | 59.9 | | 8183649 | 13644230 | | 66.7 | | 727697 | 1219409 | | 67.6 | |  |
| Gabon | 12526 | | | 24097 | | 92.4 | | 57016 | 116015 | | 103.5 | | 5121 | 10252 | | 100.2 | |  |
| Gambia | 6850 | | | 19188 | | 180.1 | | 31839 | 95245 | | 199.1 | | 2801 | 8181 | | 192.1 | |  |
| Georgia | 158184 | | | 139799 | | -11.6 | | 656772 | 609275 | | -7.2 | | 58553 | 52689 | | -10 | |  |
| Germany | 3297312 | | | 4491362 | | 36.2 | | 14447740 | 20782752 | | 43.8 | | 1393916 | 1948900 | | 39.8 | |  |
| Ghana | 128343 | | | 341956 | | 166.4 | | 625638 | 1741716 | | 178.4 | | 54206 | 150818 | | 178.2 | |  |
| Greece | 356475 | | | 486201 | | 36.4 | | 1605448 | 2357764 | | 46.9 | | 145170 | 214521 | | 47.8 | |  |
| Greenland | 748 | | | 1682 | | 124.9 | | 3729 | 9230 | | 147.5 | | 353 | 864 | | 144.6 | |  |
| Grenada | 1177 | | | 2185 | | 85.7 | | 6595 | 12615 | | 91.3 | | 556 | 1060 | | 90.7 | |  |
| Guam | 1574 | | | 4402 | | 179.6 | | 8273 | 24413 | | 195.1 | | 719 | 2077 | | 188.8 | |  |
| Guatemala | 70876 | | | 220834 | | 211.6 | | 364807 | 1210396 | | 231.8 | | 33748 | 110424 | | 227.2 | |  |
| Guinea | 67749 | | | 115345 | | 70.3 | | 304797 | 537126 | | 76.2 | | 27291 | 47796 | | 75.1 | |  |
| Guinea-Bissau | 7964 | | | 14787 | | 85.7 | | 35926 | 69465 | | 93.4 | | 3190 | 6164 | | 93.2 | |  |
| Guyana | 6461 | | | 12064 | | 86.7 | | 35207 | 68885 | | 95.7 | | 2878 | 5587 | | 94.1 | |  |
| Haiti | 55588 | | | 126969 | | 128.4 | | 285597 | 672072 | | 135.3 | | 24598 | 56860 | | 131.2 | |  |
| Honduras | 38226 | | | 123878 | | 224.1 | | 210241 | 709778 | | 237.6 | | 19532 | 67119 | | 243.6 | |  |
| Hungary | 428356 | | | 528451 | | 23.4 | | 1684903 | 2180634 | | 29.4 | | 162221 | 210193 | | 29.6 | |  |
| Iceland | 6710 | | | 13183 | | 96.5 | | 30671 | 63017 | | 105.5 | | 2848 | 5805 | | 103.8 | |  |
| India | 9792377 | | | 24065096 | | 145.8 | | 48442182 | 134411895 | | 177.5 | | 4522334 | 12447634 | | 175.2 | |  |
| Indonesia | 1905403 | | | 4942521 | | 159.4 | | 9215615 | 25764128 | | 179.6 | | 825233 | 2281418 | | 176.5 | |  |
| Iran (Islamic Republic of) | 689622 | | | 1922805 | | 178.8 | | 2819792 | 8736764 | | 209.8 | | 268831 | 818878 | | 204.6 | |  |
| Iraq | 167094 | | | 538163 | | 222.1 | | 734474 | 2547958 | | 246.9 | | 64443 | 225749 | | 250.3 | |  |
| Ireland | 95781 | | | 184901 | | 93 | | 432969 | 869663 | | 100.9 | | 42145 | 83160 | | 97.3 | |  |
| Israel | 114548 | | | 277718 | | 142.4 | | 518924 | 1315095 | | 153.4 | | 48875 | 121842 | | 149.3 | |  |
| Italy | 2186466 | | | 3220799 | | 47.3 | | 9606360 | 14947587 | | 55.6 | | 876682 | 1374605 | | 56.8 | |  |
| Jamaica | 29755 | | | 55658 | | 87.1 | | 167534 | 325467 | | 94.3 | | 14480 | 28033 | | 93.6 | |  |
| Japan | 4617410 | | | 7266090 | | 57.4 | | 21781075 | 38635005 | | 77.4 | | 2148332 | 3720250 | | 73.2 | |  |
| Jordan | 30248 | | | 178767 | | 491 | | 142516 | 895514 | | 528.4 | | 13215 | 82006 | | 520.6 | |  |
| Kazakhstan | 335770 | | | 475368 | | 41.6 | | 1374078 | 2092333 | | 52.3 | | 123579 | 185009 | | 49.7 | |  |
| Kenya | 195901 | | | 559601 | | 185.7 | | 823088 | 2494107 | | 203 | | 75745 | 225274 | | 197.4 | |  |
| Kiribati | 735 | | | 1561 | | 112.4 | | 3703 | 8055 | | 117.5 | | 314 | 685 | | 118.5 | |  |
| Kuwait | 12483 | | | 70474 | | 464.6 | | 61828 | 362235 | | 485.9 | | 5544 | 32816 | | 491.9 | |  |
| Kyrgyzstan | 77111 | | | 125860 | | 63.2 | | 315666 | 543817 | | 72.3 | | 29128 | 50385 | | 73 | |  |
| Lao People's Democratic Republic | 36853 | | | 83394 | | 126.3 | | 178732 | 442347 | | 147.5 | | 16022 | 39030 | | 143.6 | |  |
| Latvia | 99068 | | | 99697 | | 0.6 | | 403003 | 435002 | | 7.9 | | 36910 | 40216 | | 9 | |  |
| Lebanon | 48087 | | | 126507 | | 163.1 | | 218345 | 631273 | | 189.1 | | 19644 | 56443 | | 187.3 | |  |
| Lesotho | 17723 | | | 22664 | | 27.9 | | 80899 | 109579 | | 35.5 | | 7364 | 9598 | | 30.3 | |  |
| Liberia | 22987 | | | 42303 | | 84 | | 106092 | 203438 | | 91.8 | | 9186 | 17350 | | 88.9 | |  |
| Libya | 40574 | | | 120134 | | 196.1 | | 189626 | 589791 | | 211 | | 17109 | 53173 | | 210.8 | |  |
| Lithuania | 125395 | | | 148096 | | 18.1 | | 508318 | 639281 | | 25.8 | | 47157 | 59469 | | 26.1 | |  |
| Luxembourg | 13149 | | | 24795 | | 88.6 | | 58120 | 115610 | | 98.9 | | 5322 | 10531 | | 97.9 | |  |
| Madagascar | 113125 | | | 252505 | | 123.2 | | 478048 | 1106511 | | 131.5 | | 44223 | 101291 | | 129 | |  |
| Malawi | 83033 | | | 159954 | | 92.6 | | 356819 | 713870 | | 100.1 | | 32541 | 64588 | | 98.5 | |  |
| Malaysia | 158559 | | | 508981 | | 221 | | 852797 | 2925230 | | 243 | | 73206 | 249304 | | 240.6 | |  |
| Maldives | 1570 | | | 5792 | | 268.9 | | 8370 | 33537 | | 300.7 | | 714 | 2812 | | 293.9 | |  |
| Mali | 77250 | | | 174966 | | 126.5 | | 357232 | 833296 | | 133.3 | | 30914 | 71853 | | 132.4 | |  |
| Malta | 10406 | | | 21483 | | 106.4 | | 46125 | 100280 | | 117.4 | | 4322 | 9295 | | 115.1 | |  |
| Marshall Islands | 299 | | | 718 | | 139.8 | | 1520 | 3802 | | 150.1 | | 129 | 314 | | 144.3 | |  |
| Mauritania | 19105 | | | 43267 | | 126.5 | | 92016 | 218408 | | 137.4 | | 7978 | 18864 | | 136.4 | |  |
| Mauritius | 13035 | | | 34804 | | 167 | | 69829 | 199905 | | 186.3 | | 6056 | 17226 | | 184.4 | |  |
| Mexico | 805627 | | | 2553695 | | 217 | | 4731465 | 15638516 | | 230.5 | | 435876 | 1447378 | | 232.1 | |  |
| Micronesia (Federated States of) | 928 | | | 1641 | | 76.8 | | 4671 | 8516 | | 82.3 | | 400 | 720 | | 80.1 | |  |
| Monaco | 1487 | | | 2079 | | 39.8 | | 7057 | 10140 | | 43.7 | | 645 | 914 | | 41.7 | |  |
| Mongolia | 25251 | | | 60966 | | 141.4 | | 101001 | 261856 | | 159.3 | | 9185 | 23281 | | 153.5 | |  |
| Montenegro | 18406 | | | 27940 | | 51.8 | | 72015 | 113284 | | 57.3 | | 6794 | 10619 | | 56.3 | |  |
| Morocco | 321519 | | | 824497 | | 156.4 | | 1427944 | 3856702 | | 170.1 | | 133693 | 358805 | | 168.4 | |  |
| Mozambique | 134762 | | | 250728 | | 86.1 | | 557099 | 1085897 | | 94.9 | | 50366 | 97812 | | 94.2 | |  |
| Myanmar | 372290 | | | 843093 | | 126.5 | | 1931190 | 4680998 | | 142.4 | | 165341 | 395394 | | 139.1 | |  |
| Namibia | 13807 | | | 30003 | | 117.3 | | 62094 | 143632 | | 131.3 | | 5665 | 12874 | | 127.2 | |  |
| Nauru | 88 | | | 120 | | 36.1 | | 449 | 625 | | 39.2 | | 38 | 53 | | 38.5 | |  |
| Nepal | 230841 | | | 563848 | | 144.3 | | 1034551 | 2716657 | | 162.6 | | 105455 | 273868 | | 159.7 | |  |
| Netherlands | 423048 | | | 747848 | | 76.8 | | 2084224 | 3687226 | | 76.9 | | 193003 | 331840 | | 71.9 | |  |
| New Zealand | 104211 | | | 219705 | | 110.8 | | 442535 | 987597 | | 123.2 | | 41154 | 90186 | | 119.1 | |  |
| Nicaragua | 28945 | | | 96469 | | 233.3 | | 154950 | 551303 | | 255.8 | | 14152 | 50270 | | 255.2 | |  |
| Niger | 55376 | | | 173700 | | 213.7 | | 245923 | 786106 | | 219.7 | | 21872 | 70335 | | 221.6 | |  |
| Nigeria | 958225 | | | 2091643 | | 118.3 | | 4344178 | 9882646 | | 127.5 | | 385322 | 884413 | | 129.5 | |  |
| Niue | 44 | | | 46 | | 5.9 | | 228 | 253 | | 11 | | 19 | 21 | | 8.9 | |  |
| North Macedonia | 52514 | | | 93081 | | 77.2 | | 201732 | 373839 | | 85.3 | | 18841 | 34450 | | 82.8 | |  |
| Northern Mariana Islands | 334 | | | 1194 | | 257.5 | | 1735 | 6438 | | 271.1 | | 147 | 542 | | 268.7 | |  |
| Norway | 147635 | | | 220564 | | 49.4 | | 659553 | 1021556 | | 54.9 | | 61232 | 91572 | | 49.5 | |  |
| Oman | 13833 | | | 44543 | | 222 | | 61870 | 222863 | | 260.2 | | 5531 | 20008 | | 261.7 | |  |
| Pakistan | 1111919 | | | 2682895 | | 141.3 | | 5243789 | 13118464 | | 150.2 | | 494679 | 1259467 | | 154.6 | |  |
| Palau | 188 | | | 518 | | 176.1 | | 969 | 2768 | | 185.7 | | 81 | 228 | | 180.3 | |  |
| Palestine | 18937 | | | 58578 | | 209.3 | | 85171 | 278743 | | 227.3 | | 7805 | 24931 | | 219.4 | |  |
| Panama | 27049 | | | 84454 | | 212.2 | | 155639 | 511957 | | 228.9 | | 14234 | 46824 | | 229 | |  |
| Papua New Guinea | 34768 | | | 100713 | | 189.7 | | 166553 | 496674 | | 198.2 | | 14342 | 42065 | | 193.3 | |  |
| Paraguay | 42004 | | | 118591 | | 182.3 | | 234851 | 664671 | | 183 | | 20950 | 60977 | | 191.1 | |  |
| Peru | 202120 | | | 595668 | | 194.7 | | 1202397 | 3660705 | | 204.5 | | 103148 | 316446 | | 206.8 | |  |
| Philippines | 552200 | | | 1624438 | | 194.2 | | 2684089 | 8269684 | | 208.1 | | 238525 | 724441 | | 203.7 | |  |
| Poland | 1339426 | | | 2032086 | | 51.7 | | 5041454 | 8030908 | | 59.3 | | 499505 | 761639 | | 52.5 | |  |
| Portugal | 350852 | | | 545013 | | 55.3 | | 1503971 | 2527897 | | 68.1 | | 140203 | 235340 | | 67.9 | |  |
| Puerto Rico | 62671 | | | 115891 | | 84.9 | | 368911 | 721277 | | 95.5 | | 30411 | 58523 | | 92.4 | |  |
| Qatar | 2438 | | | 25307 | | 938 | | 11582 | 128007 | | 1005.2 | | 1017 | 11332 | | 1014.2 | |  |
| Republic of Korea | 747972 | | | 2223401 | | 197.3 | | 3632916 | 12136478 | | 234.1 | | 326185 | 1083017 | | 232 | |  |
| Republic of Moldova | 122746 | | | 159388 | | 29.9 | | 476878 | 667799 | | 40 | | 44020 | 61966 | | 40.8 | |  |
| Romania | 844770 | | | 995443 | | 17.8 | | 3200543 | 3986269 | | 24.5 | | 308417 | 377269 | | 22.3 | |  |
| Russian Federation | 5619255 | | | 6953562 | | 23.7 | | 22388103 | 28560274 | | 27.6 | | 2080475 | 2658762 | | 27.8 | |  |
| Rwanda | 64757 | | | 144787 | | 123.6 | | 270152 | 603317 | | 123.3 | | 25348 | 54580 | | 115.3 | |  |
| Saint Kitts and Nevis | 620 | | | 1372 | | 121.3 | | 3585 | 8111 | | 126.3 | | 303 | 692 | | 128.1 | |  |
| Saint Lucia | 1522 | | | 4477 | | 194.1 | | 8333 | 26156 | | 213.9 | | 713 | 2206 | | 209.3 | |  |
| Saint Vincent and the Grenadines | 1201 | | | 2581 | | 114.9 | | 6700 | 15153 | | 126.2 | | 559 | 1254 | | 124.4 | |  |
| Samoa | 1780 | | | 3044 | | 71 | | 8939 | 16028 | | 79.3 | | 773 | 1345 | | 73.9 | |  |
| San Marino | 805 | | | 1591 | | 97.6 | | 3725 | 7666 | | 105.8 | | 342 | 683 | | 99.8 | |  |
| Sao Tome and Principe | 1286 | | | 2255 | | 75.4 | | 6236 | 11565 | | 85.5 | | 540 | 978 | | 81.1 | |  |
| Saudi Arabia | 118196 | | | 445985 | | 277.3 | | 530188 | 2141952 | | 304 | | 46060 | 187389 | | 306.8 | |  |
| Senegal | 64340 | | | 154923 | | 140.8 | | 300928 | 767903 | | 155.2 | | 26341 | 66158 | | 151.2 | |  |
| Serbia | 340706 | | | 440556 | | 29.3 | | 1298318 | 1788061 | | 37.7 | | 125798 | 169999 | | 35.1 | |  |
| Seychelles | 985 | | | 2170 | | 120.3 | | 5314 | 12686 | | 138.7 | | 457 | 1057 | | 131.5 | |  |
| Sierra Leone | 40962 | | | 75985 | | 85.5 | | 185847 | 362039 | | 94.8 | | 16541 | 31660 | | 91.4 | |  |
| Singapore | 48321 | | | 182173 | | 277 | | 263119 | 1083858 | | 311.9 | | 23375 | 95167 | | 307.1 | |  |
| Slovakia | 169539 | | | 258657 | | 52.6 | | 668634 | 1061971 | | 58.8 | | 63186 | 98465 | | 55.8 | |  |
| Slovenia | 68594 | | | 111424 | | 62.4 | | 271353 | 462737 | | 70.5 | | 25498 | 42516 | | 66.7 | |  |
| Solomon Islands | 2665 | | | 7046 | | 164.4 | | 12839 | 34615 | | 169.6 | | 1087 | 2916 | | 168.3 | |  |
| Somalia | 51465 | | | 126982 | | 146.7 | | 213653 | 537314 | | 151.5 | | 19482 | 48717 | | 150.1 | |  |
| South Africa | 467760 | | | 1044341 | | 123.3 | | 2184126 | 5229919 | | 139.5 | | 195642 | 457627 | | 133.9 | |  |
| South Sudan | 52574 | | | 85710 | | 63 | | 227247 | 370726 | | 63.1 | | 20117 | 32729 | | 62.7 | |  |
| Spain | 1255381 | | | 1940258 | | 54.6 | | 5710224 | 9465301 | | 65.8 | | 537812 | 812142 | | 51 | |  |
| Sri Lanka | 185993 | | | 491766 | | 164.4 | | 955452 | 2682841 | | 180.8 | | 82225 | 224498 | | 173 | |  |
| Sudan | 192545 | | | 425471 | | 121 | | 838884 | 2016232 | | 140.3 | | 77788 | 185818 | | 138.9 | |  |
| Suriname | 4605 | | | 12080 | | 162.3 | | 26074 | 70452 | | 170.2 | | 2184 | 5828 | | 166.9 | |  |
| Sweden | 260286 | | | 402335 | | 54.6 | | 1307835 | 1994393 | | 52.5 | | 115395 | 178855 | | 55 | |  |
| Switzerland | 239630 | | | 405935 | | 69.4 | | 1060113 | 1879275 | | 77.3 | | 98141 | 173538 | | 76.8 | |  |
| Syrian Arab Republic | 114267 | | | 317469 | | 177.8 | | 522477 | 1495495 | | 186.2 | | 48112 | 133949 | | 178.4 | |  |
| Taiwan (Province of China) | 343807 | | | 1013446 | | 194.8 | | 1716556 | 5175253 | | 201.5 | | 153216 | 487705 | | 218.3 | |  |
| Tajikistan | 67119 | | | 147134 | | 119.2 | | 268539 | 612657 | | 128.1 | | 24510 | 55210 | | 125.3 | |  |
| Thailand | 591054 | | | 1932769 | | 227 | | 3394540 | 11547192 | | 240.2 | | 302866 | 1011712 | | 234 | |  |
| Timor-Leste | 4751 | | | 14213 | | 199.2 | | 23544 | 77071 | | 227.3 | | 2059 | 6562 | | 218.8 | |  |
| Togo | 25534 | | | 81972 | | 221 | | 115496 | 390841 | | 238.4 | | 10396 | 34717 | | 234 | |  |
| Tokelau | 27 | | | 30 | | 11.9 | | 136 | 161 | | 18.7 | | 12 | 13 | | 15.8 | |  |
| Tonga | 1160 | | | 1662 | | 43.4 | | 5841 | 8808 | | 50.8 | | 518 | 763 | | 47.4 | |  |
| Trinidad and Tobago | 14546 | | | 35361 | | 143.1 | | 83311 | 212510 | | 155.1 | | 7125 | 17946 | | 151.9 | |  |
| Tunisia | 111242 | | | 303310 | | 172.7 | | 508911 | 1491050 | | 193 | | 46768 | 136104 | | 191 | |  |
| Turkey | 794323 | | | 2176846 | | 174.1 | | 3627261 | 10711008 | | 195.3 | | 346423 | 1004905 | | 190.1 | |  |
| Turkmenistan | 47605 | | | 103546 | | 117.5 | | 194722 | 454398 | | 133.4 | | 17730 | 40563 | | 128.8 | |  |
| Tuvalu | 137 | | | 219 | | 60.2 | | 679 | 1151 | | 69.5 | | 59 | 97 | | 65.2 | |  |
| Uganda | 142119 | | | 327568 | | 130.5 | | 593423 | 1444444 | | 143.4 | | 53874 | 130919 | | 143 | |  |
| Ukraine | 2305791 | | | 2333090 | | 1.2 | | 8775928 | 9280100 | | 5.7 | | 845347 | 882462 | | 4.4 | |  |
| United Arab Emirates | 8360 | | | 115682 | | 1283.8 | | 40485 | 588486 | | 1353.6 | | 3553 | 51415 | | 1347.2 | |  |
| United Kingdom | 1931274 | | | 3018325 | | 56.3 | | 9527163 | 14266557 | | 49.7 | | 893668 | 1345123 | | 50.5 | |  |
| United Republic of Tanzania | 241231 | | | 564551 | | 134 | | 1050915 | 2549782 | | 142.6 | | 94822 | 228535 | | 141 | |  |
| United States Virgin Islands | 1538 | | | 3233 | | 110.2 | | 8821 | 19847 | | 125 | | 741 | 1622 | | 119 | |  |
| United States of America | 7565180 | | | 14523974 | | 92 | | 37966839 | 76833695 | | 102.4 | | 3580394 | 7265242 | | 102.9 | |  |
| Uruguay | 86774 | | | 122680 | | 41.4 | | 455502 | 656057 | | 44 | | 43048 | 63532 | | 47.6 | |  |
| Uzbekistan | 287202 | | | 689004 | | 139.9 | | 1185875 | 2976118 | | 151 | | 108239 | 269714 | | 149.2 | |  |
| Vanuatu | 1221 | | | 3665 | | 200.2 | | 5836 | 18033 | | 209 | | 515 | 1582 | | 207.3 | |  |
| Venezuela (Bolivarian Republic of) | 178250 | | | 577158 | | 223.8 | | 1031082 | 3517290 | | 241.1 | | 94306 | 320306 | | 239.6 | |  |
| Viet Nam | 720180 | | | 1850350 | | 156.9 | | 3458473 | 9631274 | | 178.5 | | 308465 | 851278 | | 176 | |  |
| Yemen | 104386 | | | 299239 | | 186.7 | | 444780 | 1368933 | | 207.8 | | 41466 | 125740 | | 203.2 | |  |
| Zambia | 57203 | | | 145763 | | 154.8 | | 261864 | 670953 | | 156.2 | | 22693 | 58931 | | 159.7 | |  |
| Zimbabwe | 87149 | | | 152423 | | 74.9 | | 392060 | 689621 | | 75.9 | | 35743 | 63622 | | 78 | |  |
| DALYs, disability-adjusted life-years; MSK disorders, musculoskeletal disorders. | | | | | | | | | | | | | | | | | |  |

| **Supplementary Table 5** Global age standardized incidence, prevalence and DALY rates (per 100000 population) and their average annual percentage changes for MSK disorders among adults aged 50 and over by SDI and geographic regions, 1990-2021 | | | | | | | | | | | | | |
| --- | --- | --- | --- | --- | --- | --- | --- | --- | --- | --- | --- | --- | --- |
| Location name | Age standardized incidence rates | | | | Age standardized prevalence rates | | | | Age standardized DALY rates | | | | |
|  | 1990 | 2021 | AAPC (%) | p value | 1990 | 2021 | AAPC (%) | p value | 1990 | 2021 | AAPC (%) | p value |  |
| Global | 10437.01 (10434.87 to 10439.14) | 9869.08 (9867.67 to 9870.50) | -0.181 (-0.190 to -0.172) | <0.001 | 48916.85 (48912.21 to 48921.50) | 50847.95 (50844.76 to 50851.15) | 0.126 (0.118 to 0.134) | <0.001 | 4509.94 (4508.53 to 4511.35) | 4592.19 (4591.23 to 4593.15) | 0.057 (0.042 to 0.072) | <0.001 |  |
| High SDI | 11450.12 (11445.79 to 11454.45) | 11236.60 (11233.41 to 11239.80) | -0.063 (-0.076 to -0.050) | <0.001 | 53721.29 (53711.94 to 53730.63) | 56308.09 (56300.99 to 56315.18) | 0.150 (0.136 to 0.164) | <0.001 | 5091.88 (5089.00 to 5094.76) | 5277.09 (5274.91 to 5279.26) | 0.114 (0.097 to 0.130) | <0.001 |  |
| High-middle SDI | 11242.34 (11237.95 to 11246.73) | 10175.88 (10172.92 to 10178.84) | -0.318 (-0.334 to -0.303) | <0.001 | 49106.98 (49097.77 to 49116.20) | 50026.48 (50019.90 to 50033.07) | 0.062 (0.053 to 0.071) | <0.001 | 4518.40 (4515.62 to 4521.19) | 4436.12 (4434.16 to 4438.08) | -0.054 (-0.066 to -0.043) | <0.001 |  |
| Middle SDI | 9395.38 (9391.42 to 9399.34) | 9026.78 (9024.40 to 9029.15) | -0.126 (-0.134 to -0.118) | <0.001 | 46414.58 (46405.76 to 46423.40) | 49220.12 (49214.59 to 49225.65) | 0.192 (0.184 to 0.200) | <0.001 | 4154.30 (4151.67 to 4156.93) | 4305.61 (4303.98 to 4307.24) | 0.115 (0.110 to 0.121) | <0.001 |  |
| Low-middle SDI | 9504.60 (9499.40 to 9509.79) | 9423.80 (9420.45 to 9427.15) | -0.029 (-0.061 to 0.003) | 0.072 | 45918.80 (45907.36 to 45930.25) | 49298.74 (49291.04 to 49306.44) | 0.228 (0.218 to 0.240) | <0.001 | 4260.12 (4256.65 to 4263.59) | 4577.68 (4575.35 to 4580.01) | 0.230 (0.204 to 0.256) | <0.001 |  |
| Low SDI | 9795.85 (9787.19 to 9804.51) | 9555.02 (9549.29 to 9560.74) | -0.080 (-0.093 to -0.067) | <0.001 | 44171.03 (44152.55 to 44189.51) | 46091.10 (46078.48 to 46103.72) | 0.137 (0.132 to 0.141) | <0.001 | 4029.16 (4023.61 to 4034.71) | 4178.73 (4174.94 to 4182.51) | 0.117 (0.106 to 0.129) | <0.001 |  |
| Central Asia | 11232.05 (11212.00 to 11252.10) | 11295.51 (11280.36 to 11310.66) | 0.018 (0.016 to 0.020) | <0.001 | 46613.40 (46572.28 to 46654.52) | 49505.35 (49473.46 to 49537.24) | 0.196 (0.186 to 0.206) | <0.001 | 4208.17 (4195.84 to 4220.50) | 4430.77 (4421.24 to 4440.29) | 0.169 (0.144 to 0.193) | <0.001 |  |
| Central Europe | 13215.23 (13202.92 to 13227.54) | 13217.01 (13206.46 to 13227.55) | 0.000 (-0.006 to 0.005) | 0.879 | 50837.14 (50812.97 to 50861.30) | 52617.89 (52596.98 to 52638.80) | 0.111 (0.108 to 0.114) | <0.001 | 4892.78 (4885.29 to 4900.27) | 4995.48 (4989.01 to 5001.95) | 0.066 (0.054 to 0.078) | <0.001 |  |
| Eastern Europe | 13519.25 (13510.27 to 13528.23) | 13391.27 (13383.06 to 13399.48) | -0.031 (-0.038 to -0.025) | <0.001 | 53680.16 (53662.19 to 53698.13) | 54452.43 (54435.91 to 54468.96) | 0.048 (0.039 to 0.058) | <0.001 | 5019.82 (5014.33 to 5025.31) | 5088.94 (5083.88 to 5093.99) | 0.049 (0.022 to 0.076) | <0.001 |  |
| Australasia | 11948.26 (11917.82 to 11978.70) | 11838.06 (11817.61 to 11858.50) | -0.032 (-0.043 to -0.021) | <0.001 | 55484.53 (55419.14 to 55549.92) | 58254.97 (58209.93 to 58300.01) | 0.160 (0.150 to 0.171) | <0.001 | 5384.45 (5364.09 to 5404.82) | 5562.59 (5548.63 to 5576.54) | 0.105 (0.077 to 0.134) | <0.001 |  |
| High-income Asia Pacific | 11945.39 (11935.34 to 11955.45) | 11239.04 (11231.97 to 11246.12) | -0.227 (-0.265 to -0.188) | <0.001 | 57005.64 (56983.59 to 57027.69) | 58643.79 (58627.83 to 58659.74) | 0.083 (0.051 to 0.116) | <0.001 | 5527.69 (5520.83 to 5534.55) | 5587.04 (5582.10 to 5591.98) | 0.021 (-0.019 to 0.060) | 0.309 |  |
| High-income North America | 11607.11 (11599.19 to 11615.03) | 11610.57 (11604.86 to 11616.27) | 0.000 (-0.015 to 0.015) | 0.987 | 57603.37 (57585.85 to 57620.89) | 61332.49 (61319.46 to 61345.53) | 0.205 (0.167 to 0.242) | <0.001 | 5486.24 (5480.81 to 5491.67) | 5821.42 (5817.40 to 5825.44) | 0.193 (0.174 to 0.212) | <0.001 |  |
| Southern Latin America | 10646.92 (10626.85 to 10666.99) | 10897.63 (10882.64 to 10912.62) | 0.053 (-0.089 to 0.194) | 0.464 | 56026.48 (55980.40 to 56072.56) | 58638.19 (58603.48 to 58672.90) | 0.148 (0.126 to 0.171) | <0.001 | 5454.02 (5439.65 to 5468.39) | 5708.23 (5697.39 to 5719.06) | 0.146 (0.084 to 0.207) | <0.001 |  |
| Western Europe | 11125.46 (11119.54 to 11131.38) | 11124.78 (11119.96 to 11129.60) | 0.000 (-0.011 to 0.010) | 0.973 | 50065.72 (50053.19 to 50078.24) | 51543.43 (51533.12 to 51553.73) | 0.094 (0.088 to 0.100) | <0.001 | 4681.05 (4677.21 to 4684.90) | 4768.77 (4765.62 to 4771.93) | 0.061 (0.043 to 0.079) | <0.001 |  |
| Andean Latin America | 7813.77 (7787.84 to 7839.70) | 8091.69 (8076.22 to 8107.15) | 0.109 (0.093 to 0.125) | <0.001 | 46267.12 (46203.70 to 46330.55) | 49624.98 (49586.61 to 49663.36) | 0.226 (0.219 to 0.232) | <0.001 | 3970.22 (3951.67 to 3988.76) | 4270.48 (4259.23 to 4281.73) | 0.235 (0.204 to 0.266) | <0.001 |  |
| Caribbean | 7914.96 (7891.70 to 7938.23) | 8045.31 (8029.26 to 8061.36) | 0.052 (0.048 to 0.056) | <0.001 | 43887.25 (43832.37 to 43942.13) | 46979.74 (46940.89 to 47018.58) | 0.218 (0.203 to 0.232) | <0.001 | 3667.78 (3651.93 to 3683.64) | 3904.61 (3893.42 to 3915.80) | 0.200 (0.178 to 0.223) | <0.001 |  |
| Central Latin America | 8659.06 (8645.55 to 8672.56) | 8876.87 (8869.07 to 8884.67) | 0.081 (0.075 to 0.087) | <0.001 | 49856.91 (49824.36 to 49889.47) | 53691.39 (53672.18 to 53710.60) | 0.239 (0.234 to 0.244) | <0.001 | 4563.30 (4553.48 to 4573.12) | 4949.48 (4943.66 to 4955.30) | 0.264 (0.250 to 0.278) | <0.001 |  |
| Tropical Latin America | 10174.27 (10160.39 to 10188.15) | 10697.65 (10689.18 to 10706.12) | 0.163 (0.147 to 0.179) | <0.001 | 52625.30 (52593.59 to 52657.01) | 54800.73 (54781.54 to 54819.92) | 0.133 (0.119 to 0.147) | <0.001 | 4892.20 (4882.58 to 4901.82) | 5093.37 (5087.53 to 5099.21) | 0.134 (0.100 to 0.168) | <0.001 |  |
| North Africa and Middle East | 10192.91 (10182.67 to 10203.14) | 10296.82 (10290.60 to 10303.03) | 0.034 (0.026 to 0.043) | <0.001 | 45242.98 (45221.38 to 45264.58) | 49244.74 (49231.13 to 49258.34) | 0.274 (0.261 to 0.287) | <0.001 | 4173.23 (4166.70 to 4179.76) | 4500.87 (4496.77 to 4504.97) | 0.243 (0.238 to 0.249) | <0.001 |  |
| South Asia | 9545.39 (9540.03 to 9550.74) | 9243.37 (9240.08 to 9246.67) | -0.102 (-0.131 to -0.073) | <0.001 | 46996.88 (46984.97 to 47008.80) | 50766.43 (50758.72 to 50774.13) | 0.248 (0.228 to 0.268) | <0.001 | 4400.84 (4397.22 to 4404.47) | 4739.82 (4737.47 to 4742.17) | 0.240 (0.207 to 0.274) | <0.001 |  |
| East Asia | 9535.88 (9531.55 to 9540.21) | 8750.72 (8748.16 to 8753.29) | -0.272 (-0.307 to -0.237) | <0.001 | 45618.16 (45608.65 to 45627.66) | 47675.80 (47669.82 to 47681.77) | 0.149 (0.131 to 0.167) | <0.001 | 4042.77 (4039.95 to 4045.59) | 4018.08 (4016.36 to 4019.81) | -0.012 (-0.036 to 0.011) | 0.308 |  |
| Oceania | 8785.20 (8713.39 to 8857.02) | 8832.96 (8788.47 to 8877.45) | 0.017 (0.008 to 0.025) | <0.001 | 43297.83 (43137.55 to 43458.12) | 45059.16 (44957.98 to 45160.33) | 0.128 (0.121 to 0.136) | <0.001 | 3675.10 (3628.72 to 3721.47) | 3758.29 (3729.26 to 3787.31) | 0.071 (0.066 to 0.077) | <0.001 |  |
| Southeast Asia | 8228.50 (8221.01 to 8235.98) | 8343.03 (8338.42 to 8347.64) | 0.045 (0.036 to 0.053) | <0.001 | 41158.38 (41141.65 to 41175.12) | 44755.97 (44745.29 to 44766.65) | 0.272 (0.268 to 0.276) | <0.001 | 3619.06 (3614.12 to 3624.00) | 3892.58 (3889.45 to 3895.72) | 0.236 (0.232 to 0.240) | <0.001 |  |
| Central Sub-Saharan Africa | 10158.33 (10130.71 to 10185.94) | 10013.52 (9996.04 to 10031.01) | -0.046 (-0.061 to -0.031) | <0.001 | 45197.64 (45138.78 to 45256.50) | 46176.01 (46138.05 to 46213.98) | 0.069 (0.063 to 0.075) | <0.001 | 4034.25 (4016.79 to 4051.72) | 4104.26 (4093.01 to 4115.51) | 0.054 (0.047 to 0.060) | <0.001 |  |
| Eastern Sub-Saharan Africa | 10258.06 (10242.55 to 10273.56) | 10174.90 (10164.63 to 10185.17) | -0.026 (-0.030 to -0.021) | <0.001 | 43643.15 (43610.95 to 43675.36) | 45813.28 (45791.32 to 45835.23) | 0.158 (0.152 to 0.164) | <0.001 | 3929.04 (3919.42 to 3938.66) | 4058.85 (4052.35 to 4065.36) | 0.108 (0.103 to 0.113) | <0.001 |  |
| Southern Sub-Saharan Africa | 10234.32 (10208.51 to 10260.13) | 10016.67 (9999.40 to 10033.94) | -0.068 (-0.074 to -0.062) | <0.001 | 48030.13 (47973.88 to 48086.38) | 50187.65 (50148.72 to 50226.57) | 0.144 (0.134 to 0.155) | <0.001 | 4296.73 (4279.95 to 4313.51) | 4400.71 (4389.21 to 4412.22) | 0.080 (0.053 to 0.108) | <0.001 |  |
| Western Sub-Saharan Africa | 9579.12 (9565.34 to 9592.90) | 9549.24 (9540.18 to 9558.29) | -0.012 (-0.021 to -0.003) | 0.01 | 44174.56 (44144.78 to 44204.33) | 46269.96 (46249.85 to 46290.07) | 0.150 (0.144 to 0.156) | <0.001 | 3879.33 (3870.55 to 3888.11) | 4053.57 (4047.65 to 4059.49) | 0.140 (0.132 to 0.148) | <0.001 |  |
| AAPC, average annual percentage changes; DALYs, disability-adjusted life-years; MSK disorders, musculoskeletal disorders; SDI, Socio-demographic Index. | | | | | | | | | | | | | |

| **Supplementary Table 6** Age standardized incidence, prevalence and DALY rates (per 100000 population) and their average annual percentage changes for MSK disorders among adults aged 50 and over across 204 countries and territories, 1990-2021 | | | | | | | | | | | | |
| --- | --- | --- | --- | --- | --- | --- | --- | --- | --- | --- | --- | --- |
| Location name | Age standardized incidence rates | | | | Age standardized prevalence rates | | | | Age standardized DALY rates | | | |
|  | 1990 | 2021 | AAPC (%) | p value | 1990 | 2021 | AAPC (%) | p value | 1990 | 2021 | AAPC (%) | p value |
| Afghanistan | 9574.14 (9526.12 to 9622.16) | 9978.81 (9936.04 to 10021.58) | 0.133 (0.125 to 0.140) | <0.001 | 39632.94 (39535.02 to 39730.86) | 43608.20 (43518.48 to 43697.93) | 0.307 (0.290 to 0.325) | <0.001 | 3544.40 (3515.29 to 3573.51) | 3901.48 (3874.91 to 3928.06) | 0.310 (0.295 to 0.325) | <0.001 |
| Albania | 12613.14 (12511.55 to 12714.73) | 12793.40 (12722.39 to 12864.41) | 0.046 (0.044 to 0.048) | <0.001 | 46535.64 (46339.03 to 46732.24) | 49520.88 (49381.25 to 49660.51) | 0.203 (0.197 to 0.208) | <0.001 | 4549.55 (4488.28 to 4610.82) | 4806.98 (4763.45 to 4850.51) | 0.184 (0.173 to 0.194) | <0.001 |
| Algeria | 9814.71 (9777.79 to 9851.64) | 9984.26 (9962.49 to 10006.04) | 0.055 (0.046 to 0.064) | <0.001 | 44686.50 (44607.46 to 44765.55) | 49368.35 (49319.90 to 49416.80) | 0.322 (0.318 to 0.326) | <0.001 | 4057.74 (4034.04 to 4081.43) | 4461.91 (4447.38 to 4476.43) | 0.306 (0.299 to 0.313) | <0.001 |
| American Samoa | 9273.64 (8430.35 to 10116.93) | 9338.69 (8777.48 to 9899.89) | 0.021 (0.014 to 0.028) | <0.001 | 49012.81 (47061.96 to 50963.65) | 50893.69 (49576.64 to 52210.73) | 0.123 (0.114 to 0.131) | <0.001 | 4140.58 (3575.82 to 4705.33) | 4207.06 (3829.59 to 4584.54) | 0.058 (0.035 to 0.081) | <0.001 |
| Andorra | 10827.08 (10251.25 to 11402.91) | 10728.79 (10380.85 to 11076.73) | -0.029 (-0.050 to -0.009) | 0.005 | 48556.51 (47334.92 to 49778.10) | 49510.54 (48763.96 to 50257.12) | 0.062 (0.053 to 0.070) | <0.001 | 4332.17 (3967.89 to 4696.46) | 4323.30 (4102.49 to 4544.12) | -0.007 (-0.025 to 0.011) | 0.422 |
| Angola | 10228.47 (10161.52 to 10295.42) | 10121.61 (10083.74 to 10159.48) | -0.034 (-0.044 to -0.025) | <0.001 | 45989.15 (45845.46 to 46132.83) | 48343.04 (48259.19 to 48426.89) | 0.162 (0.155 to 0.169) | <0.001 | 4057.75 (4015.40 to 4100.11) | 4225.86 (4201.22 to 4250.49) | 0.132 (0.124 to 0.140) | <0.001 |
| Antigua and Barbuda | 7953.85 (7421.28 to 8486.42) | 8048.39 (7696.43 to 8400.35) | 0.037 (0.030 to 0.044) | <0.001 | 45808.83 (44540.18 to 47077.48) | 48231.01 (47364.42 to 49097.61) | 0.167 (0.159 to 0.175) | <0.001 | 3831.04 (3463.17 to 4198.91) | 4048.40 (3797.63 to 4299.16) | 0.175 (0.151 to 0.200) | <0.001 |
| Argentina | 10662.32 (10638.31 to 10686.33) | 10839.02 (10820.26 to 10857.78) | 0.034 (-0.100 to 0.167) | 0.622 | 55596.19 (55541.33 to 55651.05) | 58664.94 (58621.43 to 58708.45) | 0.170 (0.155 to 0.186) | <0.001 | 5367.36 (5350.32 to 5384.39) | 5710.11 (5696.52 to 5723.69) | 0.188 (0.102 to 0.274) | <0.001 |
| Armenia | 11405.74 (11323.59 to 11487.88) | 11687.93 (11619.27 to 11756.59) | 0.078 (0.070 to 0.086) | <0.001 | 45194.91 (45030.19 to 45359.63) | 50175.82 (50034.17 to 50317.46) | 0.341 (0.331 to 0.351) | <0.001 | 4103.40 (4053.95 to 4152.85) | 4566.15 (4523.37 to 4608.94) | 0.351 (0.325 to 0.377) | <0.001 |
| Australia | 11806.90 (11773.78 to 11840.02) | 11724.24 (11702.01 to 11746.47) | -0.024 (-0.039 to -0.010) | <0.001 | 55907.37 (55835.47 to 55979.26) | 58872.24 (58822.77 to 58921.71) | 0.167 (0.146 to 0.188) | <0.001 | 5468.64 (5446.17 to 5491.10) | 5663.26 (5647.88 to 5678.65) | 0.106 (0.062 to 0.150) | <0.001 |
| Austria | 10726.64 (10685.52 to 10767.76) | 10367.23 (10334.40 to 10400.06) | -0.102 (-0.123 to -0.081) | <0.001 | 48568.01 (48480.95 to 48655.07) | 49881.97 (49810.33 to 49953.61) | 0.087 (0.069 to 0.105) | <0.001 | 4455.96 (4429.50 to 4482.42) | 4488.91 (4467.31 to 4510.51) | 0.025 (-0.005 to 0.055) | 0.101 |
| Azerbaijan | 11142.71 (11082.69 to 11202.72) | 11337.94 (11296.10 to 11379.79) | 0.058 (0.050 to 0.066) | <0.001 | 47255.11 (47130.26 to 47379.96) | 49846.79 (49758.41 to 49935.17) | 0.173 (0.156 to 0.190) | <0.001 | 4251.03 (4213.67 to 4288.38) | 4448.24 (4421.88 to 4474.60) | 0.155 (0.134 to 0.176) | <0.001 |
| Bahamas | 8056.13 (7751.88 to 8360.38) | 8133.41 (7953.04 to 8313.79) | 0.030 (0.024 to 0.036) | <0.001 | 47211.47 (46470.14 to 47952.80) | 49170.99 (48724.13 to 49617.85) | 0.132 (0.124 to 0.141) | <0.001 | 4050.96 (3834.25 to 4267.68) | 4195.49 (4065.22 to 4325.75) | 0.125 (0.088 to 0.161) | <0.001 |
| Bahrain | 9835.33 (9525.18 to 10145.48) | 9882.38 (9755.21 to 10009.54) | 0.018 (0.008 to 0.028) | <0.001 | 47832.50 (47146.15 to 48518.86) | 50519.36 (50231.61 to 50807.11) | 0.176 (0.167 to 0.184) | <0.001 | 4333.11 (4127.50 to 4538.71) | 4591.45 (4504.84 to 4678.07) | 0.188 (0.168 to 0.208) | <0.001 |
| Bangladesh | 10321.47 (10301.67 to 10341.27) | 10125.53 (10114.28 to 10136.78) | -0.063 (-0.076 to -0.051) | <0.001 | 50625.10 (50581.12 to 50669.08) | 53812.32 (53786.35 to 53838.29) | 0.199 (0.195 to 0.203) | <0.001 | 5135.95 (5121.96 to 5149.95) | 5404.90 (5396.67 to 5413.13) | 0.169 (0.154 to 0.183) | <0.001 |
| Barbados | 8149.22 (7916.26 to 8382.19) | 8185.62 (8018.80 to 8352.43) | 0.015 (0.011 to 0.020) | <0.001 | 47337.54 (46781.29 to 47893.79) | 49426.23 (49017.70 to 49834.75) | 0.139 (0.135 to 0.142) | <0.001 | 4128.35 (3963.43 to 4293.26) | 4255.39 (4135.36 to 4375.42) | 0.100 (0.065 to 0.136) | <0.001 |
| Belarus | 12386.43 (12346.46 to 12426.39) | 12502.66 (12465.73 to 12539.59) | 0.030 (0.024 to 0.035) | <0.001 | 50384.29 (50303.54 to 50465.04) | 52876.64 (52800.94 to 52952.33) | 0.156 (0.151 to 0.160) | <0.001 | 4607.33 (4582.91 to 4631.76) | 4786.24 (4763.45 to 4809.04) | 0.123 (0.114 to 0.133) | <0.001 |
| Belgium | 11100.63 (11064.47 to 11136.79) | 10952.32 (10922.18 to 10982.47) | -0.049 (-0.069 to -0.029) | <0.001 | 49169.09 (49093.24 to 49244.95) | 50012.72 (49948.81 to 50076.64) | 0.050 (0.042 to 0.058) | <0.001 | 4530.59 (4507.52 to 4553.66) | 4498.44 (4479.15 to 4517.72) | -0.020 (-0.062 to 0.022) | 0.343 |
| Belize | 7915.25 (7524.52 to 8305.98) | 8141.07 (7926.67 to 8355.48) | 0.089 (0.081 to 0.097) | <0.001 | 43991.63 (43070.00 to 44913.26) | 47444.51 (46922.48 to 47966.54) | 0.245 (0.234 to 0.256) | <0.001 | 3685.05 (3418.35 to 3951.74) | 3947.27 (3797.25 to 4097.29) | 0.225 (0.191 to 0.259) | <0.001 |
| Benin | 9514.54 (9421.61 to 9607.46) | 9452.64 (9396.27 to 9509.01) | -0.022 (-0.031 to -0.014) | <0.001 | 43578.51 (43379.04 to 43777.98) | 46291.53 (46165.84 to 46417.23) | 0.195 (0.192 to 0.198) | <0.001 | 3838.81 (3779.78 to 3897.85) | 4010.04 (3973.22 to 4046.87) | 0.139 (0.128 to 0.151) | <0.001 |
| Bermuda | 8279.66 (7797.01 to 8762.31) | 8290.97 (7956.95 to 8624.99) | -0.001 (-0.020 to 0.018) | 0.92 | 48475.02 (47304.82 to 49645.22) | 50294.17 (49476.93 to 51111.41) | 0.119 (0.116 to 0.122) | <0.001 | 4143.43 (3801.16 to 4485.71) | 4260.89 (4022.62 to 4499.17) | 0.088 (0.065 to 0.112) | <0.001 |
| Bhutan | 10059.52 (9792.53 to 10326.51) | 10082.81 (9911.11 to 10254.51) | 0.011 (-0.010 to 0.032) | 0.306 | 47682.91 (47098.89 to 48266.93) | 51019.58 (50633.01 to 51406.15) | 0.219 (0.212 to 0.227) | <0.001 | 4715.87 (4532.33 to 4899.40) | 5021.64 (4900.43 to 5142.85) | 0.205 (0.194 to 0.217) | <0.001 |
| Bolivia (Plurinational State of) | 7973.21 (7906.98 to 8039.44) | 8275.94 (8236.49 to 8315.40) | 0.118 (0.109 to 0.126) | <0.001 | 45105.12 (44946.50 to 45263.75) | 48885.61 (48789.24 to 48981.98) | 0.261 (0.255 to 0.266) | <0.001 | 3931.13 (3884.36 to 3977.89) | 4257.43 (4229.03 to 4285.83) | 0.257 (0.243 to 0.270) | <0.001 |
| Bosnia and Herzegovina | 12477.64 (12407.96 to 12547.32) | 12651.42 (12591.19 to 12711.65) | 0.043 (0.032 to 0.053) | <0.001 | 47303.07 (47166.08 to 47440.05) | 50721.92 (50601.76 to 50842.09) | 0.230 (0.215 to 0.245) | <0.001 | 4513.22 (4471.12 to 4555.32) | 4769.63 (4732.73 to 4806.54) | 0.185 (0.171 to 0.200) | <0.001 |
| Botswana | 9473.15 (9301.71 to 9644.59) | 9692.08 (9585.51 to 9798.65) | 0.073 (0.069 to 0.077) | <0.001 | 45017.33 (44640.73 to 45393.92) | 48915.14 (48673.71 to 49156.58) | 0.269 (0.258 to 0.280) | <0.001 | 4017.91 (3905.90 to 4129.93) | 4223.07 (4152.30 to 4293.85) | 0.162 (0.139 to 0.185) | <0.001 |
| Brazil | 10209.65 (10195.58 to 10223.73) | 10733.15 (10724.59 to 10741.72) | 0.163 (0.149 to 0.177) | <0.001 | 52707.95 (52675.83 to 52740.08) | 54874.46 (54855.03 to 54893.88) | 0.132 (0.118 to 0.147) | <0.001 | 4904.74 (4894.98 to 4914.50) | 5102.02 (5096.10 to 5107.94) | 0.131 (0.096 to 0.167) | <0.001 |
| Brunei Darussalam | 10237.28 (9813.74 to 10660.82) | 10251.56 (10034.80 to 10468.32) | -0.005 (-0.020 to 0.010) | 0.525 | 53114.61 (52138.51 to 54090.71) | 56646.00 (56128.86 to 57163.15) | 0.204 (0.189 to 0.220) | <0.001 | 4767.67 (4476.26 to 5059.08) | 5093.50 (4939.13 to 5247.87) | 0.206 (0.174 to 0.238) | <0.001 |
| Bulgaria | 12744.37 (12702.64 to 12786.10) | 12699.69 (12658.98 to 12740.40) | -0.011 (-0.014 to -0.007) | <0.001 | 49858.63 (49775.86 to 49941.40) | 51658.04 (51576.43 to 51739.66) | 0.115 (0.111 to 0.120) | <0.001 | 4657.01 (4631.75 to 4682.28) | 4779.87 (4755.00 to 4804.75) | 0.089 (0.078 to 0.100) | <0.001 |
| Burkina Faso | 9430.26 (9368.92 to 9491.61) | 9285.92 (9244.14 to 9327.71) | -0.050 (-0.059 to -0.041) | <0.001 | 42060.64 (41930.38 to 42190.90) | 43451.44 (43360.56 to 43542.33) | 0.104 (0.099 to 0.109) | <0.001 | 3746.99 (3708.32 to 3785.66) | 3847.99 (3821.07 to 3874.90) | 0.085 (0.065 to 0.105) | <0.001 |
| Burundi | 10169.08 (10080.65 to 10257.52) | 9788.84 (9729.88 to 9847.79) | -0.121 (-0.126 to -0.115) | <0.001 | 43512.86 (43329.01 to 43696.71) | 41632.78 (41510.32 to 41755.24) | -0.143 (-0.168 to -0.117) | <0.001 | 3979.62 (3924.21 to 4035.03) | 3675.74 (3639.54 to 3711.95) | -0.254 (-0.282 to -0.227) | <0.001 |
| Cabo Verde | 9288.86 (9020.28 to 9557.44) | 9190.80 (9003.52 to 9378.07) | -0.034 (-0.048 to -0.020) | <0.001 | 43427.50 (42848.09 to 44006.92) | 46477.51 (46054.46 to 46900.56) | 0.220 (0.217 to 0.223) | <0.001 | 3852.63 (3679.78 to 4025.48) | 3994.53 (3870.72 to 4118.35) | 0.116 (0.104 to 0.128) | <0.001 |
| Cambodia | 8490.13 (8432.54 to 8547.71) | 8328.75 (8295.06 to 8362.45) | -0.062 (-0.070 to -0.053) | <0.001 | 39663.32 (39539.00 to 39787.64) | 42145.55 (42069.96 to 42221.15) | 0.197 (0.192 to 0.203) | <0.001 | 3556.46 (3519.43 to 3593.48) | 3705.40 (3683.10 to 3727.71) | 0.134 (0.126 to 0.143) | <0.001 |
| Cameroon | 9768.88 (9707.81 to 9829.95) | 9616.98 (9580.78 to 9653.19) | -0.049 (-0.067 to -0.031) | <0.001 | 45119.57 (44987.33 to 45251.81) | 47065.61 (46984.84 to 47146.39) | 0.137 (0.134 to 0.140) | <0.001 | 4015.64 (3976.40 to 4054.88) | 4122.75 (4098.97 to 4146.52) | 0.083 (0.074 to 0.093) | <0.001 |
| Canada | 10000.59 (9976.88 to 10024.31) | 9639.91 (9624.13 to 9655.69) | -0.114 (-0.126 to -0.101) | <0.001 | 52201.33 (52147.23 to 52255.42) | 54491.93 (54454.52 to 54529.35) | 0.137 (0.125 to 0.148) | <0.001 | 5091.59 (5074.69 to 5108.48) | 5184.76 (5173.18 to 5196.34) | 0.057 (0.036 to 0.078) | <0.001 |
| Central African Republic | 10033.06 (9910.78 to 10155.35) | 9951.03 (9864.44 to 10037.62) | -0.028 (-0.036 to -0.019) | <0.001 | 43848.38 (43589.80 to 44106.97) | 44455.99 (44270.39 to 44641.58) | 0.044 (0.042 to 0.046) | <0.001 | 3933.24 (3856.39 to 4010.09) | 3970.77 (3915.75 to 4025.79) | 0.026 (0.018 to 0.034) | <0.001 |
| Chad | 9916.64 (9837.44 to 9995.84) | 9553.36 (9499.95 to 9606.77) | -0.122 (-0.132 to -0.111) | <0.001 | 43436.38 (43270.07 to 43602.69) | 43298.27 (43183.65 to 43412.89) | -0.011 (-0.021 to -0.002) | 0.018 | 3952.23 (3902.23 to 4002.23) | 3853.23 (3819.24 to 3887.21) | -0.084 (-0.094 to -0.075) | <0.001 |
| Chile | 10729.50 (10686.22 to 10772.77) | 10997.69 (10970.35 to 11025.03) | 0.065 (0.046 to 0.085) | <0.001 | 58181.68 (58080.72 to 58282.64) | 58668.92 (58605.85 to 58731.99) | 0.025 (-0.006 to 0.056) | 0.11 | 5865.30 (5833.29 to 5897.30) | 5711.47 (5691.77 to 5731.17) | -0.079 (-0.148 to -0.009) | 0.027 |
| China | 9527.91 (9523.50 to 9532.32) | 8699.10 (8696.51 to 8701.69) | -0.288 (-0.321 to -0.256) | <0.001 | 45540.62 (45530.94 to 45550.30) | 47524.40 (47518.32 to 47530.47) | 0.144 (0.127 to 0.162) | <0.001 | 4032.20 (4029.34 to 4035.06) | 3989.46 (3987.70 to 3991.23) | -0.027 (-0.050 to -0.004) | 0.022 |
| Colombia | 8859.83 (8830.19 to 8889.46) | 8985.83 (8969.08 to 9002.59) | 0.047 (0.038 to 0.055) | <0.001 | 48973.47 (48903.42 to 49043.52) | 53129.10 (53088.35 to 53169.85) | 0.267 (0.255 to 0.278) | <0.001 | 4503.19 (4482.02 to 4524.35) | 4957.96 (4945.51 to 4970.41) | 0.315 (0.272 to 0.358) | <0.001 |
| Comoros | 9527.92 (9238.36 to 9817.47) | 9754.45 (9569.62 to 9939.27) | 0.081 (0.061 to 0.102) | <0.001 | 42447.89 (41833.01 to 43062.76) | 45255.59 (44855.22 to 45655.96) | 0.208 (0.201 to 0.214) | <0.001 | 3763.30 (3581.04 to 3945.57) | 4008.18 (3889.41 to 4126.96) | 0.206 (0.194 to 0.218) | <0.001 |
| Congo | 9897.77 (9771.58 to 10023.95) | 9883.90 (9805.72 to 9962.09) | -0.004 (-0.009 to 0.000) | 0.069 | 45890.22 (45615.98 to 46164.46) | 47753.21 (47579.28 to 47927.14) | 0.126 (0.117 to 0.135) | <0.001 | 4096.82 (4015.31 to 4178.34) | 4185.96 (4134.74 to 4237.17) | 0.069 (0.051 to 0.087) | <0.001 |
| Cook Islands | 9065.64 (7949.02 to 10182.25) | 9388.11 (8606.52 to 10169.70) | 0.116 (0.085 to 0.148) | <0.001 | 47727.75 (45155.86 to 50299.65) | 51351.98 (49524.38 to 53179.58) | 0.238 (0.219 to 0.257) | <0.001 | 3997.23 (3255.31 to 4739.15) | 4254.00 (3728.23 to 4779.77) | 0.207 (0.179 to 0.235) | <0.001 |
| Costa Rica | 8710.69 (8616.22 to 8805.17) | 8806.71 (8754.14 to 8859.28) | 0.036 (0.033 to 0.040) | <0.001 | 49750.45 (49524.29 to 49976.61) | 53119.99 (52990.87 to 53249.12) | 0.214 (0.206 to 0.221) | <0.001 | 4583.00 (4514.46 to 4651.54) | 4917.34 (4878.06 to 4956.62) | 0.228 (0.210 to 0.247) | <0.001 |
| Croatia | 12326.95 (12269.17 to 12384.73) | 12596.30 (12544.69 to 12647.90) | 0.074 (0.059 to 0.089) | <0.001 | 48693.51 (48578.30 to 48808.72) | 51139.47 (51036.17 to 51242.76) | 0.156 (0.129 to 0.184) | <0.001 | 4525.70 (4490.68 to 4560.73) | 4783.13 (4751.42 to 4814.84) | 0.177 (0.128 to 0.226) | <0.001 |
| Cuba | 7933.13 (7895.91 to 7970.35) | 7972.98 (7946.62 to 7999.34) | 0.013 (-0.008 to 0.035) | 0.22 | 43176.52 (43089.68 to 43263.37) | 47020.40 (46956.33 to 47084.47) | 0.278 (0.260 to 0.295) | <0.001 | 3571.19 (3546.20 to 3596.18) | 3899.15 (3880.68 to 3917.61) | 0.278 (0.245 to 0.311) | <0.001 |
| Cyprus | 10925.67 (10772.82 to 11078.51) | 10948.86 (10851.00 to 11046.72) | 0.009 (0.003 to 0.014) | 0.002 | 47895.48 (47574.45 to 48216.51) | 50806.90 (50596.55 to 51017.24) | 0.190 (0.182 to 0.198) | <0.001 | 4618.44 (4518.44 to 4718.44) | 4781.52 (4716.84 to 4846.20) | 0.113 (0.094 to 0.132) | <0.001 |
| Czechia | 13226.77 (13185.04 to 13268.50) | 13090.12 (13055.90 to 13124.34) | -0.035 (-0.041 to -0.029) | <0.001 | 51922.66 (51840.21 to 52005.12) | 53253.34 (53184.76 to 53321.92) | 0.081 (0.076 to 0.085) | <0.001 | 4894.23 (4868.88 to 4919.57) | 5010.99 (4989.88 to 5032.10) | 0.076 (0.067 to 0.086) | <0.001 |
| C么te d'Ivoire | 9535.00 (9471.28 to 9598.72) | 9474.66 (9436.63 to 9512.68) | -0.020 (-0.031 to -0.008) | <0.001 | 43756.42 (43618.40 to 43894.44) | 45752.86 (45668.54 to 45837.18) | 0.144 (0.139 to 0.149) | <0.001 | 3806.72 (3766.36 to 3847.08) | 3997.91 (3973.13 to 4022.68) | 0.156 (0.146 to 0.166) | <0.001 |
| Democratic People's Republic of Korea | 9889.27 (9857.50 to 9921.05) | 9557.20 (9535.33 to 9579.07) | -0.113 (-0.123 to -0.103) | <0.001 | 47158.28 (47088.58 to 47227.97) | 48338.15 (48288.76 to 48387.54) | 0.082 (0.077 to 0.087) | <0.001 | 4357.14 (4335.99 to 4378.29) | 4401.96 (4387.07 to 4416.86) | 0.034 (0.028 to 0.041) | <0.001 |
| Democratic Republic of the Congo | 10184.11 (10151.01 to 10217.22) | 9994.30 (9972.92 to 10015.68) | -0.063 (-0.071 to -0.055) | <0.001 | 45080.30 (45009.95 to 45150.64) | 45356.56 (45310.50 to 45402.62) | 0.020 (0.011 to 0.028) | <0.001 | 4033.16 (4012.27 to 4054.05) | 4059.18 (4045.48 to 4072.88) | 0.017 (0.005 to 0.028) | 0.006 |
| Denmark | 11593.20 (11540.70 to 11645.71) | 10720.94 (10679.31 to 10762.57) | -0.256 (-0.270 to -0.242) | <0.001 | 56261.16 (56146.05 to 56376.27) | 54583.90 (54490.43 to 54677.37) | -0.092 (-0.144 to -0.040) | <0.001 | 5684.77 (5648.06 to 5721.48) | 5456.39 (5426.80 to 5485.99) | -0.129 (-0.161 to -0.097) | <0.001 |
| Djibouti | 9592.76 (9244.39 to 9941.13) | 9443.26 (9286.39 to 9600.14) | -0.048 (-0.059 to -0.038) | <0.001 | 42612.31 (41870.60 to 43354.01) | 45151.70 (44804.98 to 45498.42) | 0.189 (0.182 to 0.196) | <0.001 | 3804.72 (3584.51 to 4024.92) | 3903.07 (3801.66 to 4004.48) | 0.087 (0.080 to 0.093) | <0.001 |
| Dominica | 8232.73 (7727.80 to 8737.67) | 8067.52 (7668.81 to 8466.22) | -0.067 (-0.079 to -0.055) | <0.001 | 45703.78 (44517.55 to 46890.01) | 47827.49 (46853.45 to 48801.53) | 0.146 (0.141 to 0.152) | <0.001 | 3916.09 (3568.13 to 4264.05) | 4012.58 (3730.63 to 4294.53) | 0.079 (0.071 to 0.088) | <0.001 |
| Dominican Republic | 7802.40 (7741.72 to 7863.09) | 8162.72 (8124.99 to 8200.45) | 0.145 (0.140 to 0.151) | <0.001 | 44032.05 (43886.95 to 44177.15) | 47435.97 (47344.76 to 47527.19) | 0.239 (0.232 to 0.247) | <0.001 | 3669.99 (3628.13 to 3711.86) | 3927.43 (3901.21 to 3953.66) | 0.220 (0.203 to 0.236) | <0.001 |
| Ecuador | 7928.50 (7877.07 to 7979.93) | 7999.90 (7970.68 to 8029.13) | 0.022 (-0.004 to 0.049) | 0.097 | 46679.20 (46553.69 to 46804.72) | 49504.20 (49431.37 to 49577.03) | 0.186 (0.171 to 0.202) | <0.001 | 4005.05 (3968.33 to 4041.76) | 4208.35 (4187.12 to 4229.58) | 0.153 (0.093 to 0.214) | <0.001 |
| Egypt | 9805.27 (9780.33 to 9830.20) | 10139.46 (10123.20 to 10155.73) | 0.109 (0.098 to 0.120) | <0.001 | 44890.60 (44837.17 to 44944.03) | 49164.19 (49128.40 to 49199.98) | 0.293 (0.289 to 0.298) | <0.001 | 4082.95 (4066.93 to 4098.96) | 4467.62 (4456.90 to 4478.34) | 0.291 (0.279 to 0.303) | <0.001 |
| El Salvador | 8578.74 (8507.10 to 8650.38) | 8930.52 (8879.54 to 8981.50) | 0.129 (0.126 to 0.133) | <0.001 | 47121.59 (46953.36 to 47289.81) | 51723.65 (51601.15 to 51846.15) | 0.305 (0.292 to 0.317) | <0.001 | 4146.18 (4096.36 to 4196.00) | 4618.83 (4582.18 to 4655.49) | 0.355 (0.337 to 0.372) | <0.001 |
| Equatorial Guinea | 9972.36 (9676.76 to 10267.97) | 10117.01 (9931.26 to 10302.76) | 0.046 (0.036 to 0.055) | <0.001 | 43352.29 (42729.97 to 43974.61) | 49531.86 (49116.62 to 49947.11) | 0.436 (0.426 to 0.447) | <0.001 | 3903.67 (3718.28 to 4089.07) | 4374.24 (4251.45 to 4497.04) | 0.369 (0.352 to 0.386) | <0.001 |
| Eritrea | 9344.95 (9225.91 to 9463.98) | 9488.07 (9411.28 to 9564.86) | 0.052 (0.046 to 0.058) | <0.001 | 37479.63 (37237.75 to 37721.51) | 41937.37 (41774.38 to 42100.37) | 0.365 (0.350 to 0.380) | <0.001 | 3155.04 (3085.69 to 3224.38) | 3604.72 (3557.24 to 3652.19) | 0.435 (0.421 to 0.449) | <0.001 |
| Estonia | 12394.74 (12293.14 to 12496.35) | 12480.12 (12385.55 to 12574.69) | 0.021 (0.002 to 0.041) | 0.034 | 51168.67 (50961.93 to 51375.41) | 54097.42 (53902.07 to 54292.78) | 0.181 (0.168 to 0.194) | <0.001 | 4761.81 (4698.80 to 4824.83) | 5099.96 (5039.97 to 5159.96) | 0.223 (0.194 to 0.252) | <0.001 |
| Eswatini | 9076.76 (8840.89 to 9312.62) | 9243.68 (9073.96 to 9413.40) | 0.062 (0.052 to 0.072) | <0.001 | 44175.09 (43650.08 to 44700.10) | 47664.23 (47275.03 to 48053.43) | 0.246 (0.241 to 0.252) | <0.001 | 3898.78 (3743.41 to 4054.15) | 4099.73 (3985.89 to 4213.57) | 0.168 (0.140 to 0.196) | <0.001 |
| Ethiopia | 10606.88 (10576.31 to 10637.46) | 10302.95 (10282.37 to 10323.53) | -0.095 (-0.114 to -0.076) | <0.001 | 43893.97 (43831.25 to 43956.69) | 47030.06 (46985.80 to 47074.32) | 0.224 (0.218 to 0.230) | <0.001 | 3939.24 (3920.59 to 3957.90) | 4080.87 (4067.88 to 4093.87) | 0.120 (0.104 to 0.135) | <0.001 |
| Fiji | 9037.94 (8828.77 to 9247.11) | 9143.48 (9003.83 to 9283.13) | 0.037 (0.031 to 0.043) | <0.001 | 46438.40 (45960.53 to 46916.26) | 49787.87 (49460.36 to 50115.39) | 0.226 (0.219 to 0.233) | <0.001 | 3939.36 (3800.99 to 4077.74) | 4116.21 (4022.38 to 4210.03) | 0.144 (0.138 to 0.150) | <0.001 |
| Finland | 10663.78 (10611.33 to 10716.23) | 10331.42 (10290.94 to 10371.89) | -0.105 (-0.147 to -0.064) | <0.001 | 47347.53 (47237.38 to 47457.69) | 48661.19 (48574.22 to 48748.15) | 0.087 (0.070 to 0.104) | <0.001 | 4344.10 (4310.68 to 4377.52) | 4315.71 (4289.65 to 4341.78) | -0.027 (-0.069 to 0.015) | 0.204 |
| France | 10692.28 (10676.81 to 10707.74) | 10870.52 (10858.09 to 10882.95) | 0.056 (0.037 to 0.075) | <0.001 | 47132.20 (47099.90 to 47164.50) | 49527.33 (49501.05 to 49553.62) | 0.162 (0.132 to 0.192) | <0.001 | 4213.05 (4203.36 to 4222.73) | 4467.24 (4459.30 to 4475.18) | 0.190 (0.107 to 0.272) | <0.001 |
| Gabon | 9798.44 (9626.84 to 9970.04) | 9899.24 (9774.25 to 10024.23) | 0.032 (0.027 to 0.037) | <0.001 | 45048.81 (44679.04 to 45418.58) | 48546.64 (48267.28 to 48826.00) | 0.244 (0.238 to 0.249) | <0.001 | 4019.83 (3909.73 to 4129.92) | 4256.10 (4173.70 to 4338.50) | 0.186 (0.180 to 0.192) | <0.001 |
| Gambia | 9061.82 (8847.22 to 9276.42) | 9093.06 (8964.41 to 9221.72) | 0.013 (0.001 to 0.025) | 0.036 | 42690.22 (42221.29 to 43159.15) | 45676.51 (45386.43 to 45966.59) | 0.220 (0.215 to 0.225) | <0.001 | 3714.67 (3577.09 to 3852.24) | 3892.98 (3808.62 to 3977.34) | 0.156 (0.146 to 0.166) | <0.001 |
| Georgia | 10890.37 (10836.71 to 10944.04) | 10862.98 (10806.04 to 10919.91) | -0.008 (-0.024 to 0.009) | 0.373 | 45597.37 (45487.10 to 45707.64) | 46757.40 (46640.00 to 46874.81) | 0.091 (0.040 to 0.141) | <0.001 | 4053.71 (4020.88 to 4086.54) | 4063.07 (4028.38 to 4097.76) | 0.016 (-0.025 to 0.057) | 0.44 |
| Germany | 12115.56 (12102.49 to 12128.63) | 11752.75 (11741.87 to 11763.62) | -0.102 (-0.112 to -0.092) | <0.001 | 52787.96 (52760.73 to 52815.18) | 53430.09 (53407.12 to 53453.06) | 0.038 (0.026 to 0.051) | <0.001 | 5110.66 (5102.17 to 5119.14) | 5080.84 (5073.70 to 5087.97) | -0.020 (-0.062 to 0.022) | 0.354 |
| Ghana | 9245.96 (9195.37 to 9296.55) | 9043.47 (9013.15 to 9073.79) | -0.071 (-0.075 to -0.066) | <0.001 | 45818.62 (45705.07 to 45932.16) | 47032.10 (46962.24 to 47101.95) | 0.084 (0.079 to 0.090) | <0.001 | 3932.37 (3899.26 to 3965.47) | 4032.51 (4012.16 to 4052.85) | 0.083 (0.076 to 0.089) | <0.001 |
| Greece | 10650.00 (10615.03 to 10684.97) | 10749.46 (10719.24 to 10779.69) | 0.038 (-0.002 to 0.078) | 0.059 | 47888.01 (47813.94 to 47962.08) | 50887.32 (50822.37 to 50952.28) | 0.204 (0.171 to 0.237) | <0.001 | 4335.49 (4313.19 to 4357.80) | 4693.88 (4674.03 to 4713.74) | 0.257 (0.243 to 0.270) | <0.001 |
| Greenland | 9746.11 (9047.73 to 10444.50) | 9520.55 (9065.59 to 9975.50) | -0.078 (-0.093 to -0.064) | <0.001 | 49173.33 (47595.08 to 50751.58) | 52657.61 (51583.35 to 53731.87) | 0.220 (0.213 to 0.227) | <0.001 | 4575.91 (4098.67 to 5053.15) | 4856.29 (4532.46 to 5180.12) | 0.189 (0.174 to 0.204) | <0.001 |
| Grenada | 8004.39 (7547.08 to 8461.70) | 8061.14 (7723.14 to 8399.14) | 0.020 (0.014 to 0.027) | <0.001 | 44100.38 (43036.02 to 45164.74) | 47311.36 (46485.75 to 48136.98) | 0.228 (0.222 to 0.233) | <0.001 | 3744.53 (3433.21 to 4055.86) | 3965.03 (3726.29 to 4203.78) | 0.183 (0.135 to 0.231) | <0.001 |
| Guam | 9092.73 (8643.56 to 9541.91) | 9231.94 (8959.23 to 9504.65) | 0.049 (0.039 to 0.058) | <0.001 | 48256.76 (47216.90 to 49296.61) | 50990.46 (50350.81 to 51630.10) | 0.178 (0.170 to 0.186) | <0.001 | 4167.30 (3862.73 to 4471.86) | 4348.85 (4161.83 to 4535.88) | 0.138 (0.130 to 0.145) | <0.001 |
| Guatemala | 9127.26 (9060.08 to 9194.45) | 9156.88 (9118.68 to 9195.08) | 0.007 (0.001 to 0.013) | 0.018 | 47672.83 (47518.13 to 47827.53) | 50458.11 (50368.23 to 50548.00) | 0.185 (0.180 to 0.191) | <0.001 | 4366.15 (4319.56 to 4412.74) | 4589.65 (4562.58 to 4616.71) | 0.154 (0.113 to 0.196) | <0.001 |
| Guinea | 9376.18 (9305.58 to 9446.78) | 9348.45 (9294.49 to 9402.41) | -0.013 (-0.023 to -0.003) | 0.01 | 42485.56 (42334.74 to 42636.39) | 44008.75 (43891.05 to 44126.45) | 0.113 (0.106 to 0.121) | <0.001 | 3779.93 (3735.08 to 3824.77) | 3884.57 (3849.74 to 3919.39) | 0.088 (0.078 to 0.098) | <0.001 |
| Guinea-Bissau | 9215.37 (9012.98 to 9417.76) | 9198.08 (9049.83 to 9346.33) | -0.009 (-0.018 to 0.000) | 0.058 | 42213.55 (41777.03 to 42650.06) | 44082.99 (43755.16 to 44410.82) | 0.138 (0.130 to 0.146) | <0.001 | 3702.21 (3573.73 to 3830.69) | 3860.89 (3764.49 to 3957.28) | 0.133 (0.118 to 0.148) | <0.001 |
| Guyana | 7875.79 (7683.75 to 8067.83) | 8052.95 (7909.24 to 8196.65) | 0.070 (0.068 to 0.072) | <0.001 | 43446.79 (42992.95 to 43900.63) | 46684.72 (46336.10 to 47033.35) | 0.234 (0.225 to 0.243) | <0.001 | 3525.05 (3396.27 to 3653.82) | 3754.87 (3656.40 to 3853.34) | 0.211 (0.195 to 0.226) | <0.001 |
| Haiti | 7701.71 (7637.68 to 7765.74) | 7805.44 (7762.49 to 7848.38) | 0.043 (0.040 to 0.045) | <0.001 | 40327.58 (40179.68 to 40475.49) | 42210.11 (42109.19 to 42311.03) | 0.148 (0.142 to 0.154) | <0.001 | 3445.19 (3402.13 to 3488.26) | 3528.02 (3499.01 to 3557.03) | 0.077 (0.052 to 0.103) | <0.001 |
| Honduras | 8526.73 (8441.26 to 8612.21) | 8784.17 (8735.25 to 8833.09) | 0.094 (0.089 to 0.099) | <0.001 | 47464.82 (47261.93 to 47667.72) | 50863.65 (50745.32 to 50981.97) | 0.223 (0.212 to 0.234) | <0.001 | 4387.18 (4325.65 to 4448.70) | 4791.18 (4754.94 to 4827.42) | 0.282 (0.239 to 0.326) | <0.001 |
| Hungary | 13366.49 (13326.47 to 13406.51) | 13415.56 (13379.40 to 13451.72) | 0.010 (0.005 to 0.016) | <0.001 | 52533.75 (52454.43 to 52613.07) | 54633.52 (54561.00 to 54706.04) | 0.126 (0.121 to 0.131) | <0.001 | 5061.92 (5037.29 to 5086.56) | 5304.58 (5281.91 to 5327.26) | 0.152 (0.128 to 0.177) | <0.001 |
| Iceland | 11414.96 (11141.84 to 11688.09) | 11014.78 (10826.76 to 11202.80) | -0.112 (-0.123 to -0.102) | <0.001 | 51707.13 (51128.44 to 52285.82) | 52163.79 (51756.50 to 52571.08) | 0.029 (0.006 to 0.052) | 0.014 | 4824.18 (4647.02 to 5001.35) | 4831.74 (4707.44 to 4956.05) | 0.004 (-0.011 to 0.020) | 0.58 |
| India | 9508.99 (9503.03 to 9514.94) | 9059.53 (9055.91 to 9063.16) | -0.154 (-0.188 to -0.120) | <0.001 | 47043.70 (47030.45 to 47056.95) | 50646.84 (50638.28 to 50655.41) | 0.233 (0.213 to 0.254) | <0.001 | 4350.74 (4346.72 to 4354.76) | 4665.63 (4663.04 to 4668.22) | 0.219 (0.170 to 0.268) | <0.001 |
| Indonesia | 8718.96 (8706.58 to 8731.35) | 8796.89 (8789.13 to 8804.65) | 0.027 (0.016 to 0.037) | <0.001 | 42211.07 (42183.83 to 42238.31) | 45918.88 (45901.14 to 45936.61) | 0.273 (0.266 to 0.280) | <0.001 | 3731.09 (3723.03 to 3739.15) | 4018.50 (4013.28 to 4023.71) | 0.242 (0.227 to 0.258) | <0.001 |
| Iran (Islamic Republic of) | 11461.59 (11434.54 to 11488.63) | 11079.59 (11063.93 to 11095.25) | -0.100 (-0.124 to -0.076) | <0.001 | 47256.20 (47201.05 to 47311.36) | 50358.04 (50324.64 to 50391.44) | 0.207 (0.197 to 0.217) | <0.001 | 4448.68 (4431.86 to 4465.49) | 4697.11 (4686.94 to 4707.28) | 0.181 (0.171 to 0.191) | <0.001 |
| Iraq | 9921.85 (9874.28 to 9969.42) | 9977.56 (9950.91 to 10004.22) | 0.018 (0.016 to 0.021) | <0.001 | 43655.49 (43555.65 to 43755.34) | 47354.92 (47296.76 to 47413.07) | 0.263 (0.256 to 0.269) | <0.001 | 3812.74 (3783.30 to 3842.18) | 4151.07 (4133.94 to 4168.20) | 0.271 (0.261 to 0.282) | <0.001 |
| Ireland | 11191.83 (11120.96 to 11262.70) | 11174.61 (11123.67 to 11225.55) | -0.008 (-0.034 to 0.018) | 0.546 | 50358.66 (50208.66 to 50508.66) | 52215.58 (52105.83 to 52325.32) | 0.116 (0.111 to 0.120) | <0.001 | 4899.65 (4852.86 to 4946.43) | 5001.11 (4967.12 to 5035.10) | 0.066 (0.048 to 0.084) | <0.001 |
| Israel | 11256.42 (11191.23 to 11321.61) | 11054.29 (11013.16 to 11095.41) | -0.058 (-0.084 to -0.032) | <0.001 | 50751.89 (50613.80 to 50889.97) | 51878.84 (51790.17 to 51967.51) | 0.066 (0.030 to 0.101) | <0.001 | 4787.73 (4745.28 to 4830.18) | 4829.11 (4802.00 to 4856.21) | 0.023 (0.004 to 0.042) | 0.017 |
| Italy | 11455.89 (11440.70 to 11471.08) | 11515.55 (11502.96 to 11528.13) | 0.021 (-0.019 to 0.061) | 0.302 | 50082.28 (50050.61 to 50113.96) | 52433.40 (52406.83 to 52459.98) | 0.151 (0.142 to 0.160) | <0.001 | 4586.81 (4577.20 to 4596.41) | 4896.60 (4888.41 to 4904.79) | 0.218 (0.196 to 0.240) | <0.001 |
| Jamaica | 8033.62 (7942.34 to 8124.89) | 8156.43 (8088.67 to 8224.19) | 0.047 (0.040 to 0.054) | <0.001 | 44825.24 (44610.61 to 45039.88) | 47619.68 (47456.08 to 47783.28) | 0.196 (0.190 to 0.202) | <0.001 | 3892.24 (3828.84 to 3955.65) | 4099.47 (4051.49 to 4147.45) | 0.178 (0.134 to 0.222) | <0.001 |
| Japan | 12178.76 (12167.65 to 12189.88) | 11611.93 (11603.49 to 11620.38) | -0.176 (-0.226 to -0.126) | <0.001 | 57609.25 (57585.06 to 57633.43) | 59333.29 (59314.57 to 59352.00) | 0.094 (0.071 to 0.116) | <0.001 | 5668.59 (5661.01 to 5676.18) | 5789.79 (5783.91 to 5795.67) | 0.046 (-0.017 to 0.110) | 0.149 |
| Jordan | 9953.09 (9840.92 to 10065.26) | 10122.33 (10075.41 to 10169.26) | 0.054 (0.036 to 0.073) | <0.001 | 46981.96 (46738.04 to 47225.88) | 50789.36 (50684.17 to 50894.56) | 0.253 (0.249 to 0.256) | <0.001 | 4316.60 (4243.00 to 4390.20) | 4613.10 (4581.52 to 4644.67) | 0.214 (0.198 to 0.230) | <0.001 |
| Kazakhstan | 11567.68 (11528.56 to 11606.80) | 11586.16 (11553.23 to 11619.09) | 0.006 (0.001 to 0.011) | 0.023 | 48111.97 (48031.54 to 48192.41) | 51563.71 (51493.83 to 51633.58) | 0.223 (0.218 to 0.228) | <0.001 | 4315.02 (4290.97 to 4339.07) | 4555.45 (4534.70 to 4576.21) | 0.172 (0.138 to 0.206) | <0.001 |
| Kenya | 10981.10 (10932.47 to 11029.73) | 10963.56 (10934.83 to 10992.29) | -0.006 (-0.020 to 0.007) | 0.336 | 46620.07 (46519.34 to 46720.79) | 49553.65 (49492.14 to 49615.15) | 0.198 (0.190 to 0.205) | <0.001 | 4259.74 (4229.40 to 4290.09) | 4443.89 (4425.54 to 4462.24) | 0.128 (0.116 to 0.140) | <0.001 |
| Kiribati | 9173.54 (8510.39 to 9836.69) | 9429.49 (8961.74 to 9897.25) | 0.087 (0.076 to 0.098) | <0.001 | 46742.23 (45236.75 to 48247.70) | 49214.40 (48139.63 to 50289.16) | 0.168 (0.160 to 0.176) | <0.001 | 3926.49 (3491.98 to 4361.01) | 4157.82 (3846.51 to 4469.13) | 0.186 (0.180 to 0.193) | <0.001 |
| Kuwait | 9548.36 (9380.86 to 9715.86) | 9976.96 (9903.30 to 10050.61) | 0.143 (0.122 to 0.163) | <0.001 | 47295.55 (46922.74 to 47668.36) | 51141.11 (50974.57 to 51307.65) | 0.257 (0.209 to 0.304) | <0.001 | 4199.78 (4089.24 to 4310.33) | 4580.52 (4530.95 to 4630.09) | 0.283 (0.231 to 0.335) | <0.001 |
| Kyrgyzstan | 11355.38 (11275.23 to 11435.52) | 11278.32 (11216.01 to 11340.63) | -0.024 (-0.028 to -0.019) | <0.001 | 46973.34 (46809.46 to 47137.21) | 49392.65 (49261.37 to 49523.93) | 0.161 (0.150 to 0.172) | <0.001 | 4327.30 (4277.62 to 4376.99) | 4581.81 (4541.81 to 4621.81) | 0.171 (0.099 to 0.243) | <0.001 |
| Lao People's Democratic Republic | 8143.71 (8060.57 to 8226.85) | 8017.80 (7963.39 to 8072.21) | -0.050 (-0.057 to -0.043) | <0.001 | 39529.39 (39346.13 to 39712.65) | 42525.31 (42399.98 to 42650.63) | 0.237 (0.230 to 0.244) | <0.001 | 3520.92 (3466.40 to 3575.45) | 3723.30 (3686.36 to 3760.25) | 0.180 (0.173 to 0.188) | <0.001 |
| Latvia | 12309.28 (12232.63 to 12385.94) | 12346.74 (12270.10 to 12423.37) | 0.007 (0.005 to 0.010) | <0.001 | 50160.42 (50005.56 to 50315.28) | 53116.55 (52958.71 to 53274.39) | 0.185 (0.174 to 0.196) | <0.001 | 4589.17 (4542.35 to 4636.00) | 4917.00 (4868.94 to 4965.06) | 0.229 (0.209 to 0.249) | <0.001 |
| Lebanon | 9724.00 (9637.10 to 9810.91) | 10051.79 (9996.40 to 10107.18) | 0.109 (0.097 to 0.120) | <0.001 | 44148.03 (43962.85 to 44333.21) | 50128.04 (50004.38 to 50251.70) | 0.415 (0.400 to 0.431) | <0.001 | 3939.12 (3884.03 to 3994.22) | 4496.29 (4459.18 to 4533.39) | 0.431 (0.414 to 0.448) | <0.001 |
| Lesotho | 9584.97 (9443.85 to 9726.09) | 9564.73 (9440.21 to 9689.25) | -0.006 (-0.013 to 0.000) | 0.066 | 44167.57 (43863.20 to 44471.94) | 46924.14 (46646.31 to 47201.97) | 0.197 (0.182 to 0.212) | <0.001 | 3998.68 (3907.34 to 4090.01) | 4089.12 (4007.31 to 4170.93) | 0.073 (0.050 to 0.095) | <0.001 |
| Liberia | 9295.90 (9175.73 to 9416.07) | 9255.43 (9167.23 to 9343.63) | -0.013 (-0.016 to -0.010) | <0.001 | 43229.07 (42968.93 to 43489.20) | 45290.73 (45093.93 to 45487.53) | 0.152 (0.144 to 0.160) | <0.001 | 3718.28 (3642.24 to 3794.33) | 3825.36 (3768.44 to 3882.27) | 0.092 (0.077 to 0.107) | <0.001 |
| Libya | 9831.40 (9735.73 to 9927.07) | 10042.43 (9985.65 to 10099.22) | 0.069 (0.062 to 0.077) | <0.001 | 45921.13 (45714.44 to 46127.81) | 49189.05 (49063.52 to 49314.59) | 0.222 (0.212 to 0.231) | <0.001 | 4122.82 (4061.04 to 4184.60) | 4385.95 (4348.67 to 4423.23) | 0.199 (0.185 to 0.213) | <0.001 |
| Lithuania | 12394.22 (12325.62 to 12462.82) | 12431.96 (12368.65 to 12495.27) | 0.010 (0.004 to 0.016) | 0.002 | 50271.66 (50133.46 to 50409.86) | 52999.49 (52869.56 to 53129.42) | 0.171 (0.165 to 0.176) | <0.001 | 4661.25 (4619.18 to 4703.31) | 4943.10 (4903.37 to 4982.83) | 0.193 (0.169 to 0.217) | <0.001 |
| Luxembourg | 11195.33 (11003.98 to 11386.69) | 10971.68 (10835.10 to 11108.25) | -0.066 (-0.073 to -0.059) | <0.001 | 49295.49 (48894.71 to 49696.27) | 50870.41 (50577.17 to 51163.64) | 0.100 (0.093 to 0.106) | <0.001 | 4524.59 (4403.03 to 4646.15) | 4652.97 (4564.11 to 4741.84) | 0.087 (0.071 to 0.102) | <0.001 |
| Madagascar | 10150.91 (10091.76 to 10210.06) | 9808.20 (9769.94 to 9846.46) | -0.109 (-0.123 to -0.094) | <0.001 | 43361.10 (43238.19 to 43484.01) | 43777.97 (43696.40 to 43859.55) | 0.031 (0.025 to 0.037) | <0.001 | 3982.03 (3944.91 to 4019.15) | 3963.47 (3939.06 to 3987.87) | -0.011 (-0.022 to 0.000) | 0.042 |
| Malawi | 9975.47 (9907.62 to 10043.33) | 10012.53 (9963.47 to 10061.59) | 0.013 (0.008 to 0.018) | <0.001 | 43298.52 (43156.44 to 43440.60) | 45222.64 (45117.74 to 45327.54) | 0.143 (0.138 to 0.148) | <0.001 | 3916.73 (3874.18 to 3959.28) | 4062.62 (4031.28 to 4093.96) | 0.123 (0.110 to 0.136) | <0.001 |
| Malaysia | 7813.46 (7775.01 to 7851.92) | 8010.38 (7988.37 to 8032.39) | 0.078 (0.060 to 0.096) | <0.001 | 42109.13 (42019.75 to 42198.51) | 45976.16 (45923.47 to 46028.84) | 0.284 (0.276 to 0.291) | <0.001 | 3591.00 (3564.99 to 3617.00) | 3896.86 (3881.57 to 3912.15) | 0.261 (0.245 to 0.278) | <0.001 |
| Maldives | 7464.04 (7094.85 to 7833.22) | 7668.38 (7470.89 to 7865.87) | 0.085 (0.070 to 0.099) | <0.001 | 40014.37 (39157.15 to 40871.60) | 44588.45 (44111.23 to 45065.67) | 0.350 (0.338 to 0.362) | <0.001 | 3355.54 (3109.39 to 3601.70) | 3708.02 (3570.98 to 3845.07) | 0.324 (0.312 to 0.335) | <0.001 |
| Mali | 8619.56 (8558.78 to 8680.34) | 8831.93 (8790.55 to 8873.30) | 0.080 (0.073 to 0.086) | <0.001 | 40421.12 (40288.56 to 40553.67) | 42681.36 (42589.71 to 42773.01) | 0.175 (0.168 to 0.182) | <0.001 | 3451.17 (3412.69 to 3489.64) | 3633.75 (3607.18 to 3660.33) | 0.166 (0.152 to 0.178) | <0.001 |
| Malta | 11283.91 (11067.10 to 11500.73) | 11350.00 (11198.22 to 11501.78) | 0.015 (0.003 to 0.028) | 0.014 | 50058.32 (49601.48 to 50515.16) | 52025.44 (51703.43 to 52347.45) | 0.123 (0.114 to 0.132) | <0.001 | 4685.48 (4545.79 to 4825.16) | 4867.61 (4768.65 to 4966.57) | 0.120 (0.095 to 0.144) | <0.001 |
| Marshall Islands | 8870.24 (7865.25 to 9875.23) | 8865.99 (8217.31 to 9514.67) | -0.003 (-0.011 to 0.006) | 0.543 | 45444.35 (43160.05 to 47728.65) | 47772.47 (46253.98 to 49290.96) | 0.161 (0.154 to 0.168) | <0.001 | 3813.90 (3154.77 to 4473.03) | 3896.18 (3465.36 to 4327.01) | 0.070 (0.057 to 0.084) | <0.001 |
| Mauritania | 8795.77 (8671.04 to 8920.50) | 8992.71 (8907.98 to 9077.44) | 0.070 (0.057 to 0.082) | <0.001 | 42724.57 (42448.50 to 43000.63) | 45881.76 (45689.32 to 46074.19) | 0.230 (0.226 to 0.233) | <0.001 | 3677.61 (3596.92 to 3758.30) | 3934.53 (3878.37 to 3990.68) | 0.218 (0.204 to 0.231) | <0.001 |
| Mauritius | 8296.80 (8154.37 to 8439.23) | 8270.07 (8183.18 to 8356.95) | -0.010 (-0.013 to -0.008) | <0.001 | 44384.08 (44054.88 to 44713.28) | 47281.14 (47073.87 to 47488.41) | 0.203 (0.194 to 0.213) | <0.001 | 3836.32 (3739.69 to 3932.95) | 4071.93 (4011.13 to 4132.73) | 0.187 (0.152 to 0.223) | <0.001 |
| Mexico | 8597.92 (8579.14 to 8616.69) | 8912.50 (8901.56 to 8923.44) | 0.118 (0.104 to 0.132) | <0.001 | 50962.14 (50916.22 to 51008.06) | 55036.67 (55009.39 to 55063.95) | 0.247 (0.236 to 0.259) | <0.001 | 4675.89 (4662.01 to 4689.77) | 5074.17 (5065.90 to 5082.44) | 0.269 (0.233 to 0.305) | <0.001 |
| Micronesia (Federated States of) | 9117.84 (8531.21 to 9704.47) | 9350.78 (8898.36 to 9803.21) | 0.080 (0.072 to 0.087) | <0.001 | 46054.89 (44734.04 to 47375.73) | 49015.73 (47974.66 to 50056.80) | 0.202 (0.198 to 0.206) | <0.001 | 3918.74 (3534.54 to 4302.94) | 4106.12 (3806.10 to 4406.14) | 0.153 (0.142 to 0.165) | <0.001 |
| Monaco | 11026.52 (10466.05 to 11586.98) | 10821.41 (10356.20 to 11286.61) | -0.060 (-0.068 to -0.053) | <0.001 | 51302.65 (50105.64 to 52499.66) | 51853.13 (50843.84 to 52862.41) | 0.034 (0.027 to 0.040) | <0.001 | 4746.93 (4380.70 to 5113.15) | 4721.88 (4415.79 to 5027.98) | -0.015 (-0.022 to -0.009) | <0.001 |
| Mongolia | 11074.19 (10937.60 to 11210.79) | 11238.64 (11149.43 to 11327.86) | 0.048 (0.042 to 0.054) | <0.001 | 44829.63 (44553.15 to 45106.10) | 49527.17 (49337.46 to 49716.88) | 0.322 (0.316 to 0.327) | <0.001 | 4059.71 (3976.68 to 4142.73) | 4399.07 (4342.56 to 4455.57) | 0.259 (0.250 to 0.269) | <0.001 |
| Montenegro | 12765.17 (12580.75 to 12949.59) | 12893.10 (12741.92 to 13044.27) | 0.032 (0.018 to 0.046) | <0.001 | 50407.11 (50038.94 to 50775.28) | 52255.76 (51951.45 to 52560.07) | 0.118 (0.112 to 0.123) | <0.001 | 4733.56 (4621.00 to 4846.12) | 4896.70 (4803.56 to 4989.84) | 0.110 (0.097 to 0.123) | <0.001 |
| Morocco | 10406.48 (10370.52 to 10442.45) | 10550.22 (10527.44 to 10572.99) | 0.046 (0.036 to 0.056) | <0.001 | 46348.66 (46272.64 to 46424.69) | 49355.38 (49306.12 to 49404.63) | 0.202 (0.192 to 0.212) | <0.001 | 4315.55 (4292.43 to 4338.68) | 4567.85 (4552.89 to 4582.80) | 0.182 (0.174 to 0.191) | <0.001 |
| Mozambique | 10123.69 (10069.63 to 10177.74) | 10206.20 (10166.25 to 10246.14) | 0.028 (0.023 to 0.033) | <0.001 | 42377.53 (42266.24 to 42488.82) | 44888.86 (44804.42 to 44973.30) | 0.188 (0.180 to 0.196) | <0.001 | 3787.86 (3754.77 to 3820.94) | 4011.29 (3986.14 to 4036.44) | 0.192 (0.148 to 0.236) | <0.001 |
| Myanmar | 7339.32 (7315.74 to 7362.90) | 7598.76 (7582.53 to 7614.99) | 0.114 (0.105 to 0.123) | <0.001 | 37941.30 (37887.79 to 37994.81) | 42072.52 (42034.40 to 42110.65) | 0.336 (0.324 to 0.348) | <0.001 | 3217.99 (3202.49 to 3233.50) | 3525.28 (3514.28 to 3536.27) | 0.297 (0.284 to 0.310) | <0.001 |
| Namibia | 9678.18 (9516.74 to 9839.63) | 9729.65 (9619.55 to 9839.74) | 0.019 (0.014 to 0.024) | <0.001 | 44062.79 (43716.20 to 44409.37) | 47340.02 (47095.20 to 47584.85) | 0.234 (0.224 to 0.244) | <0.001 | 3985.59 (3881.80 to 4089.37) | 4218.93 (4146.06 to 4291.81) | 0.186 (0.173 to 0.199) | <0.001 |
| Nauru | 8906.20 (7050.25 to 10762.14) | 9458.59 (7768.75 to 11148.42) | 0.195 (0.185 to 0.206) | <0.001 | 45860.81 (41619.88 to 50101.74) | 49747.68 (45848.32 to 53647.04) | 0.265 (0.252 to 0.278) | <0.001 | 3836.79 (2618.44 to 5055.15) | 4158.76 (3036.82 to 5280.70) | 0.264 (0.246 to 0.283) | <0.001 |
| Nepal | 10994.29 (10949.44 to 11039.13) | 10807.66 (10779.45 to 10835.86) | -0.053 (-0.059 to -0.048) | <0.001 | 49683.10 (49587.35 to 49778.85) | 52236.88 (52174.77 to 52298.99) | 0.164 (0.158 to 0.171) | <0.001 | 5032.39 (5002.01 to 5062.77) | 5254.18 (5234.50 to 5273.86) | 0.134 (0.113 to 0.155) | <0.001 |
| Netherlands | 10218.08 (10187.29 to 10248.87) | 10307.98 (10284.62 to 10331.34) | 0.029 (-0.002 to 0.059) | 0.063 | 49887.22 (49819.48 to 49954.96) | 50339.29 (50287.89 to 50390.68) | 0.032 (0.016 to 0.048) | <0.001 | 4631.52 (4610.86 to 4652.17) | 4553.87 (4538.37 to 4569.38) | -0.055 (-0.091 to -0.018) | 0.003 |
| New Zealand | 12660.09 (12583.22 to 12736.96) | 12418.80 (12366.88 to 12470.72) | -0.067 (-0.080 to -0.054) | <0.001 | 53346.66 (53189.49 to 53503.84) | 55152.07 (55043.29 to 55260.85) | 0.108 (0.096 to 0.120) | <0.001 | 4961.09 (4913.15 to 5009.03) | 5052.30 (5019.34 to 5085.27) | 0.063 (0.029 to 0.098) | <0.001 |
| Nicaragua | 8666.82 (8566.97 to 8766.66) | 8866.54 (8810.59 to 8922.50) | 0.073 (0.065 to 0.081) | <0.001 | 46782.90 (46549.96 to 47015.85) | 51062.16 (50927.37 to 51196.95) | 0.281 (0.273 to 0.290) | <0.001 | 4241.41 (4171.54 to 4311.28) | 4635.37 (4594.86 to 4675.89) | 0.285 (0.263 to 0.307) | <0.001 |
| Niger | 9132.71 (9056.65 to 9208.78) | 9316.52 (9272.72 to 9360.33) | 0.060 (0.028 to 0.093) | <0.001 | 41238.97 (41075.98 to 41401.96) | 42772.62 (42678.07 to 42867.17) | 0.117 (0.111 to 0.124) | <0.001 | 3614.84 (3566.94 to 3662.74) | 3775.95 (3748.04 to 3803.86) | 0.140 (0.132 to 0.147) | <0.001 |
| Nigeria | 9814.95 (9795.29 to 9834.61) | 9874.48 (9861.09 to 9887.87) | 0.018 (0.008 to 0.028) | <0.001 | 45050.54 (45008.19 to 45092.90) | 47624.81 (47595.12 to 47654.51) | 0.180 (0.173 to 0.187) | <0.001 | 3962.57 (3950.07 to 3975.08) | 4206.84 (4198.08 to 4215.60) | 0.193 (0.176 to 0.210) | <0.001 |
| Niue | 9204.21 (6477.60 to 11930.83) | 9285.93 (6613.00 to 11958.86) | 0.024 (0.008 to 0.040) | 0.003 | 47805.80 (41593.93 to 54017.67) | 50645.85 (44399.12 to 56892.59) | 0.187 (0.177 to 0.198) | <0.001 | 4064.67 (2251.46 to 5877.89) | 4210.74 (2411.16 to 6010.31) | 0.114 (0.102 to 0.126) | <0.001 |
| North Macedonia | 12267.98 (12163.06 to 12372.90) | 12362.58 (12283.16 to 12442.00) | 0.024 (0.018 to 0.029) | <0.001 | 47596.28 (47388.58 to 47803.99) | 49949.93 (49789.82 to 50110.05) | 0.156 (0.151 to 0.161) | <0.001 | 4417.14 (4354.07 to 4480.21) | 4583.77 (4535.36 to 4632.18) | 0.119 (0.113 to 0.126) | <0.001 |
| Northern Mariana Islands | 8973.14 (8010.93 to 9935.34) | 9116.75 (8599.72 to 9633.78) | 0.052 (-0.032 to 0.137) | 0.222 | 47587.50 (45348.26 to 49826.74) | 49649.75 (48436.94 to 50862.56) | 0.142 (0.093 to 0.190) | <0.001 | 3984.53 (3340.41 to 4628.64) | 4132.63 (3784.73 to 4480.53) | 0.121 (0.035 to 0.206) | 0.006 |
| Norway | 11163.42 (11106.48 to 11220.35) | 10881.59 (10836.18 to 10927.00) | -0.109 (-0.139 to -0.078) | <0.001 | 49016.04 (48897.73 to 49134.35) | 49756.27 (49659.78 to 49852.77) | 0.041 (-0.016 to 0.098) | 0.156 | 4581.35 (4545.08 to 4617.63) | 4487.74 (4458.68 to 4516.81) | -0.071 (-0.131 to -0.011) | 0.021 |
| Oman | 9659.81 (9498.83 to 9820.78) | 9808.79 (9717.69 to 9899.89) | 0.052 (0.047 to 0.056) | <0.001 | 43481.27 (43138.65 to 43823.90) | 49030.48 (48826.91 to 49234.05) | 0.390 (0.382 to 0.397) | <0.001 | 3838.54 (3737.38 to 3939.69) | 4347.87 (4287.62 to 4408.12) | 0.407 (0.394 to 0.421) | <0.001 |
| Pakistan | 9036.87 (9020.07 to 9053.67) | 9749.75 (9738.09 to 9761.41) | 0.240 (0.207 to 0.273) | <0.001 | 42991.81 (42955.02 to 43028.60) | 48340.03 (48313.86 to 48366.20) | 0.380 (0.364 to 0.396) | <0.001 | 4045.09 (4033.82 to 4056.36) | 4631.93 (4623.84 to 4640.03) | 0.439 (0.414 to 0.464) | <0.001 |
| Palau | 9088.40 (7787.55 to 10389.25) | 9180.08 (8389.28 to 9970.88) | 0.031 (0.007 to 0.054) | 0.011 | 47118.50 (44151.83 to 50085.18) | 49607.05 (47759.11 to 51455.00) | 0.166 (0.159 to 0.173) | <0.001 | 3914.73 (3063.27 to 4766.20) | 4023.94 (3501.15 to 4546.74) | 0.089 (0.064 to 0.113) | <0.001 |
| Palestine | 10021.15 (9878.42 to 10163.87) | 9967.40 (9886.68 to 10048.11) | -0.016 (-0.021 to -0.011) | <0.001 | 45168.44 (44865.09 to 45471.79) | 47482.60 (47306.32 to 47658.88) | 0.157 (0.136 to 0.178) | <0.001 | 4113.04 (4021.78 to 4204.29) | 4198.69 (4146.57 to 4250.80) | 0.064 (0.038 to 0.089) | <0.001 |
| Panama | 8412.15 (8311.90 to 8512.41) | 8691.25 (8632.63 to 8749.87) | 0.102 (0.092 to 0.111) | <0.001 | 48640.82 (48399.17 to 48882.47) | 52736.56 (52592.11 to 52881.01) | 0.261 (0.257 to 0.265) | <0.001 | 4433.80 (4360.97 to 4506.64) | 4823.19 (4779.50 to 4866.87) | 0.271 (0.262 to 0.280) | <0.001 |
| Papua New Guinea | 8647.72 (8556.82 to 8738.63) | 8673.94 (8620.37 to 8727.50) | 0.010 (-0.002 to 0.022) | 0.108 | 41792.12 (41591.41 to 41992.82) | 43362.49 (43241.89 to 43483.09) | 0.119 (0.111 to 0.127) | <0.001 | 3537.97 (3480.07 to 3595.87) | 3608.84 (3574.34 to 3643.33) | 0.064 (0.052 to 0.077) | <0.001 |
| Paraguay | 8744.16 (8660.55 to 8827.78) | 9157.65 (9105.53 to 9209.76) | 0.150 (0.144 to 0.156) | <0.001 | 49211.18 (49012.14 to 49410.22) | 51596.64 (51472.59 to 51720.68) | 0.152 (0.147 to 0.158) | <0.001 | 4371.62 (4312.43 to 4430.81) | 4717.56 (4680.12 to 4754.99) | 0.246 (0.235 to 0.258) | <0.001 |
| Peru | 7722.56 (7688.88 to 7756.23) | 8085.47 (8064.93 to 8106.02) | 0.145 (0.132 to 0.157) | <0.001 | 46398.15 (46315.22 to 46481.08) | 49882.77 (49831.67 to 49933.87) | 0.235 (0.221 to 0.250) | <0.001 | 3966.55 (3942.35 to 3990.76) | 4306.91 (4291.89 to 4321.92) | 0.266 (0.241 to 0.290) | <0.001 |
| Philippines | 8489.87 (8467.48 to 8512.25) | 8726.81 (8713.38 to 8740.23) | 0.090 (0.074 to 0.105) | <0.001 | 41573.79 (41524.04 to 41623.53) | 44583.15 (44552.77 to 44613.53) | 0.226 (0.217 to 0.236) | <0.001 | 3657.64 (3642.96 to 3672.32) | 3882.23 (3873.30 to 3891.17) | 0.193 (0.185 to 0.200) | <0.001 |
| Poland | 13857.08 (13833.62 to 13880.54) | 13850.90 (13831.85 to 13869.95) | -0.002 (-0.024 to 0.020) | 0.857 | 52224.78 (52179.19 to 52270.37) | 53929.21 (53891.91 to 53966.51) | 0.103 (0.100 to 0.106) | <0.001 | 5163.74 (5149.41 to 5178.07) | 5150.77 (5139.21 to 5162.33) | -0.011 (-0.036 to 0.013) | 0.353 |
| Portugal | 11453.78 (11415.87 to 11491.68) | 11484.79 (11454.29 to 11515.29) | 0.004 (-0.011 to 0.020) | 0.583 | 49055.43 (48977.03 to 49133.83) | 52188.36 (52124.04 to 52252.69) | 0.198 (0.172 to 0.224) | <0.001 | 4571.34 (4547.41 to 4595.27) | 4915.77 (4895.91 to 4935.62) | 0.228 (0.176 to 0.280) | <0.001 |
| Puerto Rico | 8066.79 (8003.64 to 8129.94) | 8252.05 (8204.54 to 8299.56) | 0.071 (0.066 to 0.076) | <0.001 | 47360.42 (47207.60 to 47513.24) | 50120.04 (50004.38 to 50235.70) | 0.184 (0.178 to 0.189) | <0.001 | 3915.37 (3871.37 to 3959.37) | 4119.21 (4085.83 to 4152.59) | 0.160 (0.139 to 0.182) | <0.001 |
| Qatar | 9602.30 (9221.13 to 9983.46) | 9898.94 (9776.97 to 10020.92) | 0.101 (0.087 to 0.116) | <0.001 | 45966.04 (45128.90 to 46803.17) | 50049.19 (49775.01 to 50323.37) | 0.270 (0.264 to 0.277) | <0.001 | 3976.64 (3732.24 to 4221.03) | 4357.09 (4276.87 to 4437.32) | 0.293 (0.279 to 0.307) | <0.001 |
| Republic of Korea | 10832.80 (10808.24 to 10857.36) | 10467.01 (10453.25 to 10480.77) | -0.114 (-0.162 to -0.065) | <0.001 | 54130.22 (54074.55 to 54185.88) | 57088.41 (57056.29 to 57120.53) | 0.173 (0.153 to 0.194) | <0.001 | 4837.72 (4821.12 to 4854.32) | 5092.82 (5083.24 to 5102.41) | 0.160 (0.134 to 0.187) | <0.001 |
| Republic of Moldova | 12219.25 (12150.88 to 12287.61) | 12318.37 (12257.88 to 12378.85) | 0.025 (0.020 to 0.031) | <0.001 | 47975.16 (47839.00 to 48111.32) | 51299.47 (51176.42 to 51422.52) | 0.216 (0.210 to 0.222) | <0.001 | 4411.87 (4370.65 to 4453.09) | 4767.85 (4730.31 to 4805.38) | 0.248 (0.232 to 0.264) | <0.001 |
| Romania | 13069.55 (13041.68 to 13097.42) | 12900.11 (12874.77 to 12925.46) | -0.043 (-0.052 to -0.034) | <0.001 | 49982.27 (49927.50 to 50037.03) | 51103.64 (51053.46 to 51153.81) | 0.071 (0.065 to 0.077) | <0.001 | 4794.12 (4777.21 to 4811.04) | 4860.67 (4845.16 to 4876.17) | 0.044 (0.038 to 0.051) | <0.001 |
| Russian Federation | 13477.62 (13466.46 to 13488.77) | 13358.54 (13348.61 to 13368.48) | -0.030 (-0.039 to -0.021) | <0.001 | 54148.27 (54125.85 to 54170.69) | 54528.14 (54508.15 to 54548.14) | 0.026 (0.011 to 0.041) | <0.001 | 5021.63 (5014.81 to 5028.45) | 5079.22 (5073.12 to 5085.31) | 0.040 (0.014 to 0.066) | 0.003 |
| Rwanda | 10385.94 (10305.95 to 10465.93) | 10281.77 (10228.81 to 10334.73) | -0.029 (-0.044 to -0.014) | <0.001 | 43884.26 (43718.77 to 44049.74) | 43494.04 (43384.28 to 43603.80) | -0.025 (-0.044 to -0.006) | 0.01 | 4081.28 (4031.05 to 4131.52) | 3905.46 (3872.69 to 3938.23) | -0.139 (-0.162 to -0.117) | <0.001 |
| Saint Kitts and Nevis | 8083.47 (7447.20 to 8719.75) | 8091.99 (7663.79 to 8520.19) | 0.001 (-0.011 to 0.012) | 0.92 | 45954.74 (44450.33 to 47459.16) | 48609.37 (47551.52 to 49667.22) | 0.183 (0.176 to 0.190) | <0.001 | 3939.71 (3496.35 to 4383.06) | 4146.48 (3837.53 to 4455.44) | 0.173 (0.126 to 0.220) | <0.001 |
| Saint Lucia | 8178.34 (7767.48 to 8589.19) | 8132.36 (7894.14 to 8370.58) | -0.020 (-0.024 to -0.015) | <0.001 | 44749.93 (43789.06 to 45710.80) | 47800.97 (47221.65 to 48380.29) | 0.214 (0.205 to 0.223) | <0.001 | 3856.58 (3573.54 to 4139.63) | 4026.04 (3858.03 to 4194.05) | 0.137 (0.115 to 0.159) | <0.001 |
| Saint Vincent and the Grenadines | 7918.03 (7470.21 to 8365.85) | 7916.18 (7610.80 to 8221.57) | -0.002 (-0.007 to 0.004) | 0.538 | 44074.19 (43018.81 to 45129.57) | 46659.11 (45916.17 to 47402.05) | 0.186 (0.180 to 0.192) | <0.001 | 3687.39 (3381.65 to 3993.13) | 3861.09 (3647.39 to 4074.78) | 0.155 (0.141 to 0.168) | <0.001 |
| Samoa | 9360.01 (8925.15 to 9794.88) | 9262.68 (8933.62 to 9591.75) | -0.035 (-0.039 to -0.031) | <0.001 | 47311.21 (46330.40 to 48292.01) | 49145.29 (48384.46 to 49906.13) | 0.122 (0.118 to 0.126) | <0.001 | 4067.91 (3781.22 to 4354.60) | 4098.76 (3879.72 to 4317.81) | 0.024 (0.019 to 0.028) | <0.001 |
| San Marino | 10994.74 (10235.34 to 11754.14) | 10832.05 (10299.82 to 11364.29) | -0.047 (-0.060 to -0.034) | <0.001 | 50459.39 (48838.92 to 52079.86) | 51295.19 (50146.90 to 52443.47) | 0.052 (0.049 to 0.055) | <0.001 | 4652.09 (4158.99 to 5145.18) | 4638.74 (4290.92 to 4986.56) | -0.013 (-0.029 to 0.002) | 0.099 |
| Sao Tome and Principe | 8849.62 (8365.89 to 9333.35) | 8913.77 (8545.84 to 9281.70) | 0.020 (0.014 to 0.027) | <0.001 | 43259.51 (42185.78 to 44333.24) | 46508.46 (45660.80 to 47356.12) | 0.234 (0.230 to 0.239) | <0.001 | 3725.62 (3411.35 to 4039.89) | 3891.81 (3647.91 to 4135.72) | 0.141 (0.135 to 0.147) | <0.001 |
| Saudi Arabia | 9558.73 (9504.25 to 9613.22) | 9935.62 (9906.46 to 9964.79) | 0.123 (0.117 to 0.129) | <0.001 | 43044.44 (42928.56 to 43160.31) | 47680.59 (47616.73 to 47744.45) | 0.327 (0.314 to 0.339) | <0.001 | 3690.22 (3656.53 to 3723.91) | 4071.48 (4053.04 to 4089.93) | 0.316 (0.303 to 0.329) | <0.001 |
| Senegal | 9091.45 (9021.20 to 9161.70) | 8925.53 (8881.08 to 8969.98) | -0.062 (-0.073 to -0.052) | <0.001 | 42975.12 (42821.57 to 43128.67) | 44775.27 (44675.11 to 44875.42) | 0.133 (0.124 to 0.143) | <0.001 | 3727.83 (3682.81 to 3772.85) | 3823.75 (3794.61 to 3852.90) | 0.081 (0.071 to 0.090) | <0.001 |
| Serbia | 12772.09 (12729.20 to 12814.97) | 12905.58 (12867.48 to 12943.68) | 0.034 (0.028 to 0.039) | <0.001 | 49216.01 (49131.36 to 49300.66) | 51818.62 (51742.67 to 51894.57) | 0.166 (0.162 to 0.170) | <0.001 | 4741.93 (4715.73 to 4768.14) | 4955.10 (4931.54 to 4978.66) | 0.143 (0.110 to 0.175) | <0.001 |
| Seychelles | 8089.05 (7583.88 to 8594.22) | 7981.54 (7645.68 to 8317.41) | -0.045 (-0.054 to -0.036) | <0.001 | 43631.33 (42458.25 to 44804.41) | 46684.15 (45871.77 to 47496.53) | 0.219 (0.216 to 0.223) | <0.001 | 3749.76 (3405.79 to 4093.72) | 3868.90 (3635.64 to 4102.16) | 0.102 (0.092 to 0.113) | <0.001 |
| Sierra Leone | 9371.44 (9280.69 to 9462.18) | 9178.03 (9112.76 to 9243.30) | -0.067 (-0.073 to -0.060) | <0.001 | 42773.59 (42579.12 to 42968.06) | 44292.49 (44148.21 to 44436.76) | 0.112 (0.107 to 0.117) | <0.001 | 3785.79 (3728.09 to 3843.49) | 3837.73 (3795.46 to 3880.01) | 0.042 (0.031 to 0.053) | <0.001 |
| Singapore | 9808.61 (9721.15 to 9896.06) | 9429.92 (9386.62 to 9473.21) | -0.129 (-0.164 to -0.094) | <0.001 | 54473.75 (54265.60 to 54681.90) | 56202.62 (56096.82 to 56308.42) | 0.103 (0.093 to 0.113) | <0.001 | 4815.54 (4753.80 to 4877.28) | 4932.83 (4901.49 to 4964.17) | 0.078 (0.042 to 0.114) | <0.001 |
| Slovakia | 13091.48 (13029.18 to 13153.79) | 12842.05 (12792.56 to 12891.54) | -0.063 (-0.070 to -0.057) | <0.001 | 51570.24 (51446.62 to 51693.86) | 52321.88 (52222.37 to 52421.39) | 0.047 (0.035 to 0.058) | <0.001 | 4875.05 (4837.04 to 4913.05) | 4868.26 (4837.86 to 4898.66) | -0.004 (-0.018 to 0.011) | 0.596 |
| Slovenia | 12552.08 (12458.14 to 12646.02) | 12469.70 (12396.47 to 12542.93) | -0.023 (-0.035 to -0.012) | <0.001 | 49700.28 (49513.28 to 49887.29) | 50897.75 (50751.10 to 51044.40) | 0.077 (0.068 to 0.086) | <0.001 | 4664.69 (4607.44 to 4721.94) | 4717.03 (4672.18 to 4761.87) | 0.031 (0.011 to 0.051) | 0.002 |
| Solomon Islands | 8621.56 (8294.21 to 8948.92) | 9163.87 (8949.90 to 9377.84) | 0.197 (0.187 to 0.206) | <0.001 | 42057.93 (41330.42 to 42785.44) | 45665.80 (45184.72 to 46146.88) | 0.267 (0.260 to 0.274) | <0.001 | 3499.38 (3291.35 to 3707.42) | 3789.89 (3652.32 to 3927.47) | 0.261 (0.244 to 0.279) | <0.001 |
| Somalia | 9924.64 (9838.89 to 10010.39) | 10017.39 (9962.29 to 10072.48) | 0.033 (0.027 to 0.038) | <0.001 | 42147.51 (41968.80 to 42326.23) | 43138.74 (43023.39 to 43254.08) | 0.077 (0.073 to 0.080) | <0.001 | 3788.47 (3735.28 to 3841.67) | 3869.05 (3834.69 to 3903.41) | 0.069 (0.060 to 0.078) | <0.001 |
| South Africa | 10422.67 (10392.80 to 10452.54) | 10054.79 (10035.50 to 10074.08) | -0.114 (-0.120 to -0.108) | <0.001 | 49186.05 (49120.82 to 49251.28) | 50970.85 (50927.16 to 51014.53) | 0.115 (0.106 to 0.123) | <0.001 | 4388.68 (4369.24 to 4408.13) | 4445.69 (4432.82 to 4458.57) | 0.049 (0.025 to 0.072) | <0.001 |
| South Sudan | 9622.44 (9540.18 to 9704.71) | 9688.37 (9623.52 to 9753.23) | 0.025 (0.009 to 0.041) | 0.002 | 41904.87 (41732.57 to 42077.17) | 42750.94 (42613.33 to 42888.55) | 0.067 (0.062 to 0.072) | <0.001 | 3686.41 (3635.47 to 3737.35) | 3723.02 (3682.69 to 3763.36) | 0.033 (0.028 to 0.039) | <0.001 |
| Spain | 10724.54 (10705.78 to 10743.29) | 10035.98 (10021.87 to 10050.09) | -0.212 (-0.226 to -0.198) | <0.001 | 48457.44 (48417.69 to 48497.18) | 48176.17 (48145.48 to 48206.87) | -0.015 (-0.039 to 0.009) | 0.213 | 4580.34 (4568.09 to 4592.59) | 4174.85 (4165.77 to 4183.92) | -0.274 (-0.396 to -0.152) | <0.001 |
| Sri Lanka | 7908.65 (7872.70 to 7944.59) | 8047.37 (8024.87 to 8069.87) | 0.058 (0.053 to 0.062) | <0.001 | 40587.57 (40506.19 to 40668.95) | 43762.39 (43710.01 to 43814.76) | 0.246 (0.236 to 0.255) | <0.001 | 3461.52 (3437.86 to 3485.17) | 3644.91 (3629.84 to 3659.98) | 0.170 (0.154 to 0.187) | <0.001 |
| Sudan | 9648.81 (9605.71 to 9691.91) | 9859.64 (9830.01 to 9889.28) | 0.073 (0.056 to 0.090) | <0.001 | 42343.04 (42252.43 to 42433.65) | 46800.00 (46735.40 to 46864.61) | 0.323 (0.316 to 0.330) | <0.001 | 3891.21 (3863.87 to 3918.56) | 4272.71 (4253.29 to 4292.13) | 0.302 (0.288 to 0.316) | <0.001 |
| Suriname | 7934.65 (7705.47 to 8163.83) | 8188.51 (8042.49 to 8334.53) | 0.099 (0.092 to 0.106) | <0.001 | 45553.58 (45000.64 to 46106.51) | 48271.28 (47914.83 to 48627.72) | 0.188 (0.182 to 0.194) | <0.001 | 3791.13 (3632.12 to 3950.15) | 3976.83 (3874.73 to 4078.92) | 0.160 (0.130 to 0.189) | <0.001 |
| Sweden | 8573.32 (8540.40 to 8606.25) | 9668.76 (9638.89 to 9698.63) | 0.388 (0.359 to 0.416) | <0.001 | 43548.03 (43473.39 to 43622.66) | 47446.10 (47380.25 to 47511.96) | 0.280 (0.206 to 0.355) | <0.001 | 3856.50 (3834.25 to 3878.74) | 4303.27 (4283.32 to 4323.22) | 0.354 (0.283 to 0.426) | <0.001 |
| Switzerland | 11362.42 (11316.93 to 11407.91) | 11203.67 (11169.21 to 11238.13) | -0.046 (-0.058 to -0.034) | <0.001 | 49743.75 (49649.06 to 49838.43) | 51190.84 (51117.66 to 51264.03) | 0.092 (0.077 to 0.106) | <0.001 | 4637.46 (4608.45 to 4666.46) | 4768.64 (4746.20 to 4791.09) | 0.088 (0.057 to 0.119) | <0.001 |
| Syrian Arab Republic | 9895.09 (9837.72 to 9952.46) | 10026.04 (9991.17 to 10060.91) | 0.042 (0.038 to 0.046) | <0.001 | 45209.93 (45087.33 to 45332.53) | 47145.07 (47069.51 to 47220.63) | 0.134 (0.122 to 0.146) | <0.001 | 4132.43 (4095.50 to 4169.35) | 4167.56 (4145.23 to 4189.88) | 0.022 (-0.001 to 0.045) | 0.061 |
| Taiwan (Province of China) | 9623.32 (9591.15 to 9655.48) | 10825.80 (10804.73 to 10846.87) | 0.384 (0.307 to 0.462) | <0.001 | 48104.12 (48032.15 to 48176.09) | 55054.50 (55007.07 to 55101.93) | 0.438 (0.403 to 0.474) | <0.001 | 4272.00 (4250.62 to 4293.39) | 5186.80 (5172.24 to 5201.37) | 0.627 (0.607 to 0.646) | <0.001 |
| Tajikistan | 10809.49 (10727.72 to 10891.27) | 10684.07 (10629.48 to 10738.65) | -0.041 (-0.051 to -0.031) | <0.001 | 43737.89 (43572.47 to 43903.32) | 45260.72 (45147.40 to 45374.05) | 0.110 (0.102 to 0.118) | <0.001 | 3975.57 (3925.81 to 4025.34) | 4048.22 (4014.45 to 4081.99) | 0.056 (0.040 to 0.071) | <0.001 |
| Thailand | 7447.43 (7428.44 to 7466.42) | 7783.30 (7772.32 to 7794.27) | 0.144 (0.137 to 0.151) | <0.001 | 42463.59 (42418.41 to 42508.77) | 46373.01 (46346.26 to 46399.77) | 0.287 (0.280 to 0.295) | <0.001 | 3758.73 (3745.34 to 3772.12) | 4060.73 (4052.81 to 4068.64) | 0.253 (0.229 to 0.276) | <0.001 |
| Timor-Leste | 7703.39 (7484.34 to 7922.44) | 7637.59 (7512.04 to 7763.15) | -0.028 (-0.036 to -0.019) | <0.001 | 38096.48 (37609.86 to 38583.11) | 41441.43 (41148.84 to 41734.02) | 0.273 (0.263 to 0.283) | <0.001 | 3274.61 (3133.16 to 3416.07) | 3508.60 (3423.71 to 3593.49) | 0.225 (0.213 to 0.236) | <0.001 |
| Togo | 9465.46 (9349.35 to 9581.57) | 9334.63 (9270.73 to 9398.52) | -0.050 (-0.066 to -0.034) | <0.001 | 43429.28 (43178.81 to 43679.75) | 45430.31 (45287.88 to 45572.75) | 0.146 (0.138 to 0.154) | <0.001 | 3861.30 (3787.07 to 3935.52) | 3985.91 (3943.99 to 4027.83) | 0.108 (0.096 to 0.120) | <0.001 |
| Tokelau | 9017.49 (5596.47 to 12438.52) | 9182.43 (5889.65 to 12475.21) | 0.061 (0.039 to 0.083) | <0.001 | 45696.49 (38003.18 to 53389.81) | 49427.36 (41789.00 to 57065.71) | 0.258 (0.240 to 0.277) | <0.001 | 3895.53 (1651.04 to 6140.03) | 4114.72 (1911.37 to 6318.07) | 0.176 (0.142 to 0.210) | <0.001 |
| Tonga | 9223.88 (8692.97 to 9754.78) | 9279.60 (8833.53 to 9725.68) | 0.019 (0.012 to 0.027) | <0.001 | 46791.60 (45591.59 to 47991.61) | 49397.09 (48365.48 to 50428.70) | 0.175 (0.166 to 0.184) | <0.001 | 4120.65 (3765.61 to 4475.68) | 4268.32 (3965.36 to 4571.27) | 0.114 (0.100 to 0.128) | <0.001 |
| Trinidad and Tobago | 8066.38 (7935.30 to 8197.47) | 8176.63 (8091.41 to 8261.85) | 0.043 (0.038 to 0.049) | <0.001 | 46425.88 (46110.62 to 46741.15) | 49055.18 (48846.62 to 49263.75) | 0.180 (0.175 to 0.185) | <0.001 | 3984.45 (3891.94 to 4076.97) | 4150.13 (4089.41 to 4210.85) | 0.142 (0.103 to 0.181) | <0.001 |
| Tunisia | 9844.28 (9786.42 to 9902.14) | 10090.25 (10054.35 to 10126.16) | 0.081 (0.067 to 0.094) | <0.001 | 45256.79 (45132.45 to 45381.13) | 49639.04 (49559.37 to 49718.72) | 0.299 (0.295 to 0.302) | <0.001 | 4124.79 (4087.41 to 4162.17) | 4516.52 (4492.53 to 4540.51) | 0.294 (0.286 to 0.302) | <0.001 |
| Turkmenistan | 11091.65 (10992.00 to 11191.29) | 11149.41 (11081.49 to 11217.32) | 0.018 (0.015 to 0.021) | <0.001 | 46014.00 (45809.61 to 46218.39) | 49542.05 (49398.01 to 49686.09) | 0.239 (0.232 to 0.246) | <0.001 | 4171.44 (4110.03 to 4232.84) | 4416.83 (4373.85 to 4459.82) | 0.188 (0.176 to 0.200) | <0.001 |
| Tuvalu | 9148.52 (7615.29 to 10681.75) | 9249.62 (8024.96 to 10474.29) | 0.032 (0.022 to 0.042) | <0.001 | 45651.40 (42217.07 to 49085.73) | 48838.95 (46017.02 to 51660.88) | 0.218 (0.208 to 0.227) | <0.001 | 3927.34 (2924.16 to 4930.53) | 4103.46 (3288.02 to 4918.90) | 0.146 (0.132 to 0.159) | <0.001 |
| T眉rkiye | 10261.46 (10238.90 to 10284.02) | 10340.84 (10327.10 to 10354.58) | 0.028 (0.006 to 0.051) | 0.012 | 46765.46 (46717.34 to 46813.58) | 50898.93 (50868.45 to 50929.40) | 0.273 (0.264 to 0.282) | <0.001 | 4452.84 (4438.00 to 4467.67) | 4761.17 (4751.86 to 4770.48) | 0.212 (0.195 to 0.230) | <0.001 |
| Uganda | 10091.56 (10039.09 to 10144.03) | 10037.31 (10002.93 to 10071.69) | -0.010 (-0.016 to -0.003) | 0.007 | 42578.30 (42469.97 to 42686.63) | 44882.03 (44808.84 to 44955.22) | 0.171 (0.161 to 0.180) | <0.001 | 3833.11 (3800.75 to 3865.47) | 4038.32 (4016.45 to 4060.20) | 0.170 (0.160 to 0.178) | <0.001 |
| Ukraine | 14083.29 (14065.12 to 14101.46) | 13915.70 (13897.84 to 13933.55) | -0.037 (-0.050 to -0.024) | <0.001 | 53921.17 (53885.50 to 53956.84) | 54982.74 (54947.36 to 55018.12) | 0.063 (0.060 to 0.067) | <0.001 | 5181.34 (5170.29 to 5192.40) | 5229.01 (5218.10 to 5239.93) | 0.029 (0.009 to 0.049) | 0.004 |
| United Arab Emirates | 9094.79 (8899.83 to 9289.75) | 9280.51 (9227.02 to 9334.00) | 0.065 (0.052 to 0.078) | <0.001 | 44133.81 (43703.90 to 44563.72) | 47136.00 (47015.58 to 47256.42) | 0.216 (0.203 to 0.228) | <0.001 | 3831.05 (3705.08 to 3957.02) | 4053.84 (4018.80 to 4088.89) | 0.181 (0.131 to 0.230) | <0.001 |
| United Kingdom | 10654.01 (10638.98 to 10669.04) | 11573.16 (11560.11 to 11586.22) | 0.262 (0.225 to 0.298) | <0.001 | 51513.17 (51480.46 to 51545.88) | 53990.88 (53962.87 to 54018.89) | 0.153 (0.119 to 0.187) | <0.001 | 4864.58 (4854.48 to 4874.67) | 5115.63 (5106.98 to 5124.27) | 0.154 (0.126 to 0.182) | <0.001 |
| United Republic of Tanzania | 10040.83 (10000.77 to 10080.89) | 9951.21 (9925.26 to 9977.16) | -0.030 (-0.038 to -0.023) | <0.001 | 44261.94 (44177.31 to 44346.58) | 45513.09 (45457.23 to 45568.95) | 0.091 (0.081 to 0.101) | <0.001 | 3963.33 (3938.11 to 3988.56) | 4050.96 (4034.36 to 4067.56) | 0.072 (0.067 to 0.077) | <0.001 |
| United States Virgin Islands | 8075.89 (7672.29 to 8479.49) | 8217.43 (7934.17 to 8500.69) | 0.053 (0.040 to 0.066) | <0.001 | 47154.95 (46170.87 to 48139.03) | 49851.93 (49158.35 to 50545.52) | 0.180 (0.172 to 0.189) | <0.001 | 3942.31 (3658.36 to 4226.25) | 4098.24 (3898.81 to 4297.67) | 0.122 (0.109 to 0.136) | <0.001 |
| United States of America | 11784.42 (11776.03 to 11792.81) | 11848.49 (11842.40 to 11854.59) | 0.012 (-0.006 to 0.029) | 0.184 | 58181.33 (58162.83 to 58199.84) | 62166.01 (62152.12 to 62179.91) | 0.223 (0.152 to 0.295) | <0.001 | 5529.55 (5523.82 to 5535.27) | 5899.24 (5894.95 to 5903.54) | 0.212 (0.192 to 0.231) | <0.001 |
| Uruguay | 10291.81 (10223.33 to 10360.29) | 10994.13 (10932.61 to 11055.65) | 0.211 (0.102 to 0.320) | <0.001 | 53805.08 (53648.83 to 53961.33) | 58072.07 (57931.54 to 58212.60) | 0.245 (0.227 to 0.263) | <0.001 | 5091.70 (5043.60 to 5139.80) | 5651.46 (5607.52 to 5695.41) | 0.331 (0.262 to 0.401) | <0.001 |
| Uzbekistan | 11157.59 (11116.78 to 11198.40) | 11272.68 (11246.07 to 11299.30) | 0.034 (0.023 to 0.045) | <0.001 | 46469.20 (46385.57 to 46552.83) | 49394.05 (49337.93 to 49450.16) | 0.198 (0.193 to 0.203) | <0.001 | 4231.76 (4206.56 to 4256.97) | 4463.77 (4446.92 to 4480.63) | 0.173 (0.149 to 0.197) | <0.001 |
| Vanuatu | 9003.22 (8498.16 to 9508.27) | 9314.81 (9013.24 to 9616.38) | 0.109 (0.096 to 0.122) | <0.001 | 43469.00 (42353.74 to 44584.25) | 46341.79 (45665.39 to 47018.19) | 0.206 (0.201 to 0.210) | <0.001 | 3792.33 (3464.74 to 4119.93) | 4025.94 (3827.55 to 4224.33) | 0.190 (0.179 to 0.202) | <0.001 |
| Venezuela (Bolivarian Republic of) | 8463.79 (8424.50 to 8503.09) | 8463.68 (8441.84 to 8485.51) | -0.002 (-0.021 to 0.018) | 0.869 | 49438.30 (49342.87 to 49533.74) | 51803.09 (51748.96 to 51857.23) | 0.151 (0.144 to 0.158) | <0.001 | 4496.96 (4468.26 to 4525.65) | 4706.39 (4690.08 to 4722.70) | 0.145 (0.115 to 0.176) | <0.001 |
| Viet Nam | 8187.84 (8168.93 to 8206.75) | 8137.48 (8125.76 to 8149.20) | -0.022 (-0.030 to -0.015) | <0.001 | 39164.51 (39123.24 to 39205.79) | 42111.78 (42085.18 to 42138.38) | 0.236 (0.229 to 0.243) | <0.001 | 3481.05 (3468.76 to 3493.34) | 3695.04 (3687.20 to 3702.88) | 0.196 (0.189 to 0.204) | <0.001 |
| Yemen | 9824.07 (9764.47 to 9883.68) | 9837.29 (9802.05 to 9872.53) | 0.005 (-0.007 to 0.017) | 0.407 | 42073.54 (41949.88 to 42197.19) | 45029.75 (44954.31 to 45105.19) | 0.219 (0.208 to 0.231) | <0.001 | 3884.41 (3847.01 to 3921.80) | 4091.47 (4068.85 to 4114.09) | 0.165 (0.158 to 0.172) | <0.001 |
| Zambia | 9129.64 (9054.83 to 9204.45) | 9599.33 (9550.05 to 9648.60) | 0.161 (0.153 to 0.169) | <0.001 | 42327.94 (42165.81 to 42490.07) | 44871.64 (44764.27 to 44979.01) | 0.193 (0.174 to 0.213) | <0.001 | 3634.36 (3587.07 to 3681.66) | 3907.98 (3876.42 to 3939.54) | 0.237 (0.228 to 0.246) | <0.001 |
| Zimbabwe | 9717.33 (9652.81 to 9781.86) | 10031.79 (9981.41 to 10082.16) | 0.103 (0.100 to 0.105) | <0.001 | 44225.13 (44086.69 to 44363.56) | 46122.16 (46013.31 to 46231.02) | 0.136 (0.129 to 0.143) | <0.001 | 4000.79 (3959.31 to 4042.26) | 4215.23 (4182.48 to 4247.98) | 0.172 (0.160 to 0.184) | <0.001 |
| AAPC, average annual percentage changes; DALYs, disability-adjusted life-years; MSK disorders, musculoskeletal disorders. | | | | | | | | | | | | |

| **Supplementary Table 7** Gender difference in global age standardized incidence, prevalence and DALY rates (per 100000 population) and their average annual percent changes for MSK disorders among adults aged 50 and over by SDI, 1990-2021 | | | | | | |
| --- | --- | --- | --- | --- | --- | --- |
| Location name | Measure name | Gender | Age standardized rate | | AAPC (%) | p value |
|  |  |  | 1990 | 2021 |  |  |
| Global | Incidence rate | Women | 12120.51 (12117.36 to 12123.67) | 11530.12 (11528.02 to 11532.22) | -0.161 (-0.173 to -0.149) | <0.001 |
| Global | Incidence rate | Men | 8533.35 (8530.53 to 8536.18) | 8054.65 (8052.81 to 8056.49) | -0.184 (-0.194 to -0.174) | <0.001 |
| High SDI | Incidence rate | Women | 12786.86 (12780.72 to 12792.99) | 12694.93 (12690.26 to 12699.59) | -0.036 (-0.077 to 0.004) | 0.08 |
| High SDI | Incidence rate | Men | 9820.43 (9814.41 to 9826.45) | 9657.88 (9653.57 to 9662.20) | -0.042 (-0.062 to -0.022) | <0.001 |
| High-middle SDI | Incidence rate | Women | 12849.32 (12843.03 to 12855.62) | 11793.28 (11788.91 to 11797.65) | -0.274 (-0.300 to -0.247) | <0.001 |
| High-middle SDI | Incidence rate | Men | 9219.05 (9213.09 to 9225.01) | 8331.37 (8327.43 to 8335.31) | -0.321 (-0.338 to -0.303) | <0.001 |
| Middle SDI | Incidence rate | Women | 11241.07 (11235.02 to 11247.13) | 10735.32 (10731.75 to 10738.89) | -0.140 (-0.153 to -0.127) | <0.001 |
| Middle SDI | Incidence rate | Men | 7455.66 (7450.62 to 7460.70) | 7177.99 (7174.93 to 7181.04) | -0.120 (-0.136 to -0.104) | <0.001 |
| Low-middle SDI | Incidence rate | Women | 11503.81 (11495.66 to 11511.97) | 11308.08 (11302.98 to 11313.17) | -0.055 (-0.103 to -0.007) | 0.025 |
| Low-middle SDI | Incidence rate | Men | 7570.44 (7563.93 to 7576.94) | 7402.23 (7397.93 to 7406.52) | -0.074 (-0.091 to -0.057) | <0.001 |
| Low SDI | Incidence rate | Women | 11435.20 (11421.84 to 11448.57) | 11134.34 (11125.69 to 11142.98) | -0.086 (-0.118 to -0.054) | <0.001 |
| Low SDI | Incidence rate | Men | 8226.42 (8215.28 to 8237.55) | 7924.48 (7917.03 to 7931.93) | -0.121 (-0.129 to -0.114) | <0.001 |
| Global | Prevalence rate | Women | 55351.37 (55344.61 to 55358.13) | 57301.79 (57297.12 to 57306.45) | 0.110 (0.095 to 0.125) | <0.001 |
| Global | Prevalence rate | Men | 41642.53 (41636.25 to 41648.80) | 43779.11 (43774.82 to 43783.40) | 0.162 (0.154 to 0.170) | <0.001 |
| High SDI | Prevalence rate | Women | 59308.77 (59295.62 to 59321.92) | 62070.85 (62060.62 to 62081.08) | 0.140 (0.109 to 0.171) | <0.001 |
| High SDI | Prevalence rate | Men | 46942.57 (46929.38 to 46955.76) | 50042.82 (50033.06 to 50052.59) | 0.214 (0.184 to 0.244) | <0.001 |
| High-middle SDI | Prevalence rate | Women | 55324.47 (55311.40 to 55337.54) | 56377.32 (56367.79 to 56386.84) | 0.063 (0.052 to 0.073) | <0.001 |
| High-middle SDI | Prevalence rate | Men | 41289.20 (41276.50 to 41301.91) | 42772.70 (42763.74 to 42781.66) | 0.115 (0.101 to 0.130) | <0.001 |
| Middle SDI | Prevalence rate | Women | 53098.37 (53085.18 to 53111.57) | 55780.88 (55772.72 to 55789.03) | 0.162 (0.153 to 0.171) | <0.001 |
| Middle SDI | Prevalence rate | Men | 39422.39 (39410.77 to 39434.01) | 42103.67 (42096.26 to 42111.08) | 0.212 (0.201 to 0.224) | <0.001 |
| Low-middle SDI | Prevalence rate | Women | 53353.13 (53335.55 to 53370.71) | 56634.80 (56623.35 to 56646.24) | 0.192 (0.182 to 0.202) | <0.001 |
| Low-middle SDI | Prevalence rate | Men | 38732.30 (38717.52 to 38747.08) | 41432.64 (41422.45 to 41442.83) | 0.213 (0.201 to 0.224) | <0.001 |
| Low SDI | Prevalence rate | Women | 50209.34 (50181.20 to 50237.49) | 52017.29 (51998.52 to 52036.07) | 0.114 (0.106 to 0.122) | <0.001 |
| Low SDI | Prevalence rate | Men | 38373.88 (38349.73 to 38398.03) | 39965.31 (39948.49 to 39982.13) | 0.128 (0.123 to 0.133) | <0.001 |
| Global | DALY rate | Women | 5360.38 (5358.28 to 5362.47) | 5450.30 (5448.87 to 5451.73) | 0.055 (0.040 to 0.069) | <0.001 |
| Global | DALY rate | Men | 3546.00 (3544.18 to 3547.82) | 3651.83 (3650.59 to 3653.06) | 0.094 (0.080 to 0.109) | <0.001 |
| High SDI | DALY rate | Women | 5889.05 (5884.90 to 5893.21) | 6106.29 (6103.06 to 6109.53) | 0.113 (0.081 to 0.145) | <0.001 |
| High SDI | DALY rate | Men | 4120.60 (4116.70 to 4124.50) | 4374.62 (4371.74 to 4377.50) | 0.200 (0.178 to 0.222) | <0.001 |
| High-middle SDI | DALY rate | Women | 5336.14 (5332.08 to 5340.20) | 5268.57 (5265.65 to 5271.49) | -0.037 (-0.048 to -0.026) | <0.001 |
| High-middle SDI | DALY rate | Men | 3485.03 (3481.35 to 3488.72) | 3482.27 (3479.73 to 3484.82) | -0.002 (-0.011 to 0.007) | 0.721 |
| Middle SDI | DALY rate | Women | 5004.10 (5000.06 to 5008.13) | 5143.98 (5141.51 to 5146.45) | 0.091 (0.074 to 0.108) | <0.001 |
| Middle SDI | DALY rate | Men | 3264.11 (3260.78 to 3267.44) | 3398.79 (3396.70 to 3400.89) | 0.130 (0.116 to 0.144) | <0.001 |
| Low-middle SDI | DALY rate | Women | 5224.94 (5219.46 to 5230.43) | 5556.41 (5552.82 to 5560.00) | 0.199 (0.177 to 0.221) | <0.001 |
| Low-middle SDI | DALY rate | Men | 3326.73 (3322.42 to 3331.04) | 3526.81 (3523.85 to 3529.77) | 0.182 (0.146 to 0.219) | <0.001 |
| Low SDI | DALY rate | Women | 4814.86 (4806.18 to 4823.55) | 4963.26 (4957.47 to 4969.04) | 0.096 (0.079 to 0.114) | <0.001 |
| Low SDI | DALY rate | Men | 3275.30 (3268.28 to 3282.32) | 3367.05 (3362.19 to 3371.91) | 0.085 (0.068 to 0.102) | <0.001 |
| AAPC, average annual percent changes; DALYs, disability-adjusted life-years; MSK disorders, musculoskeletal disorders; SDI, Socio-demographic Index. | | | | | | |

| **Supplementary Table 8** Global age-specific incidence, prevalence and DALY rates (per 100000 population) and their average annual percent changes for MSK disorders among adults aged 50 and over, 1990-2021 | | | | | |
| --- | --- | --- | --- | --- | --- |
| Location name | Age group | Age standardized rate | | AAPC (%) | p value |
|  |  | 1990 | 2021 |  |  |
| Incidence rate | 50-54 | 8856.51 | 8392.5 | -0.173 (-0.194 to -0.151) | <0.001 |
| Incidence rate | 55-59 | 9600.14 | 9140.71 | -0.146 (-0.189 to -0.103) | <0.001 |
| Incidence rate | 60-64 | 10367 | 9874.8 | -0.155 (-0.179 to -0.132) | <0.001 |
| Incidence rate | 65-69 | 11087.87 | 10393.99 | -0.208 (-0.230 to -0.186) | <0.001 |
| Incidence rate | 70-74 | 12160.55 | 11413.56 | -0.205 (-0.224 to -0.185) | <0.001 |
| Incidence rate | 75-79 | 12708.61 | 11671.65 | -0.276 (-0.296 to -0.256) | <0.001 |
| Incidence rate | 80-84 | 12260.29 | 11654.57 | -0.165 (-0.177 to -0.154) | <0.001 |
| Incidence rate | 85-89 | 11152.17 | 10797.4 | -0.105 (-0.117 to -0.092) | <0.001 |
| Incidence rate | 90-94 | 9876.62 | 9821.96 | -0.018 (-0.046 to 0.011) | 0.221 |
| Incidence rate | 95+ | 9021.29 | 9168.3 | 0.051 (0.032 to 0.069) | <0.001 |
| Prevalence rate | 50-54 | 38200.43 | 39933.96 | 0.142 (0.127 to 0.157) | <0.001 |
| Prevalence rate | 55-59 | 43890.63 | 46112.58 | 0.162 (0.152 to 0.173) | <0.001 |
| Prevalence rate | 60-64 | 49006.64 | 51505.06 | 0.163 (0.145 to 0.180) | <0.001 |
| Prevalence rate | 65-69 | 53406.73 | 55475.9 | 0.125 (0.109 to 0.140) | <0.001 |
| Prevalence rate | 70-74 | 57207.56 | 59218.82 | 0.115 (0.095 to 0.134) | <0.001 |
| Prevalence rate | 75-79 | 60264.55 | 61111.57 | 0.046 (0.041 to 0.051) | <0.001 |
| Prevalence rate | 80-84 | 60999.52 | 62038.21 | 0.053 (0.045 to 0.062) | <0.001 |
| Prevalence rate | 85-89 | 60868.27 | 62129.82 | 0.063 (0.045 to 0.080) | <0.001 |
| Prevalence rate | 90-94 | 59499.13 | 61678.6 | 0.111 (0.092 to 0.130) | <0.001 |
| Prevalence rate | 95+ | 58575.17 | 61507.19 | 0.148 (0.110 to 0.185) | <0.001 |
| DALY rate | 50-54 | 3650.71 | 3719.5 | 0.059 (0.044 to 0.074) | <0.001 |
| DALY rate | 55-59 | 4108.69 | 4215.91 | 0.084 (0.066 to 0.102) | <0.001 |
| DALY rate | 60-64 | 4539.82 | 4691.32 | 0.108 (0.086 to 0.130) | <0.001 |
| DALY rate | 65-69 | 4929.07 | 5020.06 | 0.062 (0.041 to 0.083) | <0.001 |
| DALY rate | 70-74 | 5287.86 | 5380 | 0.059 (0.027 to 0.092) | <0.001 |
| DALY rate | 75-79 | 5507.65 | 5451.42 | -0.032 (-0.051 to -0.013) | 0.001 |
| DALY rate | 80-84 | 5326.34 | 5314.16 | -0.010 (-0.029 to 0.009) | 0.29 |
| DALY rate | 85-89 | 5064.91 | 5064.71 | -0.004 (-0.034 to 0.027) | 0.817 |
| DALY rate | 90-94 | 4634.09 | 4777.52 | 0.093 (0.048 to 0.139) | <0.001 |
| DALY rate | 95+ | 4332.06 | 4590.19 | 0.181 (0.123 to 0.238) | <0.001 |
| AAPC, average annual percent changes; DALYs, disability-adjusted life-years; MSK disorders, musculoskeletal disorders. | | | | | |

| **Supplementary Table 9** Global DALYs attributable to main risk factors and their proportions to overall DALYs for MSK disorders among adults aged 50 and over by SDI and geographic regions, 1990-2021 | | | | | |
| --- | --- | --- | --- | --- | --- |
| Risk factors | Location name | DALYs | Proportions (%) | DALYs | Proportions (%) |
|  |  | 1990 | | 2021 | |
| Occupational ergonomic factors | Global | 3646194 | 9.23 | 6464067 | 7.31 |
| Occupational ergonomic factors | High SDI | 558338 | 4.65 | 972915 | 4.31 |
| Occupational ergonomic factors | High-middle SDI | 839489 | 8.32 | 1250286 | 6.34 |
| Occupational ergonomic factors | Middle SDI | 1139181 | 11.88 | 2098728 | 7.86 |
| Occupational ergonomic factors | Low-middle SDI | 717500 | 12.44 | 1449170 | 9.86 |
| Occupational ergonomic factors | Low SDI | 387924 | 19.2 | 687766 | 14.69 |
| Occupational ergonomic factors | Central Asia | 63979 | 14.31 | 108199 | 13.04 |
| Occupational ergonomic factors | Central Europe | 164806 | 10.05 | 170894 | 7.45 |
| Occupational ergonomic factors | Eastern Europe | 195685 | 6.09 | 203810 | 5.23 |
| Occupational ergonomic factors | Australasia | 11923 | 4.44 | 29477 | 4.82 |
| Occupational ergonomic factors | High-income Asia Pacific | 158140 | 6.33 | 223173 | 4.55 |
| Occupational ergonomic factors | High-income North America | 157047 | 4 | 308708 | 3.84 |
| Occupational ergonomic factors | Southern Latin America | 22352 | 4.04 | 48195 | 4.52 |
| Occupational ergonomic factors | Western Europe | 212949 | 3.73 | 381159 | 4.33 |
| Occupational ergonomic factors | Andean Latin America | 10606 | 6.03 | 42309 | 7.64 |
| Occupational ergonomic factors | Caribbean | 9410 | 4.58 | 23027 | 4.93 |
| Occupational ergonomic factors | Central Latin America | 47934 | 5.78 | 144520 | 5.21 |
| Occupational ergonomic factors | Tropical Latin America | 75395 | 7.59 | 192703 | 6.6 |
| Occupational ergonomic factors | North Africa and Middle East | 111886 | 7.13 | 248282 | 5.35 |
| Occupational ergonomic factors | South Asia | 648630 | 11.5 | 1199766 | 7.67 |
| Occupational ergonomic factors | East Asia | 1145626 | 14.5 | 1817849 | 8.81 |
| Occupational ergonomic factors | Oceania | 1530 | 6.34 | 5547 | 8.61 |
| Occupational ergonomic factors | Southeast Asia | 240986 | 11.7 | 603974 | 10.2 |
| Occupational ergonomic factors | Central Sub-Saharan Africa | 44358 | 21.64 | 85617 | 16.74 |
| Occupational ergonomic factors | Eastern Sub-Saharan Africa | 162486 | 25.33 | 305819 | 20.44 |
| Occupational ergonomic factors | Southern Sub-Saharan Africa | 18508 | 7.35 | 36522 | 6.49 |
| Occupational ergonomic factors | Western Sub-Saharan Africa | 141954 | 18.95 | 284518 | 15.79 |
| Smoking | Global | 3509067 | 8.88 | 5316226 | 6.01 |
| Smoking | High SDI | 1310963 | 10.93 | 1678250 | 7.44 |
| Smoking | High-middle SDI | 972431 | 9.64 | 1450937 | 7.36 |
| Smoking | Middle SDI | 733746 | 7.65 | 1316541 | 4.93 |
| Smoking | Low-middle SDI | 378830 | 6.57 | 682781 | 4.65 |
| Smoking | Low SDI | 107726 | 5.33 | 181236 | 3.87 |
| Smoking | Central Asia | 31594 | 7.07 | 56015 | 6.75 |
| Smoking | Central Europe | 246080 | 15.01 | 278274 | 12.13 |
| Smoking | Eastern Europe | 252624 | 7.86 | 308286 | 7.9 |
| Smoking | Australasia | 27581 | 10.26 | 38938 | 6.37 |
| Smoking | High-income Asia Pacific | 236661 | 9.47 | 259372 | 5.29 |
| Smoking | High-income North America | 425319 | 10.82 | 585496 | 7.29 |
| Smoking | Southern Latin America | 48125 | 8.69 | 74885 | 7.03 |
| Smoking | Western Europe | 649819 | 11.37 | 775425 | 8.81 |
| Smoking | Andean Latin America | 6497 | 3.69 | 15976 | 2.89 |
| Smoking | Caribbean | 14257 | 6.94 | 24166 | 5.17 |
| Smoking | Central Latin America | 43584 | 5.25 | 84490 | 3.05 |
| Smoking | Tropical Latin America | 100576 | 10.13 | 193559 | 6.63 |
| Smoking | North Africa and Middle East | 119309 | 7.61 | 280767 | 6.06 |
| Smoking | South Asia | 358410 | 6.35 | 606994 | 3.88 |
| Smoking | East Asia | 730179 | 9.24 | 1301775 | 6.31 |
| Smoking | Oceania | 1927 | 7.99 | 4557 | 7.07 |
| Smoking | Southeast Asia | 134672 | 6.54 | 286788 | 4.85 |
| Smoking | Central Sub-Saharan Africa | 7726 | 3.77 | 16962 | 3.32 |
| Smoking | Eastern Sub-Saharan Africa | 31895 | 4.97 | 57178 | 3.82 |
| Smoking | Southern Sub-Saharan Africa | 20746 | 8.24 | 26563 | 4.72 |
| Smoking | Western Sub-Saharan Africa | 21483 | 2.87 | 39760 | 2.21 |
| High body-mass index | Global | 3060456 | 7.75 | 9137349 | 10.33 |
| High body-mass index | High SDI | 1238837 | 10.32 | 2913379 | 12.91 |
| High body-mass index | High-middle SDI | 993987 | 9.85 | 2466344 | 12.52 |
| High body-mass index | Middle SDI | 524162 | 5.47 | 2455657 | 9.19 |
| High body-mass index | Low-middle SDI | 225584 | 3.91 | 1021187 | 6.95 |
| High body-mass index | Low SDI | 72824 | 3.6 | 270798 | 5.78 |
| High body-mass index | Central Asia | 54164 | 12.11 | 116176 | 14 |
| High body-mass index | Central Europe | 224210 | 13.67 | 372109 | 16.21 |
| High body-mass index | Eastern Europe | 421560 | 13.12 | 653454 | 16.75 |
| High body-mass index | Australasia | 31279 | 11.64 | 98348 | 16.09 |
| High body-mass index | High-income Asia Pacific | 132344 | 5.3 | 335864 | 6.85 |
| High body-mass index | High-income North America | 497413 | 12.66 | 1254381 | 15.61 |
| High body-mass index | Southern Latin America | 59776 | 10.8 | 150710 | 14.14 |
| High body-mass index | Western Europe | 620032 | 10.85 | 1201572 | 13.66 |
| High body-mass index | Andean Latin America | 15054 | 8.55 | 66354 | 11.99 |
| High body-mass index | Caribbean | 17930 | 8.73 | 56394 | 12.06 |
| High body-mass index | Central Latin America | 77343 | 9.33 | 333642 | 12.03 |
| High body-mass index | Tropical Latin America | 86062 | 8.67 | 361510 | 12.38 |
| High body-mass index | North Africa and Middle East | 160702 | 10.24 | 695803 | 15.01 |
| High body-mass index | South Asia | 128530 | 2.28 | 756450 | 4.84 |
| High body-mass index | East Asia | 359043 | 4.54 | 1920123 | 9.3 |
| High body-mass index | Oceania | 2252 | 9.33 | 7517 | 11.67 |
| High body-mass index | Southeast Asia | 64460 | 3.13 | 355209 | 6 |
| High body-mass index | Central Sub-Saharan Africa | 8864 | 4.32 | 43006 | 8.41 |
| High body-mass index | Eastern Sub-Saharan Africa | 26243 | 4.09 | 103816 | 6.94 |
| High body-mass index | Southern Sub-Saharan Africa | 27461 | 10.91 | 82700 | 14.71 |
| High body-mass index | Western Sub-Saharan Africa | 45732 | 6.1 | 172212 | 9.56 |
| kidney dysfunction | Global | 73657 | 0.19 | 191383 | 0.22 |
| kidney dysfunction | High SDI | 31321 | 0.26 | 85892 | 0.38 |
| kidney dysfunction | High-middle SDI | 16072 | 0.16 | 35651 | 0.18 |
| kidney dysfunction | Middle SDI | 15693 | 0.16 | 44215 | 0.17 |
| kidney dysfunction | Low-middle SDI | 7757 | 0.13 | 19429 | 0.13 |
| kidney dysfunction | Low SDI | 2762 | 0.14 | 6088 | 0.13 |
| kidney dysfunction | Central Asia | 879 | 0.2 | 1715 | 0.21 |
| kidney dysfunction | Central Europe | 1667 | 0.1 | 2943 | 0.13 |
| kidney dysfunction | Eastern Europe | 3607 | 0.11 | 5462 | 0.14 |
| kidney dysfunction | Australasia | 986 | 0.37 | 3018 | 0.49 |
| kidney dysfunction | High-income Asia Pacific | 5279 | 0.21 | 15078 | 0.31 |
| kidney dysfunction | High-income North America | 14600 | 0.37 | 46676 | 0.58 |
| kidney dysfunction | Southern Latin America | 928 | 0.17 | 2191 | 0.21 |
| kidney dysfunction | Western Europe | 12076 | 0.21 | 22452 | 0.26 |
| kidney dysfunction | Andean Latin America | 101 | 0.06 | 394 | 0.07 |
| kidney dysfunction | Caribbean | 141 | 0.07 | 383 | 0.08 |
| kidney dysfunction | Central Latin America | 413 | 0.05 | 1668 | 0.06 |
| kidney dysfunction | Tropical Latin America | 653 | 0.07 | 2535 | 0.09 |
| kidney dysfunction | North Africa and Middle East | 2361 | 0.15 | 7326 | 0.16 |
| kidney dysfunction | South Asia | 7278 | 0.13 | 19262 | 0.12 |
| kidney dysfunction | East Asia | 15616 | 0.2 | 40479 | 0.2 |
| kidney dysfunction | Oceania | 48 | 0.2 | 133 | 0.21 |
| kidney dysfunction | Southeast Asia | 3931 | 0.19 | 12874 | 0.22 |
| kidney dysfunction | Central Sub-Saharan Africa | 419 | 0.2 | 1025 | 0.2 |
| kidney dysfunction | Eastern Sub-Saharan Africa | 549 | 0.09 | 1208 | 0.08 |
| kidney dysfunction | Southern Sub-Saharan Africa | 590 | 0.23 | 1374 | 0.24 |
| kidney dysfunction | Western Sub-Saharan Africa | 1534 | 0.2 | 3184 | 0.18 |
| DALYs, disability-adjusted life-years; MSK disorders, musculoskeletal disorders; SDI, Socio-demographic Index. | | | | | |

| **Supplementary Table 10** DALYs attributable to main risk factors and their proportions to overall DALYs for MSK disorders among adults aged 50 and over across 204 countries and territories, 1990-2021 | | | | | |
| --- | --- | --- | --- | --- | --- |
| Risk factors | Location name | DALYs | Proportions (%) | DALYs | Proportions (%) |
|  |  | 1990 | | 2021 | |
| High body-mass index | Afghanistan | 56968 | 8.34 | 82785 | 10.44 |
| High body-mass index | Albania | 21185 | 12.37 | 46857 | 15.49 |
| High body-mass index | Algeria | 112623 | 8.75 | 362960 | 13.12 |
| High body-mass index | American Samoa | 206 | 16.66 | 477 | 19.54 |
| High body-mass index | Andorra | 543 | 10.95 | 1473 | 13.51 |
| High body-mass index | Angola | 35257 | 3.97 | 112962 | 7.46 |
| High body-mass index | Antigua and Barbuda | 417 | 9.14 | 1001 | 12.71 |
| High body-mass index | Argentina | 381577 | 10.88 | 678754 | 13.96 |
| High body-mass index | Armenia | 26457 | 13.98 | 43755 | 15.05 |
| High body-mass index | Australia | 227653 | 11.34 | 520988 | 15.97 |
| High body-mass index | Austria | 108956 | 10.82 | 165979 | 12.75 |
| High body-mass index | Azerbaijan | 49724 | 11.85 | 109426 | 14.74 |
| High body-mass index | Bahamas | 1342 | 10.36 | 3985 | 13.87 |
| High body-mass index | Bahrain | 1706 | 10.91 | 10797 | 15.7 |
| High body-mass index | Bangladesh | 517570 | 1.6 | 1657731 | 3.81 |
| High body-mass index | Barbados | 2407 | 10.07 | 4828 | 13.95 |
| High body-mass index | Belarus | 136815 | 12.92 | 169464 | 17.03 |
| High body-mass index | Belgium | 148044 | 9.94 | 209065 | 13.54 |
| High body-mass index | Belize | 733 | 12.4 | 2659 | 15.48 |
| High body-mass index | Benin | 16242 | 7.79 | 45560 | 10.49 |
| High body-mass index | Bermuda | 563 | 11.51 | 1228 | 15.35 |
| High body-mass index | Bhutan | 2536 | 6.32 | 6595 | 8.37 |
| High body-mass index | Bolivia (Plurinational State of) | 27156 | 7.74 | 86285 | 11.33 |
| High body-mass index | Bosnia and Herzegovina | 44163 | 12.34 | 64152 | 15.25 |
| High body-mass index | Botswana | 4942 | 7.72 | 13679 | 12.71 |
| High body-mass index | Brazil | 971836 | 8.66 | 2859147 | 12.39 |
| High body-mass index | Brunei Darussalam | 1028 | 6.27 | 4183 | 11.11 |
| High body-mass index | Bulgaria | 130589 | 14.43 | 141806 | 16.04 |
| High body-mass index | Burkina Faso | 36057 | 3.15 | 78523 | 4.53 |
| High body-mass index | Burundi | 19815 | 2.93 | 39624 | 4.56 |
| High body-mass index | Cabo Verde | 1908 | 6.87 | 3999 | 11.11 |
| High body-mass index | Cambodia | 35451 | 2.8 | 105935 | 4.21 |
| High body-mass index | Cameroon | 40217 | 10.03 | 115436 | 14.13 |
| High body-mass index | Canada | 348719 | 9.61 | 769959 | 11.87 |
| High body-mass index | Central African Republic | 10064 | 3.77 | 20008 | 7.17 |
| High body-mass index | Chad | 23995 | 5.3 | 49356 | 7.03 |
| High body-mass index | Chile | 128926 | 10.59 | 323213 | 14.71 |
| High body-mass index | China | 7585091 | 4.54 | 19818337 | 9.34 |
| High body-mass index | Colombia | 173781 | 8.1 | 609353 | 11.26 |
| High body-mass index | Comoros | 1638 | 4.43 | 4375 | 8.6 |
| High body-mass index | Congo | 9705 | 5.64 | 25673 | 9.98 |
| High body-mass index | Cook Islands | 112 | 16.31 | 251 | 18.95 |
| High body-mass index | Costa Rica | 17172 | 9.21 | 60188 | 11.74 |
| High body-mass index | Croatia | 64134 | 13.2 | 87366 | 16.79 |
| High body-mass index | Cuba | 78483 | 8.84 | 171395 | 12.44 |
| High body-mass index | Cyprus | 8196 | 8.73 | 20996 | 12.93 |
| High body-mass index | Czechia | 143268 | 15.49 | 216289 | 17.11 |
| High body-mass index | C么te d'Ivoire | 34169 | 7.3 | 100032 | 10.48 |
| High body-mass index | Democratic People's Republic of Korea | 163155 | 3.19 | 335881 | 5.7 |
| High body-mass index | Democratic Republic of the Congo | 143116 | 4.17 | 337682 | 8.47 |
| High body-mass index | Denmark | 92143 | 9.44 | 130601 | 11.67 |
| High body-mass index | Djibouti | 1147 | 2.95 | 5690 | 4.62 |
| High body-mass index | Dominica | 487 | 13 | 778 | 15.88 |
| High body-mass index | Dominican Republic | 29515 | 7.39 | 86185 | 11.49 |
| High body-mass index | Ecuador | 45722 | 9.45 | 150898 | 13.77 |
| High body-mass index | Egypt | 249657 | 12.57 | 666139 | 17.88 |
| High body-mass index | El Salvador | 26602 | 10.84 | 60982 | 13.71 |
| High body-mass index | Equatorial Guinea | 1703 | 6.62 | 4875 | 11.56 |
| High body-mass index | Eritrea | 7954 | 2.89 | 22149 | 4.69 |
| High body-mass index | Estonia | 21937 | 13.97 | 27756 | 15.62 |
| High body-mass index | Eswatini | 2419 | 11.77 | 4982 | 16.65 |
| High body-mass index | Ethiopia | 171338 | 3.39 | 379368 | 4.51 |
| High body-mass index | Fiji | 3114 | 13.31 | 7394 | 17.46 |
| High body-mass index | Finland | 64950 | 11.37 | 105221 | 14.42 |
| High body-mass index | France | 727697 | 9.53 | 1219409 | 13.53 |
| High body-mass index | Gabon | 5121 | 9.02 | 10252 | 13.8 |
| High body-mass index | Gambia | 2801 | 7.5 | 8181 | 9.95 |
| High body-mass index | Georgia | 58553 | 12.19 | 52689 | 13.5 |
| High body-mass index | Germany | 1393916 | 12.32 | 1948900 | 14.13 |
| High body-mass index | Ghana | 54206 | 5.09 | 150818 | 9.88 |
| High body-mass index | Greece | 145170 | 11.03 | 214521 | 14.28 |
| High body-mass index | Greenland | 353 | 10.92 | 864 | 11.99 |
| High body-mass index | Grenada | 556 | 8.7 | 1060 | 12.56 |
| High body-mass index | Guam | 719 | 12.3 | 2077 | 14.44 |
| High body-mass index | Guatemala | 33748 | 10.03 | 110424 | 12.84 |
| High body-mass index | Guinea | 27291 | 5.73 | 47796 | 8.03 |
| High body-mass index | Guinea-Bissau | 3190 | 5.89 | 6164 | 8.61 |
| High body-mass index | Guyana | 2878 | 8.99 | 5587 | 11.92 |
| High body-mass index | Haiti | 24598 | 3.62 | 56860 | 6.27 |
| High body-mass index | Honduras | 19532 | 8.73 | 67119 | 11.05 |
| High body-mass index | Hungary | 162221 | 15.47 | 210193 | 17.86 |
| High body-mass index | Iceland | 2848 | 12.38 | 5805 | 14.63 |
| High body-mass index | India | 4522334 | 2.19 | 12447634 | 4.72 |
| High body-mass index | Indonesia | 825233 | 2.63 | 2281418 | 5.4 |
| High body-mass index | Iran (Islamic Republic of) | 268831 | 8.52 | 818878 | 13.92 |
| High body-mass index | Iraq | 64443 | 13.94 | 225749 | 14.7 |
| High body-mass index | Ireland | 42145 | 10.26 | 83160 | 13.43 |
| High body-mass index | Israel | 48875 | 11.61 | 121842 | 13.87 |
| High body-mass index | Italy | 876682 | 9.79 | 1374605 | 12.15 |
| High body-mass index | Jamaica | 14480 | 9.62 | 28033 | 13.61 |
| High body-mass index | Japan | 2148332 | 5.32 | 3720250 | 6.42 |
| High body-mass index | Jordan | 13215 | 13.21 | 82006 | 17.36 |
| High body-mass index | Kazakhstan | 123579 | 12.55 | 185009 | 14.75 |
| High body-mass index | Kenya | 75745 | 4.64 | 225274 | 8.5 |
| High body-mass index | Kiribati | 314 | 12.42 | 685 | 16.54 |
| High body-mass index | Kuwait | 5544 | 12.68 | 32816 | 18.28 |
| High body-mass index | Kyrgyzstan | 29128 | 12.18 | 50385 | 14.34 |
| High body-mass index | Lao People's Democratic Republic | 16022 | 2.89 | 39030 | 5.37 |
| High body-mass index | Latvia | 36910 | 14.81 | 40216 | 16.29 |
| High body-mass index | Lebanon | 19644 | 11.63 | 56443 | 15.18 |
| High body-mass index | Lesotho | 7364 | 10.23 | 9598 | 13.55 |
| High body-mass index | Liberia | 9186 | 9.32 | 17350 | 13.49 |
| High body-mass index | Libya | 17109 | 10.9 | 53173 | 16.89 |
| High body-mass index | Lithuania | 47157 | 13.12 | 59469 | 15.94 |
| High body-mass index | Luxembourg | 5322 | 11.45 | 10531 | 14.03 |
| High body-mass index | Madagascar | 44223 | 3.37 | 101291 | 6.2 |
| High body-mass index | Malawi | 32541 | 3.59 | 64588 | 6.48 |
| High body-mass index | Malaysia | 73206 | 6.22 | 249304 | 9.46 |
| High body-mass index | Maldives | 714 | 4.94 | 2812 | 8.82 |
| High body-mass index | Mali | 30914 | 5.19 | 71853 | 6.61 |
| High body-mass index | Malta | 4322 | 9.34 | 9295 | 13.75 |
| High body-mass index | Marshall Islands | 129 | 13.47 | 314 | 16.72 |
| High body-mass index | Mauritania | 7978 | 9.67 | 18864 | 12.97 |
| High body-mass index | Mauritius | 6056 | 6.55 | 17226 | 9.76 |
| High body-mass index | Mexico | 435876 | 9.47 | 1447378 | 12.05 |
| High body-mass index | Micronesia (Federated States of) | 400 | 13.99 | 720 | 18.12 |
| High body-mass index | Monaco | 645 | 12.59 | 914 | 14.88 |
| High body-mass index | Mongolia | 9185 | 10.01 | 23281 | 11.04 |
| High body-mass index | Montenegro | 6794 | 15.02 | 10619 | 18.53 |
| High body-mass index | Morocco | 133693 | 8.23 | 358805 | 12.22 |
| High body-mass index | Mozambique | 50366 | 4.21 | 97812 | 7.62 |
| High body-mass index | Myanmar | 165341 | 3.48 | 395394 | 5.2 |
| High body-mass index | Namibia | 5665 | 7.13 | 12874 | 12.17 |
| High body-mass index | Nauru | 38 | 16.26 | 53 | 19.5 |
| High body-mass index | Nepal | 105455 | 2.36 | 273868 | 4.42 |
| High body-mass index | Netherlands | 193003 | 9.59 | 331840 | 12.54 |
| High body-mass index | New Zealand | 41154 | 13.3 | 90186 | 16.82 |
| High body-mass index | Nicaragua | 14152 | 10.76 | 50270 | 13.31 |
| High body-mass index | Niger | 21872 | 5.3 | 70335 | 6.74 |
| High body-mass index | Nigeria | 385322 | 5.94 | 884413 | 9.82 |
| High body-mass index | Niue | 19 | 12.72 | 21 | 17.16 |
| High body-mass index | North Macedonia | 18841 | 13.81 | 34450 | 16.42 |
| High body-mass index | Northern Mariana Islands | 147 | 15.35 | 542 | 18.65 |
| High body-mass index | Norway | 61232 | 9.7 | 91572 | 11.87 |
| High body-mass index | Oman | 5531 | 9.02 | 20008 | 16.22 |
| High body-mass index | Pakistan | 494679 | 3.78 | 1259467 | 7.37 |
| High body-mass index | Palau | 81 | 14.72 | 228 | 18.26 |
| High body-mass index | Palestine | 7805 | 12.76 | 24931 | 16.65 |
| High body-mass index | Panama | 14234 | 10.04 | 46824 | 13.07 |
| High body-mass index | Papua New Guinea | 14342 | 7.13 | 42065 | 9.5 |
| High body-mass index | Paraguay | 20950 | 9.12 | 60977 | 11.97 |
| High body-mass index | Peru | 103148 | 8.37 | 316446 | 11.31 |
| High body-mass index | Philippines | 238525 | 4.16 | 724441 | 6.81 |
| High body-mass index | Poland | 499505 | 12.51 | 761639 | 14.97 |
| High body-mass index | Portugal | 140203 | 10.35 | 235340 | 13.64 |
| High body-mass index | Puerto Rico | 30411 | 12.73 | 58523 | 15.79 |
| High body-mass index | Qatar | 1017 | 13.2 | 11332 | 17.99 |
| High body-mass index | Republic of Korea | 326185 | 5.17 | 1083017 | 8.07 |
| High body-mass index | Republic of Moldova | 44020 | 14.68 | 61966 | 18.09 |
| High body-mass index | Romania | 308417 | 13.44 | 377269 | 16.25 |
| High body-mass index | Russian Federation | 2080475 | 12.7 | 2658762 | 16.66 |
| High body-mass index | Rwanda | 25348 | 3.56 | 54580 | 6.27 |
| High body-mass index | Saint Kitts and Nevis | 303 | 9.49 | 692 | 13.34 |
| High body-mass index | Saint Lucia | 713 | 8.58 | 2206 | 11.99 |
| High body-mass index | Saint Vincent and the Grenadines | 559 | 7.31 | 1254 | 10.72 |
| High body-mass index | Samoa | 773 | 16.33 | 1345 | 19.03 |
| High body-mass index | San Marino | 342 | 11.81 | 683 | 14.54 |
| High body-mass index | Sao Tome and Principe | 540 | 8.23 | 978 | 11.59 |
| High body-mass index | Saudi Arabia | 46060 | 12.26 | 187389 | 18.7 |
| High body-mass index | Senegal | 26341 | 6.73 | 66158 | 9.23 |
| High body-mass index | Serbia | 125798 | 13.07 | 169999 | 17.55 |
| High body-mass index | Seychelles | 457 | 8.63 | 1057 | 12.77 |
| High body-mass index | Sierra Leone | 16541 | 5.66 | 31660 | 8.17 |
| High body-mass index | Singapore | 23375 | 5.05 | 95167 | 9.81 |
| High body-mass index | Slovakia | 63186 | 16.33 | 98465 | 18.04 |
| High body-mass index | Slovenia | 25498 | 14.29 | 42516 | 16.73 |
| High body-mass index | Solomon Islands | 1087 | 9.81 | 2916 | 12.94 |
| High body-mass index | Somalia | 19482 | 4.28 | 48717 | 6.35 |
| High body-mass index | South Africa | 195642 | 11.93 | 457627 | 15.22 |
| High body-mass index | South Sudan | 20117 | 2.83 | 32729 | 4.06 |
| High body-mass index | Spain | 537812 | 11.29 | 812142 | 15.64 |
| High body-mass index | Sri Lanka | 82225 | 4.43 | 224498 | 7.37 |
| High body-mass index | Sudan | 77788 | 8.73 | 185818 | 13.45 |
| High body-mass index | Suriname | 2184 | 7.04 | 5828 | 10.46 |
| High body-mass index | Sweden | 115395 | 9.22 | 178855 | 11.58 |
| High body-mass index | Switzerland | 98141 | 10.17 | 173538 | 11.65 |
| High body-mass index | Syrian Arab Republic | 48112 | 11.3 | 133949 | 18.1 |
| High body-mass index | Taiwan (Province of China) | 153216 | 6.09 | 487705 | 10.23 |
| High body-mass index | Tajikistan | 24510 | 11.53 | 55210 | 13.22 |
| High body-mass index | Thailand | 302866 | 3.82 | 1011712 | 8.09 |
| High body-mass index | Timor-Leste | 2059 | 1.65 | 6562 | 2.63 |
| High body-mass index | Togo | 10396 | 6.06 | 34717 | 9.16 |
| High body-mass index | Tokelau | 12 | 12.57 | 13 | 16.5 |
| High body-mass index | Tonga | 518 | 16.65 | 763 | 19.86 |
| High body-mass index | Trinidad and Tobago | 7125 | 10.13 | 17946 | 13.45 |
| High body-mass index | Tunisia | 46768 | 8.62 | 136104 | 13.23 |
| High body-mass index | Turkmenistan | 17730 | 11.43 | 40563 | 12.49 |
| High body-mass index | Tuvalu | 59 | 12.66 | 97 | 16.58 |
| High body-mass index | T眉rkiye | 346423 | 11.19 | 1004905 | 15.56 |
| High body-mass index | Uganda | 53874 | 4.21 | 130919 | 6.9 |
| High body-mass index | Ukraine | 845347 | 14.01 | 882462 | 17.01 |
| High body-mass index | United Arab Emirates | 3553 | 10.46 | 51415 | 17.73 |
| High body-mass index | United Kingdom | 893668 | 11.35 | 1345123 | 14.52 |
| High body-mass index | United Republic of Tanzania | 94822 | 5.92 | 228535 | 10.29 |
| High body-mass index | United States Virgin Islands | 741 | 13.15 | 1622 | 15.31 |
| High body-mass index | United States of America | 3580394 | 12.95 | 7265242 | 16.01 |
| High body-mass index | Uruguay | 43048 | 10.72 | 63532 | 13.17 |
| High body-mass index | Uzbekistan | 108239 | 11.64 | 269714 | 13.68 |
| High body-mass index | Vanuatu | 515 | 8.98 | 1582 | 12.49 |
| High body-mass index | Venezuela (Bolivarian Republic of) | 94306 | 10.02 | 320306 | 12.72 |
| High body-mass index | Viet Nam | 308465 | 1.7 | 851278 | 3.62 |
| High body-mass index | Yemen | 41466 | 5.54 | 125740 | 9.62 |
| High body-mass index | Zambia | 22693 | 4.48 | 58931 | 9.63 |
| High body-mass index | Zimbabwe | 35743 | 6.45 | 63622 | 12 |
| Kidney dysfunction | Afghanistan | 56968 | 0.16 | 82785 | 0.13 |
| Kidney dysfunction | Albania | 21185 | 0.1 | 46857 | 0.12 |
| Kidney dysfunction | Algeria | 112623 | 0.15 | 362960 | 0.15 |
| Kidney dysfunction | American Samoa | 206 | 0.26 | 477 | 0.3 |
| Kidney dysfunction | Andorra | 543 | 0.23 | 1473 | 0.25 |
| Kidney dysfunction | Angola | 35257 | 0.19 | 112962 | 0.18 |
| Kidney dysfunction | Antigua and Barbuda | 417 | 0.08 | 1001 | 0.07 |
| Kidney dysfunction | Argentina | 381577 | 0.16 | 678754 | 0.2 |
| Kidney dysfunction | Armenia | 26457 | 0.18 | 43755 | 0.23 |
| Kidney dysfunction | Australia | 227653 | 0.34 | 520988 | 0.49 |
| Kidney dysfunction | Austria | 108956 | 0.22 | 165979 | 0.27 |
| Kidney dysfunction | Azerbaijan | 49724 | 0.18 | 109426 | 0.2 |
| Kidney dysfunction | Bahamas | 1342 | 0.06 | 3985 | 0.07 |
| Kidney dysfunction | Bahrain | 1706 | 0.14 | 10797 | 0.13 |
| Kidney dysfunction | Bangladesh | 517570 | 0.11 | 1657731 | 0.11 |
| Kidney dysfunction | Barbados | 2407 | 0.07 | 4828 | 0.08 |
| Kidney dysfunction | Belarus | 136815 | 0.13 | 169464 | 0.15 |
| Kidney dysfunction | Belgium | 148044 | 0.22 | 209065 | 0.27 |
| Kidney dysfunction | Belize | 733 | 0.08 | 2659 | 0.08 |
| Kidney dysfunction | Benin | 16242 | 0.18 | 45560 | 0.17 |
| Kidney dysfunction | Bermuda | 563 | 0.06 | 1228 | 0.08 |
| Kidney dysfunction | Bhutan | 2536 | 0.12 | 6595 | 0.16 |
| Kidney dysfunction | Bolivia (Plurinational State of) | 27156 | 0.06 | 86285 | 0.08 |
| Kidney dysfunction | Bosnia and Herzegovina | 44163 | 0.09 | 64152 | 0.12 |
| Kidney dysfunction | Botswana | 4942 | 0.21 | 13679 | 0.23 |
| Kidney dysfunction | Brazil | 971836 | 0.07 | 2859147 | 0.09 |
| Kidney dysfunction | Brunei Darussalam | 1028 | 0.25 | 4183 | 0.24 |
| Kidney dysfunction | Bulgaria | 130589 | 0.11 | 141806 | 0.14 |
| Kidney dysfunction | Burkina Faso | 36057 | 0.16 | 78523 | 0.16 |
| Kidney dysfunction | Burundi | 19815 | 0.09 | 39624 | 0.08 |
| Kidney dysfunction | Cabo Verde | 1908 | 0.19 | 3999 | 0.17 |
| Kidney dysfunction | Cambodia | 35451 | 0.17 | 105935 | 0.18 |
| Kidney dysfunction | Cameroon | 40217 | 0.22 | 115436 | 0.26 |
| Kidney dysfunction | Canada | 348719 | 0.37 | 769959 | 0.43 |
| Kidney dysfunction | Central African Republic | 10064 | 0.2 | 20008 | 0.18 |
| Kidney dysfunction | Chad | 23995 | 0.16 | 49356 | 0.16 |
| Kidney dysfunction | Chile | 128926 | 0.17 | 323213 | 0.22 |
| Kidney dysfunction | China | 7585091 | 0.2 | 19818337 | 0.19 |
| Kidney dysfunction | Colombia | 173781 | 0.04 | 609353 | 0.05 |
| Kidney dysfunction | Comoros | 1638 | 0.09 | 4375 | 0.09 |
| Kidney dysfunction | Congo | 9705 | 0.21 | 25673 | 0.22 |
| Kidney dysfunction | Cook Islands | 112 | 0.25 | 251 | 0.29 |
| Kidney dysfunction | Costa Rica | 17172 | 0.07 | 60188 | 0.08 |
| Kidney dysfunction | Croatia | 64134 | 0.1 | 87366 | 0.13 |
| Kidney dysfunction | Cuba | 78483 | 0.07 | 171395 | 0.08 |
| Kidney dysfunction | Cyprus | 8196 | 0.21 | 20996 | 0.24 |
| Kidney dysfunction | Czechia | 143268 | 0.1 | 216289 | 0.13 |
| Kidney dysfunction | C么te d'Ivoire | 34169 | 0.16 | 100032 | 0.16 |
| Kidney dysfunction | Democratic People's Republic of Korea | 163155 | 0.17 | 335881 | 0.2 |
| Kidney dysfunction | Democratic Republic of the Congo | 143116 | 0.21 | 337682 | 0.2 |
| Kidney dysfunction | Denmark | 92143 | 0.18 | 130601 | 0.22 |
| Kidney dysfunction | Djibouti | 1147 | 0.07 | 5690 | 0.08 |
| Kidney dysfunction | Dominica | 487 | 0.07 | 778 | 0.08 |
| Kidney dysfunction | Dominican Republic | 29515 | 0.06 | 86185 | 0.08 |
| Kidney dysfunction | Ecuador | 45722 | 0.06 | 150898 | 0.09 |
| Kidney dysfunction | Egypt | 249657 | 0.15 | 666139 | 0.16 |
| Kidney dysfunction | El Salvador | 26602 | 0.05 | 60982 | 0.06 |
| Kidney dysfunction | Equatorial Guinea | 1703 | 0.2 | 4875 | 0.22 |
| Kidney dysfunction | Eritrea | 7954 | 0.08 | 22149 | 0.07 |
| Kidney dysfunction | Estonia | 21937 | 0.14 | 27756 | 0.19 |
| Kidney dysfunction | Eswatini | 2419 | 0.25 | 4982 | 0.24 |
| Kidney dysfunction | Ethiopia | 171338 | 0.09 | 379368 | 0.09 |
| Kidney dysfunction | Fiji | 3114 | 0.22 | 7394 | 0.24 |
| Kidney dysfunction | Finland | 64950 | 0.18 | 105221 | 0.25 |
| Kidney dysfunction | France | 727697 | 0.19 | 1219409 | 0.26 |
| Kidney dysfunction | Gabon | 5121 | 0.24 | 10252 | 0.25 |
| Kidney dysfunction | Gambia | 2801 | 0.17 | 8181 | 0.18 |
| Kidney dysfunction | Georgia | 58553 | 0.21 | 52689 | 0.26 |
| Kidney dysfunction | Germany | 1393916 | 0.19 | 1948900 | 0.27 |
| Kidney dysfunction | Ghana | 54206 | 0.09 | 150818 | 0.1 |
| Kidney dysfunction | Greece | 145170 | 0.26 | 214521 | 0.31 |
| Kidney dysfunction | Greenland | 353 | 0.28 | 864 | 0.36 |
| Kidney dysfunction | Grenada | 556 | 0.08 | 1060 | 0.08 |
| Kidney dysfunction | Guam | 719 | 0.2 | 2077 | 0.28 |
| Kidney dysfunction | Guatemala | 33748 | 0.04 | 110424 | 0.06 |
| Kidney dysfunction | Guinea | 27291 | 0.18 | 47796 | 0.18 |
| Kidney dysfunction | Guinea-Bissau | 3190 | 0.17 | 6164 | 0.15 |
| Kidney dysfunction | Guyana | 2878 | 0.07 | 5587 | 0.08 |
| Kidney dysfunction | Haiti | 24598 | 0.06 | 56860 | 0.06 |
| Kidney dysfunction | Honduras | 19532 | 0.05 | 67119 | 0.06 |
| Kidney dysfunction | Hungary | 162221 | 0.1 | 210193 | 0.12 |
| Kidney dysfunction | Iceland | 2848 | 0.2 | 5805 | 0.2 |
| Kidney dysfunction | India | 4522334 | 0.13 | 12447634 | 0.12 |
| Kidney dysfunction | Indonesia | 825233 | 0.18 | 2281418 | 0.2 |
| Kidney dysfunction | Iran (Islamic Republic of) | 268831 | 0.17 | 818878 | 0.19 |
| Kidney dysfunction | Iraq | 64443 | 0.19 | 225749 | 0.16 |
| Kidney dysfunction | Ireland | 42145 | 0.24 | 83160 | 0.27 |
| Kidney dysfunction | Israel | 48875 | 0.24 | 121842 | 0.27 |
| Kidney dysfunction | Italy | 876682 | 0.18 | 1374605 | 0.2 |
| Kidney dysfunction | Jamaica | 14480 | 0.08 | 28033 | 0.09 |
| Kidney dysfunction | Japan | 2148332 | 0.22 | 3720250 | 0.34 |
| Kidney dysfunction | Jordan | 13215 | 0.14 | 82006 | 0.16 |
| Kidney dysfunction | Kazakhstan | 123579 | 0.19 | 185009 | 0.2 |
| Kidney dysfunction | Kenya | 75745 | 0.08 | 225274 | 0.08 |
| Kidney dysfunction | Kiribati | 314 | 0.21 | 685 | 0.2 |
| Kidney dysfunction | Kuwait | 5544 | 0.15 | 32816 | 0.14 |
| Kidney dysfunction | Kyrgyzstan | 29128 | 0.19 | 50385 | 0.18 |
| Kidney dysfunction | Lao People's Democratic Republic | 16022 | 0.22 | 39030 | 0.23 |
| Kidney dysfunction | Latvia | 36910 | 0.14 | 40216 | 0.18 |
| Kidney dysfunction | Lebanon | 19644 | 0.15 | 56443 | 0.18 |
| Kidney dysfunction | Lesotho | 7364 | 0.21 | 9598 | 0.22 |
| Kidney dysfunction | Liberia | 9186 | 0.2 | 17350 | 0.18 |
| Kidney dysfunction | Libya | 17109 | 0.16 | 53173 | 0.15 |
| Kidney dysfunction | Lithuania | 47157 | 0.15 | 59469 | 0.17 |
| Kidney dysfunction | Luxembourg | 5322 | 0.22 | 10531 | 0.22 |
| Kidney dysfunction | Madagascar | 44223 | 0.08 | 101291 | 0.07 |
| Kidney dysfunction | Malawi | 32541 | 0.08 | 64588 | 0.08 |
| Kidney dysfunction | Malaysia | 73206 | 0.27 | 249304 | 0.29 |
| Kidney dysfunction | Maldives | 714 | 0.23 | 2812 | 0.24 |
| Kidney dysfunction | Mali | 30914 | 0.17 | 71853 | 0.18 |
| Kidney dysfunction | Malta | 4322 | 0.2 | 9295 | 0.27 |
| Kidney dysfunction | Marshall Islands | 129 | 0.23 | 314 | 0.22 |
| Kidney dysfunction | Mauritania | 7978 | 0.2 | 18864 | 0.2 |
| Kidney dysfunction | Mauritius | 6056 | 0.23 | 17226 | 0.3 |
| Kidney dysfunction | Mexico | 435876 | 0.05 | 1447378 | 0.06 |
| Kidney dysfunction | Micronesia (Federated States of) | 400 | 0.26 | 720 | 0.24 |
| Kidney dysfunction | Monaco | 645 | 0.26 | 914 | 0.28 |
| Kidney dysfunction | Mongolia | 9185 | 0.23 | 23281 | 0.18 |
| Kidney dysfunction | Montenegro | 6794 | 0.11 | 10619 | 0.12 |
| Kidney dysfunction | Morocco | 133693 | 0.11 | 358805 | 0.11 |
| Kidney dysfunction | Mozambique | 50366 | 0.08 | 97812 | 0.07 |
| Kidney dysfunction | Myanmar | 165341 | 0.21 | 395394 | 0.22 |
| Kidney dysfunction | Namibia | 5665 | 0.22 | 12874 | 0.21 |
| Kidney dysfunction | Nauru | 38 | 0.26 | 53 | 0.23 |
| Kidney dysfunction | Nepal | 105455 | 0.17 | 273868 | 0.2 |
| Kidney dysfunction | Netherlands | 193003 | 0.21 | 331840 | 0.26 |
| Kidney dysfunction | New Zealand | 41154 | 0.49 | 90186 | 0.53 |
| Kidney dysfunction | Nicaragua | 14152 | 0.06 | 50270 | 0.08 |
| Kidney dysfunction | Niger | 21872 | 0.16 | 70335 | 0.15 |
| Kidney dysfunction | Nigeria | 385322 | 0.24 | 884413 | 0.19 |
| Kidney dysfunction | Niue | 19 | 0.28 | 21 | 0.29 |
| Kidney dysfunction | North Macedonia | 18841 | 0.13 | 34450 | 0.15 |
| Kidney dysfunction | Northern Mariana Islands | 147 | 0.27 | 542 | 0.28 |
| Kidney dysfunction | Norway | 61232 | 0.17 | 91572 | 0.2 |
| Kidney dysfunction | Oman | 5531 | 0.14 | 20008 | 0.14 |
| Kidney dysfunction | Pakistan | 494679 | 0.14 | 1259467 | 0.14 |
| Kidney dysfunction | Palau | 81 | 0.28 | 228 | 0.27 |
| Kidney dysfunction | Palestine | 7805 | 0.16 | 24931 | 0.15 |
| Kidney dysfunction | Panama | 14234 | 0.05 | 46824 | 0.06 |
| Kidney dysfunction | Papua New Guinea | 14342 | 0.18 | 42065 | 0.19 |
| Kidney dysfunction | Paraguay | 20950 | 0.08 | 60977 | 0.09 |
| Kidney dysfunction | Peru | 103148 | 0.05 | 316446 | 0.06 |
| Kidney dysfunction | Philippines | 238525 | 0.19 | 724441 | 0.23 |
| Kidney dysfunction | Poland | 499505 | 0.1 | 761639 | 0.12 |
| Kidney dysfunction | Portugal | 140203 | 0.16 | 235340 | 0.19 |
| Kidney dysfunction | Puerto Rico | 30411 | 0.08 | 58523 | 0.11 |
| Kidney dysfunction | Qatar | 1017 | 0.15 | 11332 | 0.13 |
| Kidney dysfunction | Republic of Korea | 326185 | 0.17 | 1083017 | 0.22 |
| Kidney dysfunction | Republic of Moldova | 44020 | 0.16 | 61966 | 0.2 |
| Kidney dysfunction | Romania | 308417 | 0.11 | 377269 | 0.15 |
| Kidney dysfunction | Russian Federation | 2080475 | 0.1 | 2658762 | 0.13 |
| Kidney dysfunction | Rwanda | 25348 | 0.08 | 54580 | 0.08 |
| Kidney dysfunction | Saint Kitts and Nevis | 303 | 0.08 | 692 | 0.07 |
| Kidney dysfunction | Saint Lucia | 713 | 0.07 | 2206 | 0.08 |
| Kidney dysfunction | Saint Vincent and the Grenadines | 559 | 0.07 | 1254 | 0.08 |
| Kidney dysfunction | Samoa | 773 | 0.25 | 1345 | 0.27 |
| Kidney dysfunction | San Marino | 342 | 0.22 | 683 | 0.27 |
| Kidney dysfunction | Sao Tome and Principe | 540 | 0.19 | 978 | 0.19 |
| Kidney dysfunction | Saudi Arabia | 46060 | 0.19 | 187389 | 0.18 |
| Kidney dysfunction | Senegal | 26341 | 0.15 | 66158 | 0.13 |
| Kidney dysfunction | Serbia | 125798 | 0.08 | 169999 | 0.1 |
| Kidney dysfunction | Seychelles | 457 | 0.26 | 1057 | 0.26 |
| Kidney dysfunction | Sierra Leone | 16541 | 0.18 | 31660 | 0.17 |
| Kidney dysfunction | Singapore | 23375 | 0.22 | 95167 | 0.27 |
| Kidney dysfunction | Slovakia | 63186 | 0.11 | 98465 | 0.12 |
| Kidney dysfunction | Slovenia | 25498 | 0.1 | 42516 | 0.13 |
| Kidney dysfunction | Solomon Islands | 1087 | 0.22 | 2916 | 0.22 |
| Kidney dysfunction | Somalia | 19482 | 0.08 | 48717 | 0.07 |
| Kidney dysfunction | South Africa | 195642 | 0.24 | 457627 | 0.25 |
| Kidney dysfunction | South Sudan | 20117 | 0.11 | 32729 | 0.09 |
| Kidney dysfunction | Spain | 537812 | 0.23 | 812142 | 0.29 |
| Kidney dysfunction | Sri Lanka | 82225 | 0.23 | 224498 | 0.25 |
| Kidney dysfunction | Sudan | 77788 | 0.15 | 185818 | 0.15 |
| Kidney dysfunction | Suriname | 2184 | 0.07 | 5828 | 0.08 |
| Kidney dysfunction | Sweden | 115395 | 0.25 | 178855 | 0.26 |
| Kidney dysfunction | Switzerland | 98141 | 0.23 | 173538 | 0.26 |
| Kidney dysfunction | Syrian Arab Republic | 48112 | 0.16 | 133949 | 0.15 |
| Kidney dysfunction | Taiwan (Province of China) | 153216 | 0.29 | 487705 | 0.3 |
| Kidney dysfunction | Tajikistan | 24510 | 0.18 | 55210 | 0.17 |
| Kidney dysfunction | Thailand | 302866 | 0.23 | 1011712 | 0.32 |
| Kidney dysfunction | Timor-Leste | 2059 | 0.19 | 6562 | 0.24 |
| Kidney dysfunction | Togo | 10396 | 0.15 | 34717 | 0.14 |
| Kidney dysfunction | Tokelau | 12 | 0.25 | 13 | 0.29 |
| Kidney dysfunction | Tonga | 518 | 0.24 | 763 | 0.28 |
| Kidney dysfunction | Trinidad and Tobago | 7125 | 0.07 | 17946 | 0.08 |
| Kidney dysfunction | Tunisia | 46768 | 0.15 | 136104 | 0.15 |
| Kidney dysfunction | Turkmenistan | 17730 | 0.2 | 40563 | 0.22 |
| Kidney dysfunction | Tuvalu | 59 | 0.2 | 97 | 0.25 |
| Kidney dysfunction | T眉rkiye | 346423 | 0.14 | 1004905 | 0.15 |
| Kidney dysfunction | Uganda | 53874 | 0.1 | 130919 | 0.08 |
| Kidney dysfunction | Ukraine | 845347 | 0.12 | 882462 | 0.15 |
| Kidney dysfunction | United Arab Emirates | 3553 | 0.18 | 51415 | 0.17 |
| Kidney dysfunction | United Kingdom | 893668 | 0.26 | 1345123 | 0.27 |
| Kidney dysfunction | United Republic of Tanzania | 94822 | 0.08 | 228535 | 0.08 |
| Kidney dysfunction | United States Virgin Islands | 741 | 0.06 | 1622 | 0.09 |
| Kidney dysfunction | United States of America | 3580394 | 0.37 | 7265242 | 0.6 |
| Kidney dysfunction | Uruguay | 43048 | 0.23 | 63532 | 0.25 |
| Kidney dysfunction | Uzbekistan | 108239 | 0.21 | 269714 | 0.21 |
| Kidney dysfunction | Vanuatu | 515 | 0.21 | 1582 | 0.22 |
| Kidney dysfunction | Venezuela (Bolivarian Republic of) | 94306 | 0.05 | 320306 | 0.06 |
| Kidney dysfunction | Viet Nam | 308465 | 0.14 | 851278 | 0.12 |
| Kidney dysfunction | Yemen | 41466 | 0.12 | 125740 | 0.13 |
| Kidney dysfunction | Zambia | 22693 | 0.1 | 58931 | 0.09 |
| Kidney dysfunction | Zimbabwe | 35743 | 0.23 | 63622 | 0.19 |
| Occupational ergonomic factors | Afghanistan | 56968 | 9.76 | 82785 | 8.15 |
| Occupational ergonomic factors | Albania | 21185 | 16.51 | 46857 | 15.79 |
| Occupational ergonomic factors | Algeria | 112623 | 3.9 | 362960 | 3.07 |
| Occupational ergonomic factors | American Samoa | 206 | 4.43 | 477 | 5.39 |
| Occupational ergonomic factors | Andorra | 543 | 4.6 | 1473 | 5.19 |
| Occupational ergonomic factors | Angola | 35257 | 22.85 | 112962 | 14.64 |
| Occupational ergonomic factors | Antigua and Barbuda | 417 | 3.66 | 1001 | 4.19 |
| Occupational ergonomic factors | Argentina | 381577 | 3.93 | 678754 | 4.14 |
| Occupational ergonomic factors | Armenia | 26457 | 17.17 | 43755 | 15.42 |
| Occupational ergonomic factors | Australia | 227653 | 4.23 | 520988 | 4.42 |
| Occupational ergonomic factors | Austria | 108956 | 2.94 | 165979 | 4.15 |
| Occupational ergonomic factors | Azerbaijan | 49724 | 15.07 | 109426 | 12.62 |
| Occupational ergonomic factors | Bahamas | 1342 | 3.65 | 3985 | 4 |
| Occupational ergonomic factors | Bahrain | 1706 | 3.88 | 10797 | 4.87 |
| Occupational ergonomic factors | Bangladesh | 517570 | 10.12 | 1657731 | 9.4 |
| Occupational ergonomic factors | Barbados | 2407 | 3.2 | 4828 | 4.02 |
| Occupational ergonomic factors | Belarus | 136815 | 6.24 | 169464 | 5.88 |
| Occupational ergonomic factors | Belgium | 148044 | 2.22 | 209065 | 3.55 |
| Occupational ergonomic factors | Belize | 733 | 5.93 | 2659 | 6.21 |
| Occupational ergonomic factors | Benin | 16242 | 21.5 | 45560 | 17.86 |
| Occupational ergonomic factors | Bermuda | 563 | 4.85 | 1228 | 4.04 |
| Occupational ergonomic factors | Bhutan | 2536 | 22.62 | 6595 | 18.8 |
| Occupational ergonomic factors | Bolivia (Plurinational State of) | 27156 | 9.98 | 86285 | 11.56 |
| Occupational ergonomic factors | Bosnia and Herzegovina | 44163 | 7.72 | 64152 | 6.22 |
| Occupational ergonomic factors | Botswana | 4942 | 9.96 | 13679 | 10.15 |
| Occupational ergonomic factors | Brazil | 971836 | 7.64 | 2859147 | 6.55 |
| Occupational ergonomic factors | Brunei Darussalam | 1028 | 4.72 | 4183 | 3.96 |
| Occupational ergonomic factors | Bulgaria | 130589 | 5.08 | 141806 | 6.3 |
| Occupational ergonomic factors | Burkina Faso | 36057 | 23.18 | 78523 | 17.63 |
| Occupational ergonomic factors | Burundi | 19815 | 27.49 | 39624 | 27.65 |
| Occupational ergonomic factors | Cabo Verde | 1908 | 11.18 | 3999 | 8.93 |
| Occupational ergonomic factors | Cambodia | 35451 | 23.44 | 105935 | 18.78 |
| Occupational ergonomic factors | Cameroon | 40217 | 23.86 | 115436 | 22.53 |
| Occupational ergonomic factors | Canada | 348719 | 3.57 | 769959 | 3.62 |
| Occupational ergonomic factors | Central African Republic | 10064 | 22.07 | 20008 | 19.49 |
| Occupational ergonomic factors | Chad | 23995 | 22.57 | 49356 | 21.43 |
| Occupational ergonomic factors | Chile | 128926 | 4.58 | 323213 | 5.27 |
| Occupational ergonomic factors | China | 7585091 | 14.77 | 19818337 | 8.89 |
| Occupational ergonomic factors | Colombia | 173781 | 4.32 | 609353 | 4.7 |
| Occupational ergonomic factors | Comoros | 1638 | 18.07 | 4375 | 13.94 |
| Occupational ergonomic factors | Congo | 9705 | 15.75 | 25673 | 11.42 |
| Occupational ergonomic factors | Cook Islands | 112 | 5.09 | 251 | 6.49 |
| Occupational ergonomic factors | Costa Rica | 17172 | 4.26 | 60188 | 4.04 |
| Occupational ergonomic factors | Croatia | 64134 | 6.77 | 87366 | 5.73 |
| Occupational ergonomic factors | Cuba | 78483 | 3.33 | 171395 | 2.94 |
| Occupational ergonomic factors | Cyprus | 8196 | 5.16 | 20996 | 4.73 |
| Occupational ergonomic factors | Czechia | 143268 | 5.28 | 216289 | 5.94 |
| Occupational ergonomic factors | C么te d'Ivoire | 34169 | 19.15 | 100032 | 15 |
| Occupational ergonomic factors | Democratic People's Republic of Korea | 163155 | 10.22 | 335881 | 9.27 |
| Occupational ergonomic factors | Democratic Republic of the Congo | 143116 | 22.1 | 337682 | 18.03 |
| Occupational ergonomic factors | Denmark | 92143 | 4.7 | 130601 | 5.16 |
| Occupational ergonomic factors | Djibouti | 1147 | 17.39 | 5690 | 14.06 |
| Occupational ergonomic factors | Dominica | 487 | 5.43 | 778 | 5.78 |
| Occupational ergonomic factors | Dominican Republic | 29515 | 5.79 | 86185 | 6.06 |
| Occupational ergonomic factors | Ecuador | 45722 | 5.23 | 150898 | 7.07 |
| Occupational ergonomic factors | Egypt | 249657 | 6.76 | 666139 | 7.17 |
| Occupational ergonomic factors | El Salvador | 26602 | 5.78 | 60982 | 5.75 |
| Occupational ergonomic factors | Equatorial Guinea | 1703 | 20.65 | 4875 | 12.11 |
| Occupational ergonomic factors | Eritrea | 7954 | 14.13 | 22149 | 12.1 |
| Occupational ergonomic factors | Estonia | 21937 | 8.23 | 27756 | 5.77 |
| Occupational ergonomic factors | Eswatini | 2419 | 7.95 | 4982 | 6.01 |
| Occupational ergonomic factors | Ethiopia | 171338 | 20.71 | 379368 | 11.24 |
| Occupational ergonomic factors | Fiji | 3114 | 4.72 | 7394 | 3.87 |
| Occupational ergonomic factors | Finland | 64950 | 4.21 | 105221 | 4.3 |
| Occupational ergonomic factors | France | 727697 | 3.4 | 1219409 | 3.97 |
| Occupational ergonomic factors | Gabon | 5121 | 11.26 | 10252 | 7.47 |
| Occupational ergonomic factors | Gambia | 2801 | 14.16 | 8181 | 11.17 |
| Occupational ergonomic factors | Georgia | 58553 | 21.49 | 52689 | 19.77 |
| Occupational ergonomic factors | Germany | 1393916 | 4.02 | 1948900 | 5.08 |
| Occupational ergonomic factors | Ghana | 54206 | 18.69 | 150818 | 14.05 |
| Occupational ergonomic factors | Greece | 145170 | 7.1 | 214521 | 4.33 |
| Occupational ergonomic factors | Greenland | 353 | 8.01 | 864 | 6.53 |
| Occupational ergonomic factors | Grenada | 556 | 5.09 | 1060 | 5.83 |
| Occupational ergonomic factors | Guam | 719 | 4.62 | 2077 | 4.35 |
| Occupational ergonomic factors | Guatemala | 33748 | 10.85 | 110424 | 7.54 |
| Occupational ergonomic factors | Guinea | 27291 | 23.03 | 47796 | 21.37 |
| Occupational ergonomic factors | Guinea-Bissau | 3190 | 19.44 | 6164 | 16.71 |
| Occupational ergonomic factors | Guyana | 2878 | 5.37 | 5587 | 4.69 |
| Occupational ergonomic factors | Haiti | 24598 | 10.17 | 56860 | 12.58 |
| Occupational ergonomic factors | Honduras | 19532 | 6.11 | 67119 | 6.59 |
| Occupational ergonomic factors | Hungary | 162221 | 4.37 | 210193 | 5.5 |
| Occupational ergonomic factors | Iceland | 2848 | 10.06 | 5805 | 7.57 |
| Occupational ergonomic factors | India | 4522334 | 11.58 | 12447634 | 6.82 |
| Occupational ergonomic factors | Indonesia | 825233 | 14.07 | 2281418 | 12.29 |
| Occupational ergonomic factors | Iran (Islamic Republic of) | 268831 | 7.17 | 818878 | 4.48 |
| Occupational ergonomic factors | Iraq | 64443 | 3.51 | 225749 | 3.37 |
| Occupational ergonomic factors | Ireland | 42145 | 4.39 | 83160 | 4.7 |
| Occupational ergonomic factors | Israel | 48875 | 3.51 | 121842 | 4.68 |
| Occupational ergonomic factors | Italy | 876682 | 3.51 | 1374605 | 3.85 |
| Occupational ergonomic factors | Jamaica | 14480 | 5.48 | 28033 | 6.73 |
| Occupational ergonomic factors | Japan | 2148332 | 5.9 | 3720250 | 3.99 |
| Occupational ergonomic factors | Jordan | 13215 | 3 | 82006 | 2.27 |
| Occupational ergonomic factors | Kazakhstan | 123579 | 11.17 | 185009 | 8.6 |
| Occupational ergonomic factors | Kenya | 75745 | 20.92 | 225274 | 19.37 |
| Occupational ergonomic factors | Kiribati | 314 | 2.15 | 685 | 4.57 |
| Occupational ergonomic factors | Kuwait | 5544 | 4.04 | 32816 | 4.57 |
| Occupational ergonomic factors | Kyrgyzstan | 29128 | 13.26 | 50385 | 11.89 |
| Occupational ergonomic factors | Lao People's Democratic Republic | 16022 | 23.56 | 39030 | 16.3 |
| Occupational ergonomic factors | Latvia | 36910 | 7.4 | 40216 | 5.88 |
| Occupational ergonomic factors | Lebanon | 19644 | 4.81 | 56443 | 2.91 |
| Occupational ergonomic factors | Lesotho | 7364 | 11.58 | 9598 | 9.02 |
| Occupational ergonomic factors | Liberia | 9186 | 19.97 | 17350 | 17.2 |
| Occupational ergonomic factors | Libya | 17109 | 4.55 | 53173 | 3.68 |
| Occupational ergonomic factors | Lithuania | 47157 | 8.18 | 59469 | 6.91 |
| Occupational ergonomic factors | Luxembourg | 5322 | 2.76 | 10531 | 3.39 |
| Occupational ergonomic factors | Madagascar | 44223 | 29.54 | 101291 | 24.37 |
| Occupational ergonomic factors | Malawi | 32541 | 25.17 | 64588 | 20.63 |
| Occupational ergonomic factors | Malaysia | 73206 | 6.77 | 249304 | 5.13 |
| Occupational ergonomic factors | Maldives | 714 | 8.52 | 2812 | 6.06 |
| Occupational ergonomic factors | Mali | 30914 | 18.47 | 71853 | 16.39 |
| Occupational ergonomic factors | Malta | 4322 | 2.89 | 9295 | 3.17 |
| Occupational ergonomic factors | Marshall Islands | 129 | 4.79 | 314 | 4.44 |
| Occupational ergonomic factors | Mauritania | 7978 | 13.08 | 18864 | 9.36 |
| Occupational ergonomic factors | Mauritius | 6056 | 3.75 | 17226 | 3.86 |
| Occupational ergonomic factors | Mexico | 435876 | 6.24 | 1447378 | 5.22 |
| Occupational ergonomic factors | Micronesia (Federated States of) | 400 | 4.65 | 720 | 7.01 |
| Occupational ergonomic factors | Monaco | 645 | 3.05 | 914 | 3.48 |
| Occupational ergonomic factors | Mongolia | 9185 | 14.53 | 23281 | 11.66 |
| Occupational ergonomic factors | Montenegro | 6794 | 5.85 | 10619 | 6.17 |
| Occupational ergonomic factors | Morocco | 133693 | 6.28 | 358805 | 9.12 |
| Occupational ergonomic factors | Mozambique | 50366 | 32.01 | 97812 | 30.44 |
| Occupational ergonomic factors | Myanmar | 165341 | 8.69 | 395394 | 8.08 |
| Occupational ergonomic factors | Namibia | 5665 | 13.36 | 12874 | 13.46 |
| Occupational ergonomic factors | Nauru | 38 | 5.49 | 53 | 6.44 |
| Occupational ergonomic factors | Nepal | 105455 | 31.05 | 273868 | 17.36 |
| Occupational ergonomic factors | Netherlands | 193003 | 2.29 | 331840 | 4.06 |
| Occupational ergonomic factors | New Zealand | 41154 | 5.55 | 90186 | 7.16 |
| Occupational ergonomic factors | Nicaragua | 14152 | 7.79 | 50270 | 7.55 |
| Occupational ergonomic factors | Niger | 21872 | 21.91 | 70335 | 20.43 |
| Occupational ergonomic factors | Nigeria | 385322 | 17.61 | 884413 | 14.51 |
| Occupational ergonomic factors | Niue | 19 | 3.67 | 21 | 5.25 |
| Occupational ergonomic factors | North Macedonia | 18841 | 5.86 | 34450 | 5.64 |
| Occupational ergonomic factors | Northern Mariana Islands | 147 | 5.95 | 542 | 5.62 |
| Occupational ergonomic factors | Norway | 61232 | 4.86 | 91572 | 5.36 |
| Occupational ergonomic factors | Oman | 5531 | 5.29 | 20008 | 4.76 |
| Occupational ergonomic factors | Pakistan | 494679 | 7.95 | 1259467 | 11.59 |
| Occupational ergonomic factors | Palau | 81 | 3.74 | 228 | 5.97 |
| Occupational ergonomic factors | Palestine | 7805 | 5.22 | 24931 | 3.92 |
| Occupational ergonomic factors | Panama | 14234 | 5.34 | 46824 | 6.07 |
| Occupational ergonomic factors | Papua New Guinea | 14342 | 6.54 | 42065 | 9.31 |
| Occupational ergonomic factors | Paraguay | 20950 | 5.65 | 60977 | 8.84 |
| Occupational ergonomic factors | Peru | 103148 | 5.34 | 316446 | 6.85 |
| Occupational ergonomic factors | Philippines | 238525 | 11.84 | 724441 | 7.93 |
| Occupational ergonomic factors | Poland | 499505 | 9.28 | 761639 | 6.91 |
| Occupational ergonomic factors | Portugal | 140203 | 7.31 | 235340 | 6.25 |
| Occupational ergonomic factors | Puerto Rico | 30411 | 2.07 | 58523 | 1.47 |
| Occupational ergonomic factors | Qatar | 1017 | 8.29 | 11332 | 8.2 |
| Occupational ergonomic factors | Republic of Korea | 326185 | 9.4 | 1083017 | 6.55 |
| Occupational ergonomic factors | Republic of Moldova | 44020 | 13.5 | 61966 | 7.9 |
| Occupational ergonomic factors | Romania | 308417 | 21.16 | 377269 | 10.86 |
| Occupational ergonomic factors | Russian Federation | 2080475 | 5.91 | 2658762 | 5.13 |
| Occupational ergonomic factors | Rwanda | 25348 | 33.11 | 54580 | 24.22 |
| Occupational ergonomic factors | Saint Kitts and Nevis | 303 | 4.18 | 692 | 4.5 |
| Occupational ergonomic factors | Saint Lucia | 713 | 7.65 | 2206 | 5.6 |
| Occupational ergonomic factors | Saint Vincent and the Grenadines | 559 | 4.47 | 1254 | 4.36 |
| Occupational ergonomic factors | Samoa | 773 | 8.39 | 1345 | 6.25 |
| Occupational ergonomic factors | San Marino | 342 | 4.26 | 683 | 4.26 |
| Occupational ergonomic factors | Sao Tome and Principe | 540 | 8.53 | 978 | 7.3 |
| Occupational ergonomic factors | Saudi Arabia | 46060 | 5.23 | 187389 | 4.48 |
| Occupational ergonomic factors | Senegal | 26341 | 15.78 | 66158 | 11.06 |
| Occupational ergonomic factors | Serbia | 125798 | 10.05 | 169999 | 8.77 |
| Occupational ergonomic factors | Seychelles | 457 | 6.17 | 1057 | 5.56 |
| Occupational ergonomic factors | Sierra Leone | 16541 | 20.35 | 31660 | 16.61 |
| Occupational ergonomic factors | Singapore | 23375 | 2.75 | 95167 | 3.6 |
| Occupational ergonomic factors | Slovakia | 63186 | 4.19 | 98465 | 5.36 |
| Occupational ergonomic factors | Slovenia | 25498 | 4.84 | 42516 | 5.39 |
| Occupational ergonomic factors | Solomon Islands | 1087 | 5.5 | 2916 | 13.53 |
| Occupational ergonomic factors | Somalia | 19482 | 27.24 | 48717 | 26.33 |
| Occupational ergonomic factors | South Africa | 195642 | 4.84 | 457627 | 3.68 |
| Occupational ergonomic factors | South Sudan | 20117 | 23.64 | 32729 | 22.66 |
| Occupational ergonomic factors | Spain | 537812 | 3.42 | 812142 | 3.5 |
| Occupational ergonomic factors | Sri Lanka | 82225 | 7.66 | 224498 | 7.76 |
| Occupational ergonomic factors | Sudan | 77788 | 9.3 | 185818 | 7.99 |
| Occupational ergonomic factors | Suriname | 2184 | 3.3 | 5828 | 3.89 |
| Occupational ergonomic factors | Sweden | 115395 | 2.91 | 178855 | 4.24 |
| Occupational ergonomic factors | Switzerland | 98141 | 6.09 | 173538 | 5.72 |
| Occupational ergonomic factors | Syrian Arab Republic | 48112 | 6.85 | 133949 | 4.33 |
| Occupational ergonomic factors | Taiwan (Province of China) | 153216 | 5.83 | 487705 | 5.01 |
| Occupational ergonomic factors | Tajikistan | 24510 | 12.13 | 55210 | 13.38 |
| Occupational ergonomic factors | Thailand | 302866 | 11.34 | 1011712 | 9.05 |
| Occupational ergonomic factors | Timor-Leste | 2059 | 14.2 | 6562 | 9.37 |
| Occupational ergonomic factors | Togo | 10396 | 20.12 | 34717 | 15.39 |
| Occupational ergonomic factors | Tokelau | 12 | 4.72 | 13 | 5.47 |
| Occupational ergonomic factors | Tonga | 518 | 6.94 | 763 | 6.07 |
| Occupational ergonomic factors | Trinidad and Tobago | 7125 | 3.12 | 17946 | 3.43 |
| Occupational ergonomic factors | Tunisia | 46768 | 5.63 | 136104 | 3.87 |
| Occupational ergonomic factors | Turkmenistan | 17730 | 12.15 | 40563 | 11.06 |
| Occupational ergonomic factors | Tuvalu | 59 | 5.45 | 97 | 6.15 |
| Occupational ergonomic factors | T眉rkiye | 346423 | 9.29 | 1004905 | 4.94 |
| Occupational ergonomic factors | Uganda | 53874 | 30.67 | 130919 | 25 |
| Occupational ergonomic factors | Ukraine | 845347 | 5.9 | 882462 | 5.04 |
| Occupational ergonomic factors | United Arab Emirates | 3553 | 7.81 | 51415 | 7.91 |
| Occupational ergonomic factors | United Kingdom | 893668 | 3.05 | 1345123 | 4.07 |
| Occupational ergonomic factors | United Republic of Tanzania | 94822 | 28.8 | 228535 | 26.31 |
| Occupational ergonomic factors | United States Virgin Islands | 741 | 4.5 | 1622 | 3.73 |
| Occupational ergonomic factors | United States of America | 3580394 | 4.04 | 7265242 | 3.86 |
| Occupational ergonomic factors | Uruguay | 43048 | 3.39 | 63532 | 4.75 |
| Occupational ergonomic factors | Uzbekistan | 108239 | 14.07 | 269714 | 15.1 |
| Occupational ergonomic factors | Vanuatu | 515 | 17.46 | 1582 | 18.21 |
| Occupational ergonomic factors | Venezuela (Bolivarian Republic of) | 94306 | 4.5 | 320306 | 4.69 |
| Occupational ergonomic factors | Viet Nam | 308465 | 7.66 | 851278 | 9.85 |
| Occupational ergonomic factors | Yemen | 41466 | 9.24 | 125740 | 5.6 |
| Occupational ergonomic factors | Zambia | 22693 | 18.96 | 58931 | 17.14 |
| Occupational ergonomic factors | Zimbabwe | 35743 | 18.89 | 63622 | 24.22 |
| Smoking | Afghanistan | 56968 | 3.2 | 82785 | 3.77 |
| Smoking | Albania | 21185 | 14 | 46857 | 14.68 |
| Smoking | Algeria | 112623 | 6.81 | 362960 | 5.26 |
| Smoking | American Samoa | 206 | 7.65 | 477 | 6.94 |
| Smoking | Andorra | 543 | 11.92 | 1473 | 10.24 |
| Smoking | Angola | 35257 | 5.82 | 112962 | 4.86 |
| Smoking | Antigua and Barbuda | 417 | 3.38 | 1001 | 3.46 |
| Smoking | Argentina | 381577 | 9.25 | 678754 | 7.45 |
| Smoking | Armenia | 26457 | 10.6 | 43755 | 9.06 |
| Smoking | Australia | 227653 | 9.79 | 520988 | 5.95 |
| Smoking | Austria | 108956 | 8.85 | 165979 | 9.46 |
| Smoking | Azerbaijan | 49724 | 7.37 | 109426 | 7.91 |
| Smoking | Bahamas | 1342 | 3.26 | 3985 | 3.01 |
| Smoking | Bahrain | 1706 | 7.21 | 10797 | 6.21 |
| Smoking | Bangladesh | 517570 | 7.76 | 1657731 | 6.23 |
| Smoking | Barbados | 2407 | 2.46 | 4828 | 1.98 |
| Smoking | Belarus | 136815 | 9.46 | 169464 | 8.94 |
| Smoking | Belgium | 148044 | 12.48 | 209065 | 9.63 |
| Smoking | Belize | 733 | 4.9 | 2659 | 4.12 |
| Smoking | Benin | 16242 | 3.52 | 45560 | 2.15 |
| Smoking | Bermuda | 563 | 4.7 | 1228 | 4.4 |
| Smoking | Bhutan | 2536 | 4.38 | 6595 | 3.4 |
| Smoking | Bolivia (Plurinational State of) | 27156 | 4.61 | 86285 | 3.67 |
| Smoking | Bosnia and Herzegovina | 44163 | 15.22 | 64152 | 14.33 |
| Smoking | Botswana | 4942 | 8.25 | 13679 | 6.22 |
| Smoking | Brazil | 971836 | 10.16 | 2859147 | 6.63 |
| Smoking | Brunei Darussalam | 1028 | 8.89 | 4183 | 5.51 |
| Smoking | Bulgaria | 130589 | 15.33 | 141806 | 11.32 |
| Smoking | Burkina Faso | 36057 | 2.9 | 78523 | 2.34 |
| Smoking | Burundi | 19815 | 4.78 | 39624 | 3.59 |
| Smoking | Cabo Verde | 1908 | 3.57 | 3999 | 2.41 |
| Smoking | Cambodia | 35451 | 8.25 | 105935 | 6.72 |
| Smoking | Cameroon | 40217 | 3.95 | 115436 | 2.71 |
| Smoking | Canada | 348719 | 10.92 | 769959 | 6.89 |
| Smoking | Central African Republic | 10064 | 4.28 | 20008 | 3.42 |
| Smoking | Chad | 23995 | 4.14 | 49356 | 3.41 |
| Smoking | Chile | 128926 | 7.49 | 323213 | 6.1 |
| Smoking | China | 7585091 | 9.32 | 19818337 | 6.33 |
| Smoking | Colombia | 173781 | 5.58 | 609353 | 3.22 |
| Smoking | Comoros | 1638 | 5.07 | 4375 | 3.7 |
| Smoking | Congo | 9705 | 3.4 | 25673 | 3.98 |
| Smoking | Cook Islands | 112 | 7.51 | 251 | 6.09 |
| Smoking | Costa Rica | 17172 | 5.72 | 60188 | 3.75 |
| Smoking | Croatia | 64134 | 16.97 | 87366 | 13.76 |
| Smoking | Cuba | 78483 | 9.75 | 171395 | 6.81 |
| Smoking | Cyprus | 8196 | 11.74 | 20996 | 10.34 |
| Smoking | Czechia | 143268 | 15.69 | 216289 | 12.85 |
| Smoking | C么te d'Ivoire | 34169 | 4.39 | 100032 | 3.53 |
| Smoking | Democratic People's Republic of Korea | 163155 | 7.23 | 335881 | 6.1 |
| Smoking | Democratic Republic of the Congo | 143116 | 3.28 | 337682 | 2.75 |
| Smoking | Denmark | 92143 | 18.82 | 130601 | 12.14 |
| Smoking | Djibouti | 1147 | 7.35 | 5690 | 6.2 |
| Smoking | Dominica | 487 | 3.25 | 778 | 3.04 |
| Smoking | Dominican Republic | 29515 | 7.29 | 86185 | 5.87 |
| Smoking | Ecuador | 45722 | 5.12 | 150898 | 2.81 |
| Smoking | Egypt | 249657 | 6.79 | 666139 | 6.66 |
| Smoking | El Salvador | 26602 | 2.89 | 60982 | 2.74 |
| Smoking | Equatorial Guinea | 1703 | 4.15 | 4875 | 3.19 |
| Smoking | Eritrea | 7954 | 3.15 | 22149 | 2.09 |
| Smoking | Estonia | 21937 | 10.59 | 27756 | 9.66 |
| Smoking | Eswatini | 2419 | 4.02 | 4982 | 2.53 |
| Smoking | Ethiopia | 171338 | 2.54 | 379368 | 1.86 |
| Smoking | Fiji | 3114 | 7.75 | 7394 | 5.9 |
| Smoking | Finland | 64950 | 9.09 | 105221 | 7.32 |
| Smoking | France | 727697 | 10.18 | 1219409 | 9.23 |
| Smoking | Gabon | 5121 | 2.79 | 10252 | 3.14 |
| Smoking | Gambia | 2801 | 5.52 | 8181 | 3.06 |
| Smoking | Georgia | 58553 | 8.16 | 52689 | 8.35 |
| Smoking | Germany | 1393916 | 12.07 | 1948900 | 9.85 |
| Smoking | Ghana | 54206 | 2.78 | 150818 | 2.52 |
| Smoking | Greece | 145170 | 12.7 | 214521 | 10.68 |
| Smoking | Greenland | 353 | 14.64 | 864 | 11.82 |
| Smoking | Grenada | 556 | 3.42 | 1060 | 3.18 |
| Smoking | Guam | 719 | 6.72 | 2077 | 5.77 |
| Smoking | Guatemala | 33748 | 4.19 | 110424 | 3.04 |
| Smoking | Guinea | 27291 | 4.01 | 47796 | 3.37 |
| Smoking | Guinea-Bissau | 3190 | 2.04 | 6164 | 2.29 |
| Smoking | Guyana | 2878 | 4.13 | 5587 | 3.34 |
| Smoking | Haiti | 24598 | 3.59 | 56860 | 2.52 |
| Smoking | Honduras | 19532 | 4.92 | 67119 | 4.16 |
| Smoking | Hungary | 162221 | 15.1 | 210193 | 11.92 |
| Smoking | Iceland | 2848 | 12.23 | 5805 | 8.01 |
| Smoking | India | 4522334 | 5.8 | 12447634 | 3.33 |
| Smoking | Indonesia | 825233 | 5.51 | 2281418 | 5.13 |
| Smoking | Iran (Islamic Republic of) | 268831 | 7.04 | 818878 | 5.36 |
| Smoking | Iraq | 64443 | 9.99 | 225749 | 7.15 |
| Smoking | Ireland | 42145 | 14.32 | 83160 | 9.11 |
| Smoking | Israel | 48875 | 10.72 | 121842 | 7.55 |
| Smoking | Italy | 876682 | 11.31 | 1374605 | 7.94 |
| Smoking | Jamaica | 14480 | 5.19 | 28033 | 4.05 |
| Smoking | Japan | 2148332 | 9.68 | 3720250 | 5.31 |
| Smoking | Jordan | 13215 | 9.6 | 82006 | 8.28 |
| Smoking | Kazakhstan | 123579 | 7.63 | 185009 | 6.85 |
| Smoking | Kenya | 75745 | 5.17 | 225274 | 3.36 |
| Smoking | Kiribati | 314 | 11.69 | 685 | 12.3 |
| Smoking | Kuwait | 5544 | 7.74 | 32816 | 5.73 |
| Smoking | Kyrgyzstan | 29128 | 7.28 | 50385 | 8.82 |
| Smoking | Lao People's Democratic Republic | 16022 | 7.28 | 39030 | 6.29 |
| Smoking | Latvia | 36910 | 10.16 | 40216 | 8.32 |
| Smoking | Lebanon | 19644 | 11.03 | 56443 | 10.51 |
| Smoking | Lesotho | 7364 | 4.97 | 9598 | 5.41 |
| Smoking | Liberia | 9186 | 3.56 | 17350 | 3.06 |
| Smoking | Libya | 17109 | 6.35 | 53173 | 5.39 |
| Smoking | Lithuania | 47157 | 8.89 | 59469 | 8.06 |
| Smoking | Luxembourg | 5322 | 11.7 | 10531 | 9.47 |
| Smoking | Madagascar | 44223 | 5.05 | 101291 | 2.74 |
| Smoking | Malawi | 32541 | 6.07 | 64588 | 5.47 |
| Smoking | Malaysia | 73206 | 5.34 | 249304 | 3.66 |
| Smoking | Maldives | 714 | 7.9 | 2812 | 5.63 |
| Smoking | Mali | 30914 | 2.76 | 71853 | 3.81 |
| Smoking | Malta | 4322 | 11.27 | 9295 | 7.75 |
| Smoking | Marshall Islands | 129 | 4.75 | 314 | 4.9 |
| Smoking | Mauritania | 7978 | 3.32 | 18864 | 2.63 |
| Smoking | Mauritius | 6056 | 5.27 | 17226 | 4.39 |
| Smoking | Mexico | 435876 | 5.3 | 1447378 | 2.78 |
| Smoking | Micronesia (Federated States of) | 400 | 9.23 | 720 | 9.63 |
| Smoking | Monaco | 645 | 10.23 | 914 | 8.68 |
| Smoking | Mongolia | 9185 | 9.21 | 23281 | 9.35 |
| Smoking | Montenegro | 6794 | 18.84 | 10619 | 16.63 |
| Smoking | Morocco | 133693 | 5.25 | 358805 | 3.92 |
| Smoking | Mozambique | 50366 | 5.37 | 97812 | 4.38 |
| Smoking | Myanmar | 165341 | 9.98 | 395394 | 4.96 |
| Smoking | Namibia | 5665 | 8.51 | 12874 | 5.79 |
| Smoking | Nauru | 38 | 9.66 | 53 | 8.46 |
| Smoking | Nepal | 105455 | 16.01 | 273868 | 9.39 |
| Smoking | Netherlands | 193003 | 12.16 | 331840 | 9.41 |
| Smoking | New Zealand | 41154 | 12.89 | 90186 | 8.81 |
| Smoking | Nicaragua | 14152 | 4.25 | 50270 | 3.42 |
| Smoking | Niger | 21872 | 1.89 | 70335 | 1.66 |
| Smoking | Nigeria | 385322 | 2.2 | 884413 | 1.5 |
| Smoking | Niue | 19 | 5.42 | 21 | 5.33 |
| Smoking | North Macedonia | 18841 | 17.44 | 34450 | 15.13 |
| Smoking | Northern Mariana Islands | 147 | 8.27 | 542 | 6.76 |
| Smoking | Norway | 61232 | 12.28 | 91572 | 7.62 |
| Smoking | Oman | 5531 | 5.18 | 20008 | 3.84 |
| Smoking | Pakistan | 494679 | 7.84 | 1259467 | 5.03 |
| Smoking | Palau | 81 | 5.7 | 228 | 5.41 |
| Smoking | Palestine | 7805 | 7.22 | 24931 | 6.88 |
| Smoking | Panama | 14234 | 4.15 | 46824 | 2.72 |
| Smoking | Papua New Guinea | 14342 | 7.93 | 42065 | 7.18 |
| Smoking | Paraguay | 20950 | 8.81 | 60977 | 6.37 |
| Smoking | Peru | 103148 | 2.82 | 316446 | 2.71 |
| Smoking | Philippines | 238525 | 8.86 | 724441 | 5.62 |
| Smoking | Poland | 499505 | 15.32 | 761639 | 12.13 |
| Smoking | Portugal | 140203 | 9.01 | 235340 | 6.14 |
| Smoking | Puerto Rico | 30411 | 4.58 | 58523 | 3.83 |
| Smoking | Qatar | 1017 | 6.59 | 11332 | 5.98 |
| Smoking | Republic of Korea | 326185 | 8.4 | 1083017 | 5.41 |
| Smoking | Republic of Moldova | 44020 | 7.99 | 61966 | 7.39 |
| Smoking | Romania | 308417 | 13.8 | 377269 | 10.34 |
| Smoking | Russian Federation | 2080475 | 7.4 | 2658762 | 8.13 |
| Smoking | Rwanda | 25348 | 9.01 | 54580 | 9.62 |
| Smoking | Saint Kitts and Nevis | 303 | 2.6 | 692 | 2.25 |
| Smoking | Saint Lucia | 713 | 4.4 | 2206 | 3.26 |
| Smoking | Saint Vincent and the Grenadines | 559 | 3.3 | 1254 | 3.26 |
| Smoking | Samoa | 773 | 8.42 | 1345 | 7.13 |
| Smoking | San Marino | 342 | 10.75 | 683 | 8.04 |
| Smoking | Sao Tome and Principe | 540 | 2.48 | 978 | 2.27 |
| Smoking | Saudi Arabia | 46060 | 4.12 | 187389 | 4.88 |
| Smoking | Senegal | 26341 | 4.13 | 66158 | 2.61 |
| Smoking | Serbia | 125798 | 16.1 | 169999 | 13.88 |
| Smoking | Seychelles | 457 | 5.29 | 1057 | 4.67 |
| Smoking | Sierra Leone | 16541 | 5.04 | 31660 | 4.04 |
| Smoking | Singapore | 23375 | 4.97 | 95167 | 3.36 |
| Smoking | Slovakia | 63186 | 11.78 | 98465 | 10.7 |
| Smoking | Slovenia | 25498 | 12.67 | 42516 | 11.14 |
| Smoking | Solomon Islands | 1087 | 9.91 | 2916 | 9.79 |
| Smoking | Somalia | 19482 | 5 | 48717 | 3.89 |
| Smoking | South Africa | 195642 | 8.44 | 457627 | 4.38 |
| Smoking | South Sudan | 20117 | 5.5 | 32729 | 4.36 |
| Smoking | Spain | 537812 | 8.64 | 812142 | 7.07 |
| Smoking | Sri Lanka | 82225 | 5.29 | 224498 | 2.71 |
| Smoking | Sudan | 77788 | 5.35 | 185818 | 4.1 |
| Smoking | Suriname | 2184 | 6.71 | 5828 | 4.8 |
| Smoking | Sweden | 115395 | 8.74 | 178855 | 8.4 |
| Smoking | Switzerland | 98141 | 11.91 | 173538 | 9.95 |
| Smoking | Syrian Arab Republic | 48112 | 8.85 | 133949 | 6.73 |
| Smoking | Taiwan (Province of China) | 153216 | 7.58 | 487705 | 5.45 |
| Smoking | Tajikistan | 24510 | 7.85 | 55210 | 5.26 |
| Smoking | Thailand | 302866 | 5.76 | 1011712 | 3.71 |
| Smoking | Timor-Leste | 2059 | 6.83 | 6562 | 5.16 |
| Smoking | Togo | 10396 | 6.18 | 34717 | 4.28 |
| Smoking | Tokelau | 12 | 6.65 | 13 | 5.75 |
| Smoking | Tonga | 518 | 8.31 | 763 | 6.49 |
| Smoking | Trinidad and Tobago | 7125 | 5.22 | 17946 | 3.9 |
| Smoking | Tunisia | 46768 | 8.11 | 136104 | 6.21 |
| Smoking | Turkmenistan | 17730 | 7.45 | 40563 | 5.07 |
| Smoking | Tuvalu | 59 | 7.73 | 97 | 7.2 |
| Smoking | T眉rkiye | 346423 | 10.5 | 1004905 | 7.15 |
| Smoking | Uganda | 53874 | 4.33 | 130919 | 3.6 |
| Smoking | Ukraine | 845347 | 8.5 | 882462 | 6.98 |
| Smoking | United Arab Emirates | 3553 | 5.97 | 51415 | 5.91 |
| Smoking | United Kingdom | 893668 | 12.57 | 1345123 | 8.54 |
| Smoking | United Republic of Tanzania | 94822 | 7.63 | 228535 | 5.95 |
| Smoking | United States Virgin Islands | 741 | 4.09 | 1622 | 3.28 |
| Smoking | United States of America | 3580394 | 10.81 | 7265242 | 7.33 |
| Smoking | Uruguay | 43048 | 7.36 | 63532 | 7.27 |
| Smoking | Uzbekistan | 108239 | 4.35 | 269714 | 5.47 |
| Smoking | Vanuatu | 515 | 5.94 | 1582 | 4.23 |
| Smoking | Venezuela (Bolivarian Republic of) | 94306 | 5.8 | 320306 | 3.6 |
| Smoking | Viet Nam | 308465 | 6.82 | 851278 | 5.33 |
| Smoking | Yemen | 41466 | 8.98 | 125740 | 7.54 |
| Smoking | Zambia | 22693 | 6.13 | 58931 | 4.46 |
| Smoking | Zimbabwe | 35743 | 8.05 | 63622 | 6.7 |
| DALYs, disability-adjusted life-years; MSK disorders, musculoskeletal disorders. | | | | | |

| **Supplementary Table 11** Gender difference in global DALYs attributable to main risk factors and their proportions to overall DALYs for MSK disorders among adults aged 50 and over by SDI and geographic regions, 1990-2021 | | | | | | |
| --- | --- | --- | --- | --- | --- | --- |
| Risk factors | Location name | Gender | DALYs | Proportions (%) | DALYs | Proportions (%) |
|  |  |  | 1990 | | 2021 | |
| High body-mass index | Global | Women | 2038573 | 8.17 | 5873725 | 10.66 |
| High body-mass index | Global | Men | 1021883 | 7.02 | 3263624 | 9.79 |
| High body-mass index | High SDI | Women | 792688 | 10.28 | 1728952 | 12.56 |
| High body-mass index | High SDI | Men | 446149 | 10.4 | 1184427 | 13.46 |
| High body-mass index | High-middle SDI | Women | 696376 | 10.48 | 1652346 | 13.17 |
| High body-mass index | High-middle SDI | Men | 297611 | 8.64 | 813998 | 11.37 |
| High body-mass index | Middle SDI | Women | 346890 | 5.88 | 1634210 | 9.82 |
| High body-mass index | Middle SDI | Men | 177272 | 4.8 | 821447 | 8.15 |
| High body-mass index | Low-middle SDI | Women | 150782 | 4.33 | 679847 | 7.34 |
| High body-mass index | Low-middle SDI | Men | 74802 | 3.27 | 341340 | 6.29 |
| High body-mass index | Low SDI | Women | 48565 | 4.1 | 172032 | 6.06 |
| High body-mass index | Low SDI | Men | 24259 | 2.9 | 98765 | 5.35 |
| High body-mass index | Central Asia | Women | 38641 | 12.45 | 78193 | 14.1 |
| High body-mass index | Central Asia | Men | 15524 | 11.34 | 37983 | 13.78 |
| High body-mass index | Central Europe | Women | 143959 | 13.47 | 234863 | 15.87 |
| High body-mass index | Central Europe | Men | 80251 | 14.06 | 137246 | 16.85 |
| High body-mass index | Eastern Europe | Women | 324420 | 13.82 | 475286 | 17.11 |
| High body-mass index | Eastern Europe | Men | 97140 | 11.23 | 178168 | 15.87 |
| High body-mass index | Australasia | Women | 20232 | 11.82 | 60751 | 15.96 |
| High body-mass index | Australasia | Men | 11046 | 11.31 | 37597 | 16.31 |
| High body-mass index | High-income Asia Pacific | Women | 89028 | 5.48 | 217443 | 7 |
| High body-mass index | High-income Asia Pacific | Men | 43316 | 4.96 | 118421 | 6.6 |
| High body-mass index | High-income North America | Women | 308566 | 12.41 | 696900 | 14.6 |
| High body-mass index | High-income North America | Men | 188847 | 13.08 | 557481 | 17.08 |
| High body-mass index | Southern Latin America | Women | 40878 | 11.15 | 100536 | 14.22 |
| High body-mass index | Southern Latin America | Men | 18898 | 10.12 | 50174 | 13.99 |
| High body-mass index | Western Europe | Women | 402216 | 10.9 | 743059 | 13.76 |
| High body-mass index | Western Europe | Men | 217816 | 10.76 | 458513 | 13.5 |
| High body-mass index | Andean Latin America | Women | 9488 | 9.06 | 41430 | 12.37 |
| High body-mass index | Andean Latin America | Men | 5566 | 7.81 | 24924 | 11.4 |
| High body-mass index | Caribbean | Women | 11932 | 9.68 | 36743 | 13.04 |
| High body-mass index | Caribbean | Men | 5998 | 7.3 | 19651 | 10.58 |
| High body-mass index | Central Latin America | Women | 52228 | 10.16 | 217425 | 12.48 |
| High body-mass index | Central Latin America | Men | 25116 | 7.97 | 116217 | 11.27 |
| High body-mass index | Tropical Latin America | Women | 57561 | 9.46 | 233351 | 12.74 |
| High body-mass index | Tropical Latin America | Men | 28501 | 7.42 | 128159 | 11.78 |
| High body-mass index | North Africa and Middle East | Women | 102639 | 11.09 | 410284 | 14.96 |
| High body-mass index | North Africa and Middle East | Men | 58063 | 9.03 | 285519 | 15.07 |
| High body-mass index | South Asia | Women | 84777 | 2.52 | 523375 | 5.28 |
| High body-mass index | South Asia | Men | 43754 | 1.92 | 233075 | 4.06 |
| High body-mass index | East Asia | Women | 233426 | 4.83 | 1283974 | 10.17 |
| High body-mass index | East Asia | Men | 125618 | 4.09 | 636148 | 7.93 |
| High body-mass index | Oceania | Women | 1439 | 10.42 | 4653 | 12.71 |
| High body-mass index | Oceania | Men | 812 | 7.88 | 2864 | 10.29 |
| High body-mass index | Southeast Asia | Women | 44653 | 3.39 | 252253 | 6.7 |
| High body-mass index | Southeast Asia | Men | 19807 | 2.67 | 102955 | 4.78 |
| High body-mass index | Central Sub-Saharan Africa | Women | 5721 | 4.63 | 26332 | 8.56 |
| High body-mass index | Central Sub-Saharan Africa | Men | 3143 | 3.86 | 16674 | 8.18 |
| High body-mass index | Eastern Sub-Saharan Africa | Women | 17668 | 4.77 | 68884 | 7.77 |
| High body-mass index | Eastern Sub-Saharan Africa | Men | 8575 | 3.16 | 34932 | 5.73 |
| High body-mass index | Southern Sub-Saharan Africa | Women | 19997 | 12.38 | 56267 | 15.59 |
| High body-mass index | Southern Sub-Saharan Africa | Men | 7464 | 8.27 | 26433 | 13.12 |
| High body-mass index | Western Sub-Saharan Africa | Women | 29104 | 6.95 | 111723 | 10.14 |
| High body-mass index | Western Sub-Saharan Africa | Men | 16628 | 5.03 | 60489 | 8.64 |
| Occupational ergonomic factors | Global | Women | 1830775 | 7.34 | 3506269 | 6.36 |
| Occupational ergonomic factors | Global | Men | 1815418 | 12.46 | 2957798 | 8.87 |
| Occupational ergonomic factors | High SDI | Women | 240884 | 3.12 | 452060 | 3.28 |
| Occupational ergonomic factors | High SDI | Men | 317454 | 7.4 | 520855 | 5.92 |
| Occupational ergonomic factors | High-middle SDI | Women | 428941 | 6.45 | 699310 | 5.57 |
| Occupational ergonomic factors | High-middle SDI | Men | 410548 | 11.92 | 550976 | 7.7 |
| Occupational ergonomic factors | Middle SDI | Women | 620767 | 10.53 | 1212105 | 7.28 |
| Occupational ergonomic factors | Middle SDI | Men | 518413 | 14.03 | 886623 | 8.8 |
| Occupational ergonomic factors | Low-middle SDI | Women | 339983 | 9.76 | 771815 | 8.33 |
| Occupational ergonomic factors | Low-middle SDI | Men | 377517 | 16.52 | 677354 | 12.48 |
| Occupational ergonomic factors | Low SDI | Women | 198357 | 16.75 | 368378 | 12.98 |
| Occupational ergonomic factors | Low SDI | Men | 189566 | 22.66 | 319388 | 17.31 |
| Occupational ergonomic factors | Central Asia | Women | 33620 | 10.84 | 60096 | 10.84 |
| Occupational ergonomic factors | Central Asia | Men | 30360 | 22.19 | 48103 | 17.45 |
| Occupational ergonomic factors | Central Europe | Women | 83739 | 7.83 | 84647 | 5.72 |
| Occupational ergonomic factors | Central Europe | Men | 81067 | 14.2 | 86246 | 10.59 |
| Occupational ergonomic factors | Eastern Europe | Women | 109857 | 4.68 | 117298 | 4.22 |
| Occupational ergonomic factors | Eastern Europe | Men | 85828 | 9.92 | 86512 | 7.71 |
| Occupational ergonomic factors | Australasia | Women | 5359 | 3.13 | 15909 | 4.18 |
| Occupational ergonomic factors | Australasia | Men | 6564 | 6.72 | 13568 | 5.89 |
| Occupational ergonomic factors | High-income Asia Pacific | Women | 78320 | 4.82 | 115080 | 3.7 |
| Occupational ergonomic factors | High-income Asia Pacific | Men | 79820 | 9.14 | 108092 | 6.02 |
| Occupational ergonomic factors | High-income North America | Women | 62988 | 2.53 | 130882 | 2.74 |
| Occupational ergonomic factors | High-income North America | Men | 94059 | 6.51 | 177826 | 5.45 |
| Occupational ergonomic factors | Southern Latin America | Women | 9404 | 2.56 | 25456 | 3.6 |
| Occupational ergonomic factors | Southern Latin America | Men | 12947 | 6.93 | 22739 | 6.34 |
| Occupational ergonomic factors | Western Europe | Women | 77816 | 2.11 | 178603 | 3.31 |
| Occupational ergonomic factors | Western Europe | Men | 135134 | 6.67 | 202556 | 5.96 |
| Occupational ergonomic factors | Andean Latin America | Women | 4537 | 4.33 | 21362 | 6.38 |
| Occupational ergonomic factors | Andean Latin America | Men | 6069 | 8.52 | 20947 | 9.58 |
| Occupational ergonomic factors | Caribbean | Women | 3113 | 2.52 | 10218 | 3.63 |
| Occupational ergonomic factors | Caribbean | Men | 6297 | 7.67 | 12809 | 6.9 |
| Occupational ergonomic factors | Central Latin America | Women | 16556 | 3.22 | 62647 | 3.6 |
| Occupational ergonomic factors | Central Latin America | Men | 31378 | 9.96 | 81873 | 7.94 |
| Occupational ergonomic factors | Tropical Latin America | Women | 33553 | 5.51 | 90812 | 4.96 |
| Occupational ergonomic factors | Tropical Latin America | Men | 41843 | 10.89 | 101891 | 9.36 |
| Occupational ergonomic factors | North Africa and Middle East | Women | 31104 | 3.36 | 77270 | 2.82 |
| Occupational ergonomic factors | North Africa and Middle East | Men | 80782 | 12.56 | 171012 | 9.03 |
| Occupational ergonomic factors | South Asia | Women | 294160 | 8.73 | 612950 | 6.19 |
| Occupational ergonomic factors | South Asia | Men | 354470 | 15.59 | 586816 | 10.23 |
| Occupational ergonomic factors | East Asia | Women | 643756 | 13.33 | 1120989 | 8.88 |
| Occupational ergonomic factors | East Asia | Men | 501870 | 16.34 | 696860 | 8.69 |
| Occupational ergonomic factors | Oceania | Women | 650 | 4.7 | 2746 | 7.5 |
| Occupational ergonomic factors | Oceania | Men | 880 | 8.54 | 2801 | 10.06 |
| Occupational ergonomic factors | Southeast Asia | Women | 142716 | 10.82 | 364330 | 9.68 |
| Occupational ergonomic factors | Southeast Asia | Men | 98270 | 13.26 | 239645 | 11.12 |
| Occupational ergonomic factors | Central Sub-Saharan Africa | Women | 26277 | 21.27 | 49163 | 15.98 |
| Occupational ergonomic factors | Central Sub-Saharan Africa | Men | 18081 | 22.21 | 36454 | 17.89 |
| Occupational ergonomic factors | Eastern Sub-Saharan Africa | Women | 91480 | 24.71 | 179482 | 20.25 |
| Occupational ergonomic factors | Eastern Sub-Saharan Africa | Men | 71006 | 26.17 | 126337 | 20.72 |
| Occupational ergonomic factors | Southern Sub-Saharan Africa | Women | 9827 | 6.08 | 21654 | 6 |
| Occupational ergonomic factors | Southern Sub-Saharan Africa | Men | 8681 | 9.62 | 14868 | 7.38 |
| Occupational ergonomic factors | Western Sub-Saharan Africa | Women | 71942 | 17.18 | 164675 | 14.94 |
| Occupational ergonomic factors | Western Sub-Saharan Africa | Men | 70013 | 21.18 | 119843 | 17.12 |
| Smoking | Global | Women | 1286736 | 5.16 | 1833878 | 3.33 |
| Smoking | Global | Men | 2222331 | 15.26 | 3482348 | 10.45 |
| Smoking | High SDI | Women | 628255 | 8.15 | 789806 | 5.74 |
| Smoking | High SDI | Men | 682708 | 15.92 | 888444 | 10.1 |
| Smoking | High-middle SDI | Women | 324509 | 4.88 | 469235 | 3.74 |
| Smoking | High-middle SDI | Men | 647922 | 18.81 | 981702 | 13.71 |
| Smoking | Middle SDI | Women | 197242 | 3.35 | 323128 | 1.94 |
| Smoking | Middle SDI | Men | 536504 | 14.52 | 993413 | 9.86 |
| Smoking | Low-middle SDI | Women | 100840 | 2.89 | 189246 | 2.04 |
| Smoking | Low-middle SDI | Men | 277990 | 12.17 | 493535 | 9.09 |
| Smoking | Low SDI | Women | 33515 | 2.83 | 59577 | 2.1 |
| Smoking | Low SDI | Men | 74211 | 8.87 | 121659 | 6.59 |
| Smoking | Central Asia | Women | 5003 | 1.61 | 8056 | 1.45 |
| Smoking | Central Asia | Men | 26591 | 19.43 | 47959 | 17.4 |
| Smoking | Central Europe | Women | 109681 | 10.26 | 130521 | 8.82 |
| Smoking | Central Europe | Men | 136400 | 23.89 | 147754 | 18.14 |
| Smoking | Eastern Europe | Women | 60326 | 2.57 | 89157 | 3.21 |
| Smoking | Eastern Europe | Men | 192298 | 22.23 | 219129 | 19.52 |
| Smoking | Australasia | Women | 16373 | 9.57 | 24902 | 6.54 |
| Smoking | Australasia | Men | 11209 | 11.47 | 14036 | 6.09 |
| Smoking | High-income Asia Pacific | Women | 77242 | 4.75 | 82881 | 2.67 |
| Smoking | High-income Asia Pacific | Men | 159419 | 18.26 | 176491 | 9.83 |
| Smoking | High-income North America | Women | 232436 | 9.35 | 305080 | 6.39 |
| Smoking | High-income North America | Men | 192883 | 13.36 | 280416 | 8.59 |
| Smoking | Southern Latin America | Women | 29704 | 8.1 | 48127 | 6.81 |
| Smoking | Southern Latin America | Men | 18421 | 9.86 | 26757 | 7.46 |
| Smoking | Western Europe | Women | 317048 | 8.59 | 389567 | 7.21 |
| Smoking | Western Europe | Men | 332771 | 16.44 | 385858 | 11.36 |
| Smoking | Andean Latin America | Women | 1789 | 1.71 | 4386 | 1.31 |
| Smoking | Andean Latin America | Men | 4708 | 6.61 | 11590 | 5.3 |
| Smoking | Caribbean | Women | 6479 | 5.26 | 11390 | 4.04 |
| Smoking | Caribbean | Men | 7778 | 9.47 | 12776 | 6.88 |
| Smoking | Central Latin America | Women | 19250 | 3.74 | 36258 | 2.08 |
| Smoking | Central Latin America | Men | 24334 | 7.72 | 48232 | 4.68 |
| Smoking | Tropical Latin America | Women | 50816 | 8.35 | 107468 | 5.87 |
| Smoking | Tropical Latin America | Men | 49760 | 12.95 | 86091 | 7.91 |
| Smoking | North Africa and Middle East | Women | 21075 | 2.28 | 52258 | 1.91 |
| Smoking | North Africa and Middle East | Men | 98234 | 15.28 | 228509 | 12.06 |
| Smoking | South Asia | Women | 94933 | 2.82 | 181141 | 1.83 |
| Smoking | South Asia | Men | 263477 | 11.58 | 425853 | 7.42 |
| Smoking | East Asia | Women | 180158 | 3.73 | 258534 | 2.05 |
| Smoking | East Asia | Men | 550020 | 17.91 | 1043241 | 13.01 |
| Smoking | Oceania | Women | 906 | 6.56 | 2155 | 5.89 |
| Smoking | Oceania | Men | 1021 | 9.91 | 2402 | 8.63 |
| Smoking | Southeast Asia | Women | 40348 | 3.06 | 65363 | 1.74 |
| Smoking | Southeast Asia | Men | 94325 | 12.73 | 221425 | 10.27 |
| Smoking | Central Sub-Saharan Africa | Women | 1223 | 0.99 | 2687 | 0.87 |
| Smoking | Central Sub-Saharan Africa | Men | 6504 | 7.99 | 14275 | 7.01 |
| Smoking | Eastern Sub-Saharan Africa | Women | 8378 | 2.26 | 15568 | 1.76 |
| Smoking | Eastern Sub-Saharan Africa | Men | 23517 | 8.67 | 41609 | 6.83 |
| Smoking | Southern Sub-Saharan Africa | Women | 9128 | 5.65 | 10040 | 2.78 |
| Smoking | Southern Sub-Saharan Africa | Men | 11618 | 12.87 | 16524 | 8.2 |
| Smoking | Western Sub-Saharan Africa | Women | 4439 | 1.06 | 8338 | 0.76 |
| Smoking | Western Sub-Saharan Africa | Men | 17044 | 5.16 | 31422 | 4.49 |
| kidney dysfunction | Global | Women | 25440 | 0.1 | 62631 | 0.11 |
| kidney dysfunction | Global | Men | 48217 | 0.33 | 128752 | 0.39 |
| kidney dysfunction | High SDI | Women | 11505 | 0.15 | 28761 | 0.21 |
| kidney dysfunction | High SDI | Men | 19816 | 0.46 | 57131 | 0.65 |
| kidney dysfunction | High-middle SDI | Women | 6008 | 0.09 | 12425 | 0.1 |
| kidney dysfunction | High-middle SDI | Men | 10064 | 0.29 | 23226 | 0.32 |
| kidney dysfunction | Middle SDI | Women | 5070 | 0.09 | 13827 | 0.08 |
| kidney dysfunction | Middle SDI | Men | 10624 | 0.29 | 30388 | 0.3 |
| kidney dysfunction | Low-middle SDI | Women | 2137 | 0.06 | 5907 | 0.06 |
| kidney dysfunction | Low-middle SDI | Men | 5620 | 0.25 | 13521 | 0.25 |
| kidney dysfunction | Low SDI | Women | 703 | 0.06 | 1673 | 0.06 |
| kidney dysfunction | Low SDI | Men | 2059 | 0.25 | 4414 | 0.24 |
| kidney dysfunction | Central Asia | Women | 368 | 0.12 | 650 | 0.12 |
| kidney dysfunction | Central Asia | Men | 511 | 0.37 | 1065 | 0.39 |
| kidney dysfunction | Central Europe | Women | 627 | 0.06 | 1152 | 0.08 |
| kidney dysfunction | Central Europe | Men | 1039 | 0.18 | 1792 | 0.22 |
| kidney dysfunction | Eastern Europe | Women | 1740 | 0.07 | 2523 | 0.09 |
| kidney dysfunction | Eastern Europe | Men | 1867 | 0.22 | 2939 | 0.26 |
| kidney dysfunction | Australasia | Women | 400 | 0.23 | 1090 | 0.29 |
| kidney dysfunction | Australasia | Men | 586 | 0.6 | 1928 | 0.84 |
| kidney dysfunction | High-income Asia Pacific | Women | 1697 | 0.1 | 4722 | 0.15 |
| kidney dysfunction | High-income Asia Pacific | Men | 3582 | 0.41 | 10356 | 0.58 |
| kidney dysfunction | High-income North America | Women | 5397 | 0.22 | 15914 | 0.33 |
| kidney dysfunction | High-income North America | Men | 9204 | 0.64 | 30762 | 0.94 |
| kidney dysfunction | Southern Latin America | Women | 307 | 0.08 | 743 | 0.11 |
| kidney dysfunction | Southern Latin America | Men | 620 | 0.33 | 1448 | 0.4 |
| kidney dysfunction | Western Europe | Women | 4543 | 0.12 | 7542 | 0.14 |
| kidney dysfunction | Western Europe | Men | 7534 | 0.37 | 14910 | 0.44 |
| kidney dysfunction | Andean Latin America | Women | 35 | 0.03 | 133 | 0.04 |
| kidney dysfunction | Andean Latin America | Men | 66 | 0.09 | 260 | 0.12 |
| kidney dysfunction | Caribbean | Women | 43 | 0.04 | 124 | 0.04 |
| kidney dysfunction | Caribbean | Men | 98 | 0.12 | 260 | 0.14 |
| kidney dysfunction | Central Latin America | Women | 172 | 0.03 | 703 | 0.04 |
| kidney dysfunction | Central Latin America | Men | 241 | 0.08 | 965 | 0.09 |
| kidney dysfunction | Tropical Latin America | Women | 203 | 0.03 | 822 | 0.04 |
| kidney dysfunction | Tropical Latin America | Men | 450 | 0.12 | 1713 | 0.16 |
| kidney dysfunction | North Africa and Middle East | Women | 687 | 0.07 | 2212 | 0.08 |
| kidney dysfunction | North Africa and Middle East | Men | 1673 | 0.26 | 5114 | 0.27 |
| kidney dysfunction | South Asia | Women | 1947 | 0.06 | 5905 | 0.06 |
| kidney dysfunction | South Asia | Men | 5331 | 0.23 | 13357 | 0.23 |
| kidney dysfunction | East Asia | Women | 5408 | 0.11 | 12968 | 0.1 |
| kidney dysfunction | East Asia | Men | 10209 | 0.33 | 27511 | 0.34 |
| kidney dysfunction | Oceania | Women | 12 | 0.09 | 34 | 0.09 |
| kidney dysfunction | Oceania | Men | 36 | 0.34 | 99 | 0.36 |
| kidney dysfunction | Southeast Asia | Women | 1056 | 0.08 | 3473 | 0.09 |
| kidney dysfunction | Southeast Asia | Men | 2875 | 0.39 | 9401 | 0.44 |
| kidney dysfunction | Central Sub-Saharan Africa | Women | 99 | 0.08 | 264 | 0.09 |
| kidney dysfunction | Central Sub-Saharan Africa | Men | 319 | 0.39 | 761 | 0.37 |
| kidney dysfunction | Eastern Sub-Saharan Africa | Women | 146 | 0.04 | 364 | 0.04 |
| kidney dysfunction | Eastern Sub-Saharan Africa | Men | 403 | 0.15 | 844 | 0.14 |
| kidney dysfunction | Southern Sub-Saharan Africa | Women | 176 | 0.11 | 438 | 0.12 |
| kidney dysfunction | Southern Sub-Saharan Africa | Men | 414 | 0.46 | 936 | 0.46 |
| kidney dysfunction | Western Sub-Saharan Africa | Women | 375 | 0.09 | 854 | 0.08 |
| kidney dysfunction | Western Sub-Saharan Africa | Men | 1160 | 0.35 | 2330 | 0.33 |
| DALYs, disability-adjusted life-years; MSK disorders, musculoskeletal disorders; SDI, Socio-demographic Index. | | | | | | |
